# Bichromophoric Ruthenium Complexes for Photocatalyzed Late-Stage Synthesis of Trifluoromethylated Indolizines

**DOI:** 10.1021/acs.joc.5c00319

**Published:** 2025-05-05

**Authors:** Kevin Klaus Stefanoni, Matthias Schmitz, Johanna Treuheit, Christoph Kerzig, René Wilhelm

**Affiliations:** † Institute of Organic Chemistry, Clausthal University of Technology, Leibnizstr. 6, 38678 Clausthal-Zellerfeld, Germany; ‡ Department of Chemistry, Johannes Gutenberg University Mainz, Duesbergweg 10-14, 55128 Mainz, Germany

## Abstract

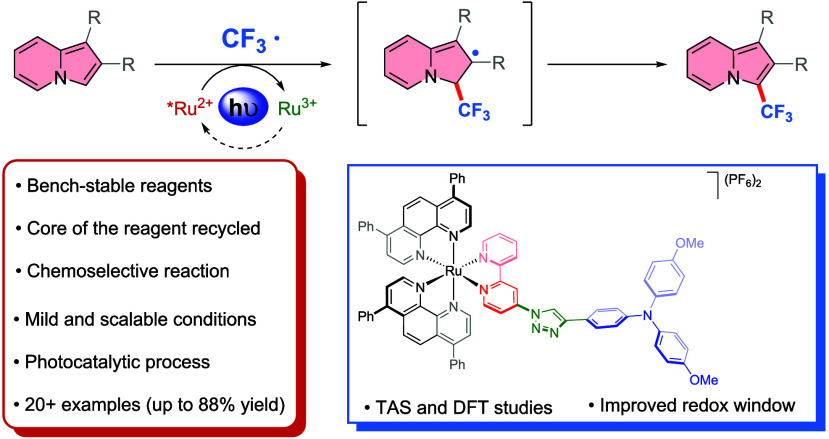

Indolizines are a promising class of biologically active compounds. However, photocatalytic methods for their selective derivatization are scarce in the literature. Herein, a mild, simple, and chemoselective protocol for the synthesis of 3-(trifluoromethyl)indolizine has been developed. The desired products were obtained in good to excellent yields and can be easily obtained on a gram scale. By tuning the redox properties of a Ru-based photocatalyst, it is possible to achieve competitive yields and further apply the optimized conditions to a broad variety of substrates. This method tolerates many functional groups and, therefore, can be used for late-stage functionalization. Our combined theoretical and spectroscopic findings revealed that the superior dyad-like ruthenium catalyst developed in this study has a completely different electronic nature of both key species that are crucial for efficient photoredox catalysis compared to commonly used homoleptic ruthenium complexes.

## Introduction

**Figure 1:**
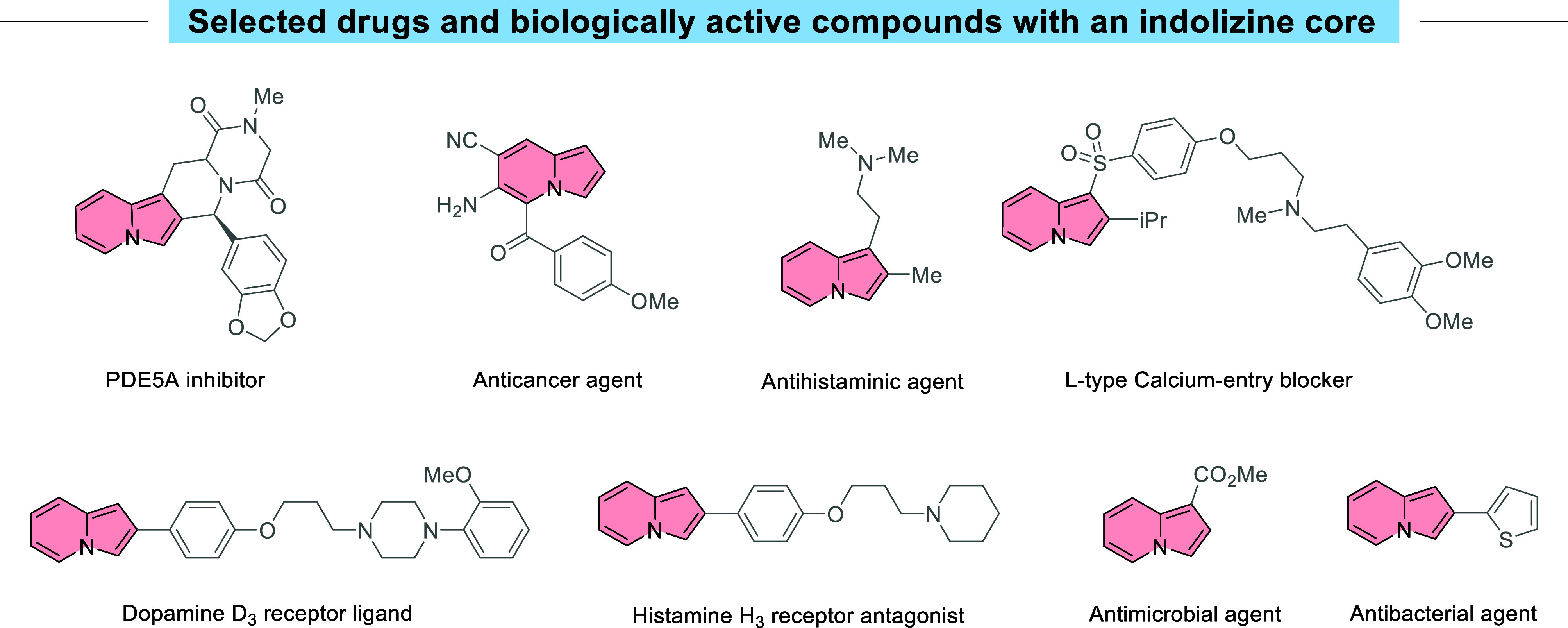
Drugs and bioactive molecules with an indolizine scaffold.

As an important class of nitrogen-containing heterocyclic compounds, indolizine derivatives have a wide range of biologically relevant properties, being a bioisostere of the indole nucleus.^1,2^ Although their biological potential is still largely unexplored, many speculations have arisen that indolizine analogues of certain biologically active indoles may confer similar or even better biological activities (Figure 1).^3^ Indolizine-containing molecules are also used in dyes and fluorescent materials, one of the most notable examples being Seoul-Fluor.^4^ Due to their relevance, numerous synthetic protocols for both synthesis and functionalization have been proposed over the last decades.^5−13^

Trifluoromethylated compounds are of importance in the pharmaceutical and agrochemical industry because they possess dramatically modified physical and biological properties compared to the parent molecules, such as solubility, lipophilicity, and catabolic stability.^14−16^ The introduction of CF_3_ groups is thus actively pursued, and new methods have been developed over the past decades.^17−20^ In the literature, well-established methods use cross-coupling reactions promoted by transition metals.^19^ The drawbacks are generally the use of toxic metals, harsh reaction conditions, and the required prefunctionalization of the substrate, which limit the applicability to more delicate substrates like drugs. With the introduction of photoredox catalysis protocols, it became possible to overcome those limits and open the door to further developments.^21−26^

**Scheme 1:**
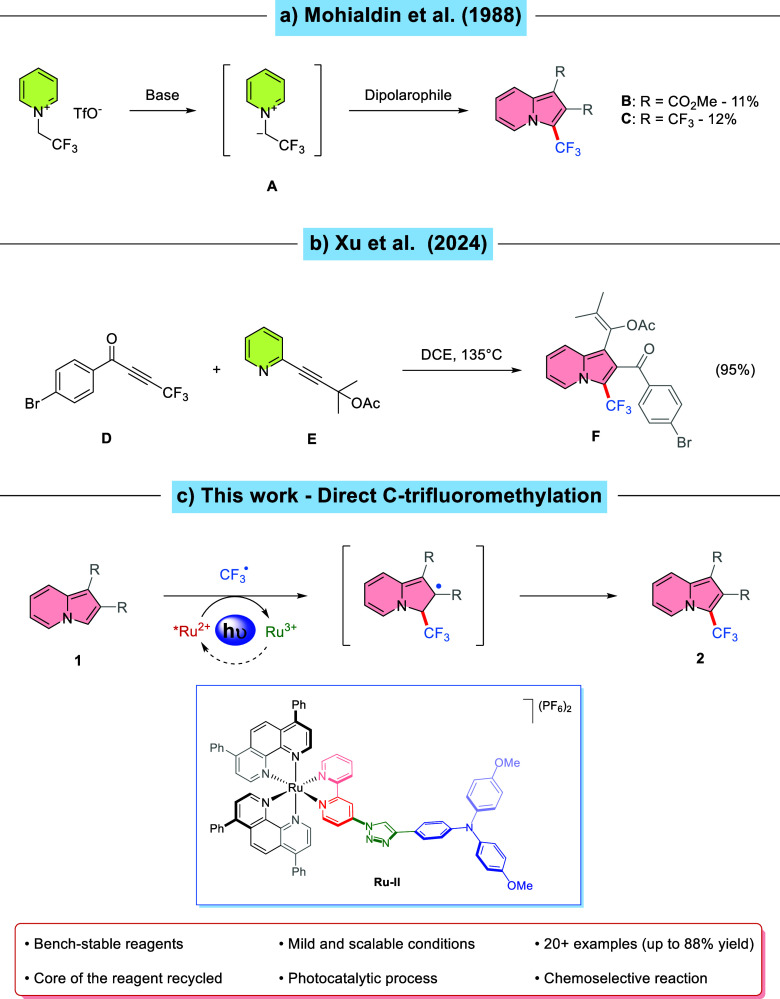
Previous and Current Protocols for the Synthesis of 3-(Trifluoromethyl)indolizines

The first reported synthesis of a 3-(trifluoromethyl)indolizine was described in 1988 by Banks and Mohialdin (Scheme 1a).^27^ Their strategy involved a 1,3-dipolar cycloaddition between 2,2,2-trifluoro-1-(pyridin-1-ium-1-yl)ethan-1-ide **A** generated in situ and a suitable alkyne as a dipolarophile; with this method, indolizine **B** and **C** were synthesized in yields of 11% and 12%, respectively. Because of the low-yielding synthesis required to install a CF_3_ group on the indolizine scaffold, interest in these analogues faded. In the past few years, with the advent of new methodologies such as photoredox catalysis and organic electrochemistry, numerous groups started to directly functionalize similar scaffolds such as imidazo[1,2-*a*]pyridines^28−30^ and 2*H*-indazoles^31,32^ with CF_3_ groups. More recently, Xu and Hoye developed a protocol for the synthesis of indolizines through the net [3 + 2] cycloaddition between bench-stable alkynes and 2-ethynylpyridine derivatives (Scheme 1b).^33^ 3-(Trifluoromethyl)indolizine **F** was prepared by reacting 4,4,4-trifluoro-1-phenylbut-2-yn-1-one **D** and 2-alkynylpyridine **E** at 135 °C in DCE in a 95% yield. Despite the excellent yield for this compound, no other indolizine derivatives have been prepared, and product **F** stands so far as the only example. Moreover, highly functionalized starting materials are required for this reaction, and to this day, no common method for installing a CF_3_ group on the indolizine moiety has been proposed.

**Scheme 2:**
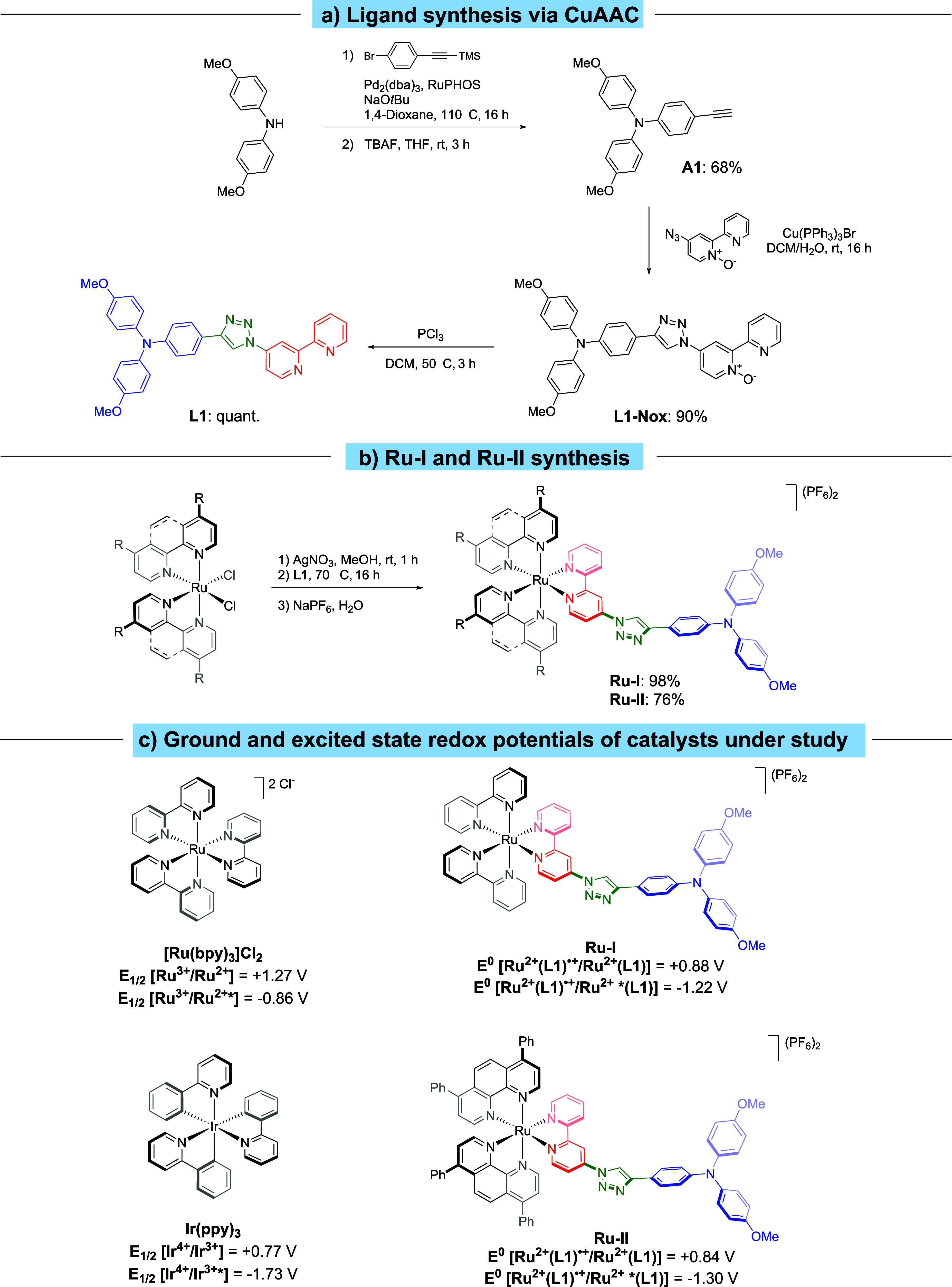
(a) Ligand **L1** Synthesis via Copper Alkyne–Azide Cycloaddition (CuAAC); (b) **Ru–I** and **Ru–II** Complex Synthesis; (c) Employed Photoredox Catalysts and Pertinent Ground-State as Well as Excited-State Redox Potentials in V vs Ag|AgCl_(3M)_; Values of [Ru(bpy)_3_]Cl_2_ and Ir(ppy)_3_ Are from the Literature^39^ and Are vs Ag|AgCl_(3M)_

Within the last years, visible-light photoredox catalysis has been established as a straightforward and effective synthetic method for the synthesis and functionalization of organic molecules, in which ruthenium- and iridium-based photocatalysts have played a major role.^24,25,34−36^ Metal complexes such as polypyridyl ruthenium(II) compounds (e.g., [Ru(bpy)_3_]^2+^) have been extensively explored as catalysts due to their promising photochemical and electrochemical properties such as their broad absorption bands, high stability of the photoexcited state, and long lifetime (1100 ns in deaerated MeCN).^37^ Moreover, modifications of the [Ru(bpy)_3_]^2+^ ligands lead to metal complexes with enhanced redox properties and with improved oxidizing or reducing power.^38−42^ This allows expanding the scope of substrates and types of synthetic transformations achievable, by carefully designing new ligands and by controlling their electronic properties. Ru^II^ polypyridine complexes bearing 2,2′-bipyridine ligands with a push system connected via a π-bridge have already been successfully studied for photodynamic therapy (PDT)^43−45^ and explored with other light-harvesting antennas.^46−48^ However, they are still less explored as catalysts in organic synthesis. Hence, we report here, based on our previous studies,^49−51^ the development of a variety of nonsymmetrical 2,2′-bipyridine ligands bearing different electron-rich motifs (e.g., triphenylamines) connected via a π-bridge (e.g., a 1,2,3-triazole unit) to the 2,2′-bipyridine for ruthenium complexes. 1,2,3-Triazoles have been explored less extensively as a π-bridge for dyes and light-harvesting units bearing a 2,2′-bipyridine scaffold.^52−57^ 1,4-Disubstituted-1,2,3-triazoles are 5-membered aromatic heterocycles that possess unique chemical stability. They are typically not cleaved by hydrolysis or oxidation due to their aromaticity, as indicated by the Bird index being comparable to that of thiophene.^58,59^ Moreover, numerous simple and high-yielding protocols for the synthesis of 1,4-disubstituted-1,2,3-triazoles have been proposed over the last years, one on top of the others being the copper alkyne–azide cycloaddition (CuAAC).^60−62^ Therefore, we investigate here the triazoles’ ability to act as the π-linking bridge within our 2,2′-bipyridine ligands for the new photoredox catalysts **Ru–I** and **Ru–II**, whose synthesis is described in Scheme 2.

Thus, to continue our ongoing effort to develop efficient and simple photocatalysts and protocols for the direct functionalization of heterocycles,^49^ we describe herein a photocatalyzed radical trifluoromethylation approach for the synthesis of variously substituted 3-(trifluoromethyl)indolizine derivatives **2**, mediated by TTCF_3_OTf as a CF_3_ radical source and new dyad-like complexes **Ru–I** and **Ru–II** as photoredox catalysts with blue LEDs exceeding the performance of its parent complex (Scheme 1c).

## Results and Discussion

First, 2-phenylindolizine-1-carbonitrile (**1a**) was investigated as a model compound for the optimization of the reaction conditions, in the presence of the commercially available [Ru(bpy)_3_]Cl_2_·6H_2_O as a photoredox catalyst and Umemoto II as a CF_3_ radical source (Table 1). The initial studies were focused on the synthesis of indolizine **2a** by irradiation of the reaction mixture in acetone. According to the UV–vis spectra of the potential catalysts (see Figures S5–S7), the experiments can be conducted using a 455 nm LED (3 W). Under these conditions, product **2a** was obtained in a scarce yield (Table 1, entry 1). Without a catalyst, the reaction gave a similar yield of 20% of the product **2a** (Table 1, entry 2). By running the reaction in darkness, no product formation was observed, proving that a light-mediated reaction is needed for obtaining a high yield (Table 1, entry 3). The effect of the solvent system was further investigated in the reaction with indolizine **1a**. By switching from acetone to acetonitrile as a polar aprotic solvent (Table 1, entries 1 and 5), a similar result was obtained. By changing the CF_3_ radical source to TTCF_3_OTf^63^ and the solvent to DMF, it was possible to slightly improve the yield of the desired product further. By employing [Ru(bpy)_3_]Cl_2_·6H_2_O as a catalyst for the reaction, a low yield of 36% was obtained (Table 1, entry 6). A similar result was obtained without the photoredox catalyst (Table 1, entry 7), as was already observed for the Umemoto II reagent (Table 1, entries 1 and 2). This indicates that [Ru(bpy)_3_]Cl_2_·6H_2_O is not active in the reaction, and a background reaction via electron donor–acceptor (EDA) complex formation occurred.^63,64^ Since the chloride counter anions of complex [Ru(bpy)_3_]Cl_2_·6H_2_O can have a negative effect in catalytic reactions,^65^ complex [Ru(bpy)_3_](PF_6_)_2_ was applied in the reaction which resulted in a yield of 66% (Table 1, entry 8). By changing the photoredox catalyst to the commercially available Ir(ppy)_3_, it was possible to achieve a yield of 80% (Table 1, entry 9). Ir(ppy)_3_ in its excited state is a stronger reducing agent compared to simple [Ru(bpy)_3_]Cl_2_·6H_2_O (*E*_1/2_^4+/3+^* = −1.73 V vs SCE vs *E*_1/2_^3+/2+^* = −0.86 V vs SCE), by virtue of its three strongly electron-donating cyclometalated 2-phenylpyridine ligands.^35^ Considering the price of iridium on the stock market nowadays (4650 $/oz) versus the ruthenium one (485 $/oz), it was decided to focus on [Ru(bpy)_3_](PF_6_)_2_ and its ligand modifications.^66^ By screening different Ru-photoredox catalysts prepared in our laboratories, [Ru(bpy)_2_(dMeOTPA-Tz-bpy)](PF_6_)_2_ (**Ru–I**) and [Ru(dpp)_2_(dMeOTPA-Tz-bpy)](PF_6_)_2_ (**Ru–II**) were identified for their activity in the reaction. **Ru–II** proved to be a superior catalyst compared to **Ru–I** and [Ru(bpy)_3_](PF_6_)_2_ (Table 1, entries 8, 10, 11), and it was selected for studying the scope of the reaction with further analogues. The synergistic role of the triarylamine-containing ligand of **Ru–II** was also highlighted in a control experiment, in which the homoleptic complex [Ru(dpp)_3_](PF_6_)_2_ (**Ru–III**) and the dMeOTPA-Tz-bpy ligand were added as the catalytic system, achieving only a 55% yield (Table 1, entry 14). A cost estimation of the prepared Ru–II showed that our catalyst was still ca. 10 times cheaper than the commercial Ir(ppy)_3_ catalyst, comparing €/mol (for a detailed calculation, see Supporting Information page S5 and S6)

**Table 1:**
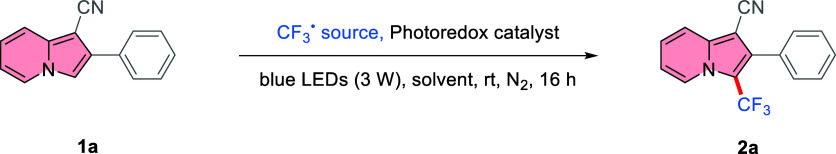
Optimization Studies for the Synthesis of Indolizine **2a**^a^

entry	photoredox catalyst	solvent	CF_3_^•^ source	yield
1	[Ru(bpy)_3_]Cl_2_·6H_2_O	acetone	Umemoto II	25%
2		acetone	Umemoto II	20%
3^b^	[Ru(bpy)_3_]Cl_2_·6H_2_O	acetone	Umemoto II	0%
4^c^	[Ru(bpy)_3_]Cl_2_·6H_2_O	acetone	Umemoto II	12%
5	[Ru(bpy)_3_]Cl_2_·6H_2_O	MeCN	Umemoto II	24%
6	[Ru(bpy)_3_]Cl_2_·6H_2_O	DMF	TTCF_3_OTf	36%
7		DMF	TTCF_3_OTf	36%
8	[Ru(bpy)_3_](PF_6_)_2_	DMF	TTCF_3_OTf	66%
9	Ir(ppy)_3_	DMF	TTCF_3_OTf	80%
10	**Ru–I**	DMF	TTCF_3_OTf	61%
11	**Ru–II**	DMF	TTCF_3_OTf	82% (76%)
12	**Ru–II**	DMF	Umemoto II	70%
13	**Ru–III**	DMF	TTCF_3_OTf	52%
14	**Ru–III** + **L1**	DMF	TTCF_3_OTf	55%

a Reactions were carried out under a N_2_ atmosphere at 25 °C for 16 h using **1a** (0.2 mmol), CF_3_^•^ source (1.25 equiv), PC (2.5 mol %), solvent (0.1 M of **1a**), irradiated by blue LEDs (3 W). Isolated yields after column chromatography are given in parentheses.

b In darkness.

c Under air.

With the optimized conditions in hand, the scope and limitations were investigated next by examining a variety of different indolizine scaffolds with catalyst **Ru–II** (Table 2). For instance, under the optimized conditions, indolizines **1c** (R^1^=CN, R^2^=4-MeO), **1d** (R^1^=H, R^2^=4-MeO), and **1e** (R^1^=CO_2_Me, R^2^=4-MeO) afforded the desired trifluoromethylated product in excellent yields. Indolizine **1f** (R^1^=CO_2_Et, R^2^=4-MeO) instead was not able to deliver the same performance, as product **2f** was recovered in a 19% yield. When 2-phenylindolizines were applied, no significant decrease in yields was observed, and products **2b** and **2j** were obtained in 81% and 75% yields, respectively. For indolizine **1b** (R^1^, R^2^=H), it was not possible to separate the desired trifluoromethylated indolizine from the thianthrene (TT) byproduct of the reaction. Several attempts to remove TT also chemically were made, but those led only to the decomposition of indolizine **2b**. The isolation of the different analogues of indolizines, such as **2h**, **2p**, and **2q**, proved to be challenging from the reaction mixture. Only upon removal of TT as thianthrene *S*-oxide (TTSO) by using either *m*CPBA or Fe(NO_3_)_3_·9H_2_O/NaBr/AcOH systems it was possible to obtain these compounds pure in good to excellent yields of 63%, 87%, and 80%, respectively. While keeping R^2^=Br and introducing a CN group on position 1, indolizine **2g** was effectively obtained in a higher yield compared to the nonsubstituted indolizine **2h**. Free OH groups were also tolerated under the optimized conditions, and both indolizine **2l** and **2m** were isolated in good yields (60% and 59%, respectively), regardless of the substituent on position 1. Indolizines **2k** and **2o** bearing CF_3_ electron-withdrawing groups on the 2-phenyl substituent were also isolated in slightly lower yields, i.e., 70% and 78%, respectively, when compared to the unsubstituted counterparts, namely, **2p** and **2q**. The protocol was also suitable for more sterically hindered substrates with substituents on the ortho position of the 2-phenyl ring, like indolizines **1n** and **1r**. While the latter afforded the desired trifluoromethylated product **2r** in a 71% yield (only 9% less compared to indolizine **2a** with the MeO group on the 4 position), indolizine **1n** failed to be fully converted into product **2n**, which was isolated with an average yield of 50%. The effect of the phenyl ring on position 2 was confirmed to be crucial, as indolizine **2i** was isolated in only a 38% yield. In the case of indolizine **1s**, where two carboxylate groups are on positions 1 and 2, the desired product **2s** was isolated with a 77% yield alongside indolizine **2s′** in a 4:1 ratio. This selectivity issue might be caused by the combined electron-withdrawing effect of both ester groups on position 3 of the indolizine, which becomes less prone to being attacked by CF_3_ radicals. By placing a phenyl substituent on position 1 as in indolizine **1t**, product **2t** was obtained in an excellent yield of 86%. At last, the reactions with indolizines bearing a thiophene ring on position 2 were carried out. In the case of indolizines **1u** and **1w**, both desired products were isolated in very good to excellent yields of 80% and 86%, respectively. The lack of a substituent at position 1 (as exemplified by indolizine **1v**) proved to be detrimental to the reaction outcome, resulting in a reduction of the yield to 55%. Product **2v**, like product **2b**, was also difficult to isolate, as TT removal (e.g., by oxidation to TTSO) led only to the decomposition of the desired trifluoromethylated product.

**Table 2:**
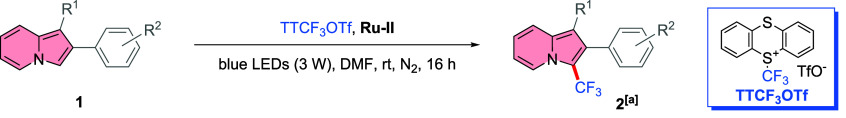

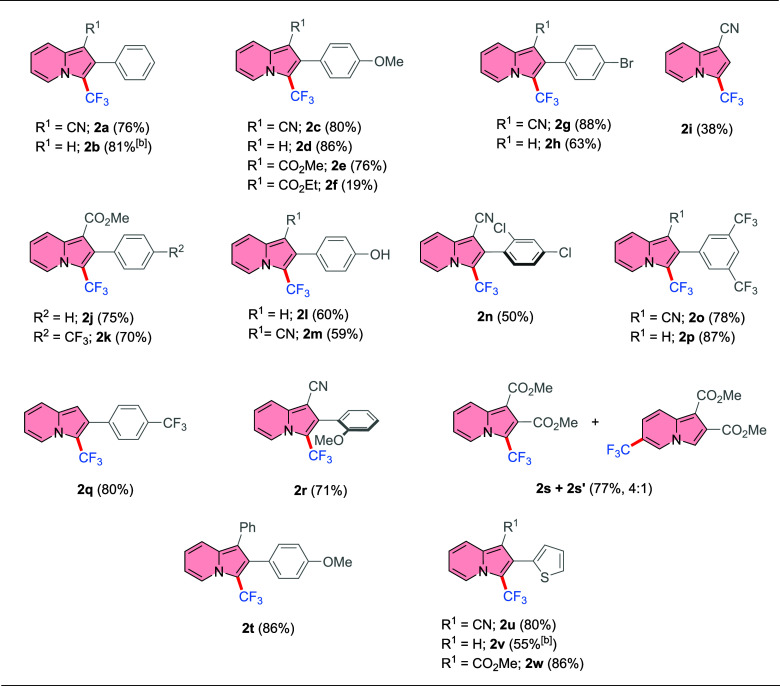
Photocatalytic Trifluoromethylation of Indolizines **1**^a^^,^^b^

a Reactions were carried out under a N_2_ atmosphere at 25 °C for 16 h using **1** (0.2 mmol), TTCF_3_OTf (1.25 equiv), PC (2.5 mol %), DMF (0.1 M), irradiated by blue LEDs (3 W). Isolated yields after column chromatography are given in parentheses.

b Yield determined by ^1^H NMR using CH_2_Br_2_ as the internal standard.

**Scheme 3:**
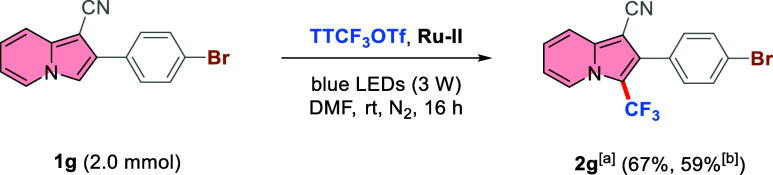
Reaction Scale-up^,^ Reactions were carried out under a N_2_ atmosphere at 25 °C for 16 h using **1** (0.2 mmol), TTCF_3_OTf (1.1 equiv), PC (1.0 mol %), DMF (0.2 M), irradiated by blue LEDs (3 W). Isolated yields after column chromatography are given in parentheses. 455–460 nm blue LEDs (25 W) were used.

The synthetic applicability of this protocol was explored in a scale-up experiment, where the amount of starting material **1g** was increased by 10-fold. Notably, only by slightly changing the reaction parameters (lowering solvent volume resulting in a concentration of 0.2 M for 1**g**, catalyst loading to 1.0 mol %, and CF_3_ source loading to 1.1 equiv)—from the optimal conditions outlined in Table 1, entry 11 product **2g** was obtained in just a slightly lower yield (67% vs 88%). When a more powerful LED (25 W) was employed, the desired product was isolated in 59%, showing that a further increase in the light intensity was not positively affecting the course of the reaction. A possible background reaction was not accelerated, and most likely a possible decomposition of the catalyst due to localized hot spots close to the surface of the reaction vessel decreased the yield (Scheme 3).

**Scheme 4:**
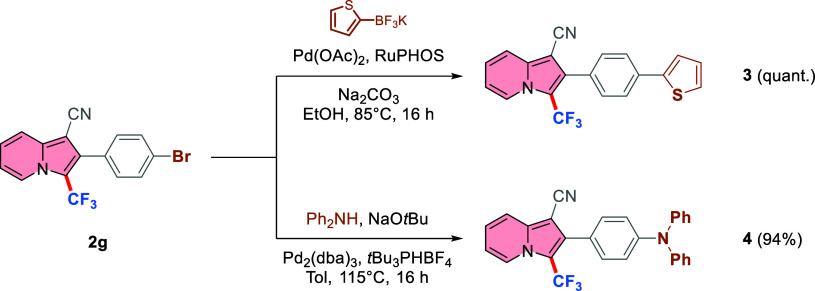
Product Diversification

The synthetic versatility of the resulting indolizine **2g** was also investigated. Both Suzuki^67^ and Buchwald^68^ coupling effectively afforded the desired functionalized indolizines **3** and **4** in excellent yields (Scheme 4). Notably, compounds **3** and **4** are potentially interesting dyes due to their extended π-system, which includes an electron-poor core (indolizine with CF_3_ and CN substituents) and an electron-donating unit like thiophene or diphenylamine, respectively. This peculiarity gives rise to push–pull systems that can be further explored for the study of novel dyes. Indeed, indolizines have been a privileged scaffold for the preparation of optic materials and various chromophores.^4,69−73^

**Scheme 5:**
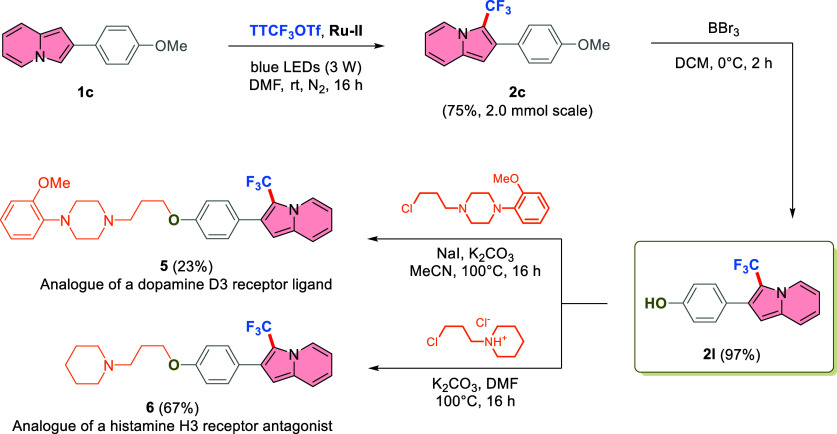
Synthesis of Analogues of Biologically Active Compounds

Considering the relevance of the indolizine scaffold in medicinal chemistry,^1,2^ 3-CF_3_ analogues of biologically active indolizines have been prepared. Starting from 2.0 mmol of indolizine **1c**, it was possible to apply the scale-up protocol to obtain the desired 3-trifluoromethylated indolizine **2c** in a 75% yield (vs 86% yield obtained under the optimized conditions). Simple high-yielding deprotection of the methoxy group with BBr_3_ and further alkylation of the free OH group led to the synthesis of products **5** and **6**, in 23% and 67% yields, respectively, over 2 steps (Scheme 5). Since trifluoromethylated molecules usually show superior chemical, physical, and biological activities in comparison to their nonfluoroalkylated counterpart,^14−16^ we believe that **5** and **6** are interesting candidates for further medicinal applications. Nontrifluoromethylated analogues of **5** and **6** have already been designed and tested as dopamine D3 receptor ligands^74^ and as histamine H3 receptor antagonists.^75^

**Scheme 6:**
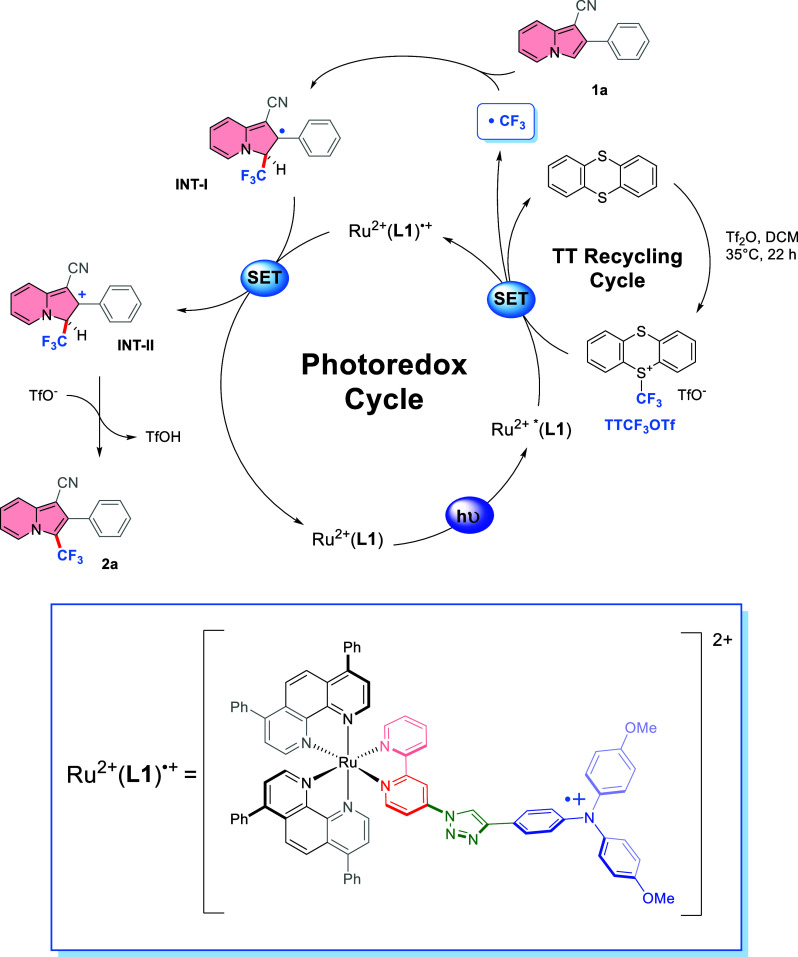
Proposed Reaction Mechanism

Based on the experiments and literature reports,^31,63,76^ a plausible reaction mechanism for the photocatalytic trifluoromethylation can be proposed (Scheme 6). Excitation of the photoredox catalyst from Ru^2+^(**L1**) to Ru^2+^ *(**L1**) with blue light can generate the CF_3_ radical from TTCF_3_OTf (*E*_p_ = −0.44 V vs SCE) because of a high reducing power of **Ru–II** (*E*^0^ [Ru^2+^(**L1**)^•+^/Ru^2+^ *(**L1**)] = −1.30 V vs SCE).^77,78^ The CF_3_ radical can be captured by indolizine **1a** to produce **INT-I**. The resulting radical species can be oxidized by Ru^2+^(**L1**)^•+^ to a carbocation **INT-II** since the reduction potential of the oxidized catalyst is sufficiently high (*E*^0^ [Ru^2+^(**L1**)^•+^/Ru^2+^(**L1**)] = +0.84 V vs SCE). This oxidation regenerates the catalyst to close the photoredox cycle. In addition, **INT-II** can be readily deprotonated by a base to restore the aromaticity and leading to the desired product **2a**. Ultimately, TT formed as a byproduct of the reaction can be easily recovered and converted back to the active TTCF_3_OTf via a one-step protocol.^63^

**Figure 2:**
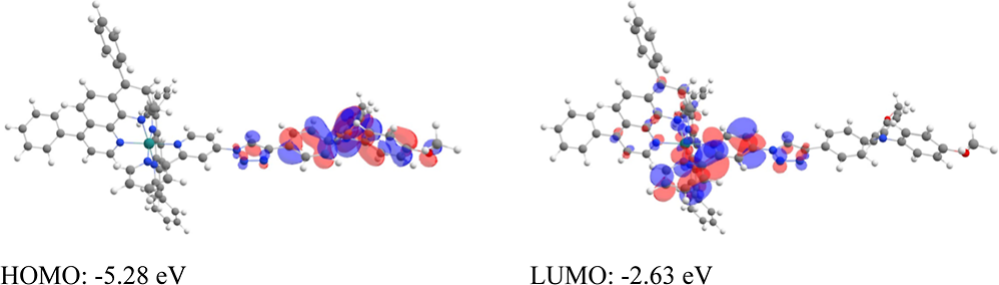
HOMO–LUMO of **Ru–II**^(2+)^ in DMF at the PBE0-def2-TZVP level of theory.

In order to further evaluate the best-performing complex, **Ru–II**, DFT studies were carried out. From the optimized structure, the triplet state and the oxidized form of **Ru–II** were further optimized with the same level (for details see the Supporting Information, page S33). The analytical frequency calculations resulted for all structures only in positive values. The HOMO–LUMO levels and orbitals for **Ru–II** are depicted in Figure 2. It is evident that the HOMO with an energy of −5.28 eV (+0.78 eV vs SHE) is located in the triphenylamine unit and the LUMO with an energy of −2.63 eV (−1.87 eV vs SHE) in the bipyridine unit of the push–pull ligand dMeOTPA-Tz-bpy. A population analysis of the Löwdin atomic charges of the **Ru–II** complex (see the Supporting Information, page S34) shows that the Ru atom has a slight positive charge of 0.13. While most nitrogen atoms are slightly positively charged, the nitrogen of the triphenylamine unit has the highest charge of 0.29. The two unsubstituted nitrogen atoms of the triazole unit show a low positive charge for the 39N and a slightly negative charge of −0.018 for the 38N atom (see the Supporting Information, page S35).

**Figure 3:**
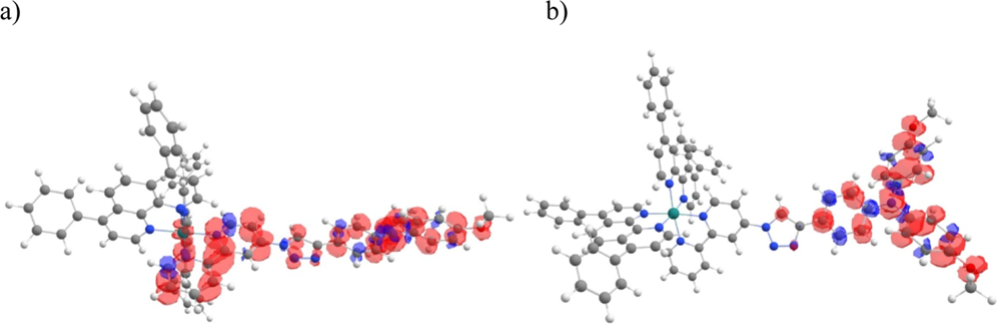
Spin densities of the (a) Ru–II^(2+)^ triplet and (b) Ru–II^(3+)^ in DMF at the PBE0-def2-TZVP level of theory.

The calculated HOMO–LUMO levels and orbitals for the triplet state of **Ru–II** are shown in the Supporting Information, page 39. The highest SOMO has a calculated energy of −3.63 eV (−0.87 eV vs SHE), while the second SOMO has an energy of −5.84 eV. The population analysis of the Löwdin atomic charges and spins of the **Ru–II** complex (see the Supporting Information, page S38) shows that the Ru atom still has a slight positive charge of 0.13. The positive charge of the nitrogen of the triphenylamine unit has increased to 0.38. The two unsubstituted nitrogen atoms of the triazole unit show a low positive charge for the 39N and a slightly negative charge of −0.028 for the 38N atom (see the Supporting Information, page S38). The spin population on the ruthenium atom is with 0.03 rather low. Combined with the high-spin populations at the triphenylamine nitrogen atom (0.242) and the two bipyridine nitrogen atoms (0.146 and 0.107), it is possible to conclude that the unpaired electrons density is rather delocalized over the dMeOTPA-Tz-bpy ligand and not localized on the ruthenium atom itself. The calculated triplet state of **Ru–II** shows a spin density located along the dMeOTPA-Tz-bpy ligand with the highest density around the triphenylamine unit and the bipyridine unit, as shown in Figure 3a.

The SOMO for the oxidized **Ru–II** complex is shown in Figure S11, page S43 of the Supporting Information and has an energy of −6.11 eV (+1.61 vs SHE), while the LUMO has a calculated energy of −2.70 eV (−1.8 eV vs SHE). The SOMO is mainly located around the triphenylamine unit of dMeOTPA-Tz-bpy. The population analysis of the Löwdin atomic charges and spins of the oxidized **Ru–II** complex (see the Supporting Information, page S41) shows that the Ru atom still has nearly the same positive charge of 0.132 compared to the nonoxidized complex. The positive charge of the nitrogen of the triphenylamine unit has increased to 0.38. The two unsubstituted nitrogen atoms of the triazole unit show a small increased positive charge for the 39N and a slightly decreased negative charge of −0.008 for the 38N atom (see the Supporting Information, page S42). The spin population on the ruthenium atom is 0.001, even lower compared to the nonoxidized triplet state. The highest spin population is at the triphenylamine nitrogen atom (0.242); however, populations of the two bipyridine nitrogen atoms have decreased nearly to zero. Hence, it is possible to conclude that the unpaired electron is rather localized in the triphenylamine unit of the dMeOTPA-Tz-bpy ligand. In addition, the spin density for **Ru–II**^(3+)^ was calculated. As shown in Figure 3, the spin density is mainly located on the triphenylamine unit, while the Ru-center remains Ru^(2+)^ with no unpaired electrons.

For comparison, all calculations were repeated for complex **Ru–III** (see the Supporting Information, page S44). Next to the expected differences in the HOMO/LUMO and SOMO/LUMO levels the calculated spin-densities of the triplet (Supporting Information, Figure S13, page S48) and the oxidized complex (Supporting Information, Figure S15, page S52) stand out. In the first case, the Ru-atom is clearly involved in the triplet state, and in the latter, the unpaired electron is completely located at the metal center without reaching into the ligands of the complex.

**Figure 4:**
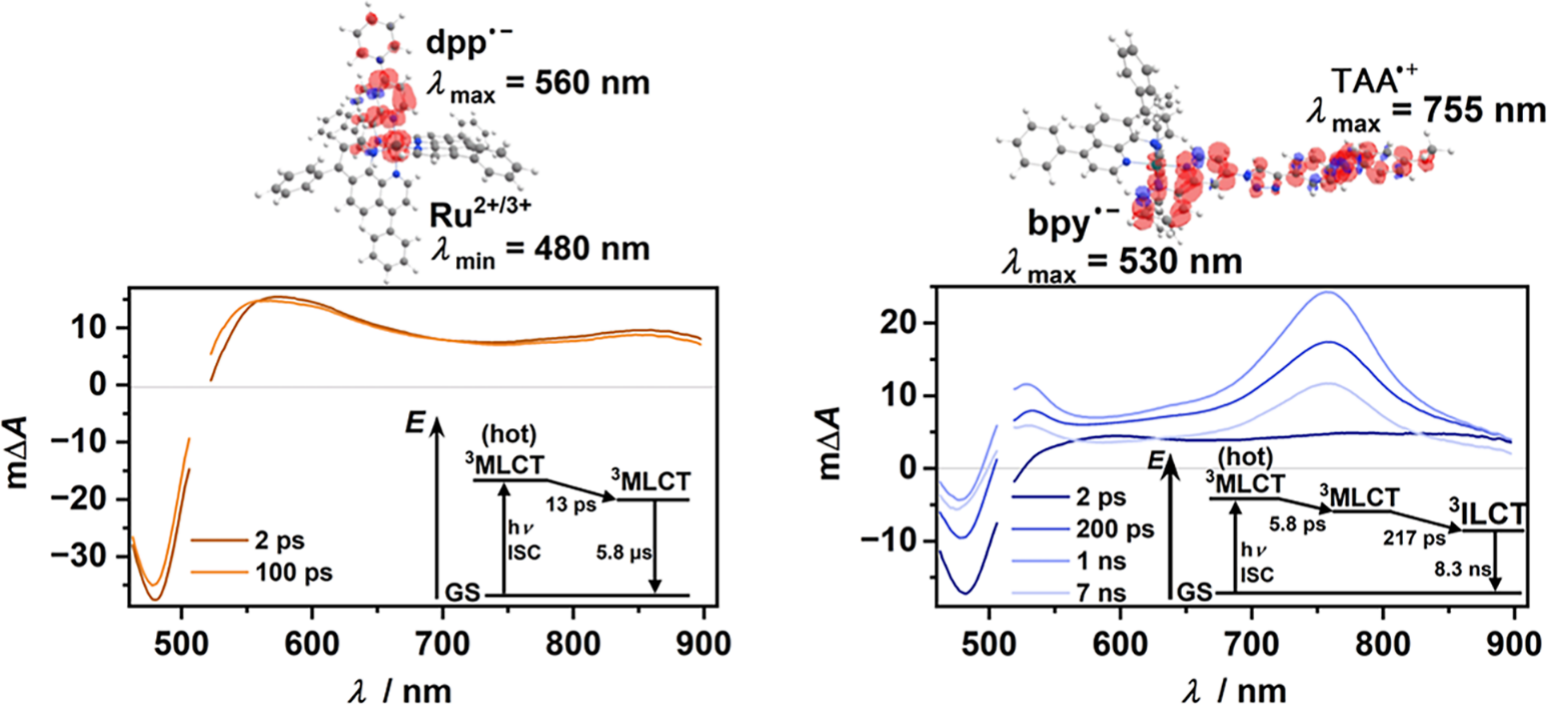
Spectroscopic investigations of **Ru–III** (left) and **Ru–II** (right) on the ps-to-ns time scale (up to 7 ns). Transient absorption spectra of the complexes (100 μM) in Ar-saturated DMF after laser excitation (515 nm, delay times between 2 ps and 7 ns), along with the depiction of the spin densities of the lowest triplets as well as associated energy diagrams.

To obtain experimental evidence for the different photochemical properties of the Ru-based photocatalysts that might explain the divergent performance observed in the test reaction of this study (compare Tables 1 and 2), time-resolved optical spectroscopy was carried out. First, we compared [Ru(dpp)_3_](PF_6_)_2_ (**Ru–III**) and [Ru(dpp)_2_(dMeOTPA-Tz-bpy)](PF_6_)_2_ (**Ru–II**) using a femtosecond transient absorption spectrometer (fs-TAS) with 515 nm pulses of 290 fs duration for selective excitation of the respective metal-to-ligand charge transfer (MLCT) absorption band (see the Supporting Information, Figures S22 and S23). The transient absorption (TA) spectra for the homoleptic complex **Ru–III** show the spectroscopic signatures that are expected for a conventional MLCT triplet state (Figure 4, left),^34^ namely, a pronounced ground-state (GS) bleach at ∼480 nm due to the oxidation of Ru^II^ to Ru^III^ along with positive TA signals in the visible region peaking at ∼560 nm as a result of the radical anion localized on the diimine ligand(s). Besides vibrational cooling being mainly visible in the region around 550 nm, these spectroscopic signals are essentially unchanged on a ps-to-ns time scale. The complete decay of the ^3^MLCT state could be observed with a laser flash photolysis (LFP) system, and a triplet lifetime of 5.8 μs was determined, which is close to the literature value in acetonitrile (5.1 μs).^79^ Almost identical ^3^MLCT signatures were recorded for **Ru–II** right after the laser pulse (Figure 4, right). However, that locally excited ^3^MLCT state is converted to another CT state with a time constant as fast as 217 ps. The resulting TA spectrum of the long-lived lowest excited state of **Ru–II** clearly contains the characteristic signatures of a triarylamine (TAA) radical cation (∼755 nm) and a bipyridine radical anion (∼530 nm)^80−82^ localized on the dMeOTPA-Tz-bpy ligand. Hence, that state is best described as an intraligand charge transfer triplet (^3^ILCT). These spectral assignments are consistent with the DFT-calculated spin densities for the lowest triplet states, which are displayed above the respective TA spectra in Figure 4.

**Figure 5:**
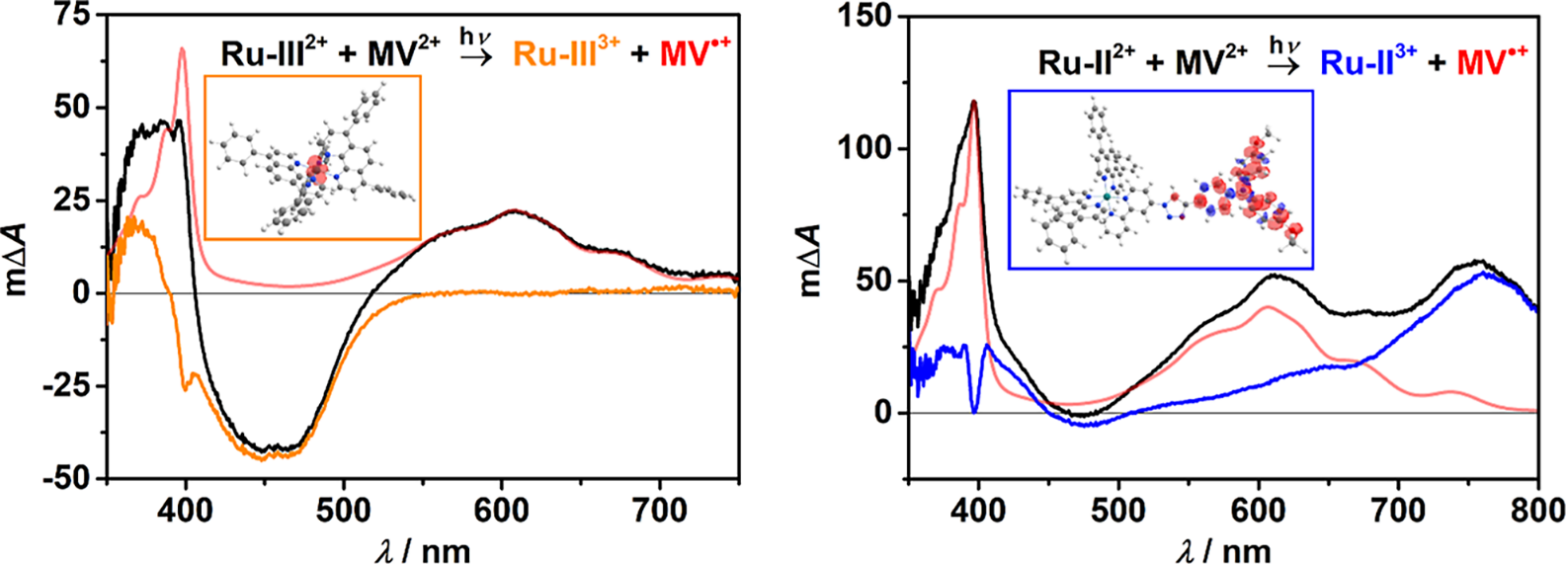
(Left) Transient absorption spectrum of **Ru–III** (38 μM) and MV^2+^ (50 mM) in Ar-saturated DMF after laser excitation (λ_exc_ = 532 nm, delay = 1 μs, black), absorption spectrum of MV^•+^ recorded upon reductive spectroelectrochemistry of MV^2+^ under conditions as in ref (89) (red) and the difference spectrum of both spectra (orange) along with the depiction of the spin density of **Ru–III**^**3+**^. (right) Corresponding results for **Ru–II** (30 μM) with MV^2+^ (50 mM), MV^•+^ reference spectrum (red), and difference spectrum (blue).

TA spectra and the dynamics are similar for **Ru–I**, but a longer lifetime of the ^3^ILCT state was measured (17.8 ns, see the Supporting Information, page S57, for details). Stern–Volmer quenching studies with TTCF_3_OTf as the quencher were unsuccessful, and we actually observed a lifetime elongation in the case of **Ru–I** when adding TTCF_3_OTf. We speculate that this observation is a result of rather inefficient overall and irreversible quenching (consistent with the relatively long irradiation time that implies low overall quantum yields) in combination with counterion effects when PF_6_^–^ is replaced by TfO^–^.^65,83,84^ To study oxidative quenching with the superior photocatalyst **Ru–II**, we alternatively selected the well-characterized electron acceptor methyl viologen MV^2+^ (as its PF_6_^–^ salt, see the Supporting Information, page S56) because its radical cation has intense and characteristic absorption bands with maxima at ∼395 and ∼605 nm.^85,86^ With the flash-quench technique (532 nm excitation with pulses of ∼5 ns duration)^87^ at relatively high MV^2+^ concentrations, we observed postquenching spectra with pronounced MV^•+^ absorption bands for both the reference compound **Ru–III** and **Ru–II** (Figure 5). Additional Stern–Volmer experiments (Figures S18 and S19) revealed that (i) oxidative catalyst quenching is the primary photochemical process and (ii) the rate constant for ^**3**^**Ru–II** quenching is higher than that for ^**3**^**Ru–III** by about 1 order of magnitude. Identical laser pulse energies and absorbances of the Ru-complexes as well as quencher concentrations were used for the comparative flash-quench experiments. The results of the spectral separation with an independently recorded MV^•+^ spectrum (in MeCN)^88,89^ allow us to draw two conclusions. First, despite the much shorter lifetime of **Ru–II** compared to **Ru–III**, an even higher concentration of the photoproduct MV^•+^ is obtained when using the dyad-like **Ru–II** complex, implying a higher inherent cage escape yield for **Ru–II**.^90,91^ Second, the nature of the oxidized Ru complex is completely different in both cases, which can be seen from the difference spectra in Figure 5, colored orange and blue. The oxidation of **Ru–III**^**2+**^ to **Ru–III**^**3+**^ is completely metal-centered as typically observed for homoleptic Ru^II^ complexes with diimine ligands,^92,93^ whereas **Ru–II**^3+^ contains a TAA radical cation indicated by the characteristic absorption band in the red spectral region. These results and assignments are again substantiated by the DFT-calculated spin densities of the oxidized complexes. Finally, quantitative laser experiments (Figure S20) revealed that cage escape for **Ru–II** is higher by a factor of 3 compared to **Ru–III** for the methyl viologen photoreduction as a test reaction, further highlighting the advantages of the novel photocatalyst. In addition, the postquenching product **Ru–II**^**3**+^, which is formed after oxidative quenching of ^**3**^**Ru–II** by TTCF_3_OTf (detected at very high quencher concentrations), could be assigned via the characteristic transient absorption spectrum (Figure S25). For that, the difference spectrum from the investigations with MV^2+^ (Figure 5 (right)) was used, which is in line with the first light-driven step in the proposed mechanism in Scheme 6.

The results of this section unambiguously demonstrate that both the lowest triplet state and the oxidized species of the dyad-like complex **Ru–II** have a completely different character compared to the respective states of the homoleptic reference complex. Given that the reactivity of both states is crucial for the reaction mechanism summarized in Scheme 6, these pronounced differences are most likely the main reason for the superior performance of the novel photocatalyst **Ru–II**.

## Conclusions

In summary, a mild, simple, and chemoselective protocol for the synthesis of 3-(trifluoromethyl)indolizine has been developed. The desired products were prepared in good to excellent yields and can be easily prepared on a gram scale. By tuning the redox properties of the developed photocatalyst **Ru–II,** it was possible to achieve competitive yields and further apply the optimized conditions to a broad variety of substrates. This method tolerates many functional groups and therefore can be used for late-stage functionalization. Moreover, TT, the byproduct of the reaction, can be recycled in an overall atom-economic transformation. Furthermore, the enhanced Ru-catalyst **Ru–II** has been prepared in good yields and was investigated using detailed theoretical and spectroscopic techniques. This new dyad-like complex will be applied in further reactions in the future. Our results revealed that the triarylamine moiety in the catalyst significantly contributes to the reactivity of the catalyst triplet and its oxidized species.

## Data Availability

The data underlying this study are available in the published article, in its Supporting Information, and openly available in the JGU library at https://doi.org/10.25358/openscience-12102.

## Supporting Information Available

Full experimental description, spectral data of the compounds, and additional DFT and time-resolved optical spectroscopy data (PDF)
