# Supplementary material for: Bichromophoric Ruthenium Complexes for Photocatalyzed Late-Stage Synthesis of Trifluoromethylated Indolizines
Source: J Org Chem. 2025 May 5;90(19):6491–503. doi: 10.1021/acs.joc.5c00319 (PMC12090221; doi:10.1021/acs.joc.5c00319)
Supplement: Supplementary file 1 — jo5c00319_si_001.pdf [file jo5c00319_si_001.pdf]

Supplementary Information

Bichromophoric Ruthenium Complexes for Photocatalyzed Late-Stage Synthesis of Trifluoromethylated Indolizines

Kevin Klaus Stefanoni[a], Matthias Schmitz,[b] Johanna Treuheit,[b] Christoph Kerzig[b]* and René Wilhelm[a]*

[a] Institute of Organic Chemistry, Leibnizstr. 6, Clausthal University of Technology
phone +495323722040, fax number: +495323722834, e-mail: rene.wilhelm@tu-clausthal.de
[b] Department of Chemistry, Duesbergweg 10-14, Johannes Gutenberg University Mainz
e-mail: ckerzig@uni-mainz.de

Table of contents

1. General experimental information S3

2. Photocatalytic reaction conditions S3

3. Starting materials preparation and costs esteem S5

4. Ligand preparation S7

5. General procedure for Ru(II) heteroleptic complex preparation S9

6. Trifluoromethylation of indolizines S12

6.1. General procedure for screening experiments S12

6.2. General procedure A for trifluoromethylation of indolizines S12

6.3. General procedure B for trifluoromethylation of indolizines (2p, 2q) S13

6.4. General procedure C for trifluoromethylation of indolizines (2h) S13

6.5. Gram scale reaction and product diversification S25

6.6. Synthesis of analogues of biologically active compounds S27

7. Absorption, emission and electrochemical porperties S29

8. DFT calculations S33

9. fs-TAS and LFP quenching experiments S56

10. NMR spectra S65

1. General experimental information

All reactions were carried out under an atmosphere of nitrogen in oven-dried glassware, unless otherwise stated. All reactions that require heating were conducted using a steel heat-on block. All chemicals were purchased and used without further purification unless otherwise mentioned. Anhydrous solvents were dried according to standard procedures before usage and stored in a glovebox. All NMR-Spectra have been measured using either BRUKER Digital AVANCE 400 MHz FTNMR or a BRUKER Digital AVANCE III 600 MHz FT-NMR. The chemical shifts are reported in ppm, the coupling constants in Hz. All mass spectra have been measured using a Hewlett-Packard Agilent LC/MSD-System Series HP 1100 with API-ES and the detector is TOF. All UV-vis spectra have been measured using a JASCO V-650 spectrophotometer or a JASCO V-760 spectrophotometer. The reactions were traced by thin layer chromatography with silica gel 60 (F254, MERCK KGAA). For the detection of substances, quenching was used at either 254 nm or 366 nm with a UV lamp. The preparative column chromatography was conducted through silica gel 60 (230–400 mesh).

All cyclic voltammetry experiments were performed in 0.1 M $[Bu_4N][PF_6]$ MeCN solution with an Autolab PGSTAT204 potentiostat/galvanostat (Metrohm). A cell with a three-electrode configuration was used. The glassy carbon working electrode (d=2 mm) was polished before each measurement with a 0.03 µm Al_2O_3 slurry and then rinsed thoroughly with deionized water and MeCN. A platinum sheet was used as counter electrode, while the reference electrode was Ag/AgCl (3.0 M KCl). The measurements were performed at a temperature of 22 °C under a nitrogen atmosphere after bubbling the solutions with the same gas for 10 min. A pre-bubbler is also included in the experimental set-up, to prevent excessive evaporation of the solvent. The IUPAC plotting convention was used to plot voltammograms. The initial potential was set to 0.0 V, and the scan proceeded in the oxidation direction up to the potential shown in the plot.

For the spectroscopic investigations, all solvents and reagents were obtained from commercial suppliers and used without further purification unless otherwise stated (*N*,*N*-dimethylformamide, 99.9%, Th.Geyer; *N*,*N*′-dimethyl-4,4′-bipyridinium dichloride (methyl viologen), 98%, Sigma-Aldrich; ammonium hexafluorophosphate, 98%, abcr; tris(2,2'-bipyridine)dichlororuthenium(II) hexahydrate, Sigma-Aldrich). The hexafluorophosphate salts of tris(2,2'-bipyridine) ruthenium(II) (**$[Ru(bpy)_3]^{2+}$**) and methyl viologen (MV^{2+}) have been prepared via precipitation from water upon addition of ammonium hexafluorophosphate, thorough washing with water and drying. Argon from Nippon Gases (5.0) was bubbled through the solutions to remove dissolved oxygen prior to experiments related to optical spectroscopy.

Transient absorption spectra and time-resolved absorption and emission traces on a nanosecond to microsecond time scale were recorded with an LP980KS setup from Edinburgh Instruments equipped with an Nd:YAG laser from Litron (Nano LG 300-10). The frequency-doubled (532 nm) output served

as the excitation source. The laser pulse duration was about 5 ns and the pulse frequency was 10 Hz. The excitation energy was adjusted with a mechanical attenuator to an intensity of about 40 mJ per laser pulse, which was measured with a pyroelectric detector from gentec-eo (QE25LPS-MB-QED-D0). The beam diameter in front of the cuvette window was approximately 1.2 cm. Transient absorption spectra were detected using an iCCD camera from Andor. The integration time of the spectral measurements was 100 ns. Single-wavelength kinetics were recorded using a Hamamatsu photomultiplier tube (R928). Spectroscopic experiments were performed using a temperature-controlled cell holder. LFP measurements were performed at 20 °C using 1 cm quartz glass cuvettes.

Transient absorption spectra on a femtosecond to nanosecond time scale (fs-TAS) were recorded with the HARPIA-LIGHT spectrometer from Light Conversion. The primary light source was a femtosecond pulsed laser operating with an output wavelength of 1030 nm, an output power of 4 W, a repetition rate of 60 kHz and a pulse length of ≤ 290 fs. The fundamental wavelength of the laser was used to generate the broadband probe. The second harmonic of the laser (515 nm) was used as the pump beam. Preprocessing of the data, including chirp and baseline correction, was performed using the CarpetView 2.0 software from Light Conversion. A global fit analysis for determining the time constants of intramolecular photoinduced processes was carried out using the python package KiMoPack.[1] Steady-state emission measurements were performed with a Perkin Elmer FL-6500 spectrometer.

2. Photocatalytic reaction conditions

Reactions were performed in open round-bottom-flasks illuminated from below with Avonec 3 W High Power LEDs (https://www.avonec.de/3w-high-power-led/3w-high-power-led-10000k-20000k-kaltweiss-46-51-52-53.html) affixed to a cooling block and the setup was prevented from heating through a water-cooled metal case, as shown in **Figure S1** as an example. No filters were used during the irradiation process. The emission spectrum of the used blue LED is shown in **Figure S2** below.

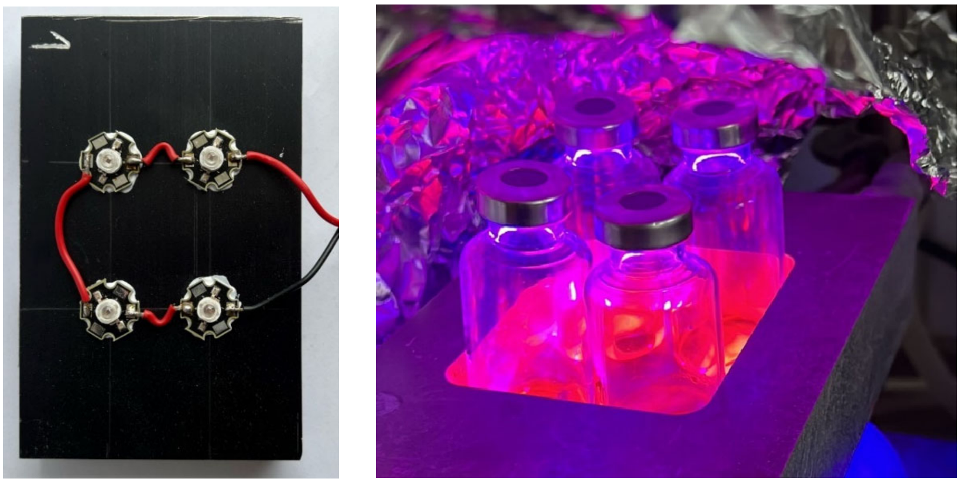

Figure S1. Custom LED photoreactor for reaction optimization and scale-up processes.

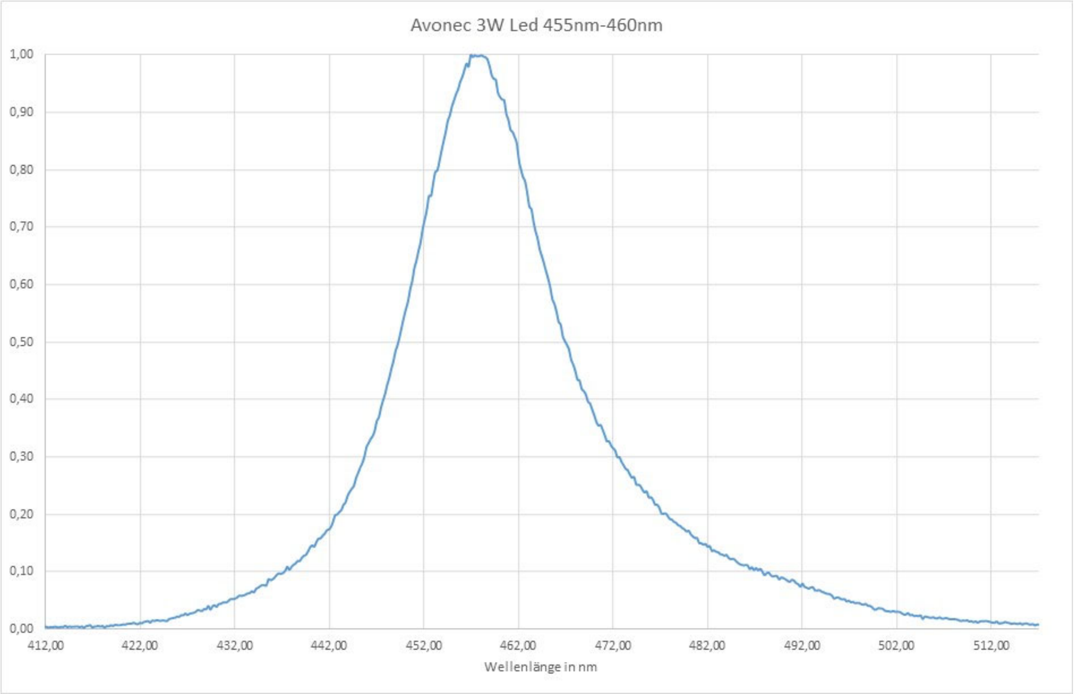

Figure S2. Emission spectrum of the used 455 nm LED lamp, as provided by the manufacturer.

3. Starting materials preparation and costs esteem

Indolizines **1a, 1c, 1e, 1f, 1g, 1j, 1k, 1m, 1n, 1o, 1r, 1u, 1w** were prepared by boiling an equimolar MeCN solution of the corresponding substituted picoline and α-bromoacetophenone for 16 h, according to literature method.[2] Indolizines **1b, 1d, 1h, 1l, 1p, 1q, 1t, 1v** were prepared according to the literature.[3,4] Indolizines **1i, 1s** were prepared according to the literature.[5] 4-azido-[2,2'-bipyridine] 1-oxide was prepared according to a published method.[6] Ru(II) precomplexes were prepared by heating an *o*DCB solution of ligand and $[Ru(COD)Cl_2]_n$ at 140°C for 3 h, according to literature.[7] 1-(3-chloropropyl)-4-(2-methoxyphenyl)piperazine was prepared according to a published method.[8] Umemoto II[9] was prepared according to a literature method.

All unitary prices for each chemical used in the synthesis of **Ru-II** complex were calculated by dividing the listed prices of the largest available unit on Fluorochem (https://dougdiscovery.com) by the amount of each compound required. The unitary price of standard $Ir(ppy)_3$ photocatalyst is 238 €/g, (156647.65 €/mol) while our designed **Ru-II** proved to be ≈ 10 times cheaper based on €/mol. However, the costs of the solvent, silica gel for column chromatography, and utilities are not included in this esteem since their cost can be considered negligible. Also working hours are not included and hence, this cost comparison is for an academic environment.

Compound	Unitary price	Amount used	Cost
bis(4-methoxyphenyl)amine	0.7 €/g	0.996 g	0.69 €
((4-bromophenyl)ethynyl)trimethylsilane	2.4 €/g	1.000 g	2.40 €
$Pd_2(dba)_3 \cdot CHCl_3$	17.3 €/g	0.109 g	1.89 €
RuPhos	0.6 €/g	0.111 g	0.06 €
NaO*t*Bu	0.2 €/g	0.418 g	0.08 €
TBAF 1 M in THF	0.59 €/mL	2.70 mL	1.59 €
	**Total for 0.872 g of A1**		6.71 €
	**Unitary cost of A1**		7.7 €/g

**A1**	7.7 €/g	0.243 g	1.86 €
$Cu(PPh_3)_3Br$	1.42 €/g	0.069 g	0.10 €
4-azido-[2,2'-bipyridine]-1-oxide	1.0 €/g	0.189 g	0.19 €
PCl_3	0.3 €/mL	0.17 mL	0.05 €
	**Total for 0.349 g of L1**		2.20 €
	**Unitary cost of L1**		6.3 €/g

**L1**	6.3 €/g	0.050 g	0.31 €
$[Ru(COD)Cl_2]_n$	14.0 €/g	0.024 g	0.34 €
$AgNO_3$	3.88 €/g	0.029 g	0.11 €
$NaPF_6$	0.57 €/g	0.036 g	0.04 €
4,7-diphenyl-1,10-phenanthroline	3.4 €/g	0.057 g	0.20 €
	**Total for 0.104 g of Ru-II**		1.01 €
	**Unitary cost of Ru-II**		9.7 €/g
	**Unitary cost of Ru-II (mol)**		15349.4 €/mol

4. Ligand preparation

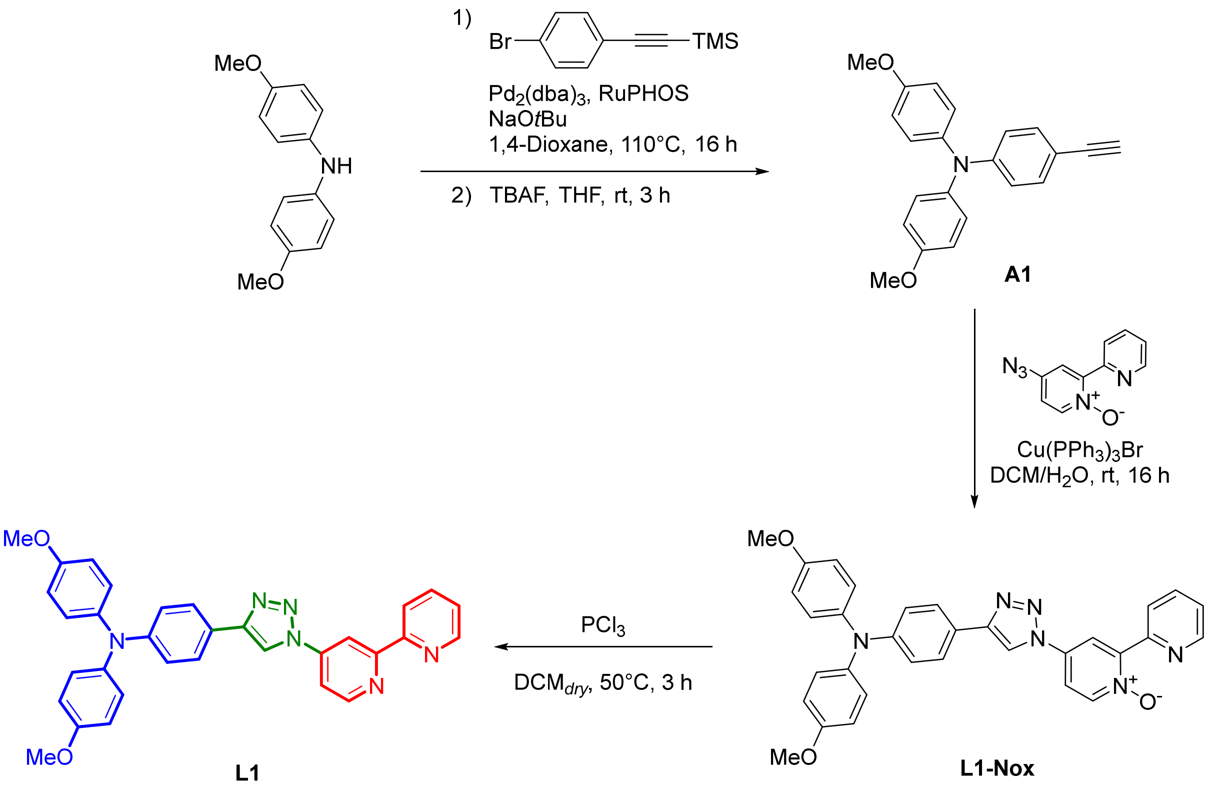

4-Ethynyl-*N*,*N*-bis(4-methoxyphenyl)aniline (A1)

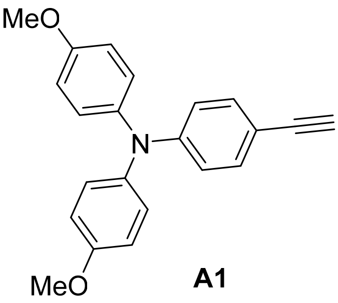

A1

Under inert conditions, ((4-bromophenyl)ethynyl)trimethylsilane (1.000 g, 3.95 mmol, 1.0 eq.), bis(4-methoxyphenyl)amine (0.996 g, 4.34 mmol, 1.1 eq.), $Pd_2(dba)_3$ (0.109 g, 0.12 mmol, 3 mol%), RuPhos (0.111 g, 0.24 mmol, 6 mol%) and NaO*t*Bu (0.418 g, 4.34 mmol, 1.1 eq.) in 1,4-dioxane (39 mL, 0.1 M) were heated to 110°C for 16 h. The reaction mixture was cooled to rt, filtered over a thin pad of Celite (EtOAc used as solvent) and the filtrate is concentrated in vacuo. The residue was purified by flash column chromatography on silica gel (PE:DCM, 1:1) to afford the intermediate product as a yellow solid: 1.049 g, 2.65 mmol, 68%.

The intermediate was dissolved in THF (52 mL, 0.05 M) and a 1 M solution of TBAF was added dropwise on an ice bath. The reaction mixture was stirred for 2 h at rt and the solvent was later evaporated under reduced pressure. The residue is partitioned between H_2O and EtOAc, the organic

phase is washed with H_2O/brine, dried over Na_2SO_4 and the solvent was removed under reduced pressure. The alkyne **A1** was obtained without further purification as a brown oil, which after 2 weeks solidified: 0.872 g, 2.65 mmol, quantitative. The spectral data sets were in agreement with the literature.[10] **^{1}H NMR** (400 MHz, $CDCl_3$) δ 7.32 – 7.18 (m, 2H), 7.13 – 7.01 (m, 4H), 6.96 – 6.76 (m, 6H), 3.80 (s, 6H), 2.99 (s, 1H).

4-(4-(4-(Bis(4-methoxyphenyl)amino)phenyl)-1*H*-1,2,3-triazol-1-yl)-[2,2'-bipyridine] 1-oxide (L1-Nox)

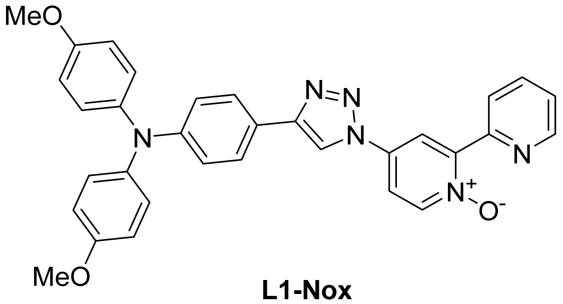

L1-Nox

To a degassed solution of DCM/H_2O (15 mL, 1:1, 0.05 M), 4-azido-[2,2'-bipyridine]-1-oxide (0.189 g, 0.89 mmol, 1.2 eq.), **A1** (0.243 g, 0.74 mmol, 1.0 eq.) and $Cu(PPh_3)_3Br$ (0.069 g, 0.07 mmol, 10 mol%) were added sequentially under inert atmosphere. The reaction mixture was stirred for 16 h at rt and later diluted with DCM. The organic phase was washed with H_2O/brine, dried over Na_2SO_4 and the solvent was removed under reduced pressure. The crude was purified by flash column chromatography on silica gel (DCM:MeOH, 97:3) to afford compound **L1-Nox** as a yellow solid: 0.361 g, 0.66 mmol, 90%. **^{1}H NMR** (400 MHz, CD_2Cl_2) δ 9.06 (dt, J = 8.0, 1.0 Hz, 1H), 8.76 (ddd, J = 4.8, 1.8, 0.9 Hz, 1H), 8.67 (d, J = 3.2 Hz, 1H), 8.38 (d, J = 7.2 Hz, 1H), 8.27 (s, 1H), 7.92 – 7.82 (m, 2H), 7.74 – 7.64 (m, 2H), 7.43 (ddd, J = 7.5, 4.7, 1.2 Hz, 1H), 7.16 – 7.05 (m, 4H), 6.99 – 6.93 (m, 2H), 6.92 – 6.83 (m, 4H), 3.80 (s, 6H). **^{13}C{^{1}H} NMR** (125 MHz, CD_2Cl_2) δ 157.7, 150.7, 150.4, 150.0, 149.2, 143.4, 141.7, 137.9, 134.4, 128.4, 127.8, 126.6, 126.3, 122.4, 121.1, 118.7, 117.4, 117.2, 116.1, 56.8. **IR** (neat, cm^{-1}): 3123, 3053, 2836, 1616, 1571, 1504, 1479, 1322, 1288, 1238, 1180, 1029, 826, 787, 768, 739, 704, 672, 602, 574, 527. **ESI-HRMS**: m/z calcd. for $C_{32}H_{26}N_6O_3Na$ $[M+Na]^+$ 565.1964, found 565.1959.

4-(4-(4-(Bis(4-methoxyphenyl)amino)phenyl)-1*H*-1,2,3-triazol-1-yl)-[2,2'-bipyridine] (L1)

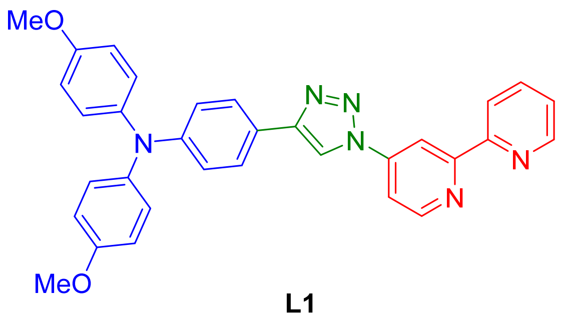

L1

To a solution of **L1-Nox** (0.361 g, 0.66 mmol, 1.0 eq.) in DCM (13 mL, 0.05 M), PCl_3 (0.274 g, 0.17 mL, 1.99 mmol, 3.0 eq.) was added at 0°C under inert atmosphere. The reaction mixture was refluxed for 3 h, then diluted with ice-cold H_2O and basified with 38% m/m NaOH solution. The aqueous layer was extracted with DCM, the combined organic phases are washed with H_2O/brine, dried over Na_2SO_4 and the solvent was removed under reduced pressure. Compound **L1** was obtained without further purification as a yellow fluffy solid: 0.349 g, 0.66 mmol, quantitative. If needed, **L1** can be purified by column chromatography on silica (DCM:MeOH, 99:1). **^{1}H NMR** (400 MHz, CD_2Cl_2) δ 8.85 – 8.77 (m, 2H), 8.70 (ddd, J = 4.7, 1.8, 0.9 Hz, 1H), 8.51 (dt, J = 8.0, 1.0 Hz, 1H), 8.40 (s, 1H), 7.94 – 7.84 (m, 2H), 7.76 – 7.67 (m, 2H), 7.39 (ddd, J = 7.5, 4.8, 1.2 Hz, 1H), 7.15 – 7.04 (m, 4H), 7.00 – 6.92 (m, 2H), 6.91 – 6.81 (m, 4H), 3.80 (s, 6H). **^{13}C{^{1}H} NMR** (125 MHz, CD_2Cl_2) δ 158.6, 156.6, 155.1, 151.3, 149.6, 149.5, 149.1, 144.5, 140.7, 137.4, 127.3, 126.8, 124.8, 121.6, 121.4, 120.1, 116.2, 115.0, 113.9, 110.5, 55.7. **IR** (neat, cm^{-1}): 3055, 2932, 2839, 1615, 1584, 1562, 1503, 1237, 1029, 826, 790, 708, 575, 526. **ESI-HRMS**: m/z calcd. for $C_{32}H_{26}N_6O_2Na$ $[M+Na]^+$ 549.2015, found 549.2009.

5. General procedure for Ru(II) heteroleptic complex preparation

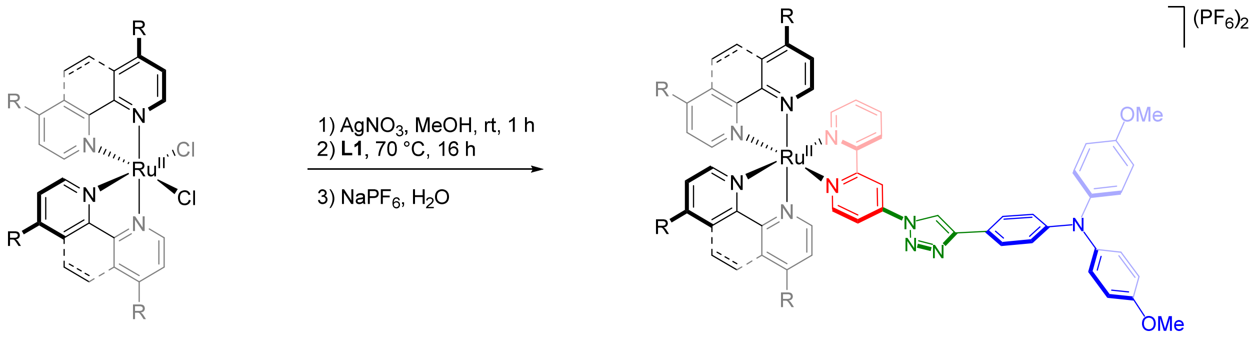

Ru(II) precomplex (86.0 µmol, 1.0 eq.) was mixed with $AgNO_3$ (29.3 mg, 173.0 µmol, 2.0 eq.) in MeOH (6 mL, 0.015 M) and the reaction mixture was stirred for 1 h at rt. The resulting suspension was filtered and then mixed with **L1** (50.0 mg, 95.0 µmol, 1.1 eq.). The mixture was heated at reflux for 16 h and later it was cooled down to rt. The solvent was evaporated under reduced pressure and the resulting solid

was dissolved in the minimum amount of MeOH. The product is precipitated by addition of a $NaPF_6$ solution in distilled H_2O, washed sequentially with distilled H_2O/hexane/Et_2O and dried in vacuo.

[Ru(bpy)$_2$(dMeOTPA-Tz-bpy)](PF$_6$)$_2$ – Ru-I

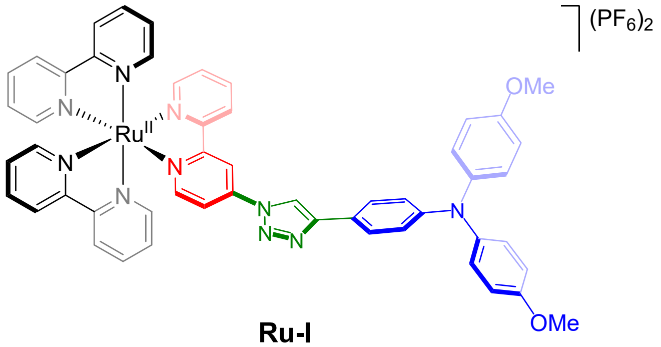

Ru-I

Complex **Ru-I** was obtained following the general procedure, using *cis*-dichlorobis(2,2'-bipyridine)ruthenium dihydrate (44.9 mg, 86.0 µmol, 1.0 eq.), as a deep red solid: 0.104 g, 84.3 µmol, 98%. **^{1}H NMR** (600 MHz, CD_3CN) δ 8.95 (dd, J = 2.1, 1.0 Hz, 1H), 8.81 (s, 1H), 8.71 (dt, J = 8.1, 1.1 Hz, 1H), 8.59 – 8.45 (m, 4H), 8.21 – 8.02 (m, 5H), 7.93 – 7.83 (m, 3H), 7.78 (m, 6H), 7.55 – 7.38 (m, 5H), 7.13 (m, 4H), 6.95 (m, 6H), 3.81 (s, 6H). **^{13}C{^{1}H} NMR** (150 MHz, CD_3CN) δ 159.5, 157.6, 157.5, 157.3, 157.0, 153.9, 152.5, 152.4, 152.4, 152.3, 149.6, 144.4, 140.7, 138.5, 128.8, 128.3, 128.2, 128.2, 127.9, 127.1, 125.5, 124.9, 124.9, 121.3, 119.6, 117.3, 115.5, 114.4, 55.7. **^{19}F{^{1}H} NMR** (377 MHz, CD_3CN) δ -71.46 (d, 12F). **IR** (neat, cm^{-1}): 3063, 2932, 2835, 1615, 1585, 1563, 1504, 1239, 1031, 828, 762, 556. **ESI-HRMS**: m/z calcd. for $C_{52}H_{42}N_8O_2Ru$ [(M-N$_2$)]$^{2+}$ 456.1237, found 456.1228.

[Ru(dpp)$_2$(dMeOTPA-Tz-bpy)](PF$_6$)$_2$ – Ru-II

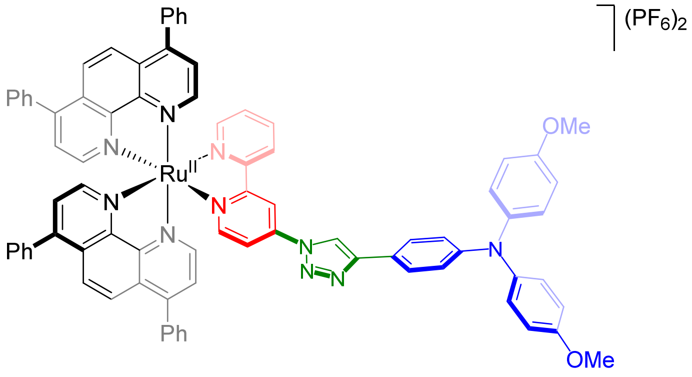

Ru-II

Complex **Ru-II** was obtained following the general procedure, using *cis*-dichlorobis(4,7-diphenyl-1,10-phenanthroline)ruthenium (72.2 mg, 86.0 µmol, 1.0 eq.), as a deep red solid: 0.104 g, 65.6 µmol, 76%. **^{1}H NMR** (600 MHz, CD_3CN) δ 9.02 (d, J = 2.3 Hz, 1H), 8.79 (d, J = 13.3 Hz, 2H), 8.43 (d, J = 5.5 Hz,

1H), 8.33 (d, J = 5.5 Hz, 1H), 8.29 (d, J = 5.5 Hz, 1H), 8.25 – 8.19 (m, 5H), 8.17 (d, J = 8.1 Hz, 2H), 8.13 (dd, J = 5.5, 2.5 Hz, 3H), 8.01 (d, J = 6.3 Hz, 2H), 7.93 (d, J = 5.2 Hz, 1H), 7.83 (dd, J = 6.3, 2.3 Hz, 1H), 7.78 (d, J = 3.6 Hz, 3H), 7.73 (d, J = 8.7 Hz, 3H), 7.65 – 7.61 (m, 18H), 7.46 – 7.41 (m, 2H), 7.09 (d, J = 9.0 Hz, 4H), 6.96 – 6.85 (m, 6H), 3.78 (s, 6H). **^{13}C{^{1}H} NMR** (150 MHz, CD_3CN) δ 140.7, 130.4, 130.4, 130.3, 129.7, 127.9, 127.1, 126.7, 115.5, 55.7. **^{19}F{^{1}H} NMR** (377 MHz, CD_3CN) δ -71.60 (d, 12F). **IR** (neat, cm^{-1}): 3061, 2931, 2909, 2835, 1615, 1502, 1504, 1239, 1031, 827, 702, 556. **ESI-HRMS**: m/z calcd. for $C_{80}H_{58}N_{10}O_2Ru$ $[M]^{2+}$ 646.1893, found 646.1894.

Ru(4,7-diphenyl-1,10-phenanthroline)$_3$(PF_6)$_2$ – Ru-III

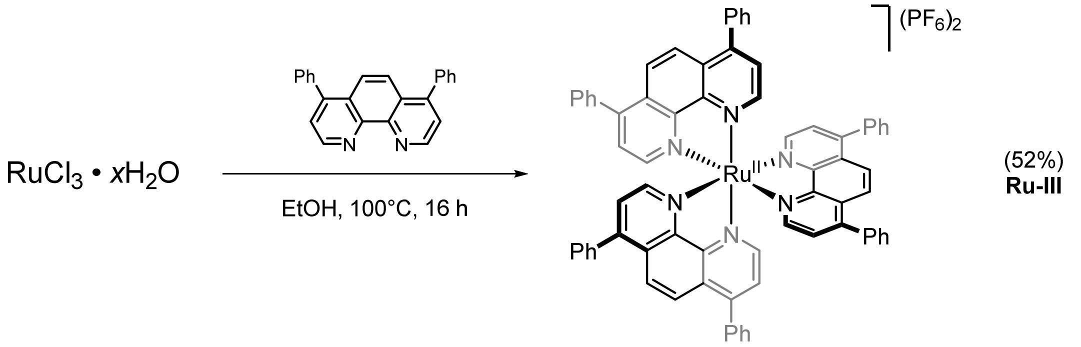

$RuCl_3$ · $x$$H_2O$ (366.1 mg, 14.0 mmol, 1.00 eq.) and 4,7-diphenyl-1,10-phenanthroline (2.792 g, 85.0 mmol, 6.00 eq.) were suspended in EtOH and the resulting mixture was refluxed at 100°C for 16 h. The reaction mixture was concentrated under reduced pressure and a $NaPF_6$ solution in H_2O was added. The resulting precipitate was filtered, washed with cold EtOH/Et_2O/hexane sequentially and dried in vacuo. The crude was purified by flash column chromatography on silica gel (DCM:MeOH, 99:1, R_f = 0.44) to afford compound **Ru-III** as a deep red solid: 1.019 g, 7.28 mmol, 52%. The spectral data sets were in agreement with the literature[11]. **^{1}H NMR** (600 MHz, CD_3CN) δ 8.34 (d, J = 5.6 Hz, 6H), 8.25 (s, 6H), 7.70 (d, J = 5.5 Hz, 6H), 7.68 – 7.57 (m, 30H). **^{13}C{^{1}H} NMR** (150 MHz, CD_3CN) δ 153.4, 150.1, 149.6, 136.8, 130.8, 130.6, 130.5, 130.1, 130.0, 127.1, 127.1. **^{19}F{^{1}H} NMR** (377 MHz, CD_3CN) δ -72.85 (d, 12F). **ESI-HRMS**: m/z calcd. for $C_{72}H_{48}N_6Ru$ $[M]^{2+}$ 549.1492, found 549.1493.

6. Trifluoromethylation of indolizines

6.1. General procedure for screening experiments

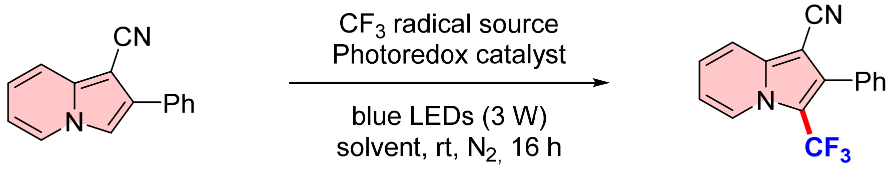

1a **2a**

In a glovebox, to a vial filled with the indolizine **1a** (0.20 mmol, 1.00 eq.), CF_3 radical source (0.25 mmol, 1.25 eq.), photoredox catalyst (5.0 µmol, 2.5 mol%) was added DMF (2.0 mL, 0.1 M). The vial was sealed and the mixture was irradiated with a blue LED (3 W, 455 – 460 nm) for 16 h at rt outside of the glovebox. The reaction mixture was partitioned between H_2O and Et_2O and the organic phase was extracted. The water phase was extracted with Et_2O 3 times and the combined organic phases were washed with H_2O and brine. The organic phase was dried over Na_2SO_4 and the solvent was removed under reduced pressure. To the crude reaction mixture, CH_2Br_2 (34.7 mg, 14.0 µL, 0.2 mmol, 1.00 equiv.) was added as an internal standard. The reaction mixture was diluted with $CDCl_3$, and the yield was determined by ^1H NMR spectroscopy by integration of the peak at δ 4.95 ppm of the internal standard (s, 2H) (**Table 1**).

6.2. General procedure A for trifluoromethylation of indolizines

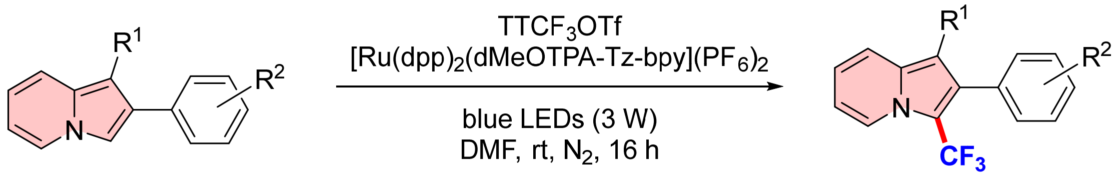

1 **2**

In a glovebox, to a vial filled with the indolizine **1** (0.20 mmol, 1.00 eq.), $TTCF_3OTf$ (108.6 mg, 0.25 mmol, 1.25 eq.), **Ru-II** (7.9 mg, 5.0 µmol, 2.5 mol%) was added DMF (2.0 mL, 0.1 M). The vial was sealed and the mixture was irradiated with a blue LED (3 W, 455 – 460 nm) for 16 h at rt outside of the glovebox. The reaction mixture was partitioned between H_2O and Et_2O and the organic phase was extracted. The water phase was extracted with Et_2O 3 times and the combined organic phases were washed with H_2O and brine. The organic phase was dried over Na_2SO_4 and the solvent was removed under reduced pressure. The residue was purified by flash column chromatography on silica gel to afford the trifluoromethylated products **2**.

6.3. General procedure B for trifluoromethylation of indolizines (2p, 2q)

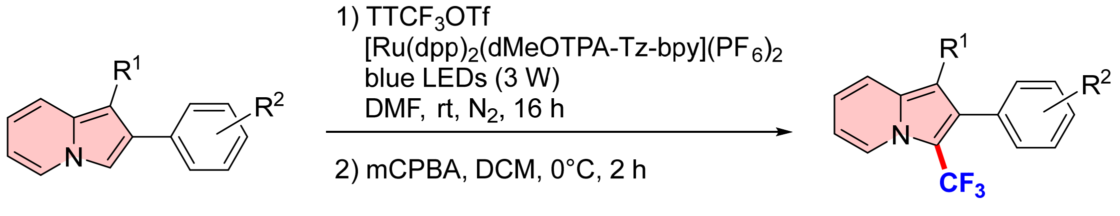

In a glovebox, to a vial filled with the indolizine **1** (0.20 mmol, 1.00 eq.), $TTCF_3OTf$ (108.6 mg, 0.25 mmol, 1.25 eq.), **Ru-II** (7.9 mg, 5.0 µmol, 2.5 mol%) was added DMF (2.0 mL, 0.1 M). The vial was sealed and the mixture was irradiated with a blue LED (3 W, 455 – 460 nm) for 16 h at rt outside of the glovebox. The reaction mixture was partitioned between H_2O and Et_2O and the organic phase was extracted. The water phase was extracted with Et_2O 3 times and the combined organic phases were washed with H_2O and brine. The organic phase was dried over Na_2SO_4 and the solvent was removed under reduced pressure. The crude was dissolved in DCM (0.5 M) and mCPBA 70% (74.0 mg, 0.3 mmol, 1.5 eq.) was added in one portion at 0°C. The reaction mixture was stirred for 2 h at 0°C. The reaction mixture was extracted with aqueous $NaHCO_3$ and DCM. Then, the organic layer was dried with Na_2SO_4 and the solvent was removed under reduced pressure. The residue was purified by flash column chromatography on silica gel to afford the trifluoromethylated products **2**.

6.4. General procedure C for trifluoromethylation of indolizines (2h)

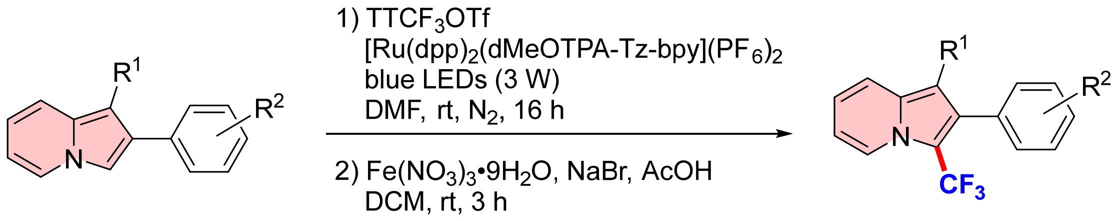

In a glovebox, to a vial filled with the indolizine **1** (0.20 mmol, 1.00 eq.), $TTCF_3OTf$ (108.6 mg, 0.25 mmol, 1.25 eq.), **Ru-II** (7.9 mg, 5.0 µmol, 2.5 mol%) was added DMF (2.0 mL, 0.1 M). The vial was sealed and the mixture was irradiated with a blue LED (3 W, 455 – 460 nm) for 16 h at rt outside of the glovebox. The reaction mixture was partitioned between H_2O and Et_2O and the organic phase was extracted. The water phase was extracted with Et_2O 3 times and the combined organic phases were washed with H_2O and brine. The organic phase was dried over Na_2SO_4 and the solvent was removed under reduced pressure. The crude was dissolved in DCM/AcOH (0.3 M, 70:1) and $Fe(NO_3)_3 \cdot 9H_2O$ (121.0 mg, 0.30 mmol, 1.5 eq.) and NaBr (1.2 mg, 12 µmol, 4.0 mol%) were added in one portion at rt. The reaction mixture was stirred for 3 h at rt. The reaction mixture was extracted with aqueous $NaHCO_3$ and DCM. Then, the organic layer was dried with Na_2SO_4 and the solvent was removed under reduced

pressure. The residue was purified by flash column chromatography on silica gel to afford the trifluoromethylated products **2**.

2-(Phenyl)-3-(trifluoromethyl)indolizine-1-carbonitrile (2a)

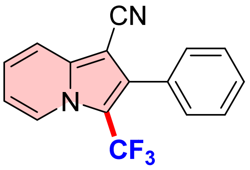

2a

Product **2a** was obtained following the general procedure **A** using 2-(phenyl)indolizine-1-carbonitrile **1a** (43.6 mg, 0.2 mmol, 1.00 eq.). The crude was purified by column chromatography on silica (PE:EtOAc, 6:1) to get the pure product as a yellow wax: 43.5 mg, 0.15 mmol, 76%. ^{1}**H NMR** (400 MHz, $CDCl_3$) δ 8.31 (dq, J = 7.1, 1.0 Hz, 1H), 7.78 (dt, J = 9.0, 1.2 Hz, 1H), 7.55 – 7.40 (m, 5H), 7.32 (ddd, J = 9.0, 6.9, 1.0 Hz, 1H), 7.01 (td, J = 7.0, 1.3 Hz, 1H). ^{13}**C{**^{1}**H} NMR** (100 MHz, $CDCl_3$) δ 139.0, 135.6, 130.4, 129.9, 129.1, 128.5, 125.8, 125.8, 125.8, 125.7, 125.2, 122.8, 120.1, 118.2, 115.1, 115.0, 85.4. ^{19}**F{**^{1}**H} NMR** (377 MHz, $CDCl_3$): δ -57.81 (s, 3 F). **ESI-HRMS**: m/z calcd. for $C_{16}H_9F_3N_2Na$ $[M+Na]^+$ 309.0616, found 309.0610.

2-(Phenyl)-3-(trifluoromethyl)indolizine (2b)

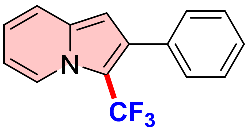

2b

Product **2b** was obtained following the general procedure **A** using 2-(phenyl)indolizine **1b** (38.6 mg, 0.2 mmol, 1.00 eq.). To the crude reaction mixture, CH_2Br_2 (34.7 mg, 14.0 μL, 0.2 mmol, 1.00 equiv.) was added as an internal standard. The reaction mixture was diluted with $CDCl_3$, and the yield was determined by ^{1}H NMR spectroscopy by integration of the peak at δ 4.95 ppm of the internal standard (s, 2H) and the peak at δ 6.50 ppm of the product **2b** (81% NMR yield, **Figure S3**).

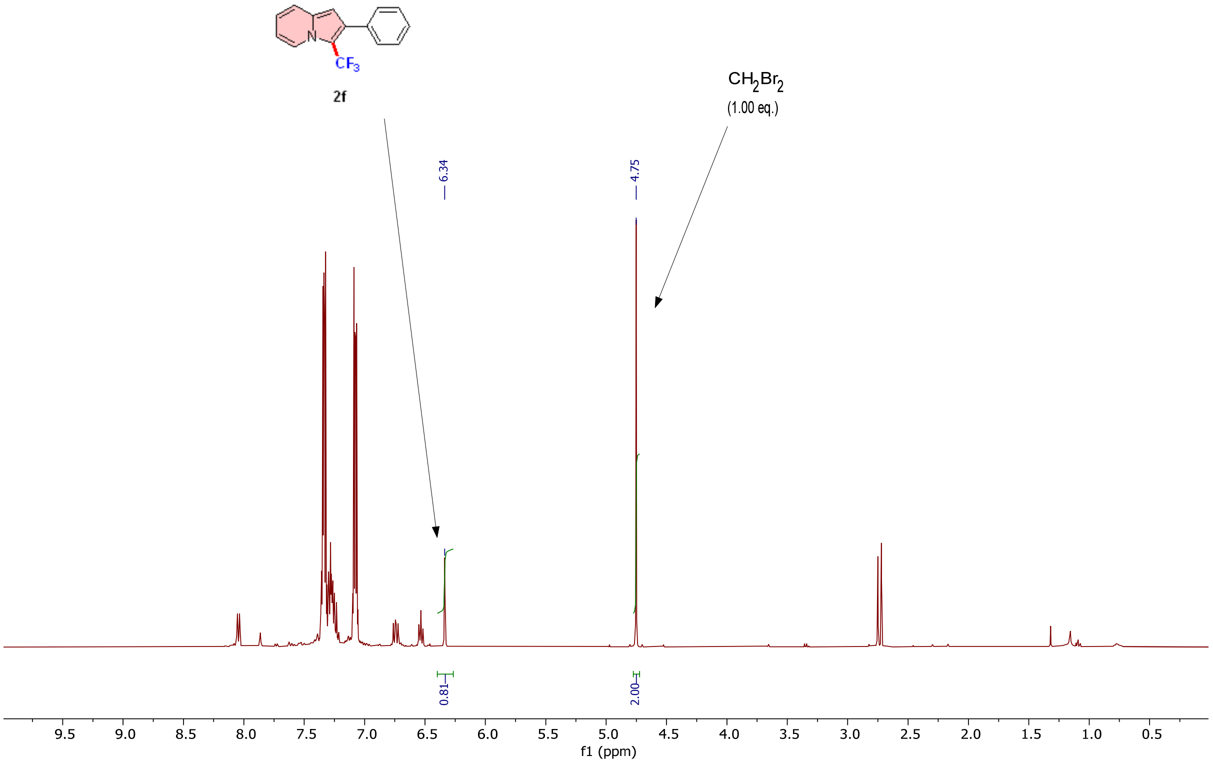

Figure S3. ^{1}H NMR of resulting mixtures for product **2b**.

2-(4-Methoxyphenyl)-3-(trifluoromethyl)indolizine-1-carbonitrile (2c)

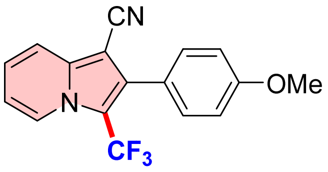

2c

Product **2c** was obtained following the general procedure **A** using 2-(4-methoxyphenyl)indolizine-1-carbonitrile **1c** (49.6 mg, 0.2 mmol, 1.00 eq.). The crude was purified by column chromatography on silica (PE:EtOAc, 4:1) to get the pure product as a white wax: 50.6 mg, 0.16 mmol, 80%. **^{1}H NMR** (400 MHz, $CDCl_3$) δ 8.29 (dq, J = 7.1, 1.0 Hz, 1H), 7.75 (dt, J = 9.0, 1.2 Hz, 1H), 7.47 – 7.40 (m, 2H), 7.34 – 7.27 (m, 1H), 7.05 – 6.95 (m, 3H), 3.87 (s, 3H). **^{13}C{^{1}H} NMR** (100 MHz, $CDCl_3$) δ 160.3, 139.0, 135.5, 135.4, 135.4, 135.4, 131.2, 125.8, 125.8, 125.7, 125.7, 125.5, 125.1, 122.8, 122.6, 120.2, 118.0, 117.5, 115.3, 114.8, 114.4, 114.0, 85.3, 55.4. **^{19}F{^{1}H} NMR** (377 MHz, $CDCl_3$): δ -57.81 (s, 3 F). **ESI-HRMS**: m/z calcd. for $C_{17}H_{11}F_3N_2ONa$ $[M+Na]^+$ 339.0721, found 339.0716.

2-(4-Methoxyphenyl)-3-(trifluoromethyl)indolizine (2d)

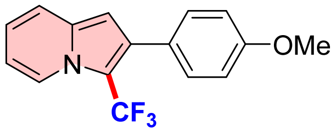

2d

Product **2d** was obtained following the general procedure **A** using 2-(4-methoxyphenyl)indolizine **1d** (44.6 mg, 0.2 mmol, 1.00 eq.). The crude was purified by column chromatography on silica (PE:EtOAc, 98:2) to get the pure product as a colourless oil: 50.1 mg, 0.17 mmol, 86%. **^{1}H NMR** (400 MHz, $CDCl_3$) δ 8.20 (dt, J = 7.2, 0.9 Hz, 1H), 7.47 – 7.39 (m, 3H), 7.01 – 6.95 (m, 2H), 6.95 – 6.86 (m, 1H), 6.70 (td, J = 6.9, 1.4 Hz, 1H), 6.47 (s, 1H), 3.88 (s, 3H). **^{13}C{^{1}H} NMR** (100 MHz, $CDCl_3$) δ 159.2, 134.7, 132.6, 130.8, 127.1, 124.7, 124.6, 124.6, 124.6, 124.0, 121.4, 120.1, 118.9, 113.5, 113.1, 112.0, 101.9, 55.3. **^{19}F{^{1}H} NMR** (377 MHz, $CDCl_3$) δ -56.99 (s, 3F). **ESI-HRMS**: m/z calcd. for $C_{16}H_{12}F_3NO$ $[M+Na]^+$ 308.0893, found 308.0895.

Methyl 2-(4-methoxyphenyl)-3-(trifluoromethyl)indolizine-1-carboxylate (2e)

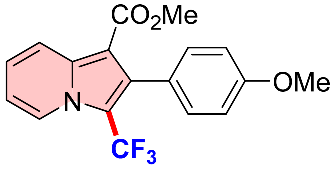

2e

Product **2e** was obtained following the general procedure **A** using methyl 2-(4-methoxyphenyl)indolizine-1-carboxylate **1e** (56.2 mg, 0.2 mmol, 1.00 eq.). The crude was purified by column chromatography on silica (PE:EtOAc, 6:1) to get the pure product as a white wax: 53.1 mg, 0.15 mmol, 76%. **^{1}H NMR** (400 MHz, $CDCl_3$) δ 8.37 (dt, J = 9.2, 1.2 Hz, 1H), 8.27 (dq, J = 7.1, 1.0 Hz, 1H), 7.31 – 7.24 (m, 3H), 6.98 – 6.91 (m, 3H), 3.88 (s, 3H), 3.71 (s, 3H). **^{13}C{^{1}H} NMR** (100 MHz, $CDCl_3$) δ 164.7, 159.2, 137.5, 134.6, 131.1, 130.9, 125.3, 125.1, 125.0, 124.7, 123.3, 120.6, 120.5, 114.2, 113.4, 112.9, 104.0, 55.3, 51.0. **^{19}F{^{1}H} NMR** (377 MHz, $CDCl_3$): δ -57.50 (s, 3F). **ESI-HRMS**: m/z calcd. for $C_{18}H_{14}F_3NO_3Na$ $[M+Na]^+$ 372.0823, found 372.0818.

Ethyl 2-(4-methoxyphenyl)-3-(trifluoromethyl)indolizine-1-carboxylate (2f)

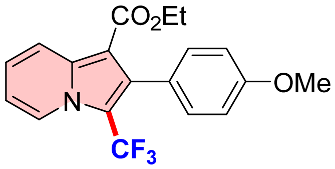

2f

Product **2f** was obtained following the general procedure **A** using ethyl 2-(4-methoxyphenyl)indolizine-1-carboxylate **1f** (57.4 mg, 0.2 mmol, 1.00 eq.). The crude was purified by column chromatography on silica (PE:EtOAc, 6:1) to get the pure product as a colourless oil: 13.8 mg, 0.04 mmol, 19%. **^{1}H NMR** (400 MHz, $CDCl_3$) δ 8.39 (d, J = 9.2 Hz, 1H), 8.26 (dd, J = 7.2, 1.0 Hz, 1H), 7.34 – 7.21 (m, 3H), 6.97 – 6.91 (m, 3H), 4.15 (q, J = 7.1 Hz, 2H), 3.87 (s, 3H), 1.10 (t, J = 7.1 Hz, 3H). **^{13}C{^{1}H} NMR** (100 MHz, $CDCl_3$) δ 164.2, 159.1, 137.4, 131.1, 130.9, 130.8, 125.5, 124.9, 124.4, 120.4, 114.1, 113.3, 113.0, 112.7, 104.3, 59.5, 55.2, 14.0. **^{19}F{^{1}H} NMR** (377 MHz, $CDCl_3$) δ -58.00 (s, 3F). **ESI-HRMS**: m/z calcd. for $C_{19}H_{16}F_3NO_3Na$ $[M+Na]^+$ 386.0980, found 386.0974.

2-(4-Bromophenyl)-3-(trifluoromethyl)indolizine-1-carbonitrile (2g)

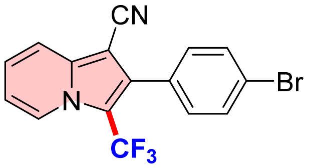

2g

Product **2g** was obtained following the general procedure **A** using methyl 2-(4-bromophenyl)indolizine-1-carbonitrile **1g** (59.4 mg, 0.2 mmol, 1.00 eq.). The crude was purified by column chromatography on silica (PE:EtOAc, 6:1) to get the pure product as a white solid: 64.2 mg, 0.18 mmol, 88%. **^{1}H NMR** (400 MHz, $CDCl_3$) δ 8.30 (dq, J = 7.1, 1.0 Hz, 1H), 7.77 (dt, J = 9.0, 1.3 Hz, 1H), 7.69 – 7.56 (m, 2H), 7.40 – 7.29 (m, 3H), 7.02 (td, J = 7.0, 1.3 Hz, 1H). **^{13}C{^{1}H} NMR** (100 MHz, $CDCl_3$) δ 139.0, 134.2, 134.2, 131.8, 131.5, 129.4, 125.8, 125.8, 125.7, 125.7, 125.4, 123.7, 122.6, 119.9, 118.1, 115.2, 114.8, 85.2. **^{19}F{^{1}H} NMR** (377 MHz, $CDCl_3$) δ -58.28 (s, 3F). **ESI-HRMS**: m/z calcd. for $C_{16}H_8BrF_3N_2Na$ $[M+Na]^+$ 386.9721, found 386.9715.

2-(4-Bromophenyl)-3-(trifluoromethyl)indolizine (2h)

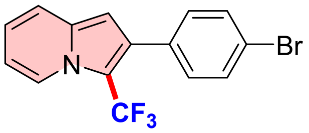

2h

Product **2h** was obtained following the general procedure **C** using 2-(4-bromophenyl)indolizine **1h** (54.4 mg, 0.2 mmol, 1.00 eq.). The crude was purified by column chromatography on silica (100% PE) to get the pure product as a colourless oil: 42.9. mg, 0.13 mmol, 63%. **^{1}H NMR** (400 MHz, $CDCl_3$) δ 8.23 – 8.15 (m, 1H), 7.58 – 7.50 (m, 2H), 7.46 (dt, J = 9.0, 1.3 Hz, 1H), 7.37 – 7.30 (m, 2H), 7.00 – 6.89 (m, 1H), 6.73 (td, J = 6.9, 1.4 Hz, 1H), 6.46 (d, J = 0.8 Hz, 1H). **^{13}C{^{1}H} NMR** (100 MHz, $CDCl_3$) δ 134.8, 133.7, 131.5, 131.3, 131.1, 128.7, 127.7, 124.6, 124.6, 121.8, 120.3, 119.1, 112.4, 101.8. **^{19}F{^{1}H} NMR**

(377 MHz, $CDCl_3$) δ -57.02 (s, 3F). **ESI-HRMS**: m/z calcd. for $C_{15}H_9BrF_3NNa$ $[M+Na]^+$ 361.9768, found 361.9774.

3-(Trifluoromethyl)indolizine-1-carbonitrile (2i)

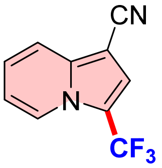

2i

Product **2i** was obtained following the general procedure **A** using indolizine-1-carbonitrile **1i** (28.4 mg, 0.2 mmol, 1.00 eq.). The crude was purified by column chromatography on silica (PE:EtOAc, 8:1) to get the pure product as a white wax: 16.0 mg, 0.08 mmol, 38%. **^{1}H NMR** (400 MHz, $CDCl_3$) δ 8.22 (dd, J = 7.1, 1.0 Hz, 1H), 7.77 (dt, J = 9.1, 1.2 Hz, 1H), 7.42 (d, J = 0.8 Hz, 1H), 7.30 (ddd, J = 9.0, 6.8, 1.0 Hz, 1H), 7.00 (td, J = 7.0, 1.3 Hz, 1H). **^{13}C{^{1}H} NMR** (100 MHz, $CDCl_3$) δ 139.9, 125.5, 125.5, 125.0, 122.2, 119.5, 119.0, 118.9, 118.9, 118.9, 118.6, 115.4, 115.0, 83.0. **^{19}F{^{1}H} NMR** (377 MHz, $CDCl_3$) δ -62.43 (s, 3F). **ESI-HRMS**: m/z calcd. for $C_{10}H_5F_3N_2Na$ $[M+Na]^+$ 233.0303, found 233.0297.

Methyl 2-(phenyl)-3-(trifluoromethyl)indolizine-1-carboxylate (2j)

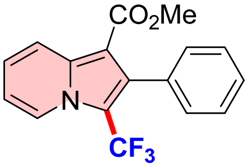

2j

Product **2j** was obtained following the general procedure **A** using methyl 2-(phenyl)indolizine-1-carboxylate **1j** (50.2 mg, 0.2 mmol, 1.00 eq.). The crude was purified by column chromatography on silica (PE:EtOAc, 7:1) to get the pure product as a yellow wax: 47.9 mg, 0.15 mmol, 75%. **^{1}H NMR** (400 MHz, $CDCl_3$) δ 8.38 (d, J = 9.2 Hz, 1H), 8.28 (dd, J = 7.1, 1.0 Hz, 1H), 7.42 – 7.38 (m, 3H), 7.36 – 7.31 (m, 2H), 7.29 (ddd, J = 9.2, 6.8, 1.1 Hz, 1H), 6.95 (td, J = 6.9, 1.4 Hz, 1H), 3.67 (s, 3H). **^{13}C{^{1}H} NMR** (100 MHz, $CDCl_3$) δ 164.7, 137.5, 133.3, 130.0, 129.6, 127.9, 127.7, 127.7, 127.4, 125.1, 125.1, 124.8, 123.2, 120.6, 114.4, 104.0, 51.0, 51.0. **^{19}F{^{1}H} NMR** (377 MHz, $CDCl_3$) δ -57.54 (s, 3F). **ESI-HRMS**: m/z calcd. for $C_{17}H_{12}F_3NO_2Na$ $[M+Na]^+$ 342.0718, found 342.0712.

Methyl 2-(4-(trifluoromethyl)phenyl)-3-(trifluoromethyl)indolizine-1-carboxylate (2k)

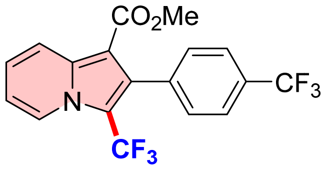

2k

Product **2k** was obtained following the general procedure **A** using methyl 2-(4-(trifluoromethyl)phenyl)indolizine-1-carboxylate **1k** (63.8 mg, 0.2 mmol, 1.00 eq.). The crude was purified by column chromatography on silica (PE:EtOAc, 7:1) to get the pure product as a yellow wax: 54.2 mg, 0.14 mmol, 70%. **^{1}H NMR** (400 MHz, $CDCl_3$) δ 8.40 (dt, J = 9.2, 1.2 Hz, 1H), 8.29 (dq, J = 7.1, 0.9 Hz, 1H), 7.72 – 7.64 (m, 2H), 7.52 – 7.43 (m, 2H), 7.32 (ddd, J = 9.2, 6.8, 1.1 Hz, 1H), 6.99 (td, J = 6.9, 1.4 Hz, 1H), 3.68 (s, 3H). **^{13}C{^{1}H} NMR** (100 MHz, $CDCl_3$) δ 164.4, 137.5, 137.4, 133.1, 133.1, 130.4, 130.1, 129.8, 125.8, 125.1, 125.1, 125.1, 125.0, 125.0, 124.8, 124.8, 124.5, 124.4, 124.4, 124.3, 123.1, 120.7, 120.4, 114.7, 103.9, 51.1. **^{19}F{^{1}H} NMR** (377 MHz, $CDCl_3$) δ -58.01 (s, 3F), -62.96 (s, 3F). **ESI-HRMS**: m/z calcd. for $C_{18}H_{11}F_6NO_2Na$ $[M+Na]^+$ 410.0592, found 410.0586.

2-(4-Hydroxyphenyl)-3-(trifluoromethyl)indolizine (2l)

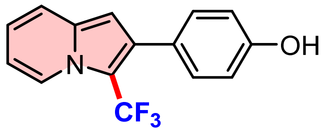

2l

Product **2l** was obtained following the general procedure **A** using 2-(4-hydroxyphenyl)indolizine **1l** (41.9 mg, 0.2 mmol, 1.00 eq.). The crude was purified by column chromatography on silica (PE:EtOAc, 6:1) to get the pure product as a red oil: 33.3 mg, 0.12 mmol, 60%. **^{1}H NMR** (400 MHz, DMSO[d^6]) δ 9.60 (s, 1H), 8.29 (dq, J = 7.2, 1.0 Hz, 1H), 7.61 (dt, J = 9.0, 1.3 Hz, 1H), 7.28 – 7.20 (m, 2H), 7.08 – 7.02 (m, 1H), 6.90 – 6.79 (m, 3H), 6.54 (d, J = 0.9 Hz, 1H). **^{13}C{^{1}H} NMR** (100 MHz, DMSO[d^6]) δ 157.6, 135.0, 133.2, 133.2, 130.9, 125.0, 124.9, 124.4, 121.8, 121.4, 119.4, 115.5, 115.1, 113.3, 106.6, 106.3, 102.3. **^{19}F{^{1}H} NMR** (377 MHz, DMSO[d^6]) δ -55.78 (s, 3F). **ESI-HRMS**: m/z calcd. for $C_{30}H_{17}F_6N_2O$ $[2M-H_3O]^+$ 535.1240, found 535.1237.

2-(4-Hydroxyphenyl)-3-(trifluoromethyl)indolizine-1-carbonitrile (2m)

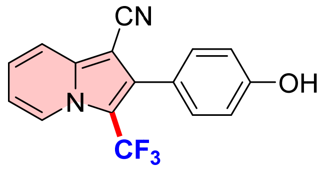

2m

Product **2m** was obtained following the general procedure **A** using 2-(4-hydroxyphenyl)indolizine-1-carbonitrile **1m** (46.8 mg, 0.2 mmol, 1.00 eq.). The crude was purified by column chromatography on silica (PE:EtOAc, 2:1) to get the pure product as a yellow solid: 35.7 mg, 0.12 mmol, 59%. ^{1}**H NMR** (400 MHz, DMSO[d^6]) δ 9.86 (bs, 1H), 8.53 (dt, J = 7.1, 1.1 Hz, 1H), 7.82 (dt, J = 9.0, 1.2 Hz, 1H), 7.54 – 7.45 (m, 1H), 7.43 – 7.26 (m, 2H), 7.20 (td, J = 7.0, 1.3 Hz, 1H), 6.99 – 6.85 (m, 2H). ^{13}**C{^{1}H} NMR** (100 MHz, DMSO[d^6]) δ 158.7, 138.8, 135.7, 131.4, 127.0, 126.7, 126.7, 126.7, 123.0, 120.8, 120.4, 117.7, 116.3, 115.7, 115.3, 109.1, 108.7, 84.4. ^{19}**F{^{1}H} NMR** (377 MHz, DMSO[d^6]) δ -56.95 (s, 3F). **ESI-HRMS**: m/z calcd. for $C_{16}H_9F_3N_2ONa$ $[M+Na]^+$ 325.0565, found 325.0559.

2-(2,4-Dichlorophenyl)-3-(trifluoromethyl)indolizine-1-carbonitrile (2n)

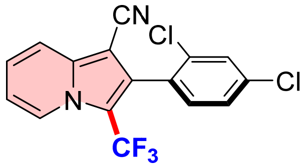

2n

Product **2n** was obtained following the general procedure **A** using methyl 2-(2,4-dichlorophenyl)indolizine-1-carbonitrile **1n** (57.4 mg, 0.2 mmol, 1.00 eq.). The crude was purified by column chromatography on silica (PE:EtOAc, 6:1) to get the pure product as a colourless oil: 35.5 mg, 0.10 mmol, 50%. ^{1}**H NMR** (400 MHz, $CDCl_3$) δ 8.29 (dq, J = 7.2, 1.0 Hz, 1H), 7.79 (dt, J = 9.0, 1.2 Hz, 1H), 7.56 (d, J = 2.0 Hz, 1H), 7.40 – 7.30 (m, 3H), 7.05 (td, J = 7.0, 1.3 Hz, 1H). ^{13}**C{^{1}H} NMR** (100 MHz, $CDCl_3$) δ 138.8, 136.1, 134.8, 132.3, 129.8, 128.4, 127.3, 125.7, 125.6, 125.6, 125.4, 122.3, 119.6, 118.3, 115.3, 114.3, 85.7. ^{19}**F{^{1}H} NMR** (377 MHz, $CDCl_3$) δ -60.59 (s, 3F). **ESI-HRMS**: m/z calcd. for $C_{16}H_7Cl_2F_3N_2Na$ $[M+Na]^+$ 376.9836, found 376.9831.

2-(3,5-Bis(trifluoromethyl)phenyl)-3-(trifluoromethyl)indolizine-1-carbonitrile (2o)

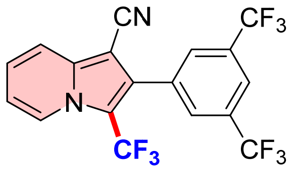

2o

Product **2o** was obtained following the general procedure **A** using 2-(3,5-bis(trifluoromethyl)phenyl)indolizine-1-carbonitrile **1o** (70.8 mg, 0.2 mmol, 1.00 eq.). The crude was purified by column chromatography on silica (PE:EtOAc, 6:1) to get the pure product as a brown wax: 65.9 mg, 0.16 mmol, 78%. **^{1}H NMR** (400 MHz, $CDCl_3$) δ 8.38 – 8.29 (m, 1H), 8.01 (s, 1H), 7.94 (s, 2H), 7.83 (dt, J = 9.0, 1.2 Hz, 1H), 7.41 (ddd, J = 9.1, 6.9, 1.0 Hz, 1H), 7.10 (td, J = 7.0, 1.3 Hz, 1H). **^{13}C{^{1}H} NMR** (100 MHz, $CDCl_3$) δ 139.2, 132.7, 132.6, 132.3, 132.0, 131.6, 130.2, 126.0, 125.8, 125.8, 124.5, 123.2, 123.1, 123.1, 122.4, 121.8, 119.7, 118.4, 115.8, 114.1, 85.5. **^{19}F{^{1}H} NMR** (377 MHz, $CDCl_3$): δ -57.93 (s, 3F), -62.92 (s, 6F). **ESI-HRMS**: m/z calcd. for $C_{18}H_7F_9N_2Na$ $[M+Na]^+$ 445.0364, found 445.0358.

2-(3,5-Bis(trifluoromethyl)phenyl)-3-(trifluoromethyl)indolizine (2p)

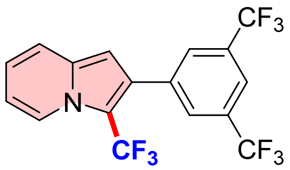

2p

Product **2p** was obtained following the general procedure **B** using 2-(3,5-bis(trifluoromethyl)phenyl)indolizine **1p** (79.4 mg, 0.2 mmol, 1.00 eq.). The crude was purified by column chromatography on silica (100% PE) to get the pure product as a yellow oil: 69.1 mg, 0.17 mmol, 87%. **^{1}H NMR** (400 MHz, $CDCl_3$) δ 8.22 (dp, J = 7.3, 1.0 Hz, 1H), 7.90 (d, J = 3.4 Hz, 3H), 7.51 (dt, J = 9.0, 1.3 Hz, 1H), 7.06 – 6.92 (m, 1H), 6.80 (td, J = 6.9, 1.4 Hz, 1H), 6.54 (d, J = 0.9 Hz, 1H). **^{13}C{^{1}H} NMR** (100 MHz, $CDCl_3$) δ 137.1, 135.1, 132.0, 131.7, 131.3, 131.0, 129.9, 129.6, 124.8, 124.8, 124.7, 124.7, 124.6, 123.7, 122.1, 121.5, 121.5, 121.4, 121.4, 121.4, 121.0, 119.4, 113.2, 102.1. **^{19}F{^{1}H} NMR** (377 MHz, $CDCl_3$) δ -57.67 (s, 3F), -63.36 (s, 6F). **ESI-HRMS**: m/z calcd. for $C_{17}H_8F_9N$ $[M+Na]^+$ 414.0535, found 414.0538.

2-(4-(Trifluoromethyl)phenyl)-3-(trifluoromethyl)indolizine (2q)

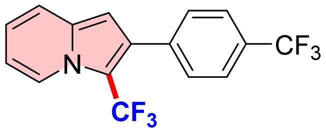

2q

Product **2q** was obtained following the general procedure **B** using 2-(4-(trifluoromethyl)phenyl)indolizine **1q** (52.2 mg, 0.2 mmol, 1.00 eq.). The crude was purified by column chromatography on silica (100% PE) to get the pure product as a colourless oil: 52.6 mg, 0.16 mmol, 80%. **^{1}H NMR** (400 MHz, $CDCl_3$) δ 8.21 (dt, J = 7.2, 1.0 Hz, 1H), 7.68 (d, J = 8.3 Hz, 2H), 7.58 (d, J =

8.0 Hz, 2H), 7.48 (d, J = 9.0 Hz, 1H), 6.98 – 6.94 (m, 1H), 6.76 (td, J = 6.9, 1.4 Hz, 1H), 6.49 (d, J = 0.9 Hz, 1H). **^{13}C{^{1}H} NMR** (100 MHz, $CDCl_3$) δ 138.5, 134.8, 131.2, 130.0, 129.8, 129.4, 125.6, 124.9, 124.9, 124.9, 124.8, 124.6, 124.6, 123.7, 122.9, 121.1, 120.5, 119.2, 112.6, 101.9. **^{19}F{^{1}H} NMR** (377 MHz, $CDCl_3$) δ -57.51 (s, 3F), -62.96 (s, 3F). **ESI-HRMS**: m/z calcd. for $C_{16}H_9F_6N$ $[M+Na]^+$ 346.0661, found 346.0661.

2-(2-Methoxyphenyl)-3-(trifluoromethyl)indolizine-1-carbonitrile (2r)

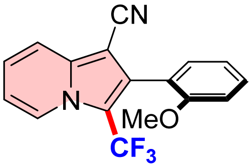

2r

Product **2r** was obtained following the general procedure **A** using 2-(2-methoxyphenyl)indolizine **1r** (44.9 mg, 0.2 mmol, 1.00 eq.). The crude was purified by column chromatography on silica (PE:EtOAc, 6:1) to get the pure product as a colourless oil: 44.9 mg, 0.14 mmol, 71%. **^{1}H NMR** (400 MHz, $CDCl_3$) δ 8.17 (dq, J = 7.3, 1.1 Hz, 1H), 7.65 (dt, J = 9.0, 1.2 Hz, 1H), 7.34 (ddd, J = 8.4, 7.5, 1.8 Hz, 1H), 7.27 (dd, J = 7.5, 1.7 Hz, 1H), 7.23 – 7.12 (m, 1H), 6.98 (td, J = 7.5, 1.1 Hz, 1H), 6.93 (dd, J = 8.4, 1.0 Hz, 1H), 6.87 (td, J = 7.0, 1.3 Hz, 1H), 3.74 (s, 3H). **^{13}C{^{1}H} NMR** (100 MHz, $CDCl_3$) δ 157.0, 138.9, 131.5, 131.2, 130.8, 125.6, 125.6, 125.5, 125.5, 124.6, 122.7, 120.6, 120.0, 119.5, 118.0, 115.2, 114.6, 111.4, 111.1, 85.7, 55.5. **^{19}F{^{1}H} NMR** (377 MHz, $CDCl_3$) δ -60.49 (s, 3F). **ESI-HRMS**: m/z calcd. for $C_{17}H_{11}F_3N_2ONa$ $[M+Na]^+$ 339.0721, found 339.0716.

Dimethyl 3-(trifluoromethyl)indolizine-1,2-dicarboxylate (2s) and Dimethyl 6-(trifluoromethyl)indolizine-1,2-dicarboxylate (2s')

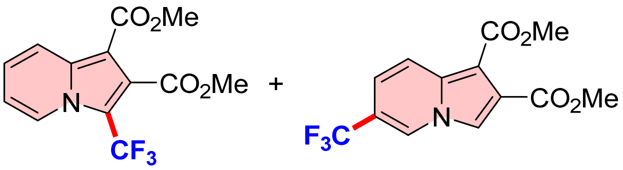

2s + 2s' (4:1)

Product **2s** and **2s'** were obtained as a 4:1 mixture respectively following the general procedure **A** using dimethyl indolizine-1,2-dicarboxylate **1s** (46.6 mg, 0.2 mmol, 1.00 eq.). The crude was purified by column chromatography on silica (PE:EtOAc, 6:1) to get the products as a white wax: 46.3 mg, 0.15 mmol, 77%. **^{1}H NMR** (400 MHz, $CDCl_3$) δ 8.35 (dt, J = 9.1, 0.7 Hz, 1H), 8.30 (dt, J = 9.2, 1.2 Hz, 1H), 8.17 (dp, J = 7.1, 0.9 Hz, 1H), 7.86 (tt, J = 2.0, 1.3 Hz, 1H), 7.30 (ddd, J = 9.3, 6.8, 1.1 Hz, 1H), 7.24 (ddd, J = 7.0, 1.3, 0.7 Hz, 1H), 7.10 (ddd, J = 9.2, 7.0, 0.8 Hz, 1H), 6.98 (td, J = 6.9, 1.3 Hz, 1H), 3.99 (s, 3H), 3.94 (s, 3H), 3.93 (s, 3H) 3.90 (s, 3H). **^{13}C{^{1}H} NMR** (100 MHz, $CDCl_3$) δ 165.1, 164.6, 163.9,

163.3, 136.7, 136.3, 125.6, 125.5, 125.5, 125.0, 125.0, 125.0, 124.9, 124.6, 122.7, 122.2, 120.9, 120.7, 119.6, 116.9, 116.6, 115.3, 114.2, 114.2, 110.7, 110.3, 105.3, 102.3, 53.2, 52.5, 51.8, 51.8. **^{19}F{^{1}H} NMR** (377 MHz, $CDCl_3$) δ -61.03 (s 3F), -69.57 (s, 3F). **ESI-HRMS**: m/z calcd. for $C_{13}H_{10}F_3NO_4Na$ $[M+Na]^+$ 324.0460, found 324.0454.

1-Phenyl-2-(4-methoxyphenyl)-3-(trifluoromethyl)indolizine (2t)

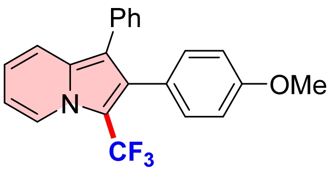

2t

Product **2t** was obtained following the general procedure **A** using 1-phenyl-2-(4-methoxyphenyl)indolizine **1t** (59.8 mg, 0.2 mmol, 1.00 eq.). The crude was purified by column chromatography on silica (PE:EtOAc, 98:2) to get the pure product as a colourless oil: 63.2 mg, 0.17 mmol, 86%. **^{1}H NMR** (400 MHz, $CDCl_3$) δ 8.24 (dq, J = 7.2, 1.0 Hz, 1H), 7.61 (dt, J = 9.2, 1.2 Hz, 1H), 7.31 – 7.26 (m, 2H), 7.26 – 7.21 (m, 1H), 7.21 – 7.16 (m, 4H), 6.95 – 6.89 (m, 1H), 6.89 – 6.83 (m, 2H), 6.74 (td, J = 6.8, 1.3 Hz, 1H), 3.83 (s, 3H). **^{13}C{^{1}H} NMR** (100 MHz, $CDCl_3$) δ 159.0, 133.8, 132.6, 132.0, 130.5, 130.5, 130.4, 130.3, 128.7, 128.4, 126.2, 125.5, 124.6, 124.6, 124.5, 124.5, 124.1, 121.5, 120.6, 118.3, 115.3, 113.5, 113.3, 112.7, 55.2. **^{19}F{^{1}H} NMR** (377 MHz, $CDCl_3$) δ -57.35 (s, 3F). **ESI-HRMS**: m/z calcd. for $C_{22}H_{16}F_3NONa$ $[M+Na]^+$ 390.1082, found 390.1073.

2-(Thiophen-2-yl)-3-(trifluoromethyl)indolizine-1-carbonitrile (2u)

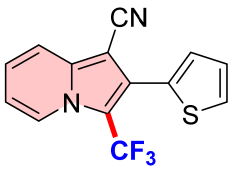

2u

Product **2u** was obtained following the general procedure **A** using 2-(thiophen-2-yl)indolizine-1-carbonitrile **1u** (44.8 mg, 0.2 mmol, 1.00 eq.). The crude was purified by column chromatography on silica (PE:EtOAc, 9:1) to get the pure product as a colourless oil: 46.8 mg, 0.16 mmol, 80%. **^{1}H NMR** (400 MHz, $CDCl_3$) δ 8.30 (dq, J = 7.1, 1.0 Hz, 1H), 7.75 (dt, J = 9.1, 1.3 Hz, 1H), 7.52 (dd, J = 5.2, 1.2 Hz, 1H), 7.34 – 7.29 (m, 2H), 7.16 (dd, J = 5.1, 3.6 Hz, 1H), 7.01 (td, J = 7.0, 1.4 Hz, 1H). **^{13}C{^{1}H} NMR** (100 MHz, $CDCl_3$) δ 138.9, 129.7, 129.7, 129.7, 128.1, 127.9, 127.9, 127.4, 125.7, 125.7, 125.6, 125.6, 125.3, 122.5, 119.8, 118.0, 115.1, 114.7, 86.1. **^{19}F{^{1}H} NMR** (377 MHz, $CDCl_3$) δ -58.20 (s, 3F). **ESI-HRMS**: m/z calcd. for $C_{14}H_7F_3N_2SNa$ $[M+Na]^+$ 315.0180, found 315.0174.

2-(Thiophen-2-yl)-3-(trifluoromethyl)indolizine (2v)

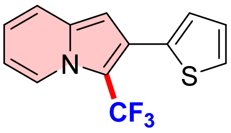

2v

Product **2v** was obtained following the general procedure **A** using 2-(thiophen-2-yl)indolizine **1v** (39.8 mg, 0.2 mmol, 1.00 eq.). To the crude reaction mixture, CH_2Br_2 (34.7 mg, 14.0 μL, 0.2 mmol, 1.00 equiv.) was added as an internal standard. The reaction mixture was diluted with $CDCl_3$, and the yield was determined by ^1H NMR spectroscopy by integration of the peak at δ 4.95 ppm of the internal standard (s, 2H) and the peak at δ 6.50 ppm of the product **2v** (55% NMR yield, **Figure S4**).

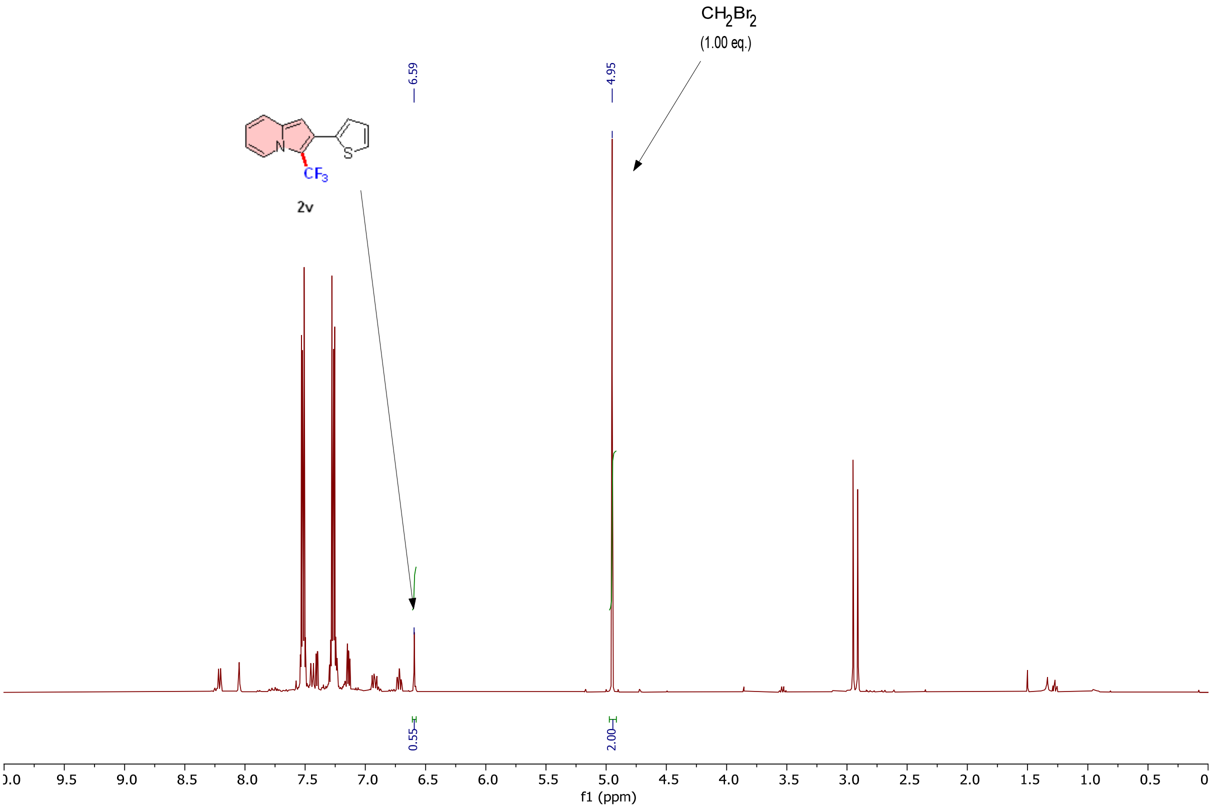

Figure S4. ^1H NMR of resulting mixtures for product **2v**.

Methyl 2-(thiophen-2-yl)-3-(trifluoromethyl)indolizine-1-carboxylate (2w)

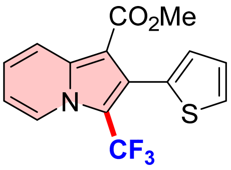

2w

Product **2w** was obtained following the general procedure **A** using methyl 2-(thiophen-2-yl)indolizine-1-carboxylate **1w** (51.4 mg, 0.2 mmol, 1.00 eq.). The crude was purified by column chromatography on silica (PE:EtOAc, 30:1) to get the pure product as a colourless oil: 56.0 mg, 0.17 mmol, 86%. **^{1}H NMR** (400 MHz, $CDCl_3$) δ 8.37 (dt, J = 9.2, 1.3 Hz, 1H), 8.27 (dq, J = 7.1, 0.9 Hz, 1H), 7.45 (dd, J = 5.1, 1.3 Hz, 1H), 7.30 – 7.25 (m, 1H), 7.10 (dd, J = 5.1, 3.5 Hz, 1H), 7.05 (dd, J = 3.5, 1.3 Hz, 1H), 6.95 (td, J = 6.9, 1.4 Hz, 1H), 3.74 (s, 3H). **^{13}C{^{1}H} NMR** (100 MHz, $CDCl_3$) δ 164.3, 137.4, 132.6, 128.5, 126.7, 126.7, 126.5, 126.4, 125.0, 124.9, 124.9, 124.9, 124.8, 123.0, 120.6, 120.3, 114.6, 105.2, 51.1. **^{19}F{^{1}H} NMR** (377 MHz, $CDCl_3$) δ -58.38 (s, 3F). **ESI-HRMS**: m/z calcd. for $C_{15}H_{10}F_3NO_2SNa$ $[M+Na]^+$ 348.0282, found 348.0277.

6.5. Gram scale reaction and product diversification

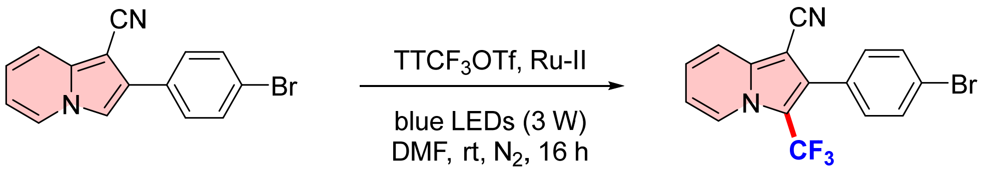

1g (2.0 mmol) **2g**

In a glovebox, to a vial filled with the indolizine **1g** (0.594 g, 2.0 mmol, 1.00 eq.), $TTCF_3OTf$ (0.954 g, 2.2 mmol, 1.1 eq.), **Ru-II** (31.6 mg, 20 µmol, 1.0 mol%) was added DMF (10.0 mL, 0.2 M). The vial was sealed and the mixture was irradiated with a blue LED (3 W, 455 – 460 nm) for 16 h at rt outside of the glovebox. The reaction mixture was partitioned between H_2O and Et_2O and the organic phase was extracted. The water phase was extracted with Et_2O 3 times and the combined organic phases were washed with H_2O and brine. The organic phase was dried over Na_2SO_4 and the solvent was removed under reduced pressure. The residue was purified by flash column chromatography on silica gel (PE:EtOAc, 6:1) to afford the trifluoromethylated product **2g** (0.489 g, 1.3 mmol, 67%) as a white solid. Spectral data sets were in agreement with previous measurements.

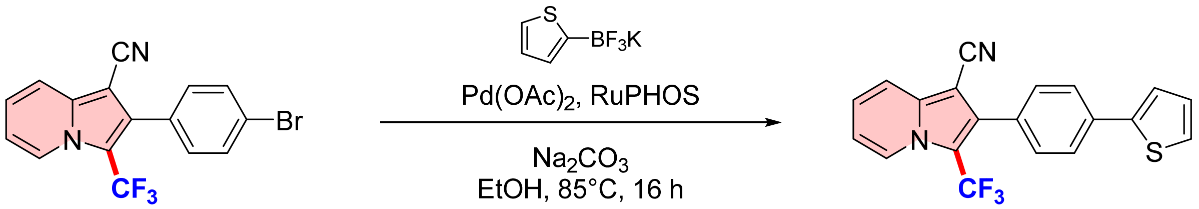

2g **3**

Under inert atmosphere, **2g** (100.0 mg, 0.27 mmol, 1.00 eq.), potassium 2-thiophenyltrifluoroborate (62.4 mg, 0.33 mmol, 1.20 eq.), $Pd(OAc)_2$ (1.8 mg, 8.2 µmol, 3.0 mol%), RuPHOS (7.7 mg, 16.4 µmol, 6.0 mol%) and Na_2CO_3 (72.5 mg, 0.68 mmol, 2.50 eq.) were suspended in absolute EtOH (1.5 mL, 0.18

M). The resulting mixture was stirred at 85°C for 16 h. The mixture was allowed to cool down to rt, filtered through a pad of Celite (eluting with EtOAc) and the filtrate was concentrated under reduced pressure. The residue was purified by flash column chromatography on silica gel (PE:EtOAc, 6:1) to afford the trifluoromethylated products **3** (100.8 mg, 0.27 mmol, quant.) as a white solid. **^{1}H NMR** (400 MHz, $CDCl_3$) δ 8.36 – 8.24 (m, 1H), 7.80 – 7.69 (m, 3H), 7.53 (d, J = 8.1 Hz, 2H), 7.40 (dd, J = 3.6, 1.2 Hz, 1H), 7.36 – 7.26 (m, 2H), 7.12 (dd, J = 5.1, 3.6 Hz, 1H), 6.98 (td, J = 7.0, 1.3 Hz, 1H). **^{13}C{^{1}H} NMR** (100 MHz, $CDCl_3$) δ 143.5, 143.3, 139.0, 135.5, 135.1, 134.9, 134.9, 134.8, 130.4, 130.1, 129.3, 128.3, 128.2, 126.1, 125.8, 125.7, 125.7, 125.7, 125.6, 125.5, 125.4, 125.2, 123.9, 123.7, 123.4, 122.7, 122.1, 120.0, 118.0, 117.3, 115.0, 115.0, 110.7, 110.3, 109.9, 109.5, 85.1. **^{19}F{^{1}H} NMR** (377 MHz, $CDCl_3$) δ -58.14 (s, 3F). **ESI-HRMS**: m/z calcd. for $C_{20}H_{11}F_3N_2SNa$ $[M+Na]^+$ 391.0493, found 391.0487.

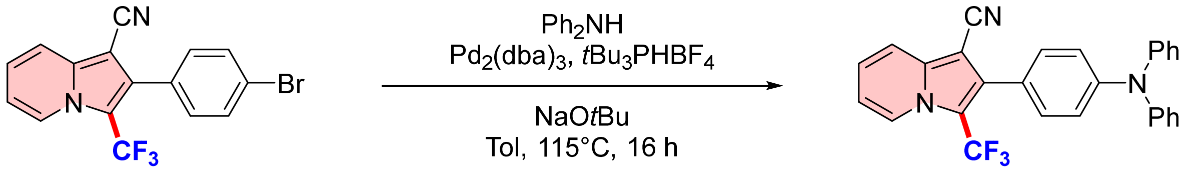

2l **4**

Under inert atmosphere, **2g** (100.0 mg, 0.27 mmol, 1.15 eq.), diphenylamine (40.0 mg, 0.24 mmol, 1.00 eq.), $Pd_2(dba)_3$ (6.5 mg, 7.1 µmol, 3.0 mol%), *t*Bu$_3$PHBF$_4$ (4.1 mg, 14.2 µmol, 6.0 mol%) and NaO*t*Bu (68.6 mg, 0.71 mmol, 3.00 eq.) were suspended in Toluene (2.0 mL, 1.20 M). The resulting mixture was stirred at 115°C for 16 h. The mixture was allowed to cool down to rt, filtered through a pad of Celite (eluting with EtOAc) and the filtrate was concentrated under reduced pressure. The residue was purified by flash column chromatography on silica gel (PE:EtOAc, 6:1) to afford the trifluoromethylated products **4** (101.5 mg, 0.22 mmol, 94%) as a white solid. **^{1}H NMR** (400 MHz, $CDCl_3$) δ 8.27 (dq, J = 7.1, 1.0 Hz, 1H), 7.73 (dt, J = 9.0, 1.2 Hz, 1H), 7.38 – 7.23 (m, 7H), 7.21 – 7.15 (m, 4H), 7.15 – 7.10 (m, 2H), 7.10 – 7.03 (m, 2H), 6.95 (td, J = 7.0, 1.3 Hz, 1H). **^{13}C{^{1}H} NMR** (100 MHz, $CDCl_3$) δ 148.6, 147.3, 139.0, 135.4, 135.4, 130.7, 130.7, 130.6, 129.6, 129.5, 125.8, 125.7, 125.7, 125.6, 125.5, 125.5, 125.2, 125.0, 123.6, 123.5, 123.2, 122.8, 121.8, 120.1, 117.9, 117.5, 115.3, 114.8, 110.5, 110.1, 109.7, 109.4, 85.0. **^{19}F{^{1}H} NMR** (377 MHz, $CDCl_3$) δ -58.12 (s, 3F). **ESI-HRMS**: m/z calcd. for $C_{28}H_{18}F_3N_3Na$ $[M+Na]^+$ 476.1351, found 476.1345.

6.6. Synthesis of analogues of biologically active compounds

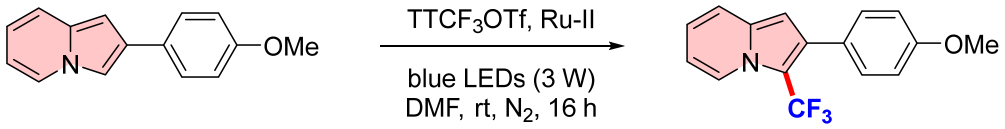

1c (2.0 mmol) **2c**

In a glovebox, to a vial filled with the indolizine **1c** (0.447 g, 2.0 mmol, 1.00 eq.), $TTCF_3OTf$ (0.954 g, 2.2 mmol, 1.1 eq.), **Ru-II** (31.6 mg, 20 µmol, 1.0 mol%) was added DMF (10.0 mL, 0.2 M). The vial was sealed and the mixture was irradiated with a blue LED (3 W, 455 – 460 nm) for 16 h at rt outside of the glovebox. The reaction mixture was partitioned between H_2O and Et_2O and the organic phase was extracted. The water phase was extracted with Et_2O 3 times and the combined organic phases were washed with H_2O and brine. The organic phase was dried over Na_2SO_4 and the solvent was removed under reduced pressure. The residue was purified by flash column chromatography on silica gel (PE:EtOAc, 98:2) to afford the trifluoromethylated products **2c** (0.437 g, 1.5 mmol, 75%) as a white solid. Spectral data sets were in agreement with previous measurements.

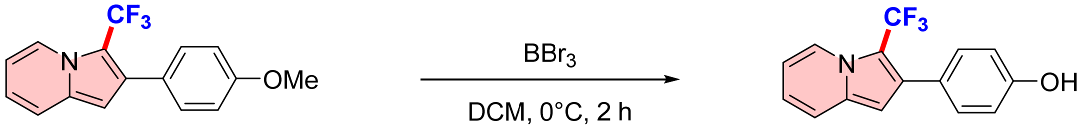

2c **2l**

Under inert atmosphere, to a solution of indolizine **2c** (0.410 g, 1.4 mmol, 1.00 eq.) in anhydrous DCM (11.0 mL, 0.13 M) at 0°C, BBr_3 1 M solution in DCM (3.0 mL, 2.8 mmol, 2.00 eq.) is added dropwise. The reaction mixture is stirred at 0°C for 2 h. The reaction in quenched by addition of water and the DCM layer is separated. The water phase was extracted with DCM 3 times and the combined organic phases were washed with H_2O and brine. The organic phase was dried over Na_2SO_4 and the solvent was removed under reduced pressure. The residue was purified by flash column chromatography on silica gel (PE:EtOAc, 4:1) to afford the trifluoromethylated products **2l** (0.379 g, 1.4 mmol, 97%) as a deep red solid. Spectral data sets were in agreement with previous measurements.

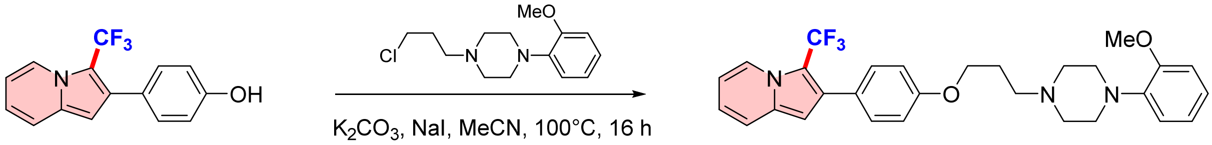

2l **5**

Indolizine **2l** (0.130 g, 0.47 mmol, 1.00 eq.), 1-(3-chloropropyl)-4-(2-methoxyphenyl)piperazine (0.126 g, 0.47 mmol, 1.00 eq.), K_2CO_3 (0.130 g, 0.94 mmol, 2.00 eq.) and NaI (7.5 mg, 0.05 mol, 10.0 mol%)

are suspended in anhydrous MeCN (5 mL, 0.10 M). The reaction mixture is stirred overnight at 100°C and it's later cooled to rt. The mixture is partitioned between DCM/H_2O and the organic phase is separated. The water phase was extracted with DCM 3 times and the combined organic phases were washed with H_2O and brine. The organic phase was dried over Na_2SO_4 and the solvent was removed under reduced pressure. The residue was purified by flash column chromatography on silica gel (PE:EtOAc, 2:1) to afford the trifluoromethylated products **5** (0.055 g, 0.11 mmol, 23%) as a yellow oil. **^{1}H NMR** (400 MHz, $CDCl_3$) δ 8.19 (dt, J = 7.3, 1.0 Hz, 1H), 7.44 (dt, J = 9.0, 1.3 Hz, 1H), 7.39 (d, J = 8.3 Hz, 2H), 7.06 – 6.85 (m, 7H), 6.69 (td, J = 6.9, 1.4 Hz, 1H), 6.45 (d, J = 0.8 Hz, 1H), 4.11 (t, J = 6.3 Hz, 2H), 3.88 (s, 3H), 3.18 (s, 4H), 2.89 – 2.63 (m, 6H), 2.11 (p, J = 6.3 Hz, 2H). **^{13}C{^{1}H} NMR** (100 MHz, $CDCl_3$) δ 158.5, 152.3, 141.2, 134.7, 132.6, 132.6, 130.8, 127.1, 124.6, 124.6, 124.6, 124.5, 124.0, 123.0, 121.4, 121.0, 120.0, 118.9, 118.3, 114.0, 112.0, 111.2, 101.9, 66.2, 55.4, 55.3, 53.5, 50.4, 26.6. **^{19}F{^{1}H} NMR** (377 MHz, $CDCl_3$) δ -57.45 (s, 3F). **ESI-HRMS**: m/z calcd. for $C_{29}H_{30}F_3N_3O_2Na$ $[M+Na]^+$ 532.2188, found 532.2182.

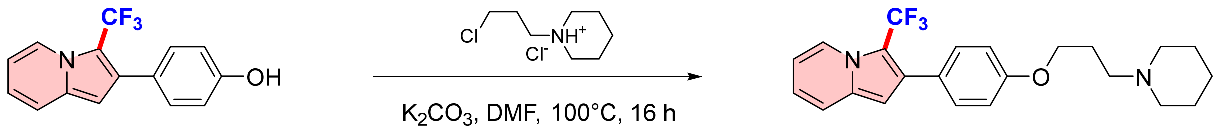

2l **6**

Indolizine **2l** (0.150 g, 0.54 mmol, 1.00 eq.), 1-(3-chloropropyl)piperidin-1-ium chloride (0.130 g, 0.65 mmol, 1.20 eq.) and K_2CO_3 (0.150 g, 1.11 mmol, 2.00 eq.) are suspended in anhydrous DMF (5 mL, 0.10 M). The reaction mixture is stirred overnight at 100°C and it's later cooled to rt. The mixture is partitioned between DCM/H_2O and the organic phase is separated. The water phase was extracted with DCM 3 times and the combined organic phases were washed with H_2O and brine. The organic phase was dried over Na_2SO_4 and the solvent was removed under reduced pressure. The residue was purified by flash column chromatography on silica gel (DCM:MeOH, 97:3) to afford the trifluoromethylated products **6** (0.146 g, 0.36 mmol, 67%) as a yellow oil. **^{1}H NMR** (400 MHz, $CDCl_3$) δ 8.17 (dt, J = 7.2, 1.1 Hz, 1H), 7.43 (dt, J = 9.0, 1.3 Hz, 1H), 7.37 (d, J = 8.5 Hz, 2H), 6.99 – 6.82 (m, 3H), 6.69 (td, J = 6.9, 1.4 Hz, 1H), 6.49 – 6.38 (m, 1H), 4.08 (t, J = 6.1 Hz, 2H), 2.86 – 2.54 (m, 6H), 2.25 – 2.12 (m, 2H), 1.78 (p, J = 5.7 Hz, 4H), 1.53 (p, J = 5.4 Hz, 2H). **^{13}C{^{1}H} NMR** (100 MHz, $CDCl_3$) δ 158.3, 134.7, 132.5, 130.8, 127.2, 124.6, 120.0, 118.9, 114.0, 112.0, 101.9, 66.0, 55.8, 54.3, 25.8, 24.8, 23.6. **^{19}F{^{1}H} NMR** (377 MHz, $CDCl_3$) δ -57.48 (s, 3F). **ESI-HRMS**: m/z calcd. for $C_{23}H_{26}F_3N_2O$ $[M+H]^+$ 403.1997, found 403.1992.

7. Absorption, emission and electrochemical properties

The absorption and emission spectra of the novel ruthenium-based complexes were investigated and compared to the homoleptic reference complexes in Ar-saturated DMF. The absorption spectra of all four complexes exhibit absorbance up to the green range, which enables the use of 532 nm and 515 nm as excitation wavelengths for spectroscopic investigations (see **Figure S5**). To investigate the triplet state lifetime, laser flash photolysis was used to measure time-resolved absorption traces of each complex at the concentration of 40 µM in Ar-saturated DMF (see **Figure S5**, insets).

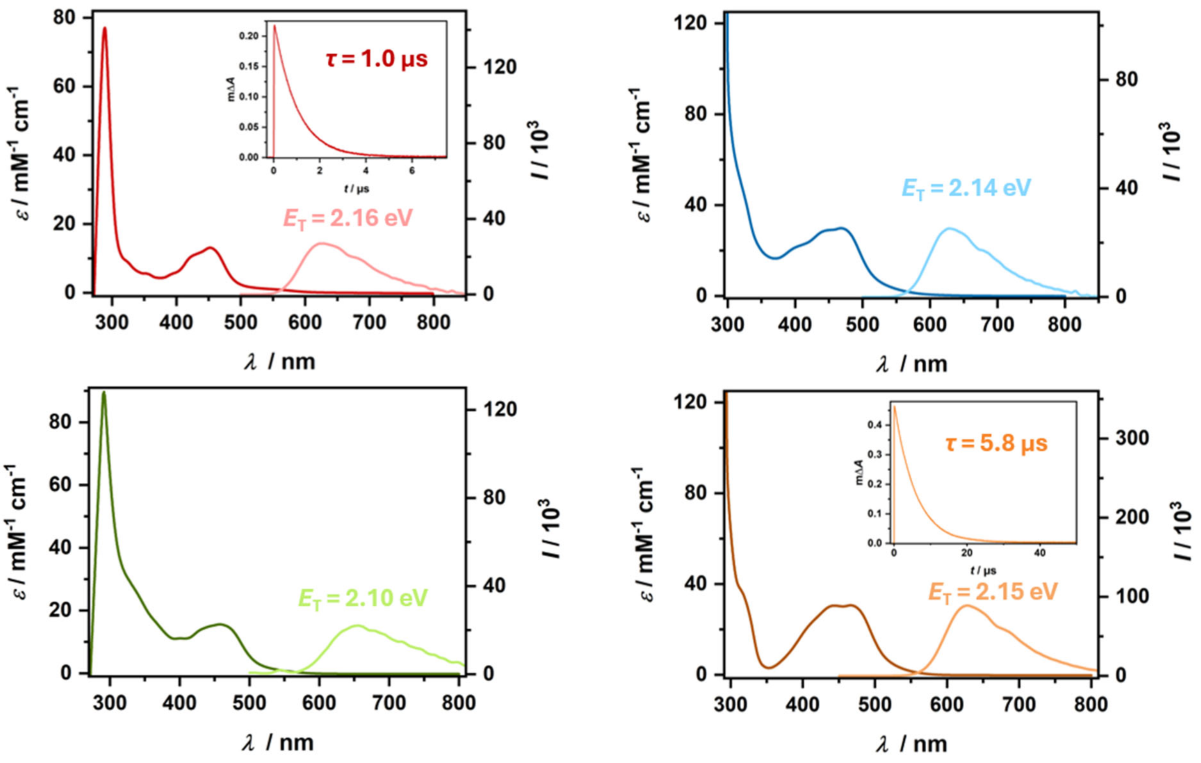

Figure S5 UV-vis absorption spectra (bold) and fluorescence spectra (light) of **[Ru(bpy)$_3$]$^{2+}$** (red), **Ru-I** (green), **Ru-II** (blue) and **Ru-III** (orange) in Ar-saturated DMF (40 µM catalyst) with associated time-resolved absorption traces at the detection wavelength 370 nm (**[Ru(bpy)$_3$]$^{2+}$** and **Ru-III**) in Ar-saturated DMF after laser excitation (λ_{exc} = 532 nm). **Ru-I** and **Ru-II** were investigated with the fs-TAS technique owing to their short lifetimes, see below. Steady-state emission spectra were recorded with 490 nm excitation.

The lifetime of excited **[Ru(bpy)$_3$]$^{2+}$** and **Ru-III** was directly obtained from a monoexponentional fit function, while for **Ru-I** and **Ru-II** the dominant initial decay is too short-lived for a reliable analysis with a nanosecond LFP setup (see section 9 for a detailed analysis). The triplet energies of the complexes were determined from the high-energy edge of the room-temperature phosphorescence spectrum, where the emission intensity amounts to 20% compared to the intensity at the emission maximum. That

procedure gave a triplet energy of 2.16 eV for **[Ru(bpy)$_3$]$^{2+}$**, which is very close to the widely accepted literature value (2.12 eV).

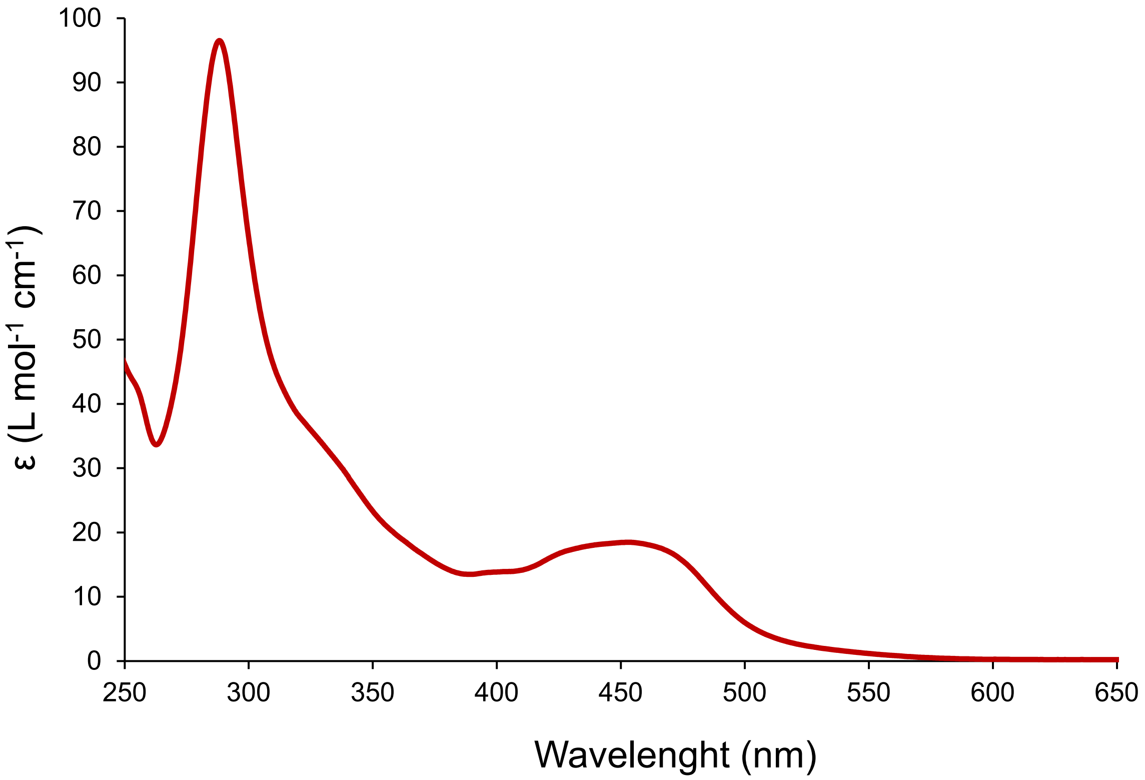

Figure S6. UV-vis spectrum of **Ru-I** (5 μM) in MeCN.

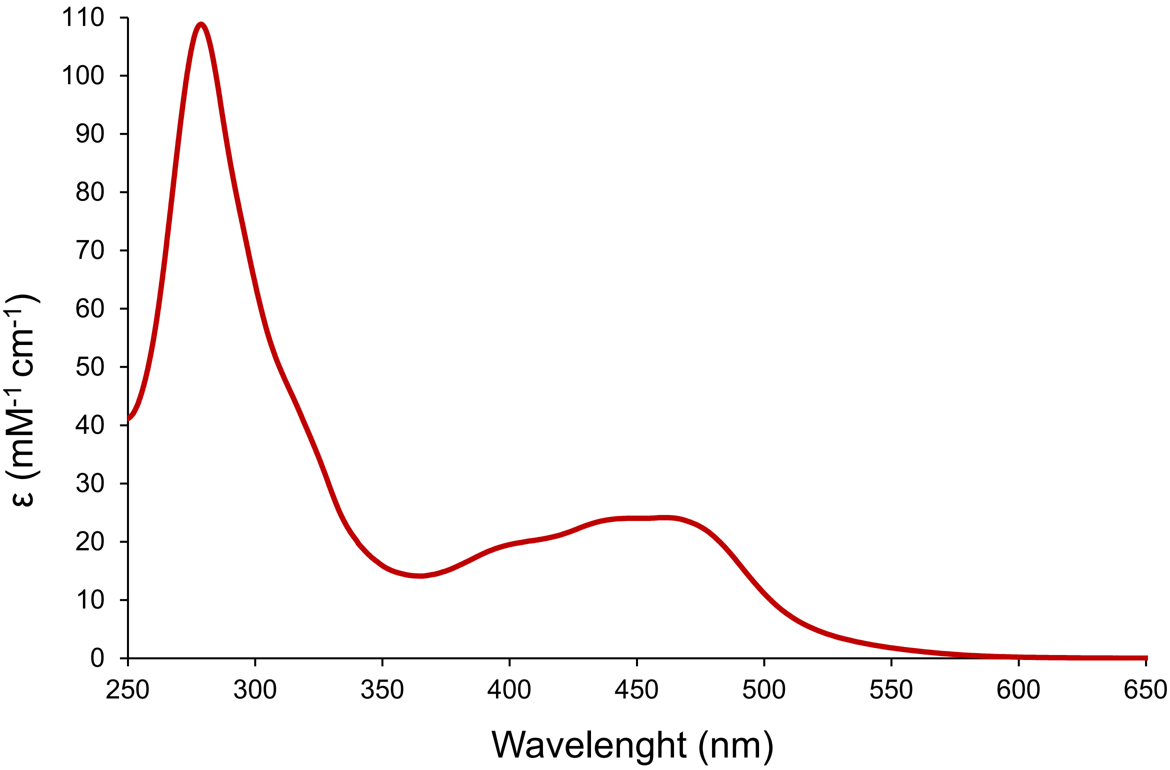

Figure S7. UV-vis spectrum of **Ru-II** (5 µM) in MeCN.

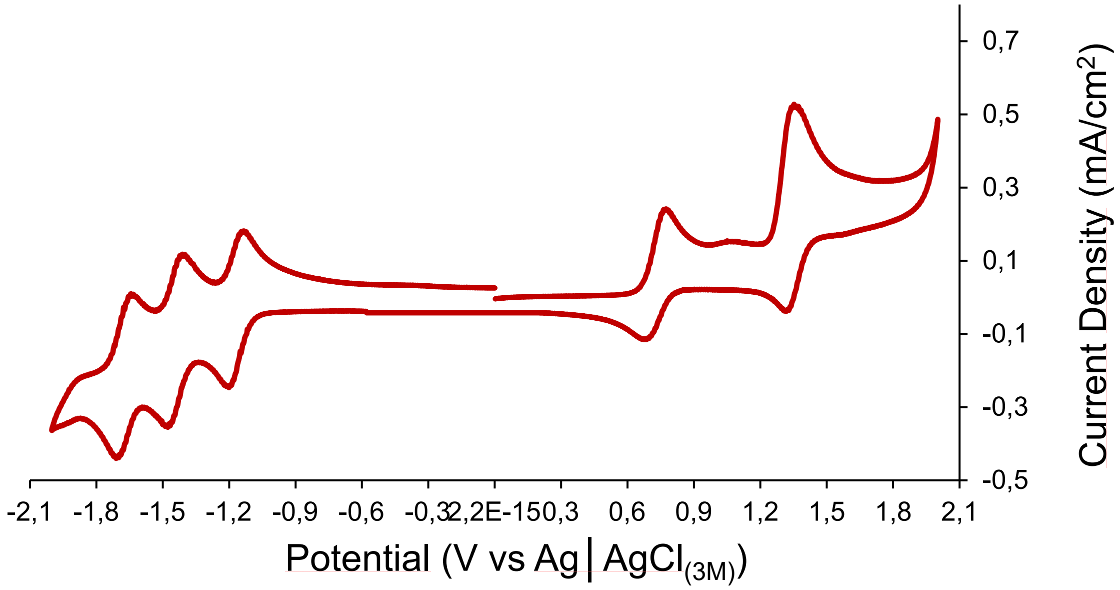

Figure S8. Cyclic voltammogram of **Ru-I** (1 mM) in a supporting electrolyte 0.1 M $[Bu_4N][PF_6]$ in MeCN referenced to Ag/Ag^+ couple at 0.1 Vs^{-1} sweep rate. The cyclic voltammetry experiments were performed in a cell with a three-electrode configuration. The working electrode (d=2 mm) was glassy carbon. A platinum sheet was used as counter electrode, while the reference electrode was Ag/AgCl (3.0 M KCl). The measurements were performed at a temperature of 22 °C under a nitrogen atmosphere after bubbling the solutions with the same gas for 10 min. The IUPAC plotting convention was used to plot voltammograms. The initial potential was set to 0.0 V, and the scan proceeded in the oxidation direction up to the potential shown in the plot.

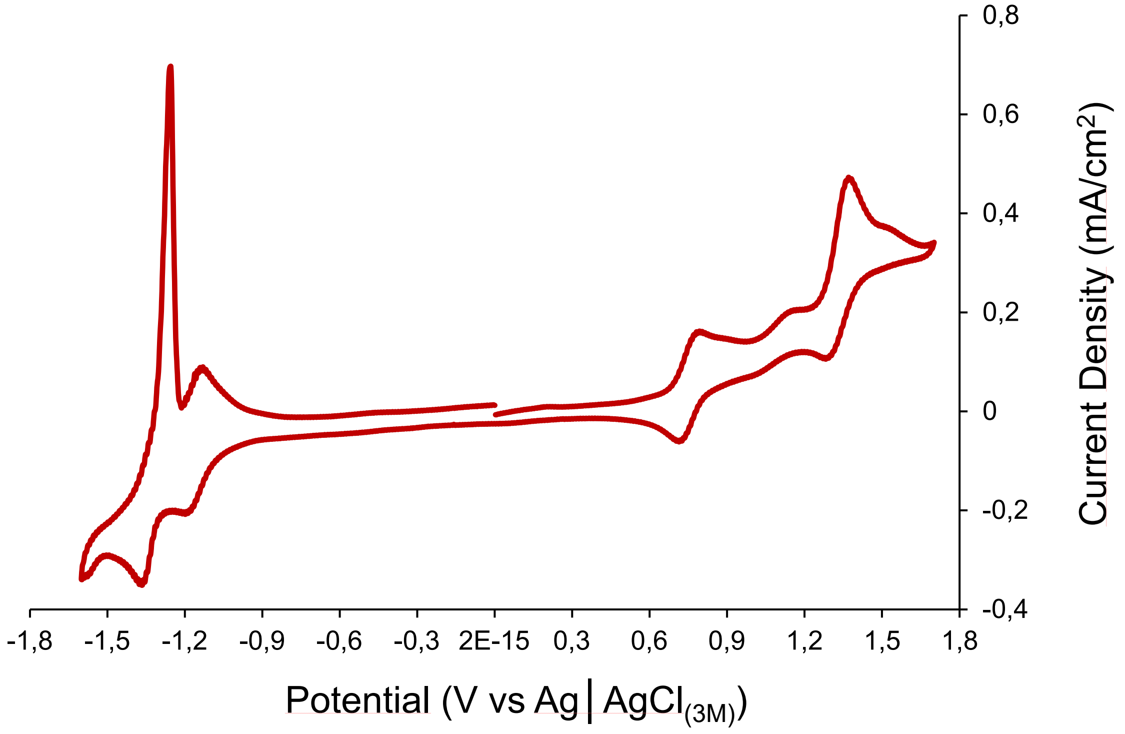

Figure S9. Cyclic voltammogram of **Ru-II** (1 mM) in a supporting electrolyte 0.1 M $[Bu_4N][PF_6]$ in MeCN referenced to Ag/Ag^+ couple at 0.1 Vs^{-1} sweep rate. For further conditions see caption of Fig. S8.

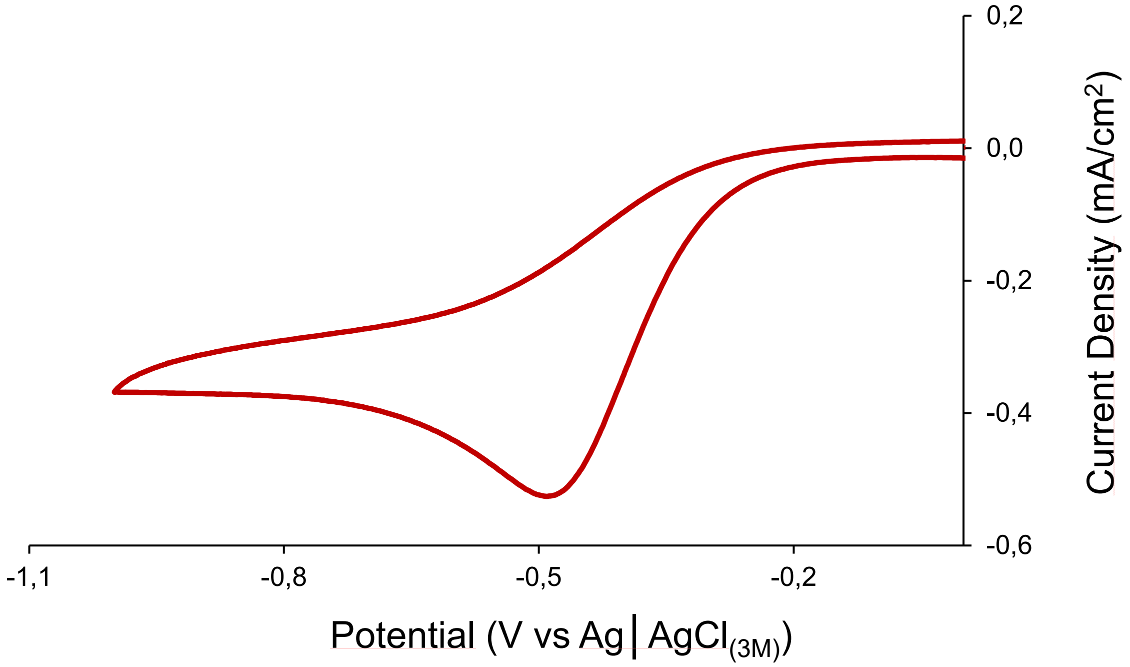

Figure S10. Cyclic voltammogram of **$TTCF_3OTf$** (2 mM) in a supporting electrolyte 0.1 M $[Bu_4N][PF_6]$ in MeCN referenced to Ag/Ag^+ couple at 0.1 Vs^{-1} sweep rate. For further conditions see caption of Fig. S8.

8. DFT Calculations

Each state of the complexes was initially optimized with XTB.[12,13] After that, we used the Conformer-Rotamer Ensemble Sampling Tool (CREST), which was developed by Grimme et al.[13,14] Afterwards, the conformer set was further processed using CENSO, developed by the same group,[15,16] to predict the most important conformer according to the Boltzmann distribution. In all calculations, DMF was incorporated as a solvent. The conformer with the highest Boltzmann weight was further calculated with high-level DFT with ORCA.[17,18] The structure was optimized using the functional PBE0 with a dispersion correction[19,20] and the def2-TZVP basis set[21] with a PCM solvent model for DMF. For all states of the complexes the analytical frequency calculations resulted in positive values. Energy levels vs. SHE were calculated the following: Energy vs SHE = –4.5 – (calculated orbital energy level)

Ru-II$^{(2+)}$ in DMF

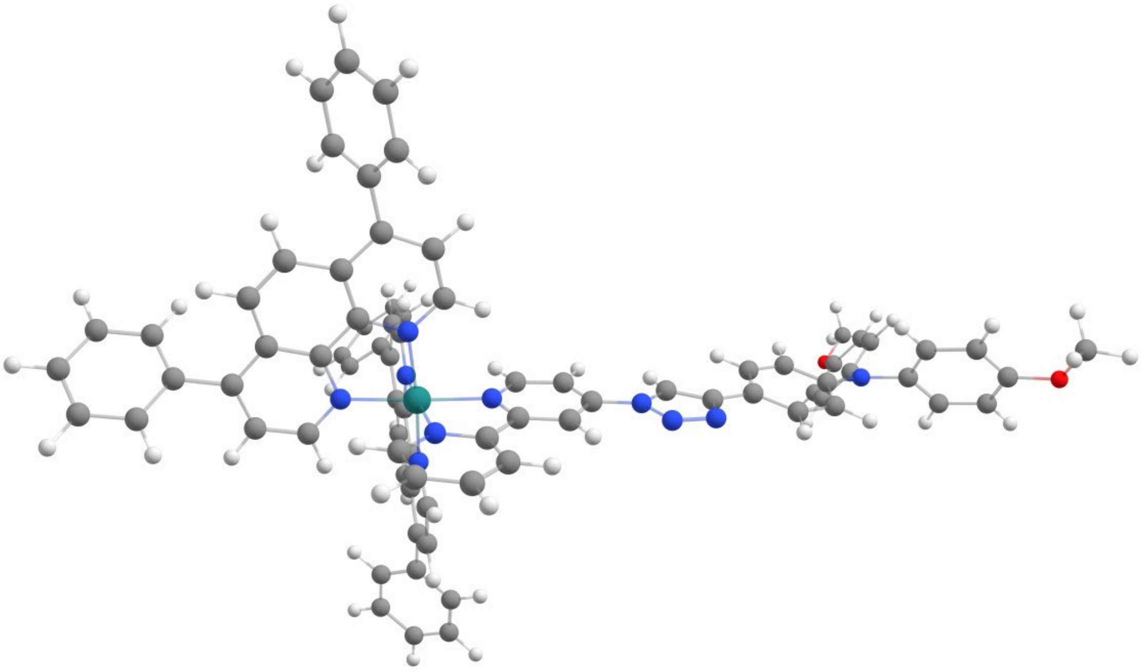

Free Energy = -2429918.3088 kcal/mol

COORDINATES:

44	3.093962000	0.294118000	-0.803033000	6	3.435029000	-2.858302000	2.875087000
7	1.059205000	0.507036000	-0.826208000	6	3.477255000	-1.503301000	3.137486000
7	2.869748000	-1.546561000	-1.679684000	6	3.358745000	-0.574046000	2.110564000
7	5.145475000	0.283806000	-0.829736000	7	3.202650000	-0.926960000	0.844315000
6	5.713586000	1.328961000	-0.179262000	6	4.816983000	2.286306000	0.373684000
6	7.100945000	1.497775000	-0.058682000	7	3.495045000	2.035017000	0.204974000
6	7.940613000	0.502147000	-0.615932000	6	2.630563000	2.917724000	0.680078000
6	7.331198000	-0.538715000	-1.288309000	6	3.027171000	4.060732000	1.364354000
6	5.946366000	-0.617566000	-1.375928000	6	4.364296000	4.328134000	1.582106000
6	2.655237000	-1.817078000	-2.957452000	6	5.306420000	3.417652000	1.043215000
6	2.529178000	-3.115613000	-3.436540000	6	0.212535000	0.014984000	0.080419000
6	2.627929000	-4.197858000	-2.584787000	6	-1.153246000	0.170335000	-0.000955000
6	2.819031000	-3.927519000	-1.207509000	6	-1.673006000	0.868178000	-1.082016000
6	2.938432000	-2.586682000	-0.812336000	6	-0.806844000	1.382444000	-2.033090000
6	3.133118000	-2.250345000	0.557040000	6	0.552322000	1.185443000	-1.878331000
6	3.222884000	-3.255071000	1.531854000	6	1.548190000	1.690388000	-2.824410000

6	1.233937000	2.425324000	-3.957536000	1	7.925601000	-1.325906000	-1.733964000
6	2.249782000	2.858290000	-4.788785000	1	5.474709000	-1.440879000	-1.896426000
6	3.558969000	2.544632000	-4.463184000	1	2.598988000	-0.974603000	-3.634265000
6	3.804163000	1.807412000	-3.321903000	1	2.381169000	-3.265416000	-4.498288000
7	2.826699000	1.382700000	-2.517242000	1	3.633873000	-1.144792000	4.146774000
7	-3.052417000	1.056278000	-1.223483000	1	3.403390000	0.486449000	2.321501000
7	-3.536539000	1.787988000	-2.236937000	1	1.580236000	2.702955000	0.533340000
7	-4.816118000	1.797749000	-2.135507000	1	2.265272000	4.724665000	1.752095000
6	-5.202659000	1.067686000	-1.046727000	1	0.649761000	-0.522759000	0.910382000
6	-4.062318000	0.585726000	-0.455224000	1	-1.777206000	-0.249538000	0.774844000
6	-6.597605000	0.894497000	-0.674803000	1	-1.199088000	1.924975000	-2.879105000
6	-6.956504000	0.325260000	0.547880000	1	0.204498000	2.658591000	-4.190636000
6	-8.279899000	0.157924000	0.900177000	1	2.020331000	3.433103000	-5.677251000
6	-9.307488000	0.551219000	0.033303000	1	4.389032000	2.862340000	-5.080342000
6	-8.949145000	1.122522000	-1.193923000	1	4.812364000	1.538682000	-3.037830000
6	-7.622734000	1.291364000	-1.534656000	1	-3.905564000	-0.039468000	0.405803000
7	-10.640760000	0.379941000	0.380650000	1	-6.188556000	0.022496000	1.251355000
6	-11.644786000	1.218612000	-0.155546000	1	-8.526137000	-0.273902000	1.861850000
6	-11.033572000	-0.630091000	1.287410000	1	-9.721636000	1.429968000	-1.887300000
6	-10.583313000	-1.941761000	1.136586000	1	-7.373881000	1.725810000	-2.495456000
6	-10.983037000	-2.927503000	2.014856000	1	-9.912037000	-2.186863000	0.321768000
6	-11.863357000	-2.631606000	3.056366000	1	-10.633247000	-3.946730000	1.897334000
6	-12.328710000	-1.329190000	3.205836000	1	-13.011823000	-1.068632000	4.002964000
6	-11.904186000	-0.340225000	2.328608000	1	-12.266281000	0.673738000	2.453697000
6	-12.804578000	0.665958000	-0.697112000	1	-12.925002000	-0.411102000	-0.714980000
6	-13.800284000	1.476739000	-1.202465000	1	-14.702532000	1.047401000	-1.622694000
6	-13.652805000	2.863847000	-1.194270000	1	-12.357039000	4.496146000	-0.631256000
6	-12.496300000	3.423837000	-0.659671000	1	-10.617735000	3.042967000	0.291517000
6	-11.509966000	2.599779000	-0.135548000	1	7.112182000	4.508403000	1.531822000
6	6.719536000	3.603518000	1.088057000	1	8.638300000	2.874441000	0.597071000
6	7.573047000	2.689672000	0.565494000	1	2.676102000	-5.957365000	-0.466540000
6	2.841285000	-4.925074000	-0.188896000	1	3.025643000	-5.385629000	1.862528000
6	3.036550000	-4.605419000	1.113504000	1	-12.693881000	-2.665390000	5.616775000
8	-12.209386000	-3.663547000	3.859519000	1	-14.068498000	-3.032584000	4.539865000
6	-13.109003000	-3.397986000	4.918190000	1	-13.259247000	-4.344787000	5.432994000
8	-14.674256000	3.577581000	-1.720271000	1	-15.468329000	5.365715000	-2.183105000
6	-14.557107000	4.987174000	-1.724361000	1	-14.470873000	5.380121000	-0.706944000
6	2.530561000	-5.565336000	-3.121651000	1	-13.692215000	5.310869000	-2.311280000
6	3.502594000	-6.523686000	-2.834212000	1	4.343109000	-6.264893000	-2.200593000
6	3.415793000	-7.794486000	-3.380095000	1	4.183400000	-8.526822000	-3.158257000
6	2.353740000	-8.126775000	-4.210303000	1	2.284275000	-9.123123000	-4.631337000
6	1.383252000	-7.178061000	-4.502215000	1	0.550671000	-7.431647000	-5.148171000
6	1.474390000	-5.902704000	-3.967771000	1	0.712497000	-5.164519000	-4.192012000
6	3.613958000	-3.826311000	3.969435000	1	5.144833000	-4.957037000	2.968408000
6	4.546935000	-4.858608000	3.867172000	1	5.472347000	-6.529249000	4.833559000
6	4.736551000	-5.738256000	4.920933000	1	4.136926000	-6.296335000	6.906012000
6	3.991563000	-5.604114000	6.084748000	1	2.476966000	-4.468858000	7.100353000
6	3.062670000	-4.578256000	6.194953000	1	2.149938000	-2.892342000	5.232616000
6	2.879491000	-3.689875000	5.147116000	1	6.046021000	4.454977000	3.704973000
6	4.759905000	5.519945000	2.350435000	1	6.647914000	6.453943000	5.009150000
6	5.639728000	5.421112000	3.428600000	1	5.703312000	8.662629000	4.407790000
6	5.971845000	6.545741000	4.167001000	1	4.140159000	8.852197000	2.496101000
6	5.437810000	7.782949000	3.832935000	1	3.535669000	6.849704000	1.190795000
6	4.559678000	7.889088000	2.762927000	1	9.420304000	0.834798000	1.631273000
6	4.216602000	6.763890000	2.030186000	1	11.875105000	0.836745000	1.809533000
6	9.407350000	0.542068000	-0.498817000	1	13.269686000	0.537310000	-0.214643000
6	10.025285000	0.717722000	0.739625000	1	12.185802000	0.225679000	-2.419707000
6	11.407278000	0.710378000	0.840075000	1	9.726651000	0.222208000	-2.598212000
6	12.188729000	0.537139000	-0.294290000				
6	11.581431000	0.359858000	-1.530055000				
6	10.199372000	0.354920000	-1.631499000				

LOEWDIN ATOMIC CHARGES

0 Ru: 0.130746	59 C : -0.141096	118 H : 0.157521	
1 N : 0.217439	60 C : -0.110957	119 H : 0.160610	
2 N : 0.218121	61 C : -0.076304	120 H : 0.162497	
3 N : 0.217362	62 C : -0.076439	121 H : 0.170456	
4 C : -0.105335	63 C : -0.076250	122 H : 0.170498	
5 C : -0.048779	64 C : -0.076436	123 H : 0.170456	
6 C : -0.035248	65 O : 0.234336	124 H : 0.170514	
7 C : -0.076943	66 C : -0.330483	125 H : 0.115784	
8 C : -0.145722	67 O : 0.234649	126 H : 0.115836	
9 C : -0.144872	68 C : -0.330437	127 H : 0.120777	
10 C : -0.076733	69 C : -0.111017	128 H : 0.120856	
11 C : -0.035263	70 C : -0.100633	129 H : 0.115824	
12 C : -0.048878	71 C : -0.138320	130 H : 0.115795	
13 C : -0.105192	72 C : -0.135898	131 H : 0.163452	
14 C : -0.104844	73 C : -0.137687	132 H : 0.156394	
15 C : -0.048849	74 C : -0.096466	133 H : 0.155472	
16 C : -0.035283	75 C : -0.111102	134 H : 0.156305	
17 C : -0.076821	76 C : -0.100304	135 H : 0.162218	
18 C : -0.145590	77 C : -0.138259	136 H : 0.163611	
19 N : 0.217719	78 C : -0.135672	137 H : 0.156453	
20 C : -0.105201	79 C : -0.137656	138 H : 0.155499	
21 N : 0.218034	80 C : -0.096363	139 H : 0.156306	
22 C : -0.144980	81 C : -0.111028	140 H : 0.162124	
23 C : -0.076856	82 C : -0.100563	141 H : 0.163519	
24 C : -0.035394	83 C : -0.138366	142 H : 0.156421	
25 C : -0.048922	84 C : -0.135825	143 H : 0.155503	
26 C : -0.140291	85 C : -0.137731	144 H : 0.156295	
27 C : -0.077075	86 C : -0.096386	145 H : 0.162198	
28 C : -0.140078	87 C : -0.110920	146 H : 0.163629	
29 C : -0.036538	88 C : -0.100366	147 H : 0.156461	
30 C : -0.142998	89 C : -0.138326	148 H : 0.155470	
31 C : -0.147227	90 C : -0.135661	149 H : 0.156298	
32 C : -0.068411	91 C : -0.137673	150 H : 0.162192	
33 C : -0.089138	92 C : -0.096444		
34 C : -0.110421	93 H : 0.172890		
35 C : -0.147637	94 H : 0.166781		
36 N : 0.219758	95 H : 0.166254		
37 N : 0.236222	96 H : 0.172921		
38 N : -0.018870	97 H : 0.172830		
39 N : 0.030825	98 H : 0.166646		
40 C : -0.237483	99 H : 0.166124		
41 C : -0.139080	100 H : 0.172917		
42 C : -0.149075	101 H : 0.169233		
43 C : -0.101256	102 H : 0.174177		
44 C : -0.118403	103 H : 0.172185		
45 C : -0.179664	104 H : 0.170391		
46 C : -0.118602	105 H : 0.166566		
47 C : -0.103746	106 H : 0.167399		
48 N : 0.294294	107 H : 0.165505		
49 C : -0.198357	108 H : 0.162561		
50 C : -0.198162	109 H : 0.160586		
51 C : -0.111676	110 H : 0.162400		
52 C : -0.124024	111 H : 0.162158		
53 C : -0.271209	112 H : 0.157179		
54 C : -0.141198	113 H : 0.162636		
55 C : -0.109430	114 H : 0.157512		
56 C : -0.108850	115 H : 0.160623		
57 C : -0.123665	116 H : 0.162338		
58 C : -0.270686	117 H : 0.162459		

Ru-II$^{(2+)}$ in DMF-triplet

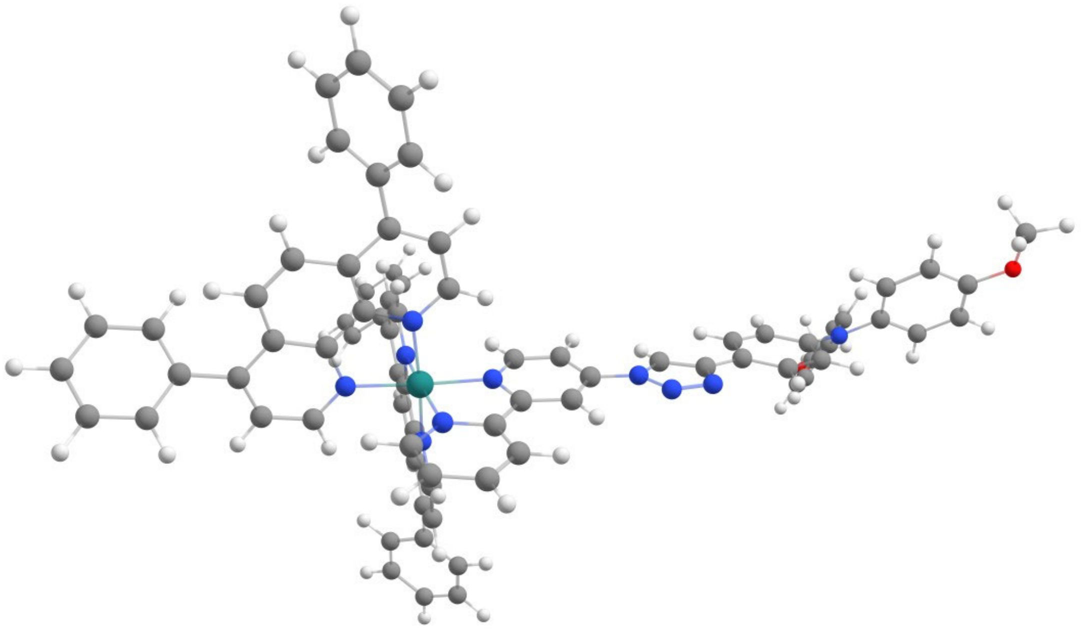

Free Energy = -2429881.6229 kcal/mol

COORDINATES:

44	3.150157000	0.204711000	-0.801697000	6	0.627955000	1.062938000	-1.960087000
7	1.113396000	0.415747000	-0.835777000	6	1.601491000	1.477905000	-2.906463000
7	2.925891000	-1.669305000	-1.583953000	6	1.314970000	2.147901000	-4.114637000
7	5.201730000	0.242670000	-0.806852000	6	2.328477000	2.507591000	-4.960958000
6	5.737961000	1.362336000	-0.260243000	6	3.656165000	2.204960000	-4.614238000
6	7.119589000	1.589523000	-0.164035000	6	3.881433000	1.547183000	-3.426976000
6	7.991655000	0.576169000	-0.630676000	7	2.905525000	1.181004000	-2.589901000
6	7.415403000	-0.547354000	-1.191953000	7	-2.999345000	1.061233000	-1.273962000
6	6.033922000	-0.680982000	-1.263833000	7	-3.491544000	1.847496000	-2.245183000
6	2.763721000	-2.003518000	-2.855422000	7	-4.775121000	1.858856000	-2.133293000
6	2.591727000	-3.319424000	-3.267845000	6	-5.147929000	1.082218000	-1.082673000
6	2.582187000	-4.355698000	-2.353656000	6	-3.999514000	0.564680000	-0.522496000
6	2.717993000	-4.015901000	-0.986009000	6	-6.534573000	0.894671000	-0.700801000
6	2.895896000	-2.662476000	-0.659982000	6	-6.873612000	0.135017000	0.424854000
6	3.057675000	-2.259420000	0.695204000	6	-8.189636000	-0.047014000	0.784055000
6	3.046179000	-3.208298000	1.729403000	6	-9.204663000	0.538023000	0.022494000
6	3.238150000	-2.746766000	3.054204000	6	-8.879759000	1.301828000	-1.102020000
6	3.382541000	-1.384611000	3.236610000	6	-7.560976000	1.472208000	-1.456701000
6	3.363663000	-0.513314000	2.154284000	7	-10.544611000	0.359422000	0.384960000
7	3.208558000	-0.927948000	0.905850000	6	-11.442624000	1.417774000	0.266464000
6	4.810360000	2.330757000	0.216891000	6	-10.976274000	-0.876006000	0.862647000
7	3.498225000	2.012356000	0.086766000	6	-10.454465000	-2.062026000	0.324742000
6	2.602872000	2.896647000	0.499907000	6	-10.887861000	-3.275137000	0.791005000
6	2.960157000	4.108804000	1.078367000	6	-11.848210000	-3.340854000	1.811503000
6	4.287904000	4.449352000	1.251993000	6	-12.365587000	-2.160585000	2.355602000
6	5.260365000	3.533774000	0.782673000	6	-11.931807000	-0.942592000	1.880660000
6	0.242862000	-0.007476000	0.083307000	6	-12.763873000	1.181652000	-0.142354000
6	-1.115987000	0.168178000	0.003166000	6	-13.641816000	2.226739000	-0.261211000
6	-1.616427000	0.843302000	-1.137691000	6	-13.230014000	3.535626000	0.029865000
6	-0.751687000	1.277796000	-2.104456000	6	-11.913831000	3.774912000	0.440175000

6	-11.032152000	2.723095000	0.552625000
6	6.666282000	3.774507000	0.796284000
6	7.550022000	2.849617000	0.348278000
6	2.630183000	-4.950267000	0.088552000
6	2.786945000	-4.566718000	1.379033000
8	-12.206049000	-4.565093000	2.199021000
6	-13.193589000	-4.696712000	3.213484000
8	-14.155021000	4.484474000	-0.113735000
6	-13.793029000	5.834620000	0.146618000
6	2.434165000	-5.744978000	-2.819324000
6	3.327574000	-6.739464000	-2.420028000
6	3.196054000	-8.033609000	-2.898246000
6	2.167213000	-8.353427000	-3.773958000
6	1.275461000	-7.369270000	-4.178371000
6	1.411478000	-6.072093000	-3.709792000
6	3.288329000	-3.652213000	4.214724000
6	4.135536000	-4.760752000	4.230087000
6	4.198040000	-5.580270000	5.345969000
6	3.410869000	-5.308810000	6.456967000
6	2.567148000	-4.206305000	6.451009000
6	2.510935000	-3.379361000	5.340280000
6	4.640151000	5.722090000	1.904308000
6	5.526847000	5.760060000	2.980897000
6	5.816005000	6.960880000	3.609544000
6	5.230946000	8.139581000	3.166890000
6	4.345760000	8.110316000	2.097787000
6	4.046830000	6.909046000	1.474503000
6	9.457440000	0.679052000	-0.533108000
6	10.078781000	0.986788000	0.677698000
6	11.460999000	1.038025000	0.763746000
6	12.240143000	0.791361000	-0.358622000
6	11.630007000	0.482740000	-1.566864000
6	10.248114000	0.420203000	-1.652678000
1	8.036080000	-1.353485000	-1.561994000
1	5.586739000	-1.566092000	-1.698063000
1	2.786241000	-1.195500000	-3.575046000
1	2.490018000	-3.523418000	-4.326171000
1	3.536318000	-0.978372000	4.228224000
1	3.482882000	0.552337000	2.301951000
1	1.562590000	2.623253000	0.380854000
1	2.176508000	4.774280000	1.417642000
1	0.666387000	-0.511368000	0.943602000
1	-1.750541000	-0.190571000	0.799139000
1	-1.135283000	1.783647000	-2.978204000
1	0.288479000	2.375867000	-4.369825000
1	2.105418000	3.021361000	-5.888755000
1	4.489105000	2.472466000	-5.250227000
1	4.887906000	1.288401000	-3.121742000
1	-3.836841000	-0.099520000	0.307398000
1	-6.096636000	-0.305563000	1.037653000
1	-8.440283000	-0.615693000	1.670709000
1	-9.668898000	1.730971000	-1.706633000
1	-7.312268000	2.047078000	-2.339312000
1	-9.732558000	-2.015291000	-0.480827000
1	-10.510621000	-4.198395000	0.368656000
1	-13.088927000	-2.188479000	3.158446000
1	-12.310333000	-0.028681000	2.321213000
1	-13.076777000	0.176674000	-0.396317000
1	-14.657633000	2.062176000	-0.598944000
1	-11.580705000	4.773171000	0.687523000
1	-10.021166000	2.904392000	0.895484000
1	7.028646000	4.728183000	1.156225000
1	8.608073000	3.074849000	0.355821000
1	2.412955000	-5.985561000	-0.137793000
1	2.692912000	-5.299850000	2.168860000
1	-12.852647000	-4.244020000	4.147513000
1	-14.134246000	-4.237775000	2.900264000
1	-13.334142000	-5.765594000	3.353299000
1	-14.686511000	6.423041000	-0.046373000
1	-13.486347000	5.960094000	1.187688000
1	-12.988223000	6.155578000	-0.518745000
1	4.143835000	-6.492206000	-1.751024000
1	3.903263000	-8.794258000	-2.588149000
1	2.062703000	-9.367259000	-4.142875000
1	0.468994000	-7.612241000	-4.860656000
1	0.709980000	-5.306316000	-4.021620000
1	4.766324000	-4.967589000	3.373188000
1	4.868324000	-6.432068000	5.348534000
1	3.457192000	-5.953881000	7.326812000
1	1.948783000	-3.989048000	7.314330000
1	1.846267000	-2.522676000	5.335466000
1	5.973127000	4.840906000	3.342965000
1	6.498490000	6.974448000	4.451510000
1	5.462207000	9.078662000	3.656317000
1	3.886185000	9.026799000	1.745950000
1	3.359708000	6.889499000	0.635912000
1	9.476799000	1.162124000	1.561855000
1	11.931092000	1.267373000	1.713103000
1	13.320983000	0.836910000	-0.290877000
1	12.232036000	0.290655000	-2.447526000
1	9.773511000	0.184790000	-2.598763000

LOEWDIN POPULATION ANALYSIS
LOEWDIN ATOMIC CHARGES AND SPIN POPULATIONS

0 Ru:	0.129002	0.029960
1 N :	0.171260	0.145884
2 N :	0.219074	0.003771
3 N :	0.216435	0.000735
4 C :	-0.107102	0.000596
5 C :	-0.052016	-0.000033
6 C :	-0.040559	0.000997
7 C :	-0.081814	0.000247
8 C :	-0.152057	0.001015
9 C :	-0.149135	0.000202
10 C :	-0.082339	0.001349
11 C :	-0.041499	0.000979
12 C :	-0.052509	0.000467
13 C :	-0.106378	0.001400
14 C :	-0.107277	0.000797
15 C :	-0.052165	0.000095
16 C :	-0.040681	0.001071
17 C :	-0.082238	0.000606
18 C :	-0.152001	0.000865
19 N :	0.217157	0.001082
20 C :	-0.106514	0.000854
21 N :	0.219389	0.003244
22 C :	-0.148775	-0.000093
23 C :	-0.082089	0.001146
24 C :	-0.041126	0.000272
25 C :	-0.052460	0.000502
26 C :	-0.196765	-0.002023
27 C :	-0.163280	0.148200
28 C :	-0.186323	0.072811
29 C :	-0.077960	0.016214
30 C :	-0.194001	0.129116
31 C :	-0.188483	0.082777
32 C :	-0.116351	0.053848
33 C :	-0.142096	0.027422
34 C :	-0.179770	0.113513
35 C :	-0.194177	-0.009238
36 N :	0.185249	0.107273
37 N :	0.240046	0.001296
38 N :	-0.027811	0.030701
39 N :	0.024582	0.017033
40 C :	-0.249507	0.009477
41 C :	-0.138705	0.046592
42 C :	-0.103678	0.067064
43 C :	-0.077911	-0.013036
44 C :	-0.082042	0.054339
45 C :	-0.161551	0.017350
46 C :	-0.081843	0.055873
47 C :	-0.079973	-0.015657
48 N :	0.381241	0.242269
49 C :	-0.168162	0.041689
50 C :	-0.168604	0.040365
51 C :	-0.082159	0.048810
52 C :	-0.095973	-0.003682
53 C :	-0.243477	0.075164
54 C :	-0.113627	-0.001850
55 C :	-0.077514	0.063827
56 C :	-0.082332	0.049743
57 C :	-0.096239	-0.005128
58 C :	-0.243216	0.075726
59 C :	-0.113043	-0.000168
60 C :	-0.076955	0.063347
61 C :	-0.079805	-0.000228
62 C :	-0.080068	0.000353
63 C :	-0.079864	-0.000154
64 C :	-0.080394	0.000177
65 O :	0.273246	0.044205
66 C :	-0.308069	0.002224
67 O :	0.273753	0.044493
68 C :	-0.307932	0.002223
69 C :	-0.111136	0.000066
70 C :	-0.101968	0.000144
71 C :	-0.139737	-0.000021
72 C :	-0.138121	0.000134
73 C :	-0.139201	-0.000018
74 C :	-0.097973	0.000092
75 C :	-0.111066	0.000056
76 C :	-0.101727	0.000139
77 C :	-0.139563	-0.000035
78 C :	-0.137837	0.000153
79 C :	-0.139109	-0.000032
80 C :	-0.098018	0.000130
81 C :	-0.111105	0.000023
82 C :	-0.101940	0.000044
83 C :	-0.139770	0.000003
84 C :	-0.138114	0.000030
85 C :	-0.139140	0.000005
86 C :	-0.097901	0.000004
87 C :	-0.111099	0.000050
88 C :	-0.101608	0.000132
89 C :	-0.139649	-0.000035
90 C :	-0.137832	0.000148
91 C :	-0.138996	-0.000032
92 C :	-0.097788	0.000129
93 H :	0.171410	-0.000006
94 H :	0.164768	-0.000009
95 H :	0.165588	0.000020
96 H :	0.171212	0.000044
97 H :	0.171360	-0.000005
98 H :	0.164852	-0.000009
99 H :	0.165768	0.000001
100 H :	0.171278	0.000048
101 H :	0.160881	-0.000222
102 H :	0.158091	0.000631
103 H :	0.163306	-0.000179
104 H :	0.159557	-0.000054
105 H :	0.155894	0.000039
106 H :	0.153726	0.000108
107 H :	0.158421	-0.000120
108 H :	0.161851	0.000169
109 H :	0.166702	0.000320
110 H :	0.171769	-0.000264
111 H :	0.171628	-0.000244
112 H :	0.163140	0.000294
113 H :	0.172765	-0.000277
114 H :	0.165684	0.000207
115 H :	0.168168	0.000268
116 H :	0.173320	-0.000246
117 H :	0.172891	-0.000202
118 H :	0.165775	0.000280
119 H :	0.168245	0.000206
120 H :	0.173142	-0.000306
121 H :	0.169216	-0.000002
122 H :	0.169346	0.000006
123 H :	0.169258	-0.000003
124 H :	0.169167	0.000005
125 H :	0.121363	0.001588
126 H :	0.121500	0.001408
127 H :	0.124704	-0.000083
128 H :	0.124725	-0.000087
129 H :	0.121435	0.001580
130 H :	0.121500	0.001429
131 H :	0.162983	0.000001
132 H :	0.155998	0.000001
133 H :	0.154996	0.000000
134 H :	0.155865	0.000001
135 H :	0.161764	-0.000003
136 H :	0.163104	-0.000001
137 H :	0.156057	0.000001
138 H :	0.155043	-0.000000
139 H :	0.155904	0.000001
140 H :	0.161705	-0.000001
141 H :	0.162950	0.000001
142 H :	0.155992	0.000000
143 H :	0.155017	0.000000
144 H :	0.155888	0.000000
145 H :	0.161781	-0.000001
146 H :	0.163109	-0.000001
147 H :	0.156051	0.000001
148 H :	0.155038	-0.000000
149 H :	0.155929	0.000001
150 H :	0.161773	-0.000002

SOMO: -3.63 eV

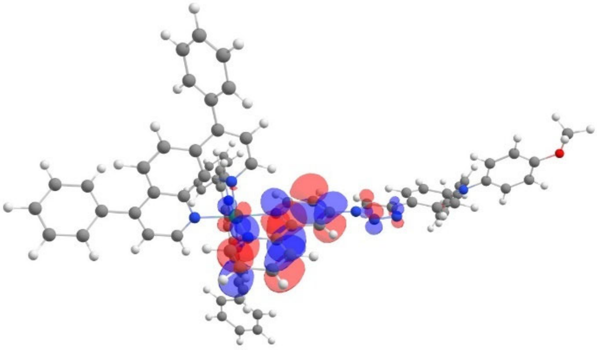

Second SOMO: -5.84 eV

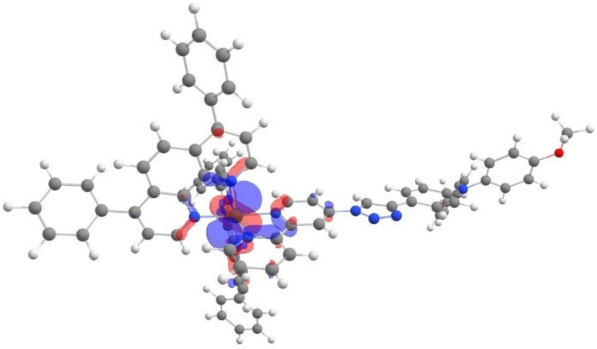

SUMO: -2.73 eV

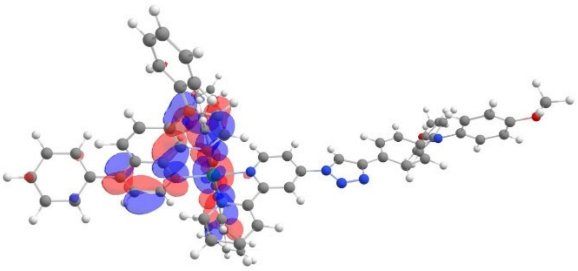

Second SUMO: -4.31 eV

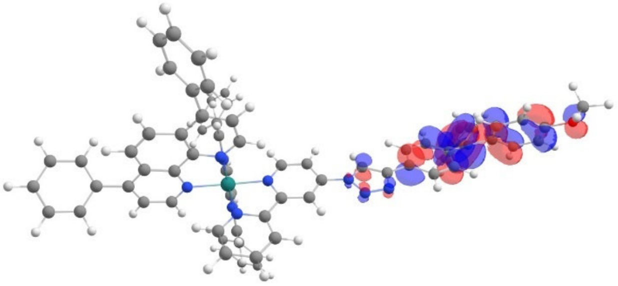

HOMO: -5.78 eV

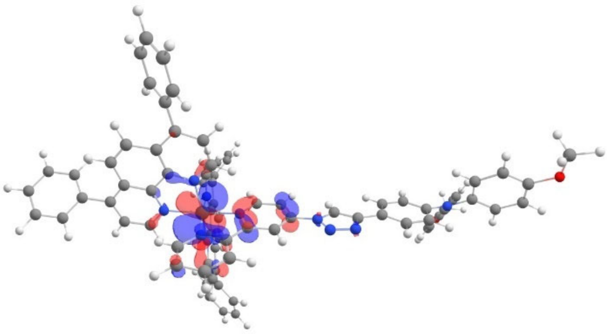

LUMO: -2.37 eV

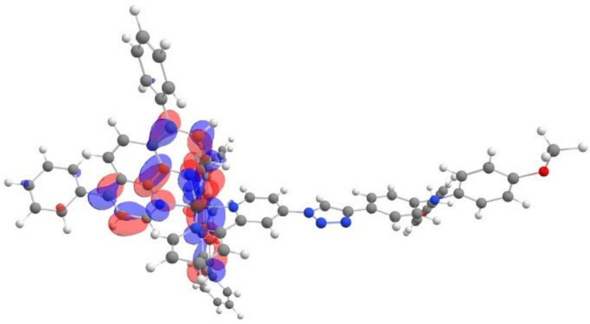

Ru-II$^{(3+)}$ in DMF

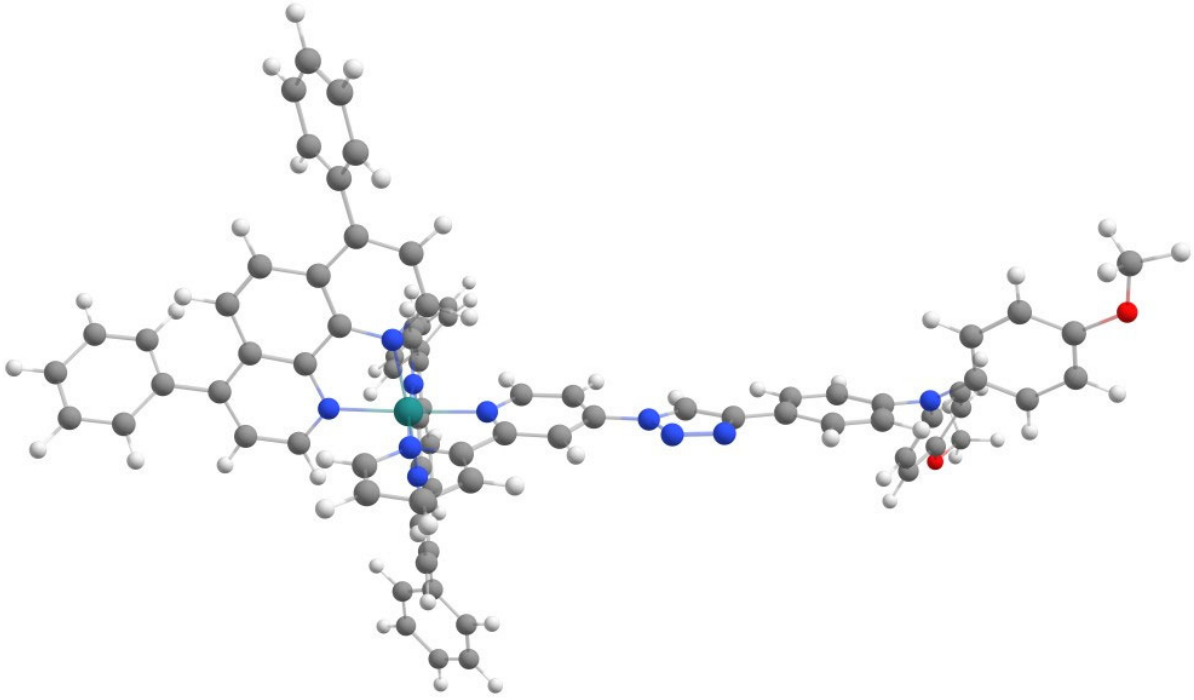

Free Energy = -2429805.6606 kcal/mol

COORDINATES:

44	3.080753000	0.256524000	-0.840418000	6	-1.683341000	0.826570000	-1.115514000
7	1.046515000	0.463899000	-0.867283000	6	-0.826993000	1.279313000	-2.104510000
7	2.854555000	-1.645075000	-1.578183000	6	0.533478000	1.081708000	-1.953076000
7	5.132582000	0.248906000	-0.876616000	6	1.522841000	1.520873000	-2.938006000
6	5.701383000	1.341092000	-0.309231000	6	1.201258000	2.185112000	-4.111894000
6	7.089007000	1.522322000	-0.210024000	6	2.211388000	2.558834000	-4.978295000
6	7.928685000	0.491654000	-0.699791000	6	3.521867000	2.257949000	-4.646055000
6	7.318262000	-0.598100000	-1.288885000	6	3.774472000	1.592371000	-3.462900000
6	5.933034000	-0.688152000	-1.359721000	7	2.802596000	1.225053000	-2.623922000
6	2.655934000	-2.012171000	-2.834324000	7	-3.065597000	1.023555000	-1.248830000
6	2.524472000	-3.342862000	-3.213768000	7	-3.550589000	1.795718000	-2.236316000
6	2.595334000	-4.356846000	-2.279011000	7	-4.826764000	1.810815000	-2.129487000
6	2.763416000	-3.981513000	-0.923257000	6	-5.210080000	1.044268000	-1.066838000
6	2.898116000	-2.615848000	-0.631946000	6	-4.071322000	0.534563000	-0.494890000
6	3.085517000	-2.177991000	0.709617000	6	-6.603395000	0.864464000	-0.696622000
6	3.138583000	-3.104317000	1.762058000	6	-6.957322000	0.074626000	0.401579000
6	3.340643000	-2.606752000	3.073170000	6	-8.278532000	-0.099791000	0.747223000
6	3.425637000	-1.236916000	3.226258000	6	-9.278534000	0.524339000	-0.001537000
6	3.345997000	-0.389732000	2.127392000	6	-8.938010000	1.319208000	-1.097521000
7	3.185946000	-0.838256000	0.892316000	6	-7.613755000	1.481909000	-1.439543000
6	4.805193000	2.332697000	0.180942000	7	-10.625288000	0.351324000	0.347254000
7	3.483033000	2.066171000	0.039990000	6	-11.500459000	1.431729000	0.282033000
6	2.618581000	2.976669000	0.459621000	6	-11.079553000	-0.900451000	0.752991000
6	3.015749000	4.164928000	1.061426000	6	-10.566650000	-2.064021000	0.159000000
6	4.353395000	4.450805000	1.250792000	6	-11.020695000	-3.293501000	0.556279000
6	5.295105000	3.509188000	0.767632000	6	-11.993035000	-3.399757000	1.562521000
6	0.206574000	0.025866000	0.073031000	6	-12.502213000	-2.242495000	2.161852000
6	-1.158980000	0.184049000	-0.003804000	6	-12.048149000	-1.007547000	1.755997000

6	-12.827077000	1.243454000	-0.135911000
6	-13.681774000	2.311448000	-0.204577000
6	-13.241271000	3.596204000	0.147063000
6	-11.920150000	3.787635000	0.567956000
6	-11.061515000	2.713393000	0.629389000
6	6.707764000	3.702044000	0.789838000
6	7.560894000	2.756630000	0.326026000
6	2.748398000	-4.896083000	0.170808000
6	2.927078000	-4.479093000	1.447882000
8	-12.368230000	-4.637078000	1.883140000
6	-13.363120000	-4.810111000	2.884557000
8	-14.144151000	4.570364000	0.047416000
6	-13.751901000	5.899737000	0.365556000
6	2.498548000	-5.761316000	-2.709433000
6	3.452593000	-6.700913000	-2.317667000
6	3.370837000	-8.009978000	-2.764782000
6	2.331624000	-8.398991000	-3.599229000
6	1.379227000	-7.469529000	-3.994760000
6	1.465513000	-6.156830000	-3.559081000
6	3.456956000	-3.484549000	4.249478000
6	4.349033000	-4.557177000	4.265021000
6	4.472969000	-5.348661000	5.395748000
6	3.702644000	-5.085293000	6.520374000
6	2.814384000	-4.018357000	6.513677000
6	2.697025000	-3.218206000	5.388434000
6	4.749472000	5.692369000	1.935985000
6	5.632817000	5.667435000	3.015728000
6	5.963046000	6.839234000	3.677682000
6	5.423906000	8.050123000	3.264611000
6	4.542715000	8.082935000	2.192326000
6	4.201295000	6.910965000	1.536202000
6	9.396339000	0.545366000	-0.597627000
6	10.022899000	0.807526000	0.621144000
6	11.405660000	0.811994000	0.711154000
6	12.179286000	0.564700000	-0.414849000
6	11.563391000	0.301710000	-1.630932000
6	10.180617000	0.284615000	-1.721141000
1	7.912439000	-1.414029000	-1.679877000
1	5.460627000	-1.550284000	-1.812282000
1	2.619275000	-1.224477000	-3.575335000
1	2.394939000	-3.573125000	-4.263464000
1	3.581478000	-0.803448000	4.205769000
1	3.421593000	0.682315000	2.253483000
1	1.567896000	2.749434000	0.335820000
1	2.254208000	4.851016000	1.409283000
1	0.649117000	-0.464426000	0.929023000
1	-1.777003000	-0.186002000	0.801557000
1	-1.225336000	1.774920000	-2.976312000
1	0.170775000	2.409550000	-4.349137000
1	1.976618000	3.078348000	-5.898857000
1	4.347660000	2.531397000	-5.289619000
1	4.784096000	1.336691000	-3.171253000
1	-3.914916000	-0.116011000	0.347652000
1	-6.190879000	-0.399132000	1.002770000
1	-8.543416000	-0.694428000	1.612415000
1	-9.717518000	1.780681000	-1.690667000
1	-7.351698000	2.083255000	-2.300320000
1	-9.835204000	-1.985287000	-0.635417000
1	-10.650328000	-4.198517000	0.090634000
1	-13.233668000	-2.302795000	2.955571000
1	-12.418928000	-0.113430000	2.241457000
1	-13.161410000	0.258807000	-0.437466000
1	-14.700658000	2.185495000	-0.549525000
1	-11.566407000	4.765910000	0.862129000
1	-10.047028000	2.856255000	0.979989000
1	7.100599000	4.636604000	1.167199000
1	8.625734000	2.946105000	0.338098000
1	2.566921000	-5.943604000	-0.028588000
1	2.886006000	-5.197989000	2.255020000
1	-13.023710000	-4.406857000	3.841434000
1	-14.297709000	-4.327951000	2.588723000
1	-13.513396000	-5.883304000	2.970423000
1	-14.632173000	6.515641000	0.199835000
1	-13.441632000	5.972777000	1.410437000
1	-12.940579000	6.230426000	-0.286922000
1	4.275999000	-6.398488000	-1.680957000
1	4.124552000	-8.727872000	-2.462672000
1	2.266002000	-9.424705000	-3.943378000
1	0.564584000	-7.767414000	-4.644700000
1	0.717553000	-5.433494000	-3.864055000
1	4.966515000	-4.756883000	3.396876000
1	5.177630000	-6.172168000	5.398912000
1	3.796925000	-5.709001000	7.401813000
1	2.209041000	-3.808103000	7.387822000
1	1.997948000	-2.389417000	5.383044000
1	6.042868000	4.722854000	3.354336000
1	6.641593000	6.804991000	4.522166000
1	5.687794000	8.966476000	3.779877000
1	4.119245000	9.025110000	1.863999000
1	3.517484000	6.939617000	0.695237000
1	9.424278000	0.982971000	1.507521000
1	11.880256000	1.005559000	1.666149000
1	13.260812000	0.574120000	-0.343847000
1	12.161599000	0.109569000	-2.514115000
1	9.701443000	0.084645000	-2.673025000

LOEWDIN ATOMIC CHARGES AND SPIN POPULATIONS

0 Ru:	0.131531	0.000783
1 N :	0.219284	0.000764
2 N :	0.217952	0.000013
3 N :	0.217416	0.000003
4 C :	-0.105156	0.000001
5 C :	-0.048590	-0.000000
6 C :	-0.034957	-0.000003
7 C :	-0.076646	0.000000
8 C :	-0.145248	-0.000000
9 C :	-0.144810	-0.000004
10 C :	-0.076643	0.000002
11 C :	-0.035069	-0.000004
12 C :	-0.048727	-0.000000
13 C :	-0.104835	-0.000004
14 C :	-0.105448	-0.000003
15 C :	-0.048553	-0.000000
16 C :	-0.034915	-0.000004
17 C :	-0.076836	0.000000
18 C :	-0.145425	-0.000004
19 N :	0.217322	-0.000003
20 C :	-0.105101	-0.000003
21 N :	0.217963	0.000013
22 C :	-0.145187	-0.000002
23 C :	-0.076801	0.000002
24 C :	-0.035077	-0.000004
25 C :	-0.048754	-0.000000
26 C :	-0.138149	-0.000266
27 C :	-0.074624	0.000832
28 C :	-0.138657	0.000093
29 C :	-0.034145	0.000494
30 C :	-0.140762	0.000046
31 C :	-0.146854	-0.000019
32 C :	-0.067890	-0.000001
33 C :	-0.088717	-0.000016
34 C :	-0.109595	0.000003
35 C :	-0.147085	-0.000008
36 N :	0.220055	-0.000028
37 N :	0.242842	0.002725
38 N :	-0.007714	0.002264
39 N :	0.042275	0.009516
40 C :	-0.232107	0.001403
41 C :	-0.119296	0.024867
42 C :	-0.104532	0.061705
43 C :	-0.074488	-0.016916
44 C :	-0.079514	0.052865
45 C :	-0.159507	0.011072
46 C :	-0.079199	0.054266
47 C :	-0.077166	-0.017433
48 N :	0.381883	0.242161
49 C :	-0.166892	0.044393
50 C :	-0.167431	0.043015
51 C :	-0.081363	0.049242
52 C :	-0.094655	-0.002404
53 C :	-0.242352	0.077627
54 C :	-0.112482	-0.001411
55 C :	-0.076402	0.065009
56 C :	-0.081437	0.050559
57 C :	-0.095060	-0.004633
58 C :	-0.242047	0.078266
59 C :	-0.111705	0.001061
60 C :	-0.075903	0.064238
61 C :	-0.076139	-0.000000
62 C :	-0.076172	0.000001
63 C :	-0.076161	0.000000
64 C :	-0.076318	0.000001
65 O :	0.275218	0.046422
66 C :	-0.306957	0.002328
67 O :	0.275826	0.046689
68 C :	-0.306918	0.002321
69 C :	-0.110983	0.000000
70 C :	-0.100380	-0.000000
71 C :	-0.138115	0.000000
72 C :	-0.135543	0.000000
73 C :	-0.137630	0.000000
74 C :	-0.096459	-0.000000
75 C :	-0.110899	-0.000000
76 C :	-0.100498	-0.000000
77 C :	-0.138108	0.000000
78 C :	-0.135541	-0.000000
79 C :	-0.137699	0.000000
80 C :	-0.096595	-0.000000
81 C :	-0.110968	-0.000000
82 C :	-0.100375	-0.000001
83 C :	-0.138239	0.000000
84 C :	-0.135695	-0.000000
85 C :	-0.137674	0.000000
86 C :	-0.096502	-0.000000
87 C :	-0.110960	-0.000000
88 C :	-0.100414	-0.000000
89 C :	-0.138229	0.000000
90 C :	-0.135581	-0.000000
91 C :	-0.137590	0.000000
92 C :	-0.096427	-0.000000
93 H :	0.172948	0.000000
94 H :	0.166670	-0.000000
95 H :	0.166288	0.000000
96 H :	0.172967	0.000000
97 H :	0.172962	-0.000000
98 H :	0.166646	-0.000000
99 H :	0.166052	-0.000000
100 H :	0.172943	0.000001
101 H :	0.169837	0.000000
102 H :	0.175023	-0.000000
103 H :	0.173119	-0.000009
104 H :	0.170508	-0.000000
105 H :	0.166705	0.000000
106 H :	0.167591	-0.000000
107 H :	0.165779	-0.000000
108 H :	0.166657	-0.000012
109 H :	0.167442	0.000314
110 H :	0.172334	-0.000277
111 H :	0.172202	-0.000257
112 H :	0.164118	0.000319
113 H :	0.172981	-0.000295
114 H :	0.166025	0.000192
115 H :	0.168574	0.000274
116 H :	0.173719	-0.000235
117 H :	0.173252	-0.000183
118 H :	0.166197	0.000289
119 H :	0.168518	0.000193
120 H :	0.173388	-0.000326
121 H :	0.170509	-0.000000
122 H :	0.170571	0.000000
123 H :	0.170555	-0.000000
124 H :	0.170462	0.000000
125 H :	0.121649	0.001668
126 H :	0.121806	0.001480
127 H :	0.124911	-0.000087
128 H :	0.124949	-0.000090
129 H :	0.121657	0.001654
130 H :	0.121772	0.001504
131 H :	0.163590	-0.000000
132 H :	0.156511	-0.000000
133 H :	0.155559	-0.000000
134 H :	0.156339	0.000000
135 H :	0.162144	0.000000
136 H :	0.163569	0.000000
137 H :	0.156509	-0.000000
138 H :	0.155548	0.000000
139 H :	0.156331	-0.000000
140 H :	0.162149	0.000000
141 H :	0.163508	0.000000
142 H :	0.156480	-0.000000
143 H :	0.155551	0.000000
144 H :	0.156319	-0.000000
145 H :	0.162171	0.000000
146 H :	0.163567	-0.000000
147 H :	0.156497	-0.000000
148 H :	0.155528	0.000000
149 H :	0.156354	-0.000000
150 H :	0.162196	0.000000

HOMO: -6.33 eV

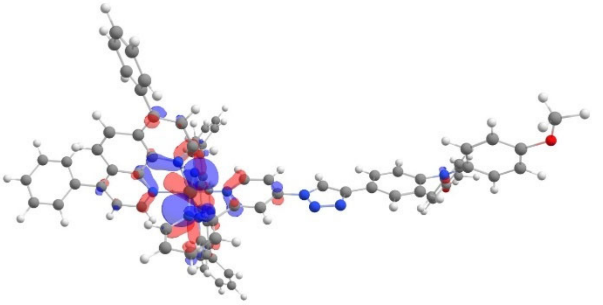

SOMO: -6.11 eV

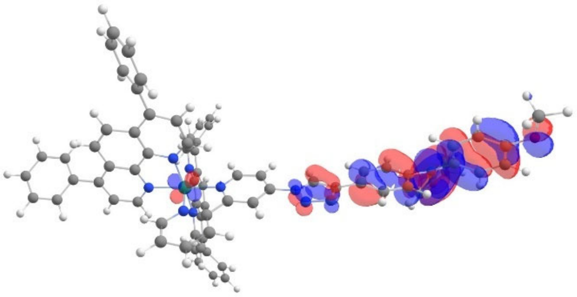

LUMO: -2.70 eV

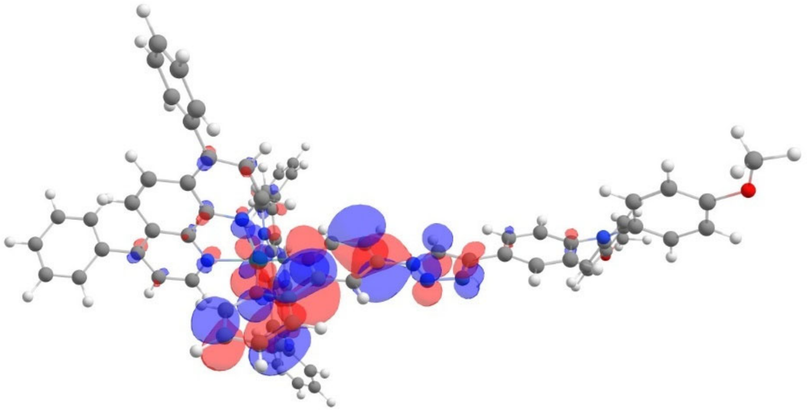

Figure S11

Ru-III$^{(2+)}$ in DMF

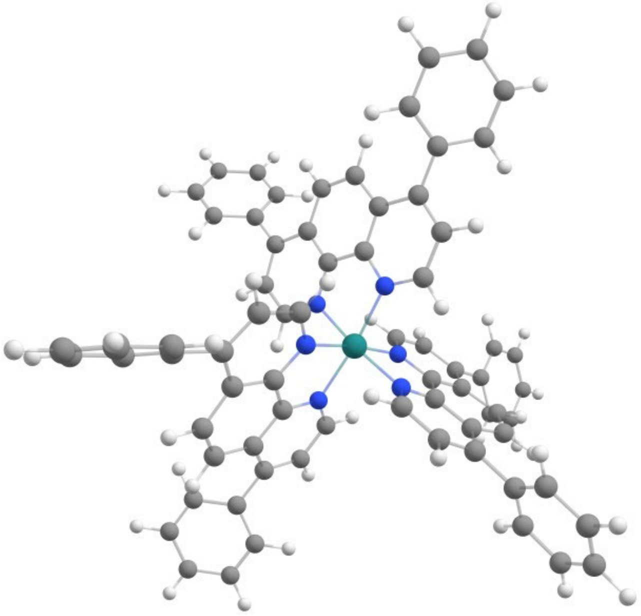

Free Energy = -2003460.2188 kcal/mol

COORDINATES:

44	-0.028483000	0.030324000	0.036483000	6	-4.098315000	-1.859799000	-2.709747000
7	-2.024579000	-0.174567000	0.461147000	7	1.955508000	0.018693000	-0.479173000
7	-0.373033000	1.984932000	-0.477065000	6	2.662600000	1.003864000	-1.009890000
6	-0.101434000	2.874523000	0.509750000	6	4.010022000	0.872315000	-1.324534000
6	-0.267382000	4.259947000	0.364692000	6	4.679131000	-0.313448000	-1.093738000
6	-0.724652000	4.745261000	-0.885026000	6	3.950075000	-1.365397000	-0.486725000
6	-1.023517000	3.811514000	-1.857517000	6	2.592289000	-1.147783000	-0.209821000
6	-0.838037000	2.453917000	-1.624322000	6	1.802563000	-2.169841000	0.388242000
6	-2.661073000	0.175003000	1.567627000	6	2.367585000	-3.414420000	0.703630000
6	-4.021379000	-0.045885000	1.750042000	6	1.525338000	-4.397833000	1.278099000
6	-4.777541000	-0.657905000	0.770349000	6	0.217433000	-4.039615000	1.539147000
6	-4.124130000	-0.997731000	-0.439669000	6	-0.254620000	-2.774135000	1.210953000
6	-2.750012000	-0.733394000	-0.539440000	7	0.507695000	-1.855250000	0.639233000
6	-2.036387000	-1.051827000	-1.728999000	6	4.518025000	-2.611153000	-0.087817000
6	-2.701312000	-1.602874000	-2.834611000	6	3.764916000	-3.586316000	0.476624000
6	-1.936341000	-1.889578000	-3.992015000	6	-6.205191000	-0.930174000	1.009066000
6	-0.578991000	-1.643532000	-3.933208000	6	-6.732943000	-2.210074000	0.837580000
6	-0.002425000	-1.092542000	-2.794796000	6	-8.069677000	-2.458220000	1.105910000
7	-0.707133000	-0.784111000	-1.717719000	6	-8.897191000	-1.431141000	1.539440000
6	0.371072000	2.326755000	1.735508000	6	-8.378735000	-0.155419000	1.715039000
7	0.478565000	0.975967000	1.784381000	6	-7.039475000	0.092776000	1.458670000
6	0.901583000	0.427521000	2.912481000	6	-2.528741000	-2.431785000	-5.226664000
6	1.249973000	1.181939000	4.026718000	6	-3.617823000	-1.811348000	-5.838731000
6	1.168240000	2.560234000	4.003339000	6	-4.135929000	-2.313917000	-7.021804000
6	0.683069000	3.163674000	2.817284000	6	-3.579681000	-3.445427000	-7.602889000
6	0.437346000	4.560491000	2.668644000	6	-2.493881000	-4.066723000	-7.001237000
6	-0.015975000	5.081682000	1.502697000	6	-1.966552000	-3.559355000	-5.824288000
6	-4.775635000	-1.568117000	-1.572569000	6	1.582340000	3.341334000	5.180600000

6	2.489107000	4.394956000	5.061575000
6	2.900737000	5.094891000	6.184482000
6	2.405990000	4.758225000	7.437236000
6	1.505049000	3.709650000	7.564197000
6	1.100946000	3.000262000	6.444307000
6	-0.882424000	6.180622000	-1.170910000
6	0.156406000	7.080095000	-0.929829000
6	0.007823000	8.421836000	-1.242702000
6	-1.181501000	8.883363000	-1.790495000
6	-2.219254000	7.994212000	-2.035092000
6	-2.068802000	6.649782000	-1.734402000
6	6.094139000	-0.445061000	-1.478273000
6	6.531113000	-1.516142000	-2.258079000
6	7.858506000	-1.603941000	-2.646274000
6	8.766523000	-0.629914000	-2.253454000
6	8.338963000	0.440069000	-1.479015000
6	7.009636000	0.536470000	-1.099409000
6	1.991695000	-5.758616000	1.590609000
6	1.739011000	-6.309266000	2.846821000
6	2.146896000	-7.600395000	3.142196000
6	2.798841000	-8.362079000	2.181894000
6	3.041963000	-7.826027000	0.924484000
6	2.645814000	-6.531114000	0.630499000
1	-1.378636000	4.127379000	-2.830075000
1	-1.054328000	1.729016000	-2.398173000
1	-2.068638000	0.628777000	2.351220000
1	-4.472444000	0.239816000	2.691680000
1	0.052088000	-1.847469000	-4.788675000
1	1.057801000	-0.877911000	-2.764193000
1	0.979171000	-0.651645000	2.932873000
1	1.615223000	0.669609000	4.907535000
1	0.596123000	5.210735000	3.518305000
1	-0.214510000	6.142685000	1.433098000
1	-5.837871000	-1.764343000	-1.519622000
1	-4.623927000	-2.303330000	-3.544366000
1	2.138015000	1.928326000	-1.213006000
1	4.520766000	1.709000000	-1.783727000
1	-0.468910000	-4.752433000	1.977892000
1	-1.283371000	-2.502627000	1.408987000
1	5.580014000	-2.766577000	-0.222133000
1	4.233190000	-4.510654000	0.786983000
1	-6.087981000	-3.020113000	0.516900000
1	-8.464969000	-3.459400000	0.979109000
1	-9.943833000	-1.625613000	1.743283000
1	-9.019290000	0.651163000	2.052506000
1	-6.637192000	1.090846000	1.591493000
1	-4.045510000	-0.917118000	-5.400021000
1	-4.975481000	-1.816314000	-7.493296000
1	-3.990030000	-3.840319000	-8.525124000
1	-2.055216000	-4.950863000	-7.449217000
1	-1.121855000	-4.049112000	-5.352736000
1	2.892662000	4.650739000	4.088615000
1	3.614023000	5.904253000	6.080234000
1	2.724558000	5.310535000	8.313741000
1	1.113827000	3.442792000	8.539142000
1	0.392843000	2.185257000	6.544313000
1	1.093597000	6.722425000	-0.519197000
1	0.826407000	9.108641000	-1.061172000
1	-1.298128000	9.934178000	-2.029267000
1	-3.150730000	8.348427000	-2.461329000
1	-2.882251000	5.957532000	-1.920757000
1	5.822851000	-2.269726000	-2.582566000
1	8.183495000	-2.435040000	-3.261380000
1	9.805567000	-0.703116000	-2.553233000
1	9.043217000	1.202878000	-1.167702000
1	6.677863000	1.369421000	-0.489753000
1	1.234860000	-5.713899000	3.599859000
1	1.955455000	-8.012332000	4.126271000
1	3.114380000	-9.373207000	2.411874000
1	3.539229000	-8.419791000	0.166264000
1	2.822180000	-6.124413000	-0.358608000

LOEWDIN ATOMIC CHARGES

0 Ru: 0.133285	59 C : -0.137849	118 H : 0.156398	
1 N : 0.218499	60 C : -0.096583	119 H : 0.155435	
2 N : 0.218215	61 C : -0.111064	120 H : 0.156256	
3 C : -0.105806	62 C : -0.100529	121 H : 0.162160	
4 C : -0.049337	63 C : -0.138498	122 H : 0.162076	
5 C : -0.035998	64 C : -0.136004	123 H : 0.156258	
6 C : -0.077411	65 C : -0.137808	124 H : 0.155435	
7 C : -0.144976	66 C : -0.096590	125 H : 0.156398	
8 C : -0.145070	67 C : -0.111008	126 H : 0.163538	
9 C : -0.077208	68 C : -0.100561		
10 C : -0.035713	69 C : -0.138468		
11 C : -0.049264	70 C : -0.136063		
12 C : -0.104920	71 C : -0.137823		
13 C : -0.104963	72 C : -0.096575		
14 C : -0.049103	73 C : -0.111087		
15 C : -0.035528	74 C : -0.096554		
16 C : -0.077286	75 C : -0.137826		
17 C : -0.144981	76 C : -0.135960		
18 N : 0.218609	77 C : -0.138446		
19 C : -0.105317	78 C : -0.100406		
20 N : 0.218536	79 H : 0.172672		
21 C : -0.145150	80 H : 0.166253		
22 C : -0.077308	81 H : 0.166105		
23 C : -0.035969	82 H : 0.172740		
24 C : -0.049300	83 H : 0.172780		
25 C : -0.076859	84 H : 0.166346		
26 C : -0.076814	85 H : 0.166064		
27 C : -0.076005	86 H : 0.172713		
28 C : -0.076102	87 H : 0.170315		
29 N : 0.218238	88 H : 0.170394		
30 C : -0.145283	89 H : 0.170103		
31 C : -0.077283	90 H : 0.170119		
32 C : -0.035792	91 H : 0.166334		
33 C : -0.049288	92 H : 0.172762		
34 C : -0.105421	93 H : 0.172719		
35 C : -0.104554	94 H : 0.166398		
36 C : -0.049125	95 H : 0.170387		
37 C : -0.035851	96 H : 0.170336		
38 C : -0.077317	97 H : 0.163341		
39 C : -0.145545	98 H : 0.156376		
40 N : 0.218663	99 H : 0.155392		
41 C : -0.076720	100 H : 0.156243		
42 C : -0.076717	101 H : 0.162076		
43 C : -0.110940	102 H : 0.163340		
44 C : -0.101383	103 H : 0.156378		
45 C : -0.138550	104 H : 0.155375		
46 C : -0.136472	105 H : 0.156226		
47 C : -0.137867	106 H : 0.162195		
48 C : -0.096933	107 H : 0.163414		
49 C : -0.110859	108 H : 0.156384		
50 C : -0.101559	109 H : 0.155454		
51 C : -0.138469	110 H : 0.156236		
52 C : -0.136409	111 H : 0.162092		
53 C : -0.137890	112 H : 0.163477		
54 C : -0.096806	113 H : 0.156384		
55 C : -0.111053	114 H : 0.155432		
56 C : -0.100540	115 H : 0.156240		
57 C : -0.138438	116 H : 0.162169		
58 C : -0.136037	117 H : 0.163430		

a) HOMO: -6.21 eV

b) LUMO: -2.55 eV

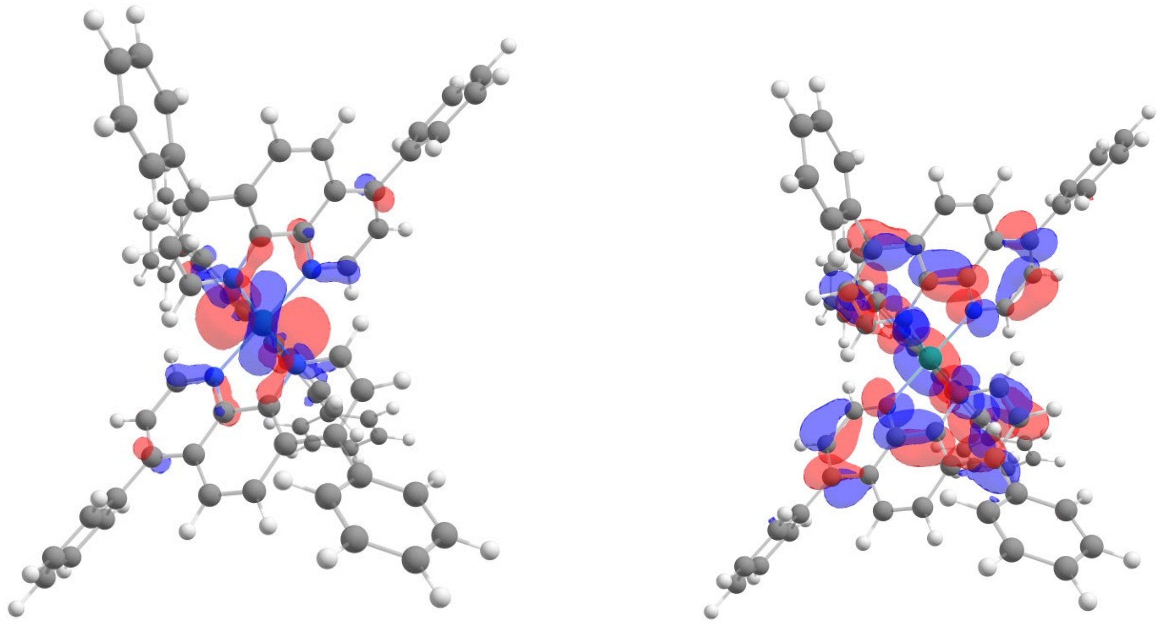

Figure S12. HOMO/LUMO orbitals of complex Ru-III

Ru-III$^{(2+)}$ in DMF-triplet

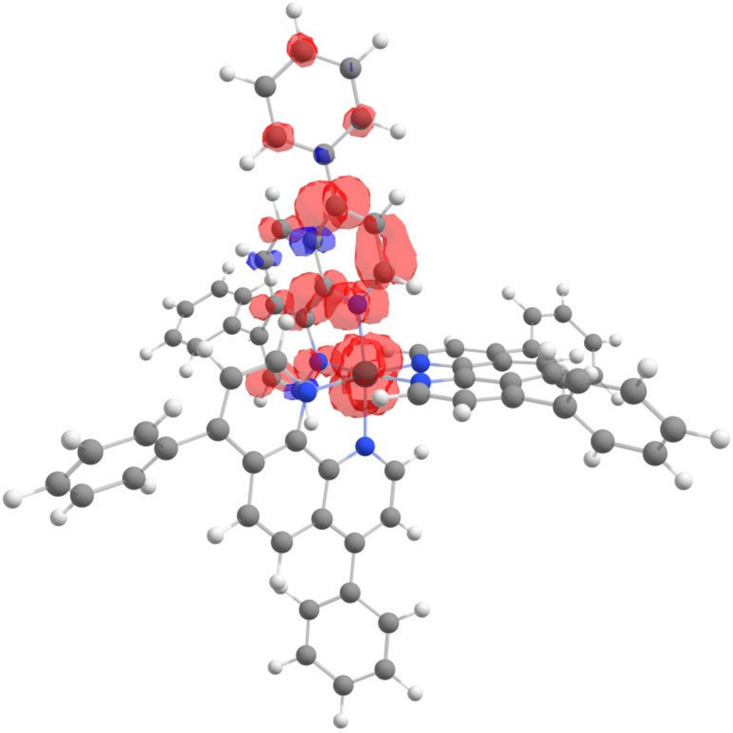

Free Energy = -2003416.9170 kcal/mol

Figure S13. Spin-Density of **R-III** triplet

Coordinates

44	-0.055127000	0.108642000	0.033903000	6	-4.723874000	-1.664024000	-1.494551000
7	-1.986276000	-0.117120000	0.475448000	6	-4.056415000	-1.974511000	-2.639111000
7	-0.387524000	2.051776000	-0.485956000	6	-4.723874000	-1.664024000	-1.494551000
6	-0.100726000	2.945400000	0.493128000	6	-4.056415000	-1.974511000	-2.639111000
6	-0.255266000	4.328143000	0.329771000	7	1.981336000	0.086082000	-0.408157000
6	-0.707861000	4.796648000	-0.930259000	6	2.721580000	1.094306000	-0.838803000
6	-1.025352000	3.853146000	-1.890523000	6	4.074187000	0.951528000	-1.119905000
6	-0.857505000	2.498901000	-1.640842000	6	4.707084000	-0.265424000	-0.954204000
6	-2.664793000	0.346507000	1.565486000	6	3.937856000	-1.341188000	-0.442975000
6	-3.985478000	0.103794000	1.754202000	6	2.577431000	-1.110862000	-0.196589000
6	-4.744885000	-0.665172000	0.819907000	6	1.750594000	-2.145440000	0.322149000
6	-4.088318000	-1.044656000	-0.375505000	6	2.276699000	-3.420061000	0.573418000
6	-2.719797000	-0.747233000	-0.517507000	6	1.393926000	-4.413641000	1.066537000
6	-2.032583000	-1.062235000	-1.691348000	6	0.089791000	-4.038771000	1.323506000
6	-2.677052000	-1.682034000	-2.792678000	6	-0.344879000	-2.744612000	1.064718000
6	-1.908477000	-1.969708000	-3.935289000	7	0.459466000	-1.820532000	0.567044000
6	-0.551076000	-1.668871000	-3.903082000	6	4.466284000	-2.621985000	-0.107742000
6	0.008715000	-1.055261000	-2.790277000	6	3.674889000	-3.612264000	0.372288000
7	-0.700001000	-0.736452000	-1.721607000	6	-6.132221000	-0.997321000	1.129499000
6	0.362821000	2.407205000	1.723833000	6	-4.723874000	-1.664024000	-1.494551000
7	0.460954000	1.057934000	1.789924000	6	-4.056415000	-1.974511000	-2.639111000
6	0.877252000	0.510430000	2.918420000	7	1.981336000	0.086082000	-0.408157000
6	1.230078000	1.274112000	4.025370000	6	2.721580000	1.094306000	-0.838803000
6	1.160670000	2.653051000	3.987322000	6	4.074187000	0.951528000	-1.119905000
6	0.680954000	3.252658000	2.795483000	6	4.707084000	-0.265424000	-0.954204000
6	0.445553000	4.648939000	2.631324000	6	-4.723874000	-1.664024000	-1.494551000
6	-0.001047000	5.161099000	1.458244000	6	-4.056415000	-1.974511000	-2.639111000

6	-4.723874000	-1.664024000	-1.494551000
6	-4.056415000	-1.974511000	-2.639111000
7	1.981336000	0.086082000	-0.408157000
6	2.721580000	1.094306000	-0.838803000
6	4.074187000	0.951528000	-1.119905000
6	4.707084000	-0.265424000	-0.954204000
6	3.937856000	-1.341188000	-0.442975000
6	2.577431000	-1.110862000	-0.196589000
6	1.750594000	-2.145440000	0.322149000
6	2.276699000	-3.420061000	0.573418000
6	1.393926000	-4.413641000	1.066537000
6	0.089791000	-4.038771000	1.323506000
6	-0.344879000	-2.744612000	1.064718000
7	0.459466000	-1.820532000	0.567044000
6	4.466284000	-2.621985000	-0.107742000
6	3.674889000	-3.612264000	0.372288000
6	-6.132221000	-0.997321000	1.129499000
6	-6.668333000	-2.266772000	0.872677000
6	-7.978002000	-2.569198000	1.205649000
6	-8.789971000	-1.614328000	1.805044000
6	-8.270499000	-0.355206000	2.079641000
6	-6.959386000	-0.052837000	1.753467000
6	-2.487841000	-2.566681000	-5.151585000
6	-3.600496000	-1.999403000	-5.773248000
6	-4.111653000	-2.550707000	-6.937705000
6	-3.523567000	-3.678975000	-7.493052000
6	-2.412914000	-4.247064000	-6.883717000
6	-1.893411000	-3.690485000	-5.725430000
6	1.581427000	3.441774000	5.155987000
6	2.490435000	4.491924000	5.023312000
6	2.907589000	5.199744000	6.138951000
6	2.414696000	4.875480000	7.395680000
6	1.511234000	3.830586000	7.535275000
6	1.102834000	3.111299000	6.423627000
6	-0.842997000	6.226977000	-1.240270000
6	0.201738000	7.117370000	-0.989123000
6	0.075629000	8.454610000	-1.328111000
6	-1.097028000	8.919965000	-1.907681000
6	-2.140327000	8.039501000	-2.160983000
6	-2.011674000	6.698437000	-1.838863000
6	6.129476000	-0.403324000	-1.302726000
6	6.569954000	-1.435045000	-2.132129000
6	7.907056000	-1.525301000	-2.483969000
6	8.819690000	-0.596075000	-2.003079000
6	8.388094000	0.433726000	-1.177995000
6	7.049349000	0.535822000	-0.836338000
6	1.818510000	-5.802184000	1.302916000
6	1.518068000	-6.422850000	2.515314000
6	1.888958000	-7.739031000	2.738661000
6	2.547906000	-8.454822000	1.748208000
6	2.836311000	-7.848322000	0.533069000
6	2.479785000	-6.527943000	0.311639000
1	-1.379571000	4.160783000	-2.865602000
1	-1.087616000	1.759539000	-2.396634000
1	-2.084266000	0.888701000	2.299630000
1	-4.452243000	0.459891000	2.663172000
1	0.078252000	-1.871791000	-4.759245000
1	1.059924000	-0.794511000	-2.779516000
1	0.944965000	-0.569649000	2.946609000
1	1.589851000	0.767683000	4.911549000
1	0.605158000	5.307083000	3.474563000
1	-0.194335000	6.222060000	1.377443000
1	-5.782183000	-1.878425000	-1.434942000
1	-4.588087000	-2.452863000	-3.450750000
1	2.225992000	2.044385000	-0.990707000
1	4.618138000	1.805214000	-1.502504000
1	-0.623465000	-4.758209000	1.704048000
1	-1.368428000	-2.451536000	1.257500000
1	5.528400000	-2.792959000	-0.219031000
1	4.113025000	-4.564934000	0.637367000
1	-6.039579000	-3.036076000	0.440500000
1	-8.364090000	-3.562581000	1.005889000
1	-9.815649000	-1.851620000	2.062757000
1	-8.893000000	0.398976000	2.548164000
1	-6.571992000	0.937984000	1.962449000
1	-4.053054000	-1.107928000	-5.354616000
1	-4.970667000	-2.093629000	-7.415441000
1	-3.927259000	-4.112207000	-8.400970000
1	-1.948122000	-5.128027000	-7.311459000
1	-1.028154000	-4.138456000	-5.249341000
1	2.892497000	4.737522000	4.047136000
1	3.623159000	6.005801000	6.025809000
1	2.736993000	5.434941000	8.266248000
1	1.121859000	3.574167000	8.513677000
1	0.392892000	2.299145000	6.532994000
1	1.126315000	6.755590000	-0.554544000
1	0.898301000	9.134920000	-1.141389000
1	-1.196371000	9.968076000	-2.165271000
1	-3.058308000	8.398001000	-2.611895000
1	-2.828668000	6.012555000	-2.032660000
1	5.858981000	-2.152402000	-2.525410000
1	8.236266000	-2.323492000	-3.138991000
1	9.866366000	-0.672687000	-2.273981000
1	9.096383000	1.160854000	-0.798256000
1	6.713884000	1.337249000	-0.187729000
1	1.008081000	-5.863316000	3.291451000
1	1.662586000	-8.206801000	3.689732000
1	2.833634000	-9.485845000	1.921863000
1	3.338636000	-8.406281000	-0.248556000
1	2.690985000	-6.065504000	-0.645660000

LOEWDIN ATOMIC CHARGES AND SPIN POPULATIONS

0 Ru:	0.372275	0.853342
1 N :	0.135625	0.198349
2 N :	0.211182	-0.002719
3 C :	-0.100509	0.007789
4 C :	-0.042363	-0.001662
5 C :	-0.018953	0.013832
6 C :	-0.066208	-0.002487
7 C :	-0.124699	0.010094
8 C :	-0.197972	0.119303
9 C :	-0.135901	0.075876
10 C :	-0.069692	0.234851
11 C :	-0.068143	-0.003482
12 C :	-0.134083	0.090463
13 C :	-0.125686	0.066973
14 C :	-0.068771	0.047886
15 C :	-0.045221	-0.000949
16 C :	-0.108130	0.077576
17 C :	-0.161874	-0.014398
18 N :	0.196641	0.064704
19 C :	-0.104546	0.000316
20 N :	0.217197	-0.001253
21 C :	-0.133210	-0.003320
22 C :	-0.067384	0.002174
23 C :	-0.025585	-0.002204
24 C :	-0.042631	0.000853
25 C :	-0.069140	-0.002609
26 C :	-0.069954	0.001781
27 C :	-0.089351	0.041137
28 C :	-0.093059	-0.021776
29 N :	0.213305	0.001379
30 C :	-0.134111	0.005037
31 C :	-0.068370	-0.000291
32 C :	-0.023839	0.003814
33 C :	-0.043108	-0.000790
34 C :	-0.103996	0.002619
35 C :	-0.101999	0.000735
36 C :	-0.042994	0.000553
37 C :	-0.025389	-0.001789
38 C :	-0.068216	0.002029
39 C :	-0.131400	-0.000263
40 N :	0.219945	0.005914
41 C :	-0.070678	0.001031
42 C :	-0.070130	-0.000937
43 C :	-0.120829	0.012464
44 C :	-0.111971	0.041938
45 C :	-0.149392	-0.010647
46 C :	-0.151215	0.049177
47 C :	-0.148906	-0.010972
48 C :	-0.107829	0.043087
49 C :	-0.109515	-0.000006
50 C :	-0.103275	-0.000179
51 C :	-0.140978	0.000544
52 C :	-0.139949	-0.000799
53 C :	-0.140549	0.000448
54 C :	-0.099378	-0.001317
55 C :	-0.110525	-0.000096
56 C :	-0.098307	-0.000259
57 C :	-0.136342	0.000064
58 C :	-0.132499	-0.000221
59 C :	-0.135612	0.000066
60 C :	-0.093806	-0.000243
61 C :	-0.109927	0.000804
62 C :	-0.096552	0.002277
63 C :	-0.135327	-0.000590
64 C :	-0.130161	0.002510
65 C :	-0.134505	-0.000544
66 C :	-0.092047	0.002293
67 C :	-0.110360	0.000232
68 C :	-0.097853	0.000550
69 C :	-0.136069	-0.000130
70 C :	-0.132040	0.000551
71 C :	-0.135483	-0.000103
72 C :	-0.093552	0.000519
73 C :	-0.110524	-0.000056
74 C :	-0.093863	-0.000207
75 C :	-0.135686	0.000058
76 C :	-0.132488	-0.000189
77 C :	-0.136352	0.000065
78 C :	-0.098015	-0.000224
79 H :	0.176215	-0.000007
80 H :	0.171459	-0.000150
81 H :	0.160029	-0.000203
82 H :	0.163772	-0.000034
83 H :	0.167607	0.000022
84 H :	0.165390	-0.000124
85 H :	0.169647	-0.000070
86 H :	0.175357	-0.000034
87 H :	0.172454	-0.000017
88 H :	0.172622	-0.000030
89 H :	0.165539	-0.000144
90 H :	0.166505	0.000003
91 H :	0.169462	-0.000011
92 H :	0.175599	0.000073
93 H :	0.175487	0.000219
94 H :	0.171420	0.000079
95 H :	0.172251	-0.000022
96 H :	0.172330	-0.000017
97 H :	0.159954	-0.000290
98 H :	0.154086	0.000231
99 H :	0.152257	-0.000062
100 H :	0.153929	0.000221
101 H :	0.158349	-0.000251
102 H :	0.162346	0.000196
103 H :	0.155689	-0.000028
104 H :	0.154643	0.000020
105 H :	0.155497	-0.000003
106 H :	0.161122	0.000064
107 H :	0.164224	-0.000002
108 H :	0.157013	-0.000003
109 H :	0.156137	0.000001
110 H :	0.156893	-0.000002
111 H :	0.163011	0.000004
112 H :	0.164766	-0.000014
113 H :	0.157347	0.000018
114 H :	0.156484	-0.000008
115 H :	0.157249	0.000016
116 H :	0.163593	0.000000
117 H :	0.164388	-0.000005
118 H :	0.157112	0.000007
119 H :	0.156188	-0.000002
120 H :	0.156966	0.000002
121 H :	0.163108	0.000007
122 H :	0.162954	0.000001
123 H :	0.156894	-0.000001
124 H :	0.156108	0.000001
125 H :	0.157026	-0.000002
126 H :	0.164331	0.000005

SOMO: -4.00 eV

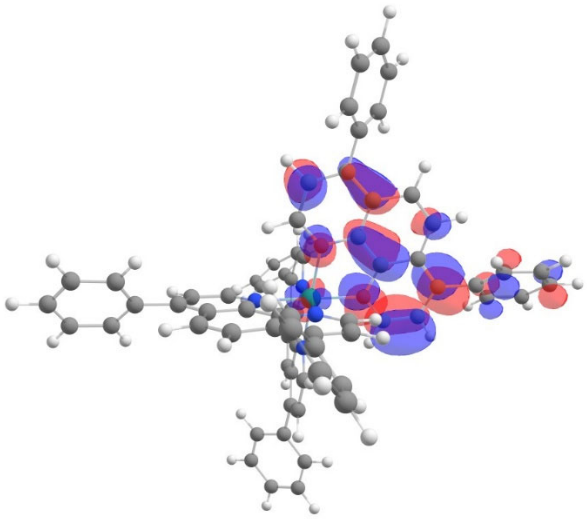

Second SOMO: -6.81 eV

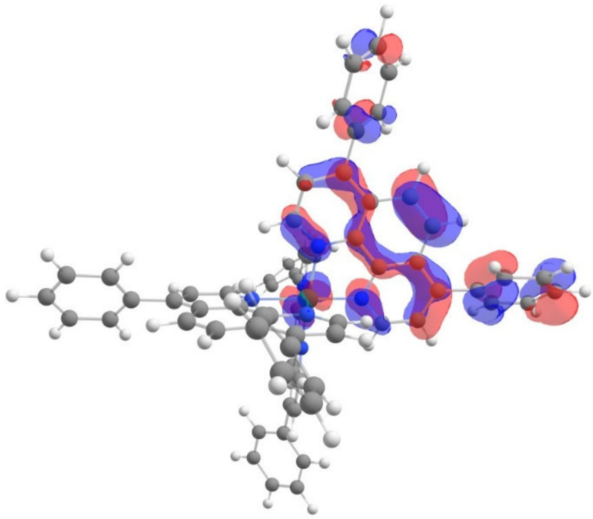

LUMO: -2.77 eV

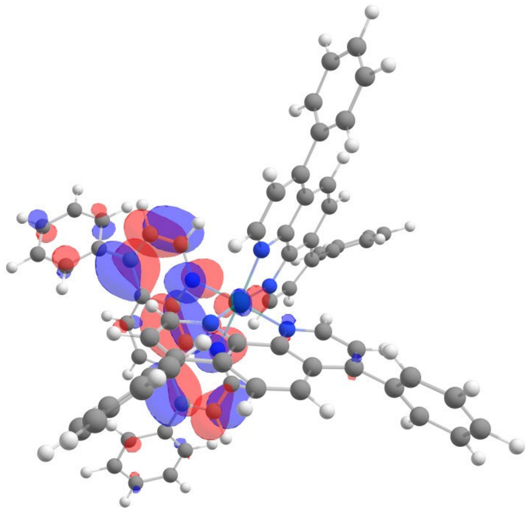

Figure S14. SOMO/LUMO orbitals of **Ru-III** triplet state

Ru-III$^{(3+)}$ in DMF

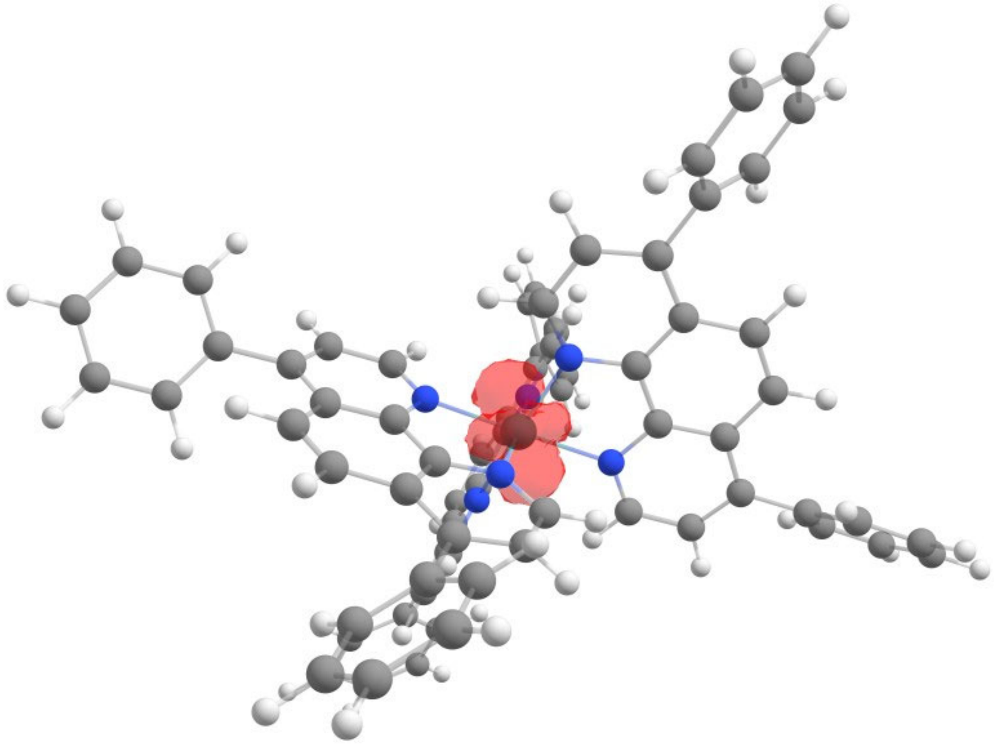

Free Energy = -2003334.0779 kcal/mol

Figure S15. Spin-Density of **Ru-III**$^{(3+)}$

Coordinates

44	-0.046312000	-0.002658000	0.038796000	6	0.747041000	3.138241000	2.783261000
7	-2.053602000	-0.163722000	0.450499000	6	0.477613000	4.530868000	2.646324000
7	-0.449908000	1.948836000	-0.456577000	6	-0.035253000	5.045229000	1.501698000
6	-0.136062000	2.839053000	0.517008000	6	-4.807973000	-1.532905000	-1.585478000
6	-0.326646000	4.219732000	0.377304000	6	-4.122253000	-1.877484000	-2.703177000
6	-0.849838000	4.692299000	-0.853536000	7	1.952075000	0.019495000	-0.434125000
6	-1.197598000	3.751084000	-1.804708000	6	2.652529000	1.039218000	-0.905832000
6	-0.990284000	2.397701000	-1.578897000	6	4.009129000	0.934417000	-1.178300000
6	-2.685716000	0.246312000	1.538924000	6	4.688781000	-0.249736000	-0.960466000
6	-4.052238000	0.069039000	1.705680000	6	3.959845000	-1.334855000	-0.409962000
6	-4.812722000	-0.551886000	0.732889000	6	2.591479000	-1.147870000	-0.176065000
6	-4.156745000	-0.950679000	-0.459672000	6	1.801240000	-2.191467000	0.372762000
6	-2.775710000	-0.732410000	-0.546959000	6	2.369001000	-3.437642000	0.667335000
6	-2.057605000	-1.096269000	-1.715808000	6	1.514542000	-4.446557000	1.181287000
6	-2.718039000	-1.662197000	-2.814065000	6	0.193386000	-4.111055000	1.411640000
6	-1.937808000	-2.002530000	-3.948871000	6	-0.284872000	-2.844126000	1.107295000
6	-0.572150000	-1.804195000	-3.870047000	7	0.494829000	-1.905294000	0.594661000
6	0.003177000	-1.238484000	-2.740728000	6	4.532939000	-2.581134000	-0.023391000
7	-0.721140000	-0.868633000	-1.696297000	6	3.774971000	-3.583368000	0.485600000
6	0.391436000	2.299408000	1.719117000	6	-6.249798000	-0.766766000	0.958883000
7	0.517354000	0.950660000	1.769832000	6	-6.824579000	-2.027813000	0.797810000
6	1.001735000	0.396863000	2.870305000	6	-8.172680000	-2.217275000	1.054825000
6	1.391157000	1.156284000	3.964953000	6	-8.962537000	-1.150379000	1.461460000
6	1.296204000	2.535114000	3.943496000	6	-8.396297000	0.106660000	1.625134000

6	-7.045263000	0.296830000	1.385200000
6	-2.523470000	-2.551109000	-5.181059000
6	-3.596380000	-1.920243000	-5.811552000
6	-4.106128000	-2.428459000	-6.995275000
6	-3.560630000	-3.576107000	-7.554426000
6	-2.491736000	-4.207575000	-6.933022000
6	-1.968501000	-3.693407000	-5.757971000
6	1.762538000	3.318079000	5.096663000
6	2.634958000	4.393988000	4.927832000
6	3.098601000	5.094978000	6.028991000
6	2.687278000	4.739240000	7.306423000
6	1.820251000	3.669040000	7.481481000
6	1.367471000	2.954893000	6.384316000
6	-1.026050000	6.122406000	-1.141469000
6	0.011493000	7.030689000	-0.926681000
6	-0.155578000	8.368422000	-1.245106000
6	-1.361556000	8.816001000	-1.767408000
6	-2.397761000	7.917607000	-1.984307000
6	-2.229044000	6.576235000	-1.683047000
6	6.116808000	-0.343033000	-1.295348000
6	6.608520000	-1.395991000	-2.067527000
6	7.950884000	-1.444106000	-2.406646000
6	8.817540000	-0.451923000	-1.968511000
6	8.334644000	0.599045000	-1.200398000
6	6.990112000	0.659845000	-0.873291000
6	1.982004000	-5.811075000	1.463096000
6	1.667250000	-6.413313000	2.681563000
6	2.078255000	-7.709120000	2.947441000
6	2.789881000	-8.423764000	1.993156000
6	3.091900000	-7.836570000	0.771863000
6	2.697153000	-6.535219000	0.508364000
1	-1.605992000	4.058934000	-2.758237000
1	-1.242233000	1.667946000	-2.336997000
1	-2.091773000	0.707614000	2.316737000
1	-4.506894000	0.396579000	2.631332000
1	0.067476000	-2.053396000	-4.706431000
1	1.069121000	-1.058053000	-2.696766000
1	1.091709000	-0.681245000	2.886164000
1	1.802838000	0.647679000	4.826778000
1	0.664940000	5.183458000	3.488012000
1	-0.253585000	6.102614000	1.441906000
1	-5.875975000	-1.696123000	-1.544125000
1	-4.647678000	-2.332071000	-3.531599000
1	2.120728000	1.961878000	-1.097099000
1	4.520876000	1.791822000	-1.595154000
1	-0.498819000	-4.844037000	1.804504000
1	-1.322552000	-2.589826000	1.278493000
1	5.601560000	-2.716068000	-0.119693000
1	4.246349000	-4.508333000	0.788474000
1	-6.209054000	-2.868258000	0.498723000
1	-8.606550000	-3.203556000	0.939185000
1	-10.018590000	-1.299600000	1.654327000
1	-9.008475000	0.943131000	1.941402000
1	-6.604843000	1.279798000	1.508789000
1	-4.014106000	-1.012971000	-5.390638000
1	-4.930956000	-1.923254000	-7.484020000
1	-3.966148000	-3.976114000	-8.476543000
1	-2.062606000	-5.104140000	-7.364921000
1	-1.136786000	-4.190154000	-5.271076000
1	2.974024000	4.663802000	3.934352000

1	-1.136786000	-4.190154000	-5.271076000
1	2.974024000	4.663802000	3.934352000
1	3.786440000	5.920493000	5.888269000
1	3.045308000	5.294026000	8.165921000
1	1.495110000	3.388433000	8.476485000
1	0.686672000	2.122326000	6.521551000
1	0.961618000	6.683477000	-0.537668000
1	0.661145000	9.063023000	-1.087353000
1	-1.492577000	9.864483000	-2.008764000
1	-3.341577000	8.262341000	-2.390326000
1	-3.040650000	5.876613000	-1.848099000
1	5.933681000	-2.163232000	-2.428784000
1	8.320170000	-2.259004000	-3.018161000
1	9.868775000	-0.496025000	-2.228661000
1	9.007236000	1.375083000	-0.854258000
1	6.614784000	1.477870000	-0.268841000
1	1.115923000	-5.854383000	3.429309000
1	1.841599000	-8.162240000	3.903007000
1	3.106101000	-9.439660000	2.199816000
1	3.634901000	-8.394941000	0.018348000
1	2.918438000	-6.089171000	-0.454260000

LOEWDIN ATOMIC CHARGES AND SPIN POPULATIONS

0 Ru:	0.414875	0.924414
1 N :	0.212168	0.000147
2 N :	0.211680	-0.000008
3 C :	-0.101914	0.004887
4 C :	-0.039228	-0.000719
5 C :	-0.017154	0.004730
6 C :	-0.062296	0.000869
7 C :	-0.124558	0.001869
8 C :	-0.124531	0.001649
9 C :	-0.062144	0.000946
10 C :	-0.017112	0.004288
11 C :	-0.039057	-0.000662
12 C :	-0.100523	0.004987
13 C :	-0.101059	0.004803
14 C :	-0.038952	-0.000598
15 C :	-0.017009	0.003866
16 C :	-0.062119	0.001075
17 C :	-0.124656	0.001300
18 N :	0.212399	0.000280
19 C :	-0.101072	0.004824
20 N :	0.212438	0.000299
21 C :	-0.125231	0.000873
22 C :	-0.062146	0.001317
23 C :	-0.017814	0.003046
24 C :	-0.038998	-0.000512
25 C :	-0.065539	-0.000674
26 C :	-0.065675	-0.000349
27 C :	-0.064590	-0.000465
28 C :	-0.064698	-0.000579
29 N :	0.212171	0.000003
30 C :	-0.124963	0.001607
31 C :	-0.062238	0.000980
32 C :	-0.017175	0.004352
33 C :	-0.039081	-0.000667
34 C :	-0.101204	0.004839
35 C :	-0.100535	0.004789
36 C :	-0.038964	-0.000558
37 C :	-0.017646	0.003298
38 C :	-0.062167	0.001241
39 C :	-0.125446	0.001140
40 N :	0.212313	0.000344
41 C :	-0.065420	-0.000406
42 C :	-0.065532	-0.000612
43 C :	-0.110061	0.000332
44 C :	-0.097308	0.000743
45 C :	-0.134551	-0.000176
46 C :	-0.129682	0.000816
47 C :	-0.133829	-0.000128
48 C :	-0.092072	0.000711
49 C :	-0.109972	0.000304
50 C :	-0.097203	0.000699
51 C :	-0.134477	-0.000166
52 C :	-0.129490	0.000778
53 C :	-0.133844	-0.000119
54 C :	-0.091933	0.000668
55 C :	-0.110094	0.000285
56 C :	-0.096177	0.000666
57 C :	-0.134428	-0.000165
58 C :	-0.129100	0.000878
59 C :	-0.133795	-0.000167
60 C :	-0.091638	0.000740
61 C :	-0.110060	0.000360
62 C :	-0.095977	0.000900
63 C :	-0.134349	-0.000220
64 C :	-0.128658	0.001024
65 C :	-0.133603	-0.000169
66 C :	-0.091411	0.000863
67 C :	-0.110071	0.000334
68 C :	-0.096295	0.000815
69 C :	-0.134373	-0.000199
70 C :	-0.128989	0.000920
71 C :	-0.133720	-0.000148
72 C :	-0.091532	0.000781
73 C :	-0.110107	0.000306
74 C :	-0.091587	0.000787
75 C :	-0.133735	-0.000178
76 C :	-0.128936	0.000933
77 C :	-0.134400	-0.000175
78 C :	-0.096100	0.000711
79 H :	0.177562	-0.000014
80 H :	0.172251	-0.000090
81 H :	0.172086	-0.000084
82 H :	0.177644	-0.000011
83 H :	0.177635	-0.000012
84 H :	0.172202	-0.000084
85 H :	0.172142	-0.000068
86 H :	0.177581	0.000020
87 H :	0.173804	-0.000030
88 H :	0.173809	-0.000026
89 H :	0.173432	-0.000030
90 H :	0.173510	-0.000030
91 H :	0.172294	-0.000091
92 H :	0.177678	-0.000011
93 H :	0.177593	0.000025
94 H :	0.172157	-0.000065
95 H :	0.173786	-0.000024
96 H :	0.173769	-0.000031
97 H :	0.164770	-0.000004
98 H :	0.157613	0.000008
99 H :	0.156723	-0.000003
100 H :	0.157473	0.000004
101 H :	0.163618	0.000010
102 H :	0.164838	-0.000004
103 H :	0.157628	0.000007
104 H :	0.156725	-0.000003
105 H :	0.157458	0.000003
106 H :	0.163727	0.000010
107 H :	0.164895	-0.000003
108 H :	0.157602	0.000003
109 H :	0.156790	-0.000003
110 H :	0.157462	0.000005
111 H :	0.163623	-0.000000
112 H :	0.165041	-0.000006
113 H :	0.157686	0.000008
114 H :	0.156848	-0.000003
115 H :	0.157526	0.000004
116 H :	0.163753	0.000010
117 H :	0.164926	-0.000005
118 H :	0.157657	0.000007
119 H :	0.156809	-0.000003
120 H :	0.157522	0.000004
121 H :	0.163731	0.000010
122 H :	0.163632	-0.000001
123 H :	0.157512	0.000006
124 H :	0.156805	-0.000003
125 H :	0.157657	0.000003
126 H :	0.164965	-0.000003

SOMO: -7.24 eV LUMO: -2.96 eV

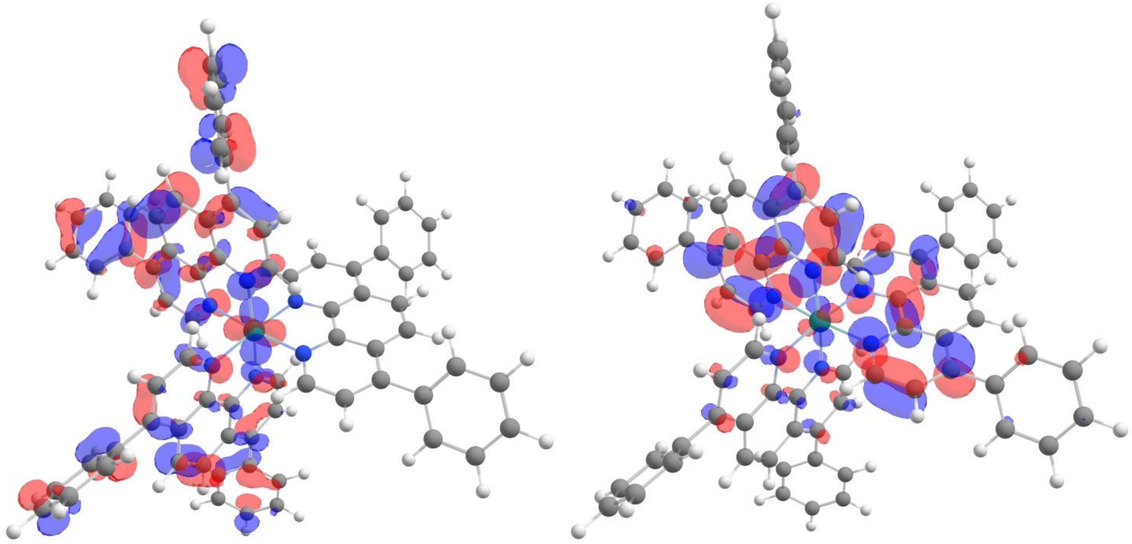

Figure S16. SOMO/LUMO orbitals of **Ru-III**$^{3+}$

9. fs-TAS and LFP quenching experiments

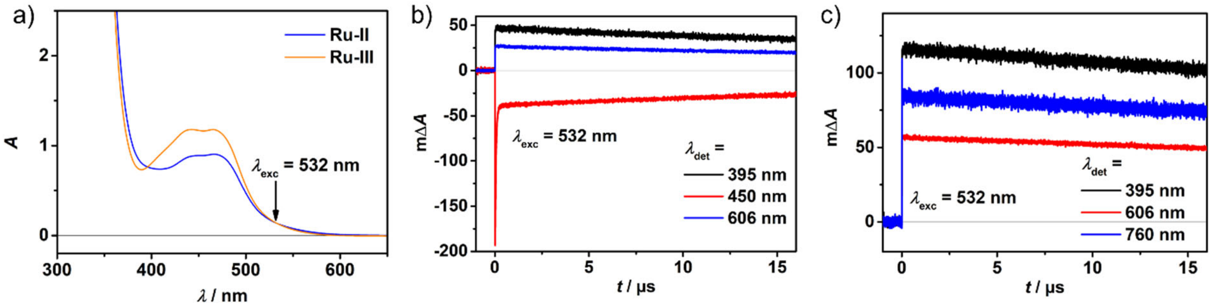

Figure S17 (a) UV-vis absorption spectra of **Ru-II** (30 µM) and **Ru-III** (38 µM) in Ar-saturated DMF. (b) Time-resolved absorption traces of **Ru-III** (38 µM) with MV^{2+} (50 mM) at different detection wavelengths in Ar-saturated DMF after laser excitation (λ_{exc} = 532 nm). (c) Time-resolved absorption traces of **Ru-II** (30 µM) with MV^{2+} (50 mM) at different detection wavelength in Ar-saturated DMF after laser excitation (λ_{exc} = 532 nm). For further details, see **Figure 5** and text in the main part.

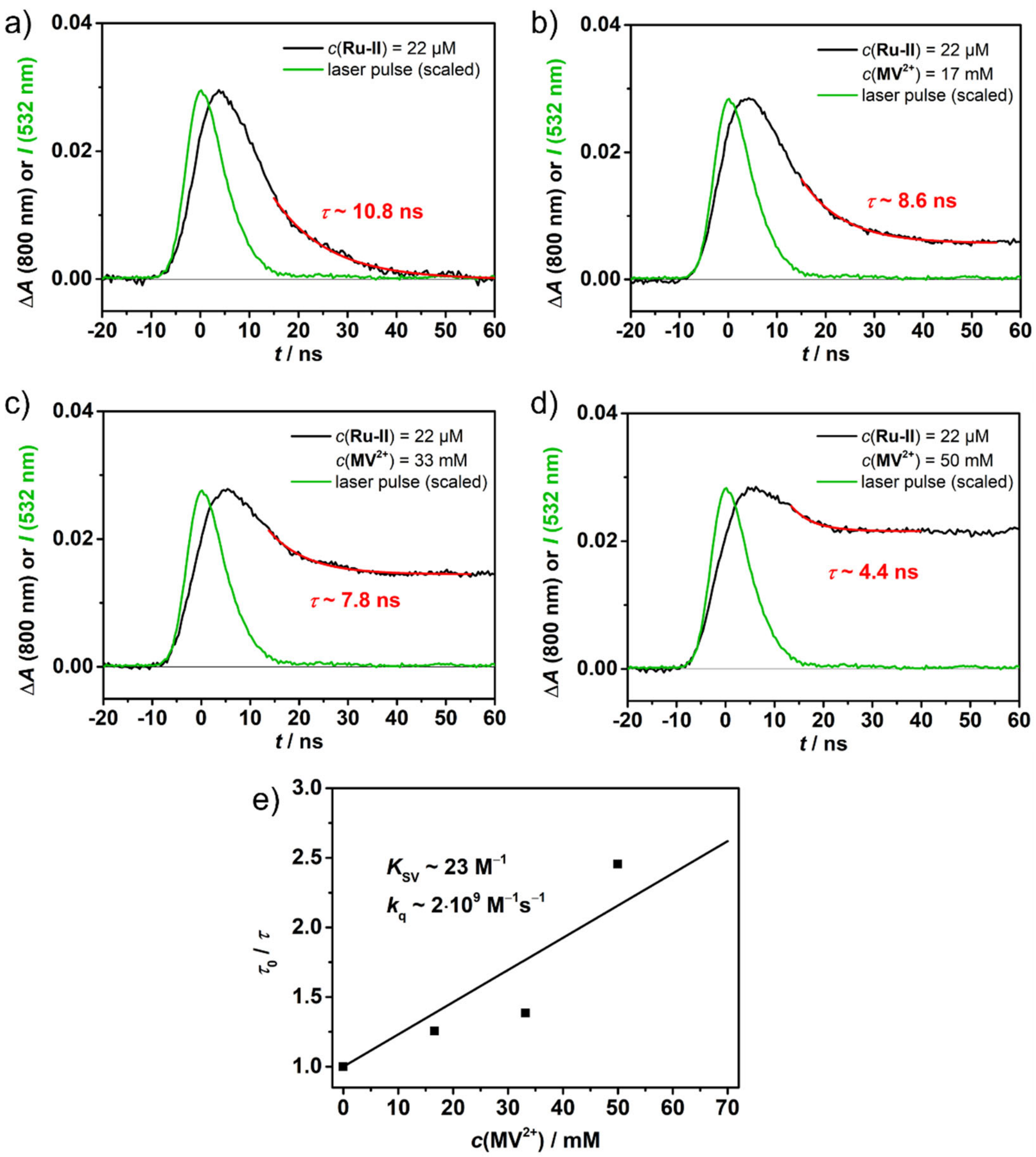

Figure S18 (a)–(d) Time-resolved detection of the laser pulse (scattered laser light of a water-containing cuvette) as well as time-resolved absorption traces of solutions containing 22 μM **Ru-II** and different concentrations of methyl viologen (MV^{2+}) in Ar-saturated DMF after laser excitation (λ_{exc} = 532 nm, λ_{det} = 793–807 nm) along with exponential fitting function after the laser pulse for lifetime-determination. (e) Corresponding Stern–Volmer plot for the estimation of K_{SV} and k_q.

The quenching of ^{3}**Ru-II** by MV^{2+} was analyzed via the transient absorption at 800 nm (see also **Figure 4** and **5** in the main part). Due to the short lifetime of ^{3}**Ru-II**, the exponential fit for lifetime estimation was applied when the laser pulse was finished. From this, the quenching rate was estimated using a Stern–Volmer plot (see **Figure S18** e). Due to the laser pulse duration of about 5 ns for this setup and a limited time-resolution, the value of the unquenched lifetime slightly differs from that shown in **Figure 5** of the main paper.

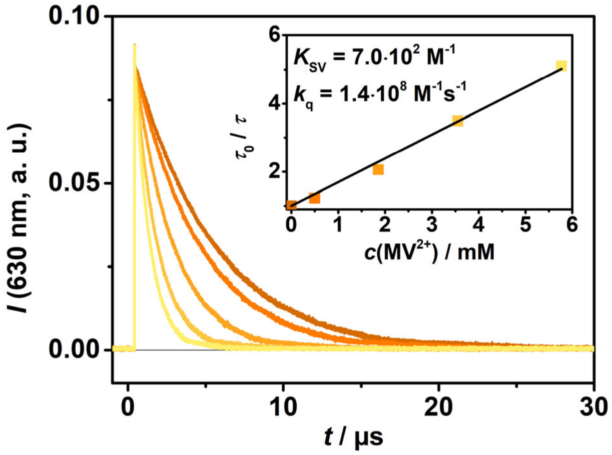

Figure S19 (Main plot) Time-resolved emission of solutions containing 20 µM **Ru-III** and different concentrations of MV^{2+} after laser excitation in Ar-saturated DMF (λ_{exc} = 532 nm, λ_{det} = 630 nm). (Inset) Corresponding Stern–Volmer plot.

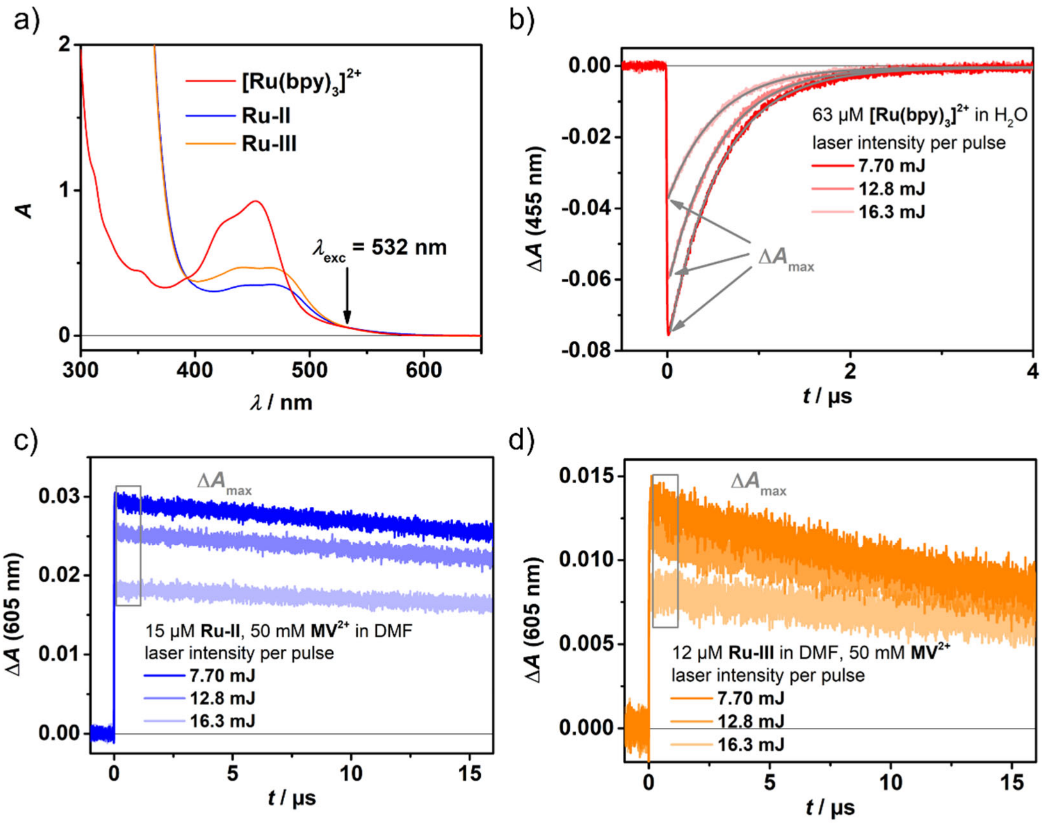

Figure S20 (a) UV-vis absorption spectra of solutions containing 63 µM **[Ru(bpy)$_3$]$^{2+}$** in H_2O, 15 µM **Ru-II** and 50 mM MV^{2+} in DMF or 12 µM **Ru-III** and 50 mM MV^{2+} in DMF. (b) Time-resolved absorption traces of a solution containing 63 µM **[Ru(bpy)$_3$]$^{2+}$** after laser excitation with different excitation energies (λ_{exc} = 532 nm, λ_{det} = 455 nm) along with exponential fitting functions for the determination of the amplitude. (c) Time-resolved absorption traces of a solution containing 15 µM **Ru-**

II after laser excitation with different excitation energies (λ_{exc} = 532 nm, λ_{det} = 605 nm) and an indication for the time-window for the determination of ΔA_{max} (mean value within gray box). (d) Time-resolved absorption traces of a solution containing 12 µM **Ru-III** after laser excitation with different excitation energies (λ_{exc} = 532 nm, λ_{det} = 605 nm) and an indication for the time-window for the determination of ΔA_{max} (mean value within gray box).

The cage escape quantum yield of the oxidation of ^{3}**Ru-II** or ^{3}**Ru-III** by MV^{2+} was determined by a relative actinometry using tris(2,2'-bipyridine)ruthenium(II) chloride (**[Ru(bpy)$_3$]$^{2+}$**) in water as a reference.

For this, the same absorption of a **[Ru(bpy)$_3$]$^{2+}$** solution and the **Ru-II** and **Ru-III** solutions was set at the excitation wavelength to ensure that the same number of photons is absorbed by each solution (see **Figure S20** a). Using the well-known differential molar absorption coefficient of ^{3}**[Ru(bpy)$_3$]$^{2+}$** at 455 nm ($\Delta\varepsilon_{455\,nm}$(^{3}**[Ru(bpy)$_3$]$^{2+}$**) = -10100 $M^{-1}cm^{-1}$)[22,23], the concentration of the excited states in the measuring window of the cuvette after laser excitation can be determined by the value ΔA_{max} (see **Figure S20** b). Similarly, the concentration of the quenching product after cage escape $MV^{\bullet+}$ was determined using the known extinction coefficient at 605 nm ($\Delta\varepsilon_{605\,nm}(MV^{\bullet+})$ ~ 13000 $M^{-1}cm^{-1}$)[24], with the assumption that this value is virtually independent of the solvent (see **Figure S20** c and d). The quenching efficiency η_q of ^{3}**Ru-II** and ^{3}**Ru-III** by MV^{2+} was determined using the quenching rate k_q (see **Figure S18** and **S19**), the unquenched lifetime (see **Figure 4** in the main part) and the quencher concentration (50 mM), which amounts to η_q(^{3}**Ru-II**,MV^{2+}) = 0.52 and η_q(^{3}**Ru-III**,MV^{2+}) = 0.98. With these values, the cage escape efficiency η_{CE} can be determined by formula S1 and S2. In the case of quenching of ^{3}**Ru-II** by MV^{2+}, only ~80% of the transient absorption at 605 nm is caused by the resulting $MV^{\bullet+}$ (see **Figure 5** in the main part), which is taken into account by a correction factor of 0.8 in formula S1.

$$\eta_{CE}(^3\mathbf{Ru{-}II},\mathbf{MV^{2+}}) = \frac{(\Delta A_{max,605\,nm}(^3\mathbf{Ru{-}II},\mathbf{MV^{2+}})\cdot 0.8)/\Delta\varepsilon_{605\,nm}(\mathbf{MV^{\bullet+}})}{(\Delta A_{max,455\,nm}(^3[\mathbf{Ru(bpy)}_3]^{2+})/\Delta\varepsilon_{455\,nm}(^3[\mathrm{Ru(bpy)}_3]^{2+}))\cdot\eta_q(^3\mathbf{Ru{-}II},\mathbf{MV^{2+}})} \tag{S1}$$

$$\eta_{CE}(^3\mathbf{Ru{-}III},\mathbf{MV^{2+}}) = \frac{\Delta A_{max,605\,nm}(^3\mathbf{Ru{-}III},\mathbf{MV^{2+}})/\Delta\varepsilon_{605\,nm}(\mathbf{MV^{\bullet+}})}{(\Delta A_{max,455\,nm}(^3[\mathbf{Ru(bpy)}_3]^{2+})/\Delta\varepsilon_{455\,nm}(^3[\mathbf{Ru(bpy)}_3]^{2+}))\cdot\eta_q(^3\mathbf{Ru{-}III},\mathbf{MV^{2+}})} \tag{S2}$$

The cage escape efficiency was determined for each laser intensity. The mean values with their corresponding standard deviations amount to

$\eta_{\mathrm{CE,mean}}(^{3}\mathbf{Ru\text{–}II}, \mathbf{MV^{2+}}) = 0.5 \pm 0.1$[1] and $\eta_{\mathrm{CE,mean}}(^{3}\mathbf{Ru\text{–}III}, \mathbf{MV^{2+}}) = 0.15 \pm 0.02$.

To gain a better understanding of the excited-state dynamics of **Ru-I** and **Ru-II**, transient absorption spectra were measured on a ps-to-ns time scale. A 100 µM Ar-saturated solution was investigated with the HARPIA-Light instrument from Light Conversion at an excitation wavelength of 515 nm. The fs-TAS contour plot (see **Figure S21** a) shows the ground state bleach (GSB) at 470 nm, which decreases with time while simultaneously a positive signal forms at 755 nm. The GSB at 470 nm is caused by Ru^{III} formation after excitation, while the signal at ~ 750 nm is typical for the oxidized TAA radical cation.[25–27] From the decay of the Ru^{III} signal when the TAA radical cation signal is formed, it can therefore be concluded that there is an electron transfer from the TAA to Ru^{III}. See main paper for a more detailed explanation.

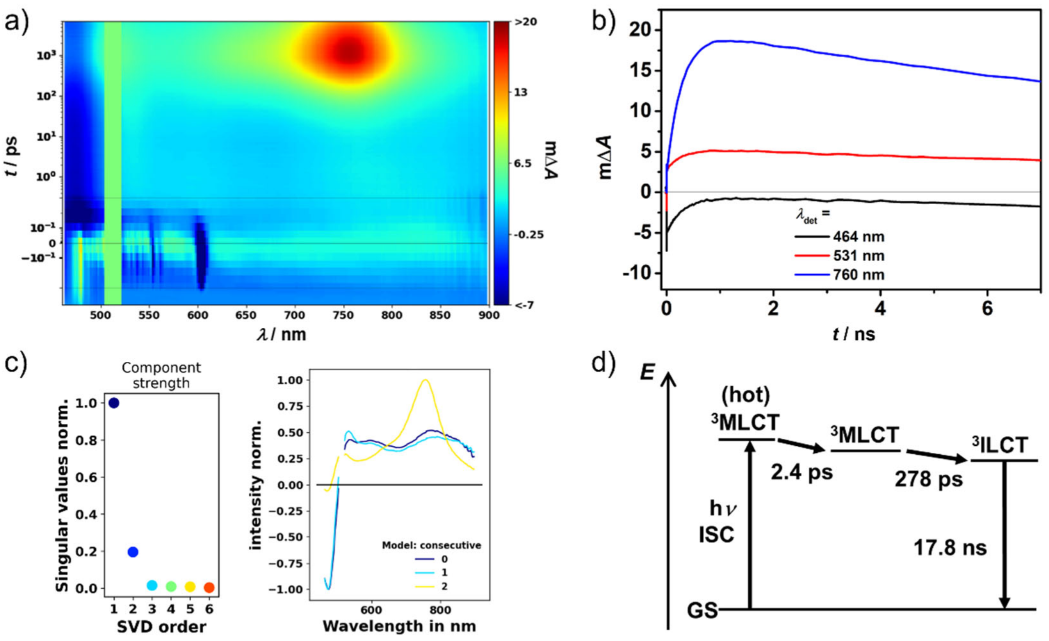

Figure S21 (a) Contour plot of the fs-TAS of **Ru-I** (100 µM) in Ar-saturated DMF after laser excitation (λ_{exc} = 515 nm, pulse length: ≤290 fs). (b) Time-resolved transient absorption traces at selected wavelengths. (c) Singular value decomposition (SVD) analysis and decay associated spectra of a three-component consecutive fitting model (range from -0.5 – 0.5 ps was omitted). (d) Schematic representation of the intramolecular processes upon photoexcitation of **Ru-I** with corresponding time constants from the global fit analysis.

[1] In addition to the mathematical standard deviation based on the three repetitions shown in **Figure S20**, an additional relative error of 10% is assumed, which results from the uncertainty in the estimation of k_q (see **Figure S18**) due to the limited time resolution.

The singular value decomposition (see **Figure S21** c) shows the relevance of the three rate constants along with the associated TA spectra. A three-component consecutive fitting model yielded the time constants shown in the simplified associated energy diagram (see **Figure S21** d). Thus, the decay associated spectra of a three-component consecutive model show very similar spectra for the first two components, with the characteristic GSB at 470 nm. For the third spectrum, a completely different species has formed, in which the GSB has decreased and a new signal has formed at 755 nm. We conclude that those spectra are associated with a ^{3}MLCT (hot state), ^{3}MLCT state after vibrational cooling and the ^{3}ILCT state with a lifetime of 17.8 ns (see also main paper).

For **Ru-II** a similar fs-TAS experiment was carried out (see **Figure S22** a). The SVD analysis reveals the relevance of three rate constants which leads to the decay associated spectra and time constants shown in **Figure S22** c and d.

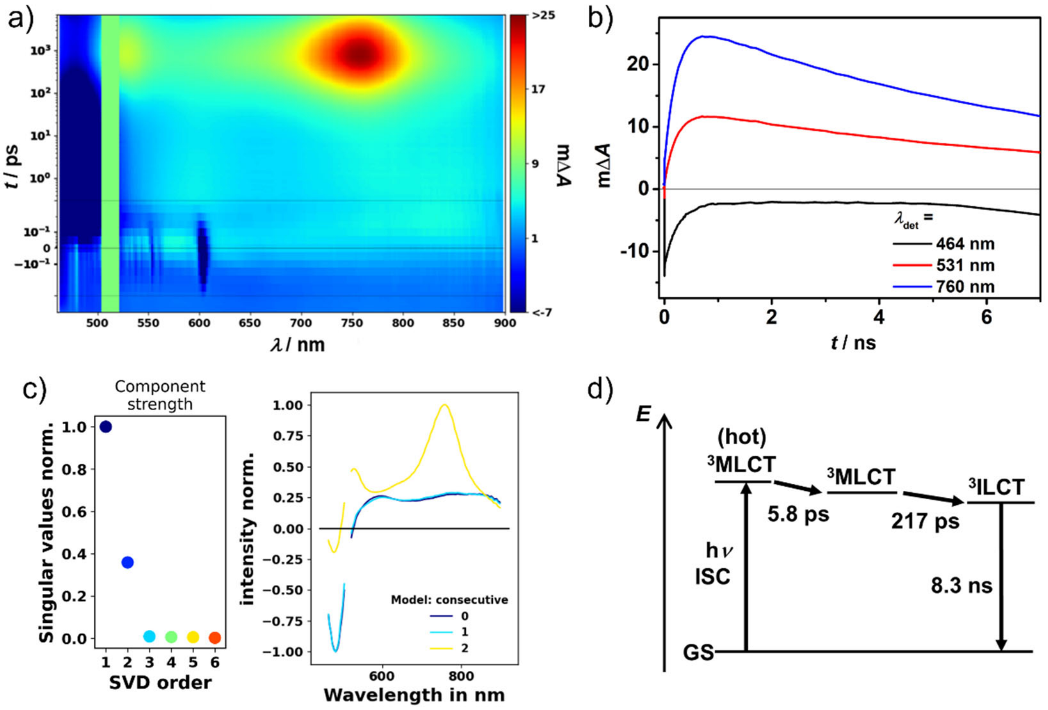

Figure S22 (a) Contour plot of the fs-TAS of **Ru-II** (100 µM) in Ar-saturated DMF after laser excitation (λ_{exc} = 515 nm, pulse length: ≤290 fs). (b) Time-resolved transient absorption traces at selected wavelengths. (c) Singular value decomposition (SVD) analysis and decay associated spectra of a three-component consecutive fitting model (range from -0.5 – 0.5 ps was omitted). (d) Schematic representation of the intramolecular processes upon photoexcitation of **Ru-II** with corresponding time constants from the global fit analysis (see also **Figure 4** and text in main part).

For **Ru-III** the fs-TAS contour plot is quite different (see **Figure S23** a). There is still the GSB at 480 nm and the radical anion of the diimine ligand at 560 nm, but no signal at ~750 nm can be observed in the complex without the TAA. This matches the expectations as the ~750 nm signal is assigned to the TAA radical cation. The SVD analysis with the decay-assigned spectra reveals a two-component

consecutive model (see **Figure S23** c) with two very similar species. This leads to the simplified energy diagram (see **Figure S23** d) with the ^{3}MLCT (hot state) that relaxes to the ^{3}MLCT with an associated lifetime of 13 ps.

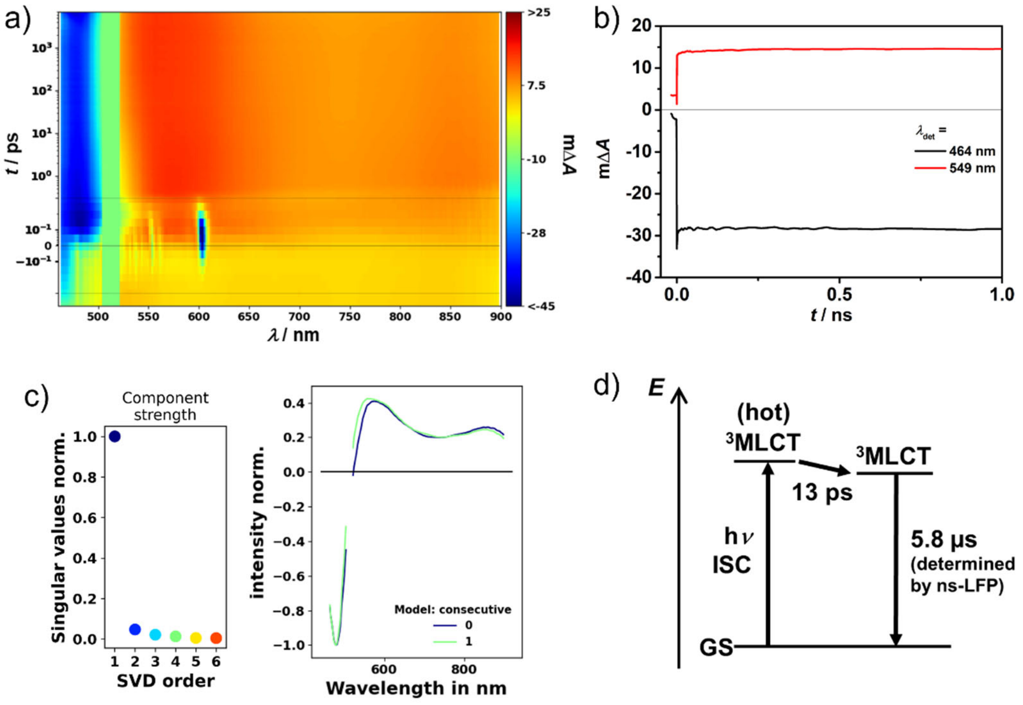

Figure S23 (a) Contour plot of the fs-TAS of **Ru-III** (100 µM) in Ar-saturated DMF after laser excitation (λ_{exc} = 515 nm, pulse length: ≤290 fs). (b) Time-resolved transient absorption traces at selected wavelengths. (c) Singular value decomposition (SVD) analysis and decay associated spectra of a two-component consecutive fitting model (range from -0.5 – 0.5 ps was omitted). (d) Schematic representation of the intramolecular processes upon photoexcitation of **Ru-III** with the corresponding time constant (13 ps) from the global fit analysis (see also Figure 5 in main part). The longer time constant was determined by ns-LFP.

To further investigate the light-induced oxidation of the complexes, a quenching study was carried out. A solution containing 40 µM **Ru-I** in DMF was investigated with and without 10 mM $TTCF_3OTf$, which serves as the oxidizing quencher of the catalyst in the mechanism under study. However, the results for the sample without the quencher $TTCF_3OTf$ showed a shorter lifetime (20.3 ns) than after the addition of the quencher (21.4 ns) (see **Figure S24**). This already indicates a more complex process. We suspect that counterion effects play a major role, which show significant influences on photophysical properties in previous studies.[28–30] However, our results presented in this study show that the excited state of the new complexes **Ru-I** and **Ru-II** behave completely different compared to their respective reference complexes **[Ru(bpy)$_3$]$^{2+}$** and **Ru-III**, which, in combination with the MV^{2+} photoreduction studies presented above, can explain the altered and improved reactivity in the synthesis of trifluoromethylated indolizines.

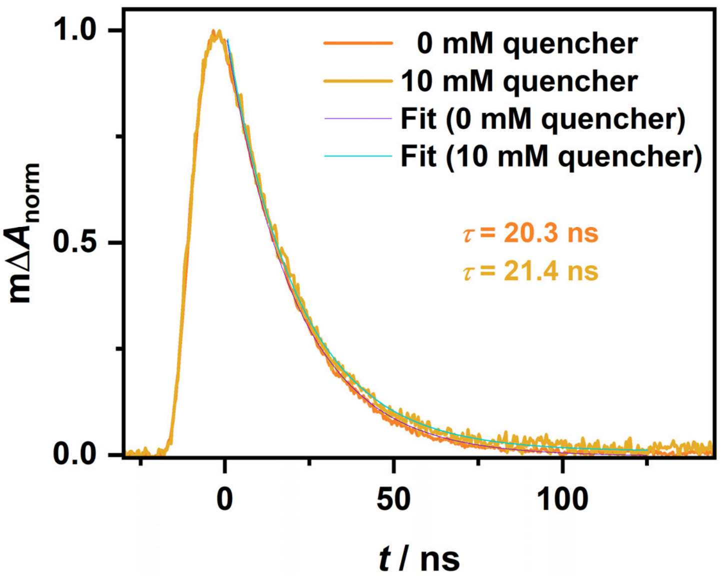

Figure S24 Time-resolved absorption traces of **Ru-I** (40 µM) in Ar-saturated DMF after laser excitation (λ_{exc} = 532 nm) at 710 nm without $TTCF_3OTf$ (orange) and with 10 mM $TTCF_3OTf$ (yellow).

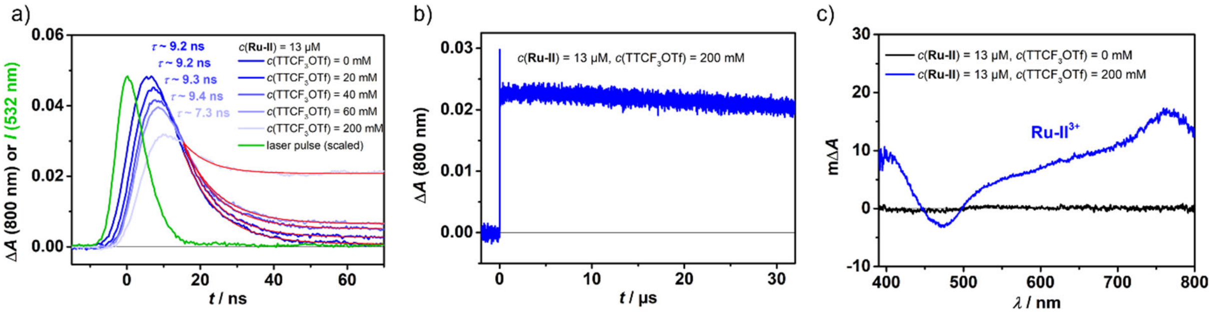

Figure S25 (a) Time-resolved detection of the laser pulse (scattered laser light of a water-containing cuvette, green) as well as time-resolved absorption traces of solutions containing 13 µM **Ru-II** and different concentrations of $TTCF_3OTf$ in Ar-saturated DMF after laser excitation (λ_{exc} = 532 nm, λ_{det} = 793–807 nm, blue) along with exponential fitting functions after the laser pulse for lifetime estimation. (b) Time-resolved absorption trace of a solution containing 13 µM **Ru-II** and 200 mM $TTCF_3OTf$ in Ar-saturated DMF after laser excitation (λ_{exc} = 532 nm, λ_{det} = 793–807 nm). (c) Transient absorption spectra of solutions containing 13 µM **Ru-II** or 13 µM **Ru-II** and 200 mM $TTCF_3OTf$ after laser excitation (λ_{exc} = 532 nm, delay 30 µs).

As in the case of ^{3}**Ru-I** (see **Figure S24**), the lifetime of ^{3}**Ru-II** is initially unchanged or slightly increases with increasing concentration of $TTCF_3OTf$ ($c(TTCF_3OTf)$ = 0 mM – 60 mM, see **Figure S25** a). However, after the decay of ^{3}**Ru-II**, an increase in the residual transient absorption can be observed,

which can be clearly assigned to the post-quenching product **Ru-II**$^{3+}$ by means of the transient absorption spectrum (see **Figure S25** c, compare to **Figure 5** (right) in the main part).

10. NMR spectra

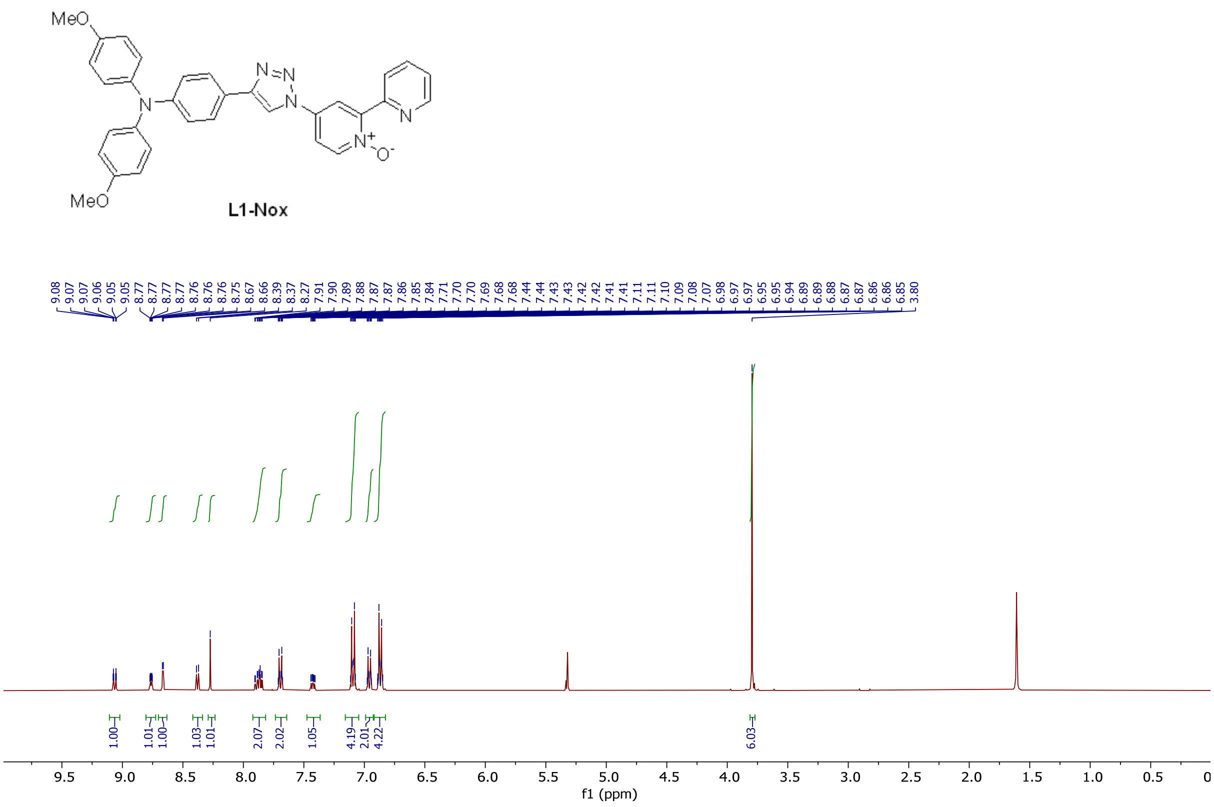

^{1}H NMR (600 MHz, CD_2Cl_2) of **L1-Nox**.

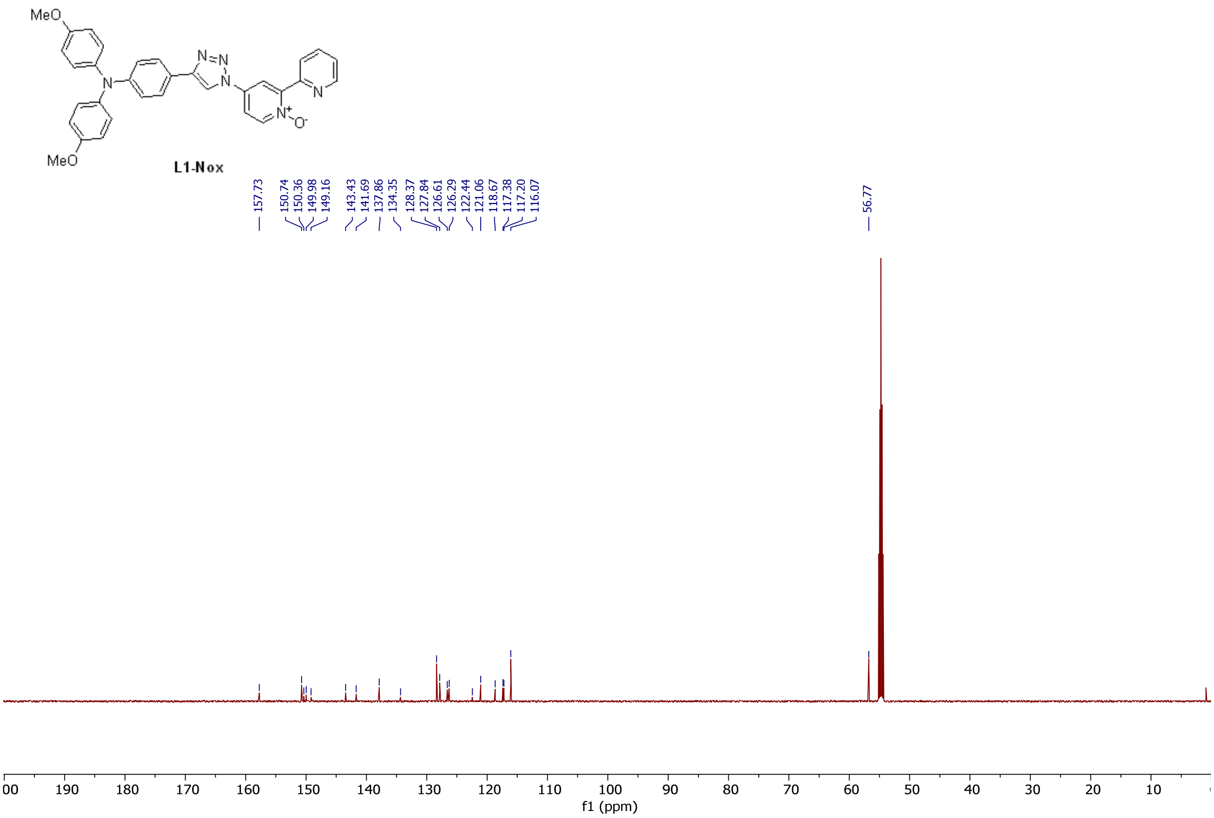

$^{13}C\{^{1}H\}$ NMR (125 MHz, CD_2Cl_2) of **L1-Nox**.

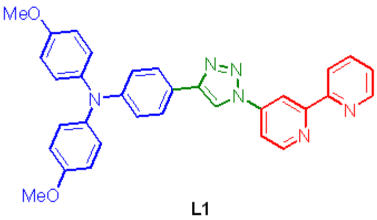

L1

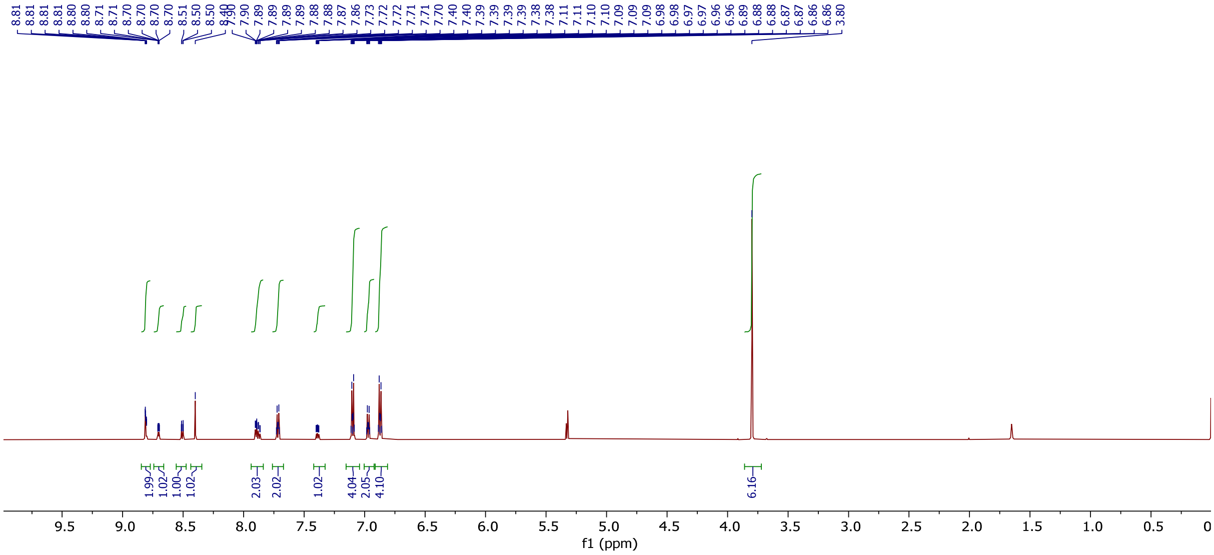

^{1}H NMR (600 MHz, CD_2Cl_2) of **L1**.

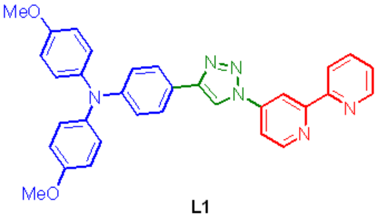

L1

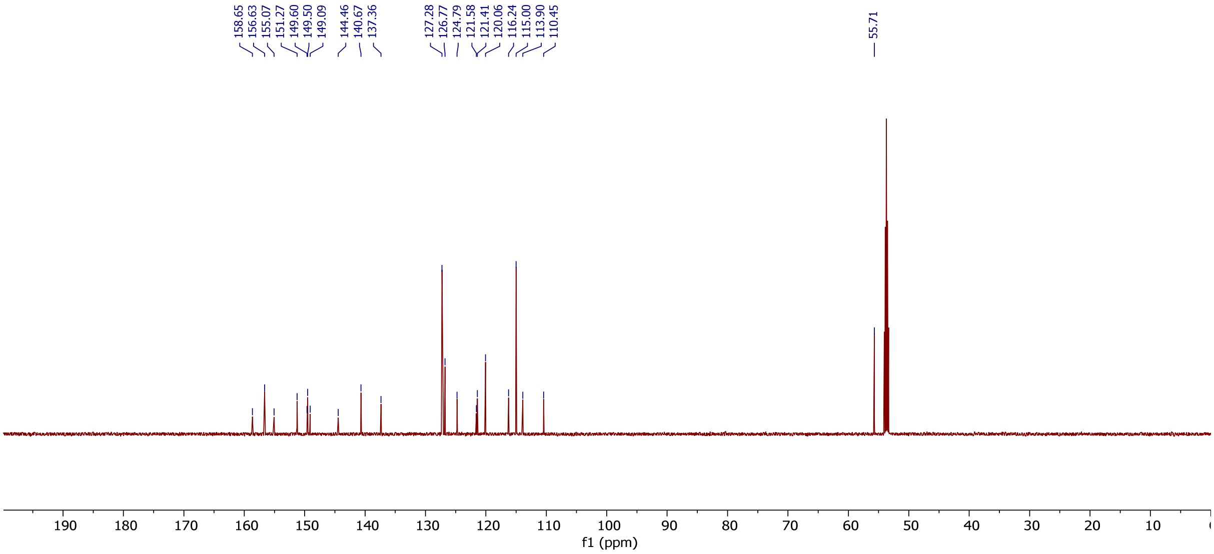

$^{13}C\{^{1}H\}$ NMR (125 MHz, CD_2Cl_2) of **L1**.

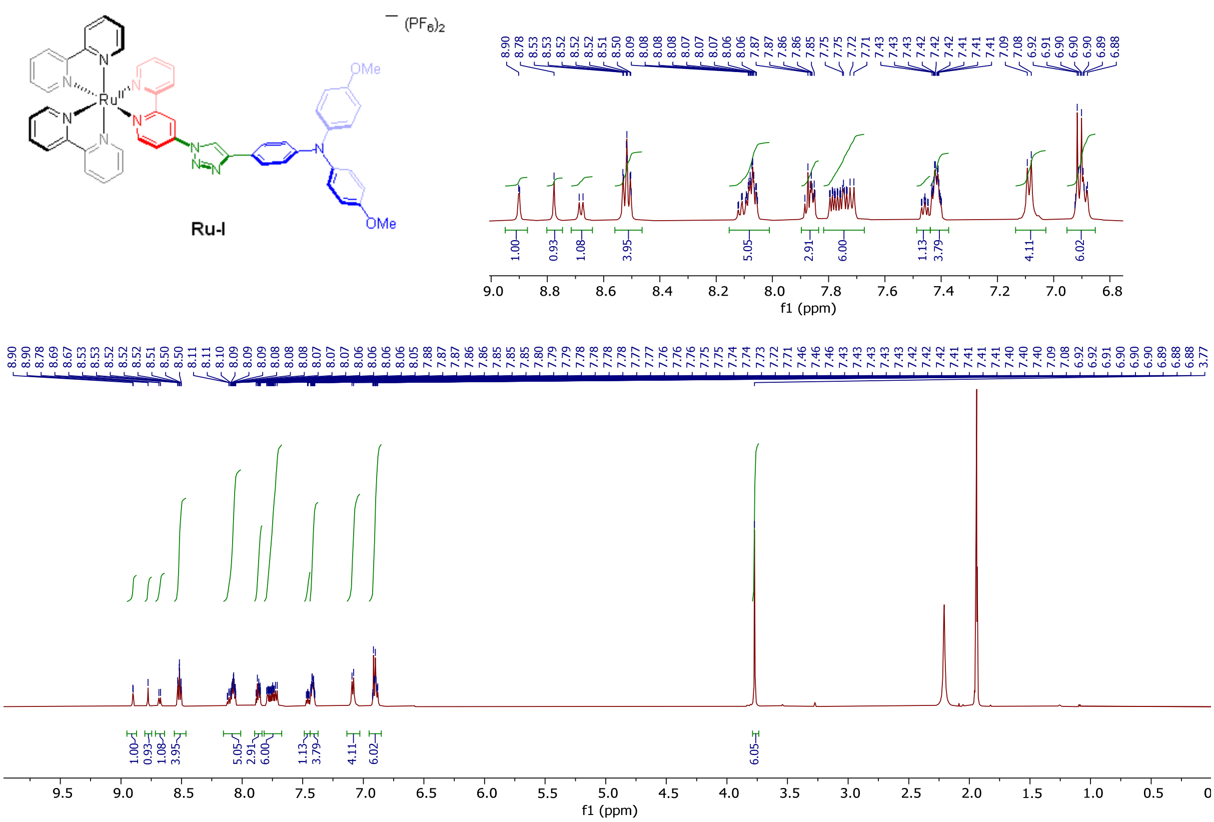

^{1}H NMR (600 MHz, CD_3CN) of **Ru-I**.

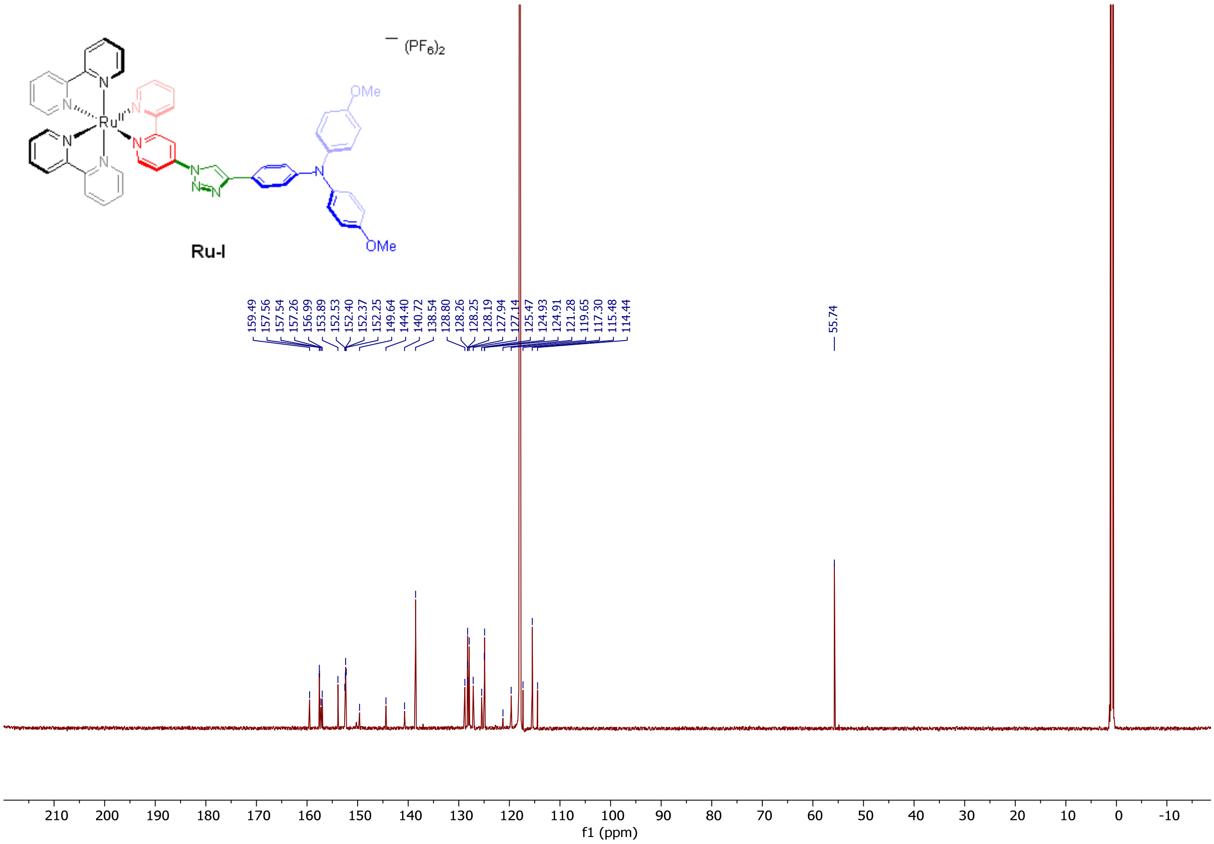

$^{13}C\{^1H\}$ NMR (125 MHz, CD_3CN) of **Ru-I**.

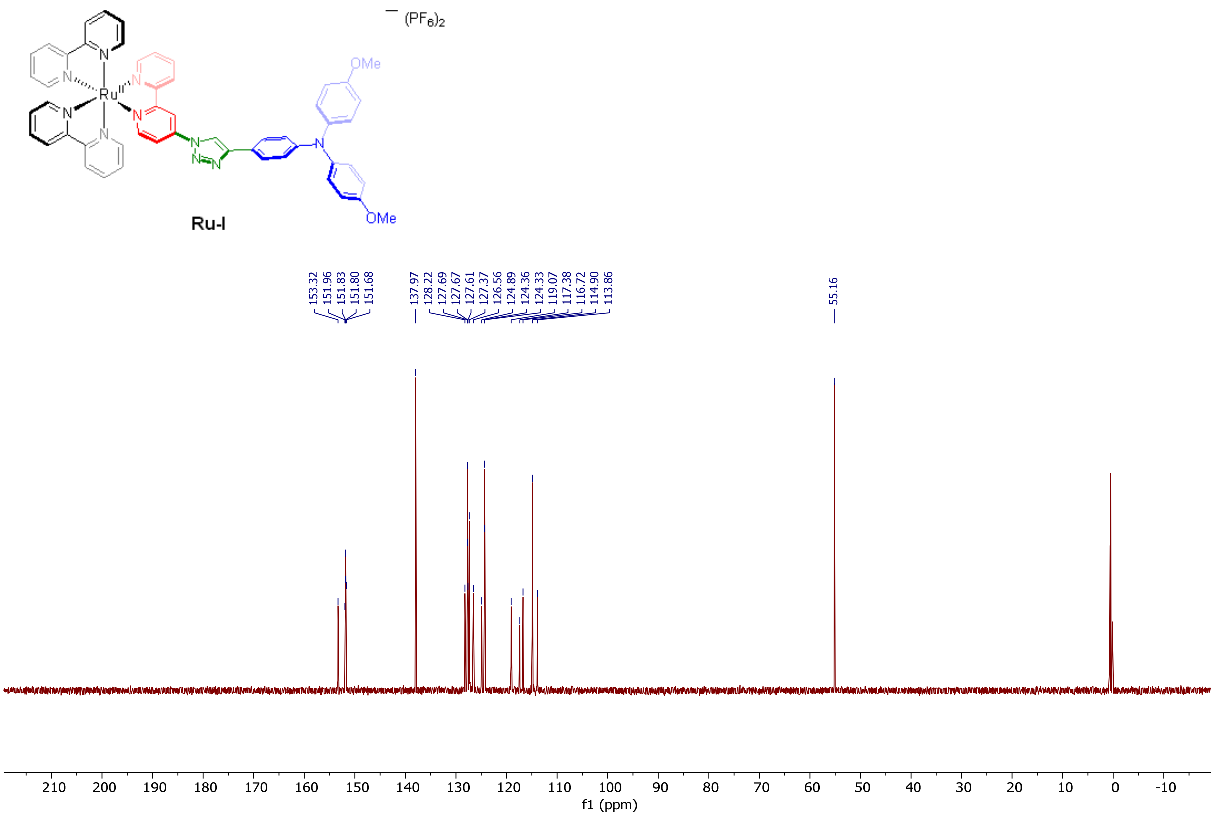

^{13}C-DEPT135 NMR (150 MHz, CD_3CN) of **Ru-I**.

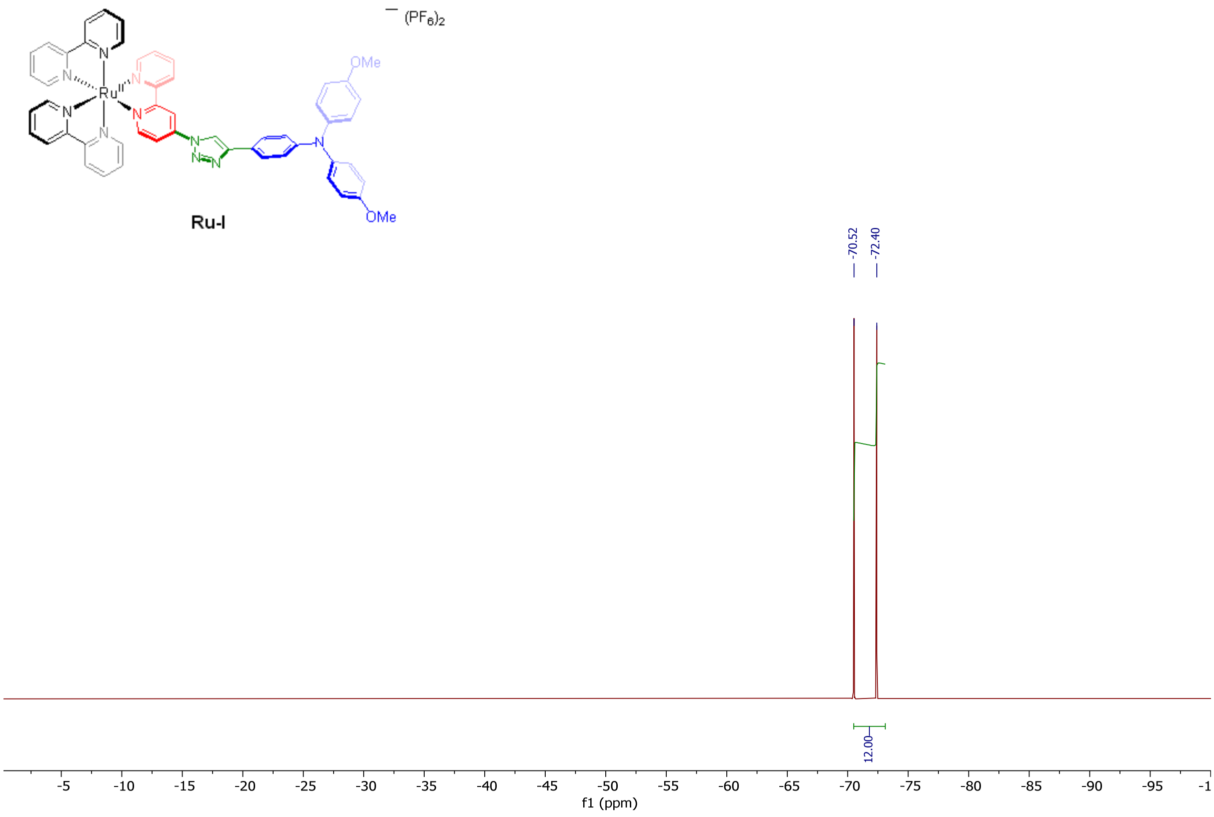

$^{19}F\{^1H\}$ NMR (377 MHz, CD_3CN) of **Ru-I**.

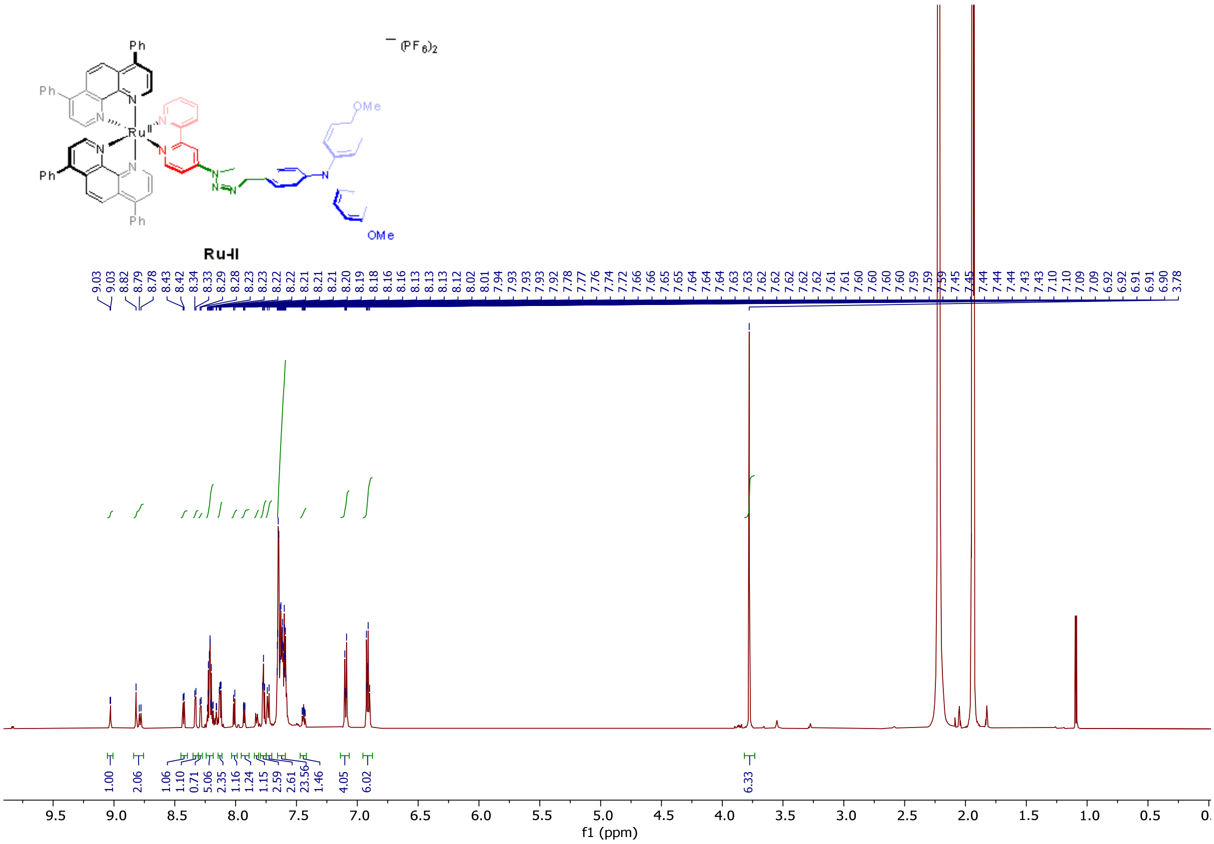

^1H NMR (600 MHz, CD_3CN) of **Ru-II**.

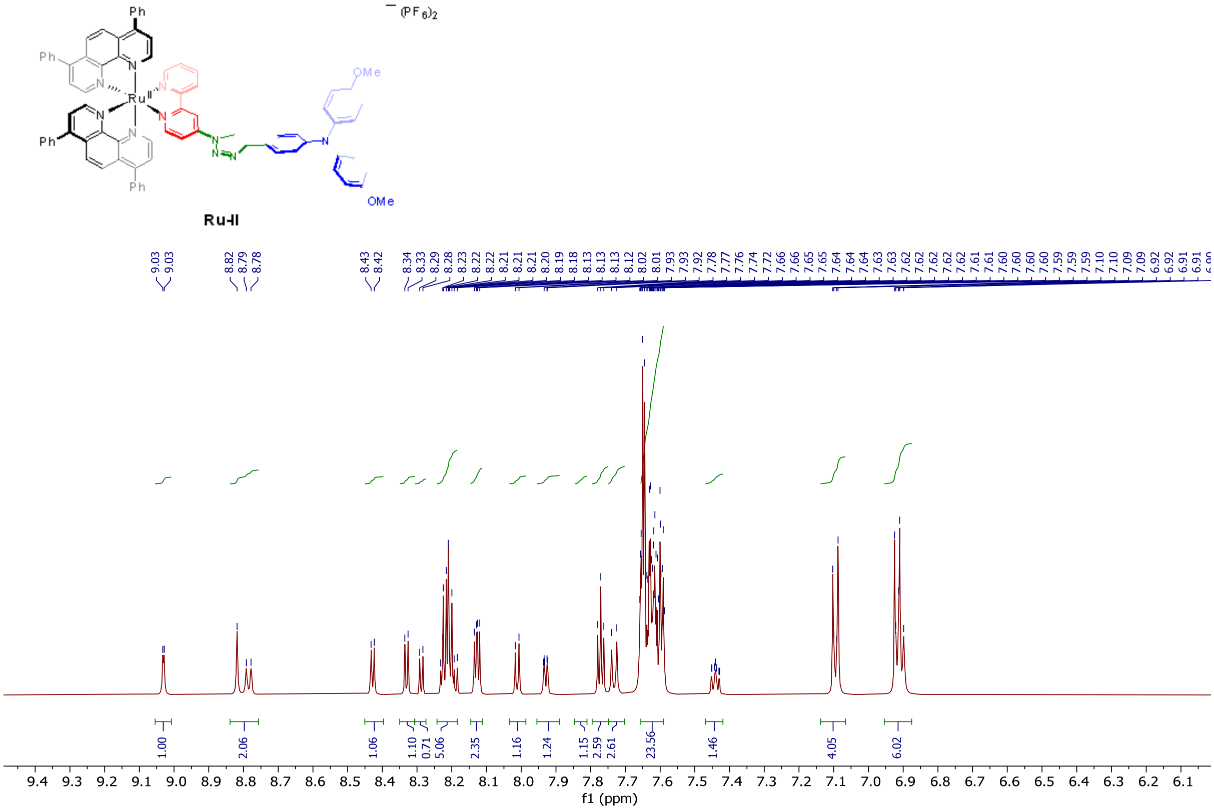

^{1}H NMR (600 MHz, CD_3CN) of **Ru-II** (expansion aromatic area 6.0 – 9.5 ppm).

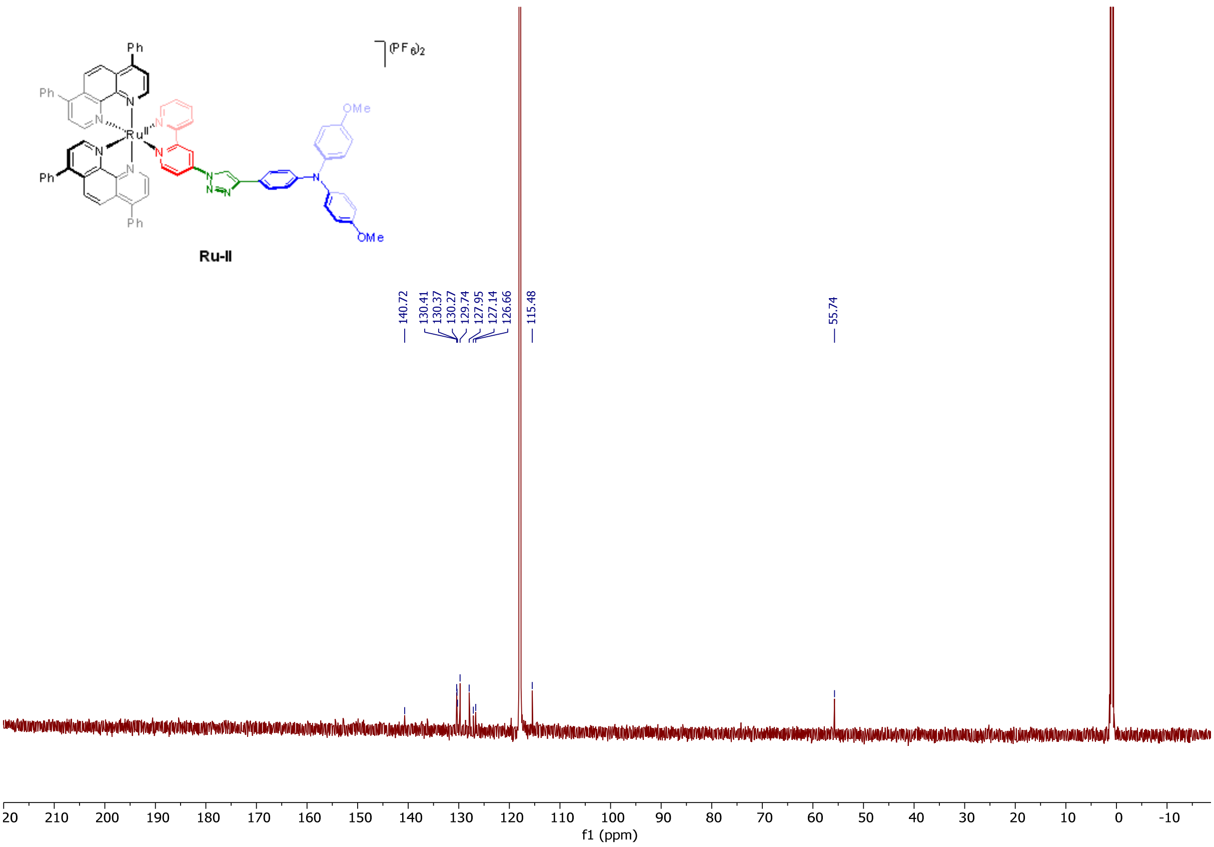

^{13}C{^{1}H} NMR (150 MHz, CD_3CN) of **Ru-II**.

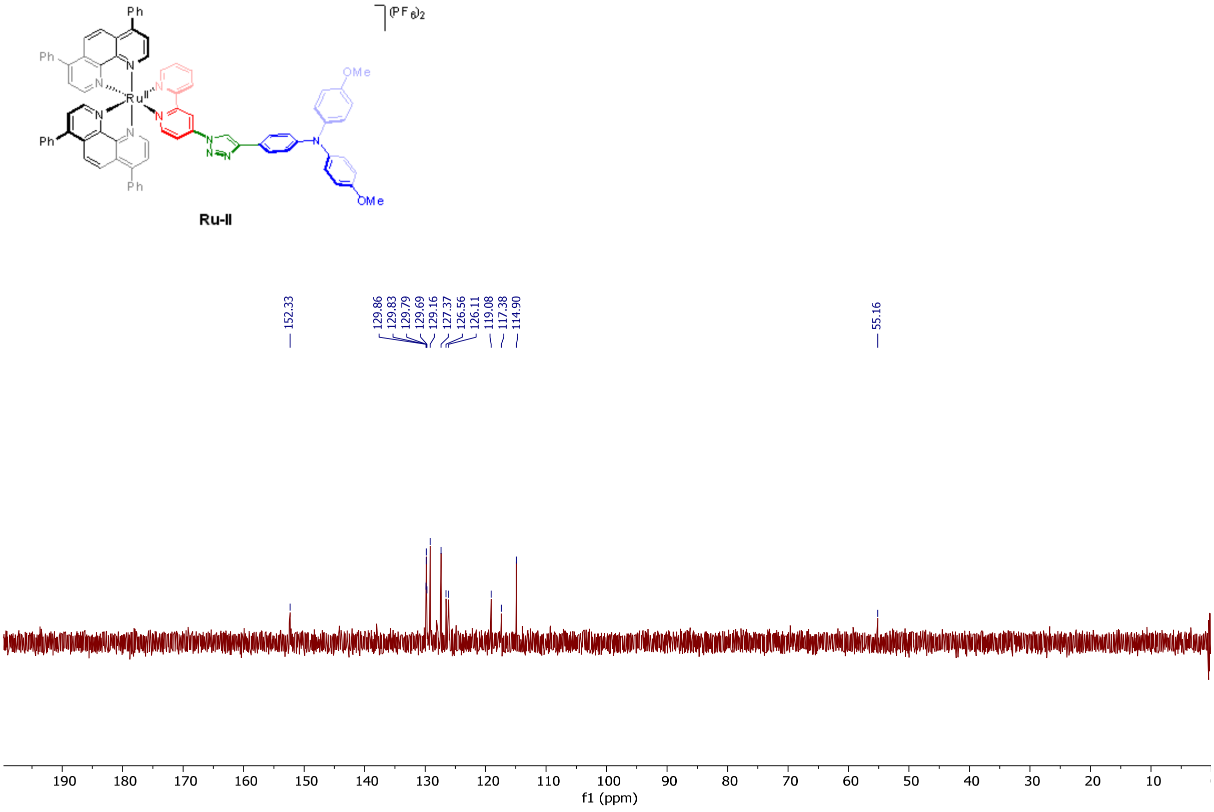

^{13}C-DEPT135 NMR (150 MHz, CD_3CN) of **Ru-II**.

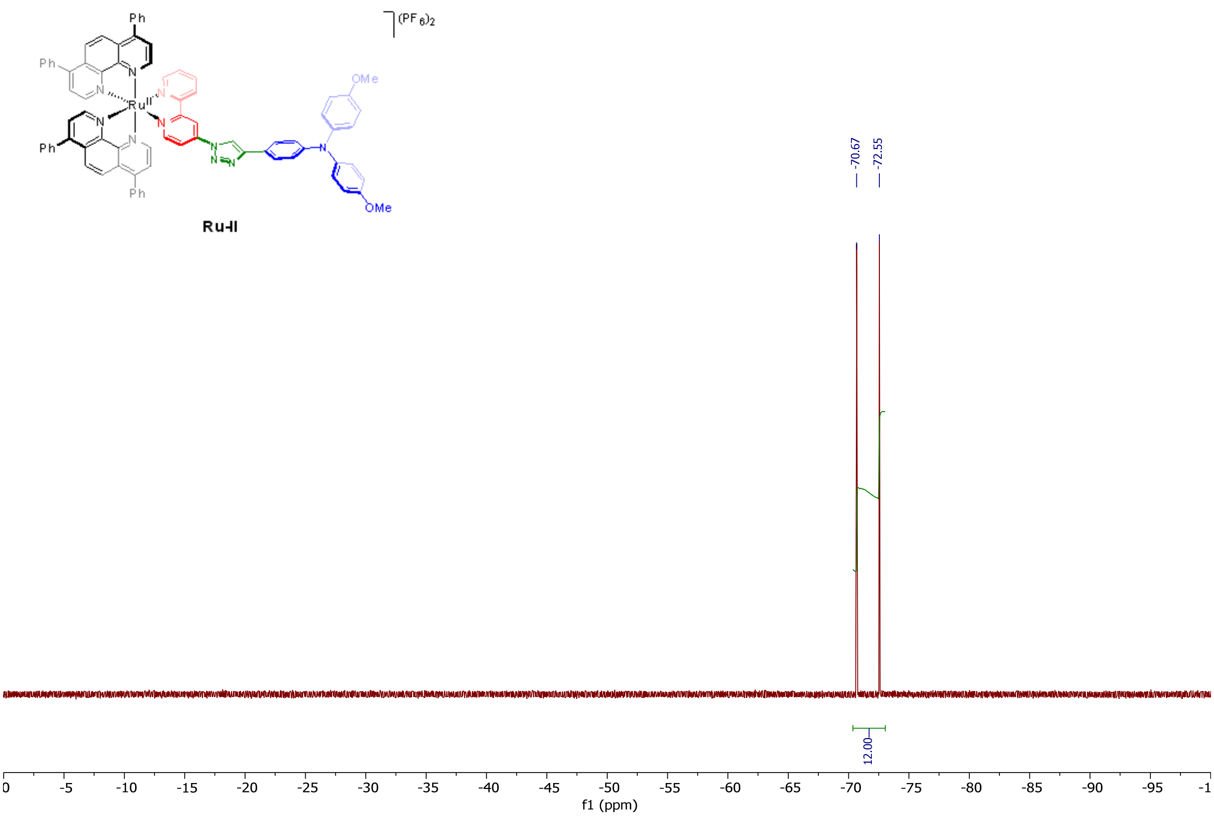

^{19}F{^{1}H} NMR (377 MHz, CD_3CN) of **Ru-II**.

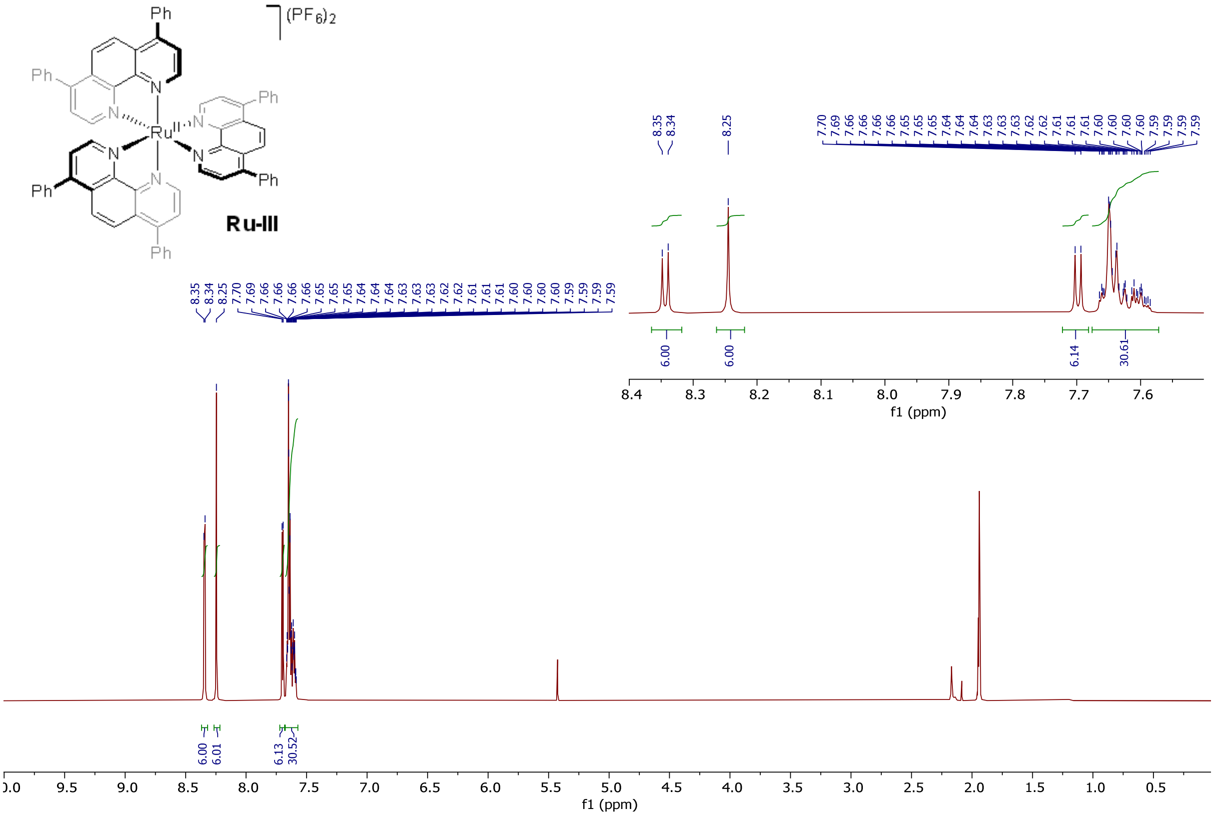

^{1}H NMR (600 MHz, CD_3CN) of **Ru-III**.

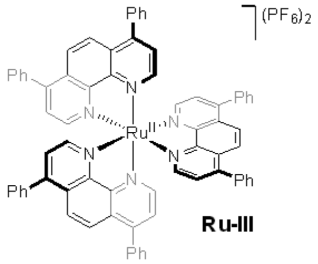

Ru-III

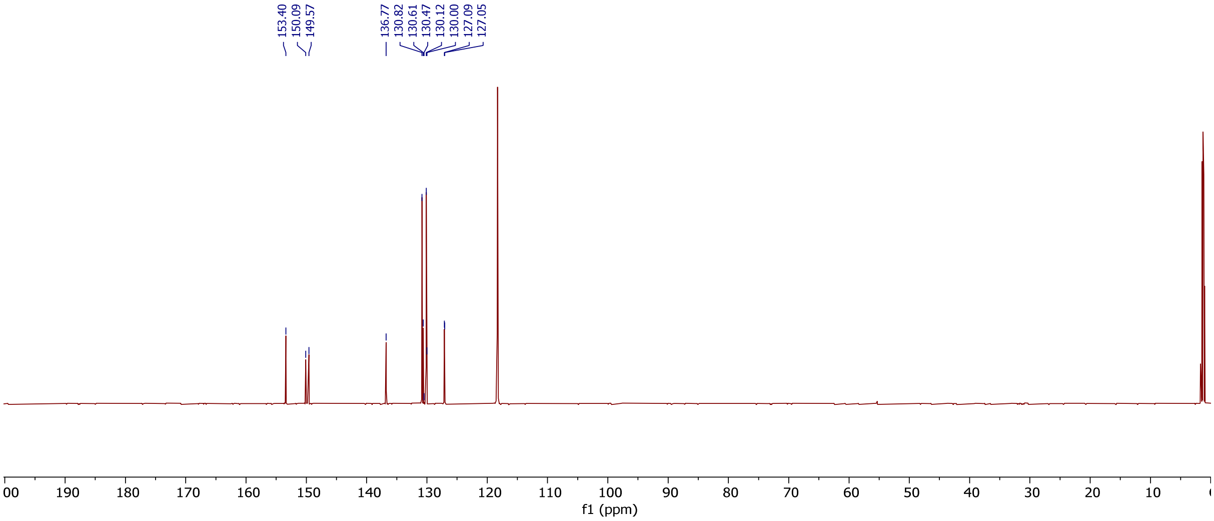

$^{13}C\{^{1}H\}$ NMR (150 MHz, CD_3CN) of **Ru-III**.

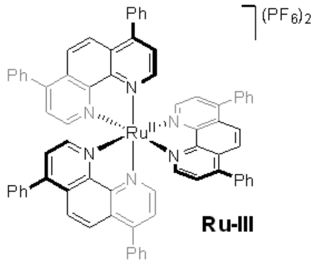

Ru-III

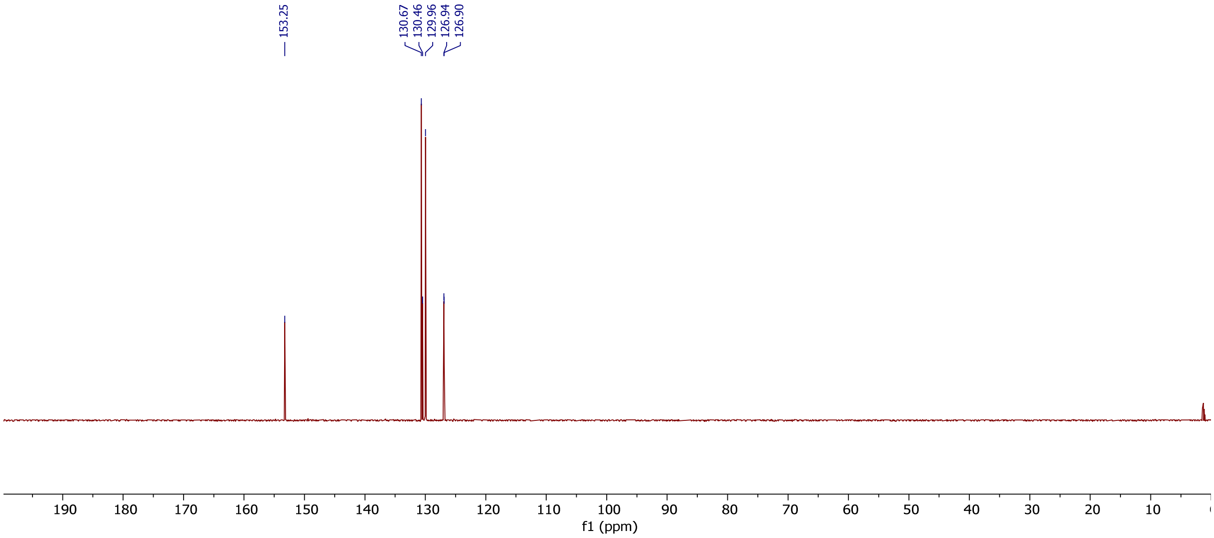

^{13}C-DEPT135 NMR (150 MHz, CD_3CN) of **Ru-III**.

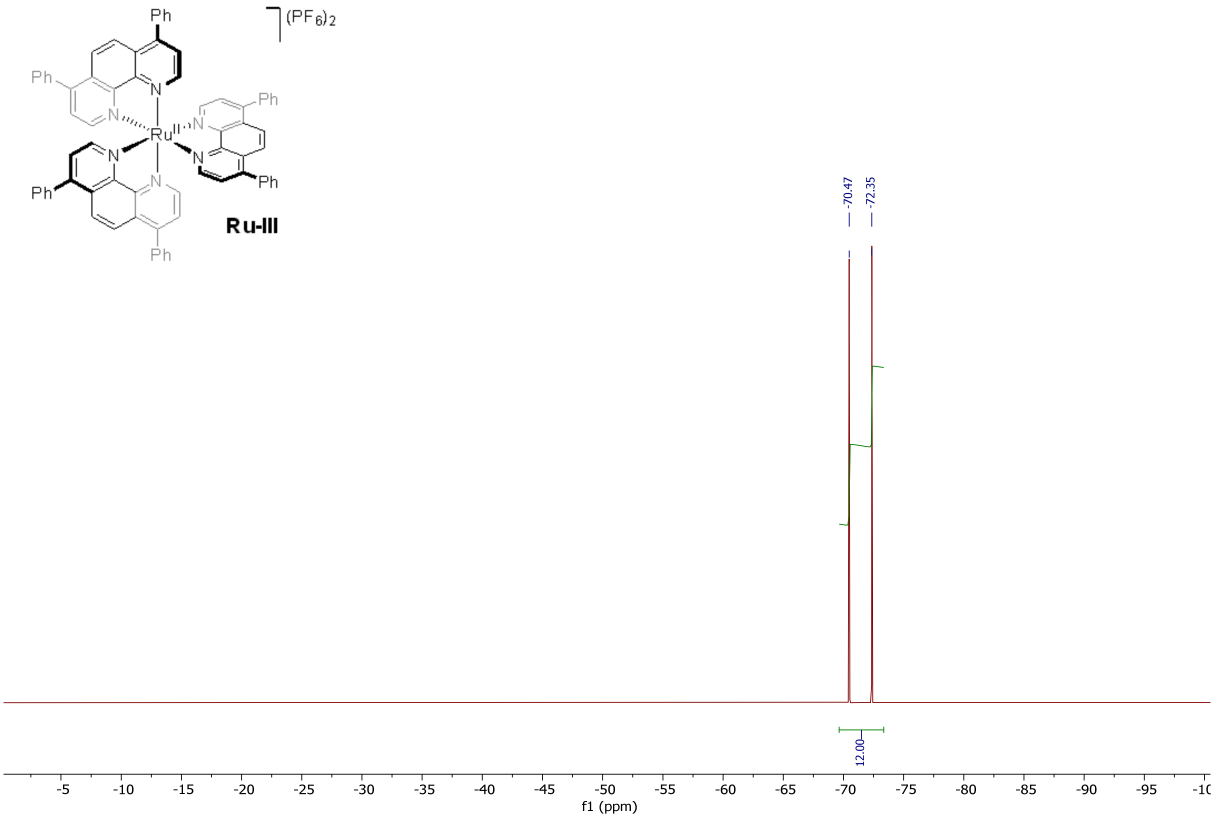

$^{19}F\{^{1}H\}$ NMR (377 MHz, CD_3CN) of **Ru-III**.

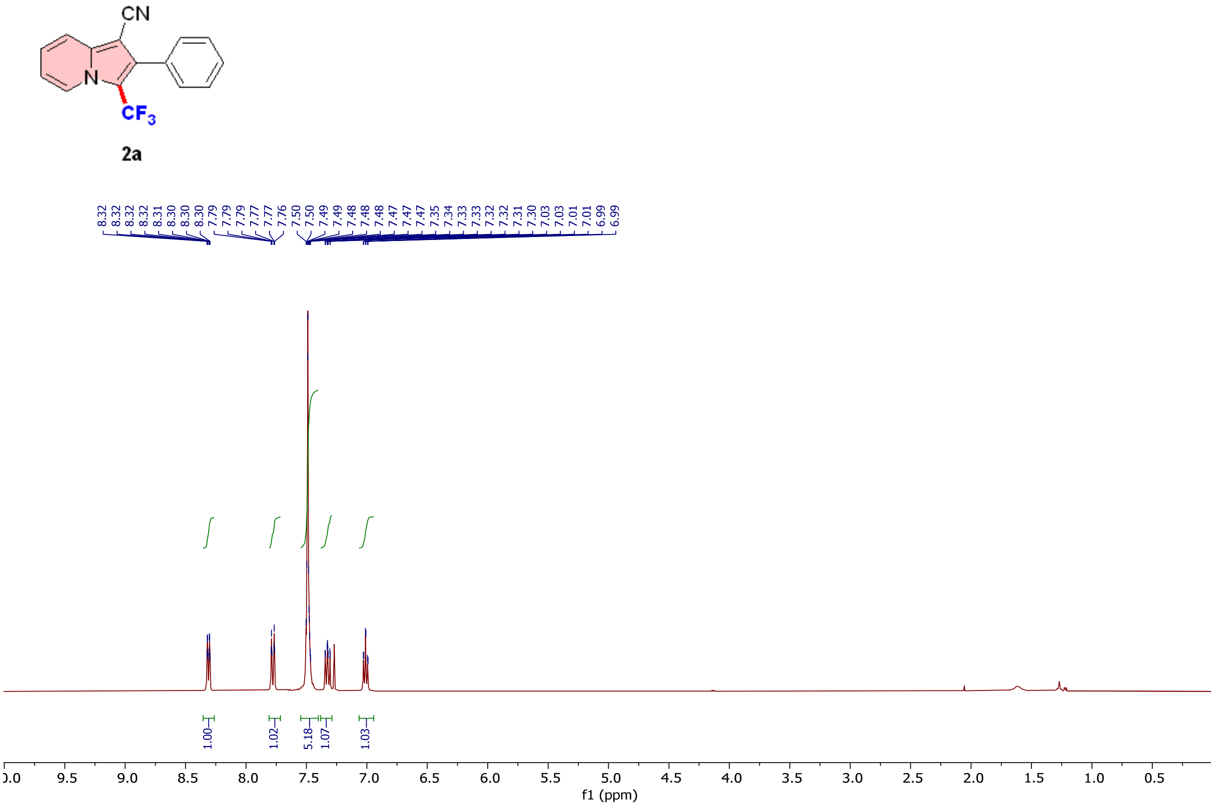

^{1}H NMR (400 MHz, $CDCl_3$) of **2a**.

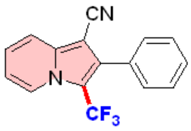

2a

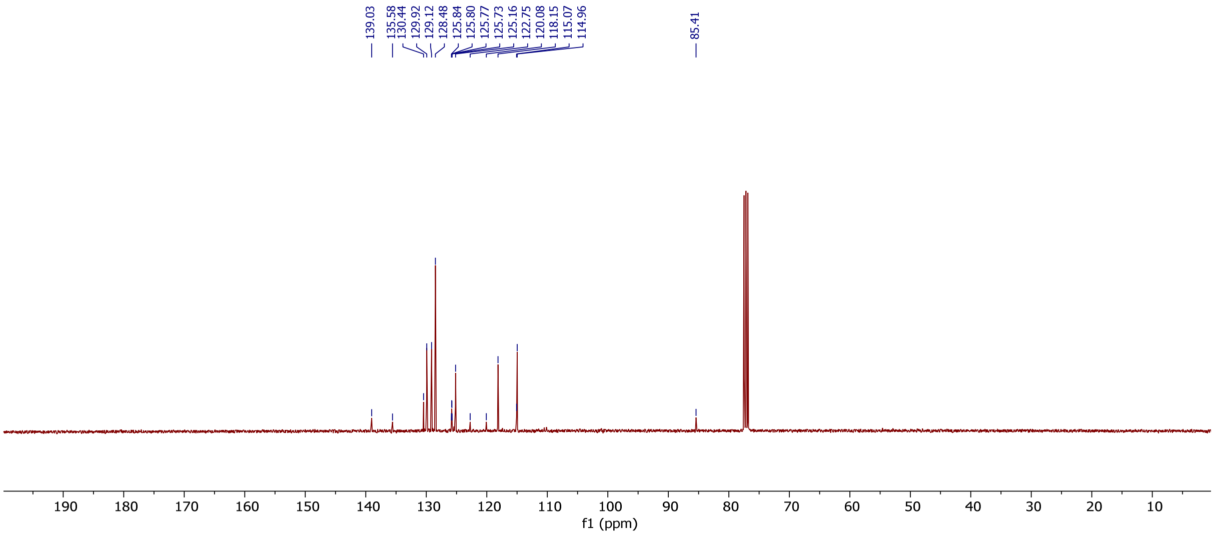

$^{13}C\{^1H\}$ NMR (100 MHz, $CDCl_3$) of **2a**.

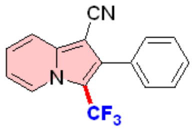

2a

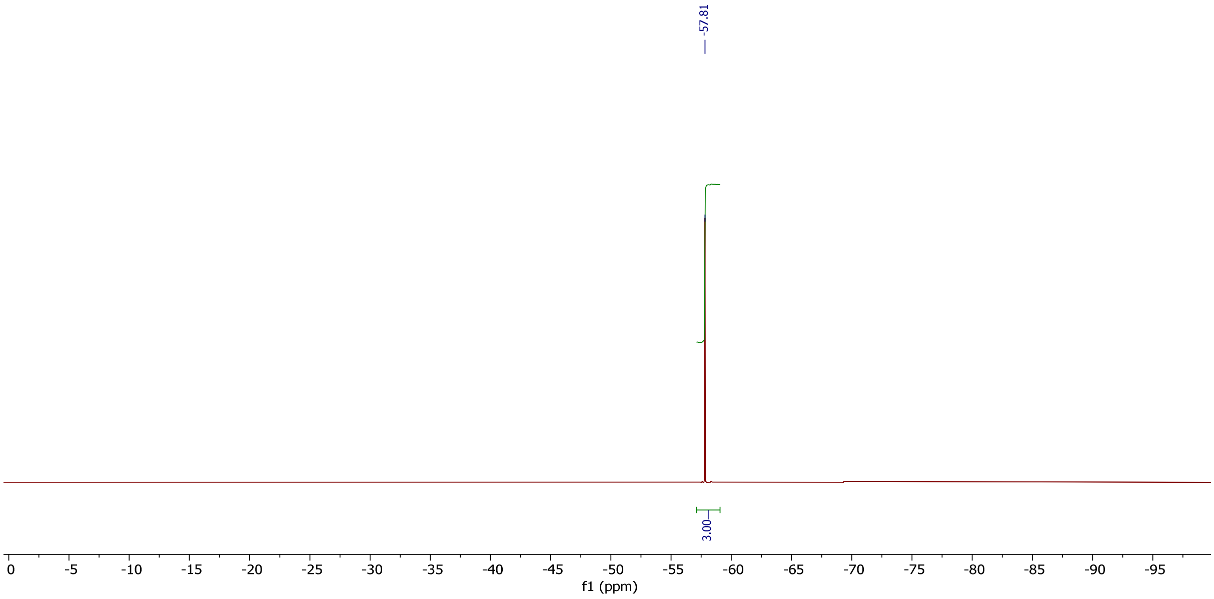

$^{19}F\{^1H\}$ NMR (377 MHz, $CDCl_3$) of **2a**.

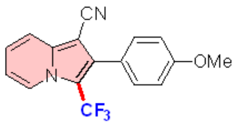

2c

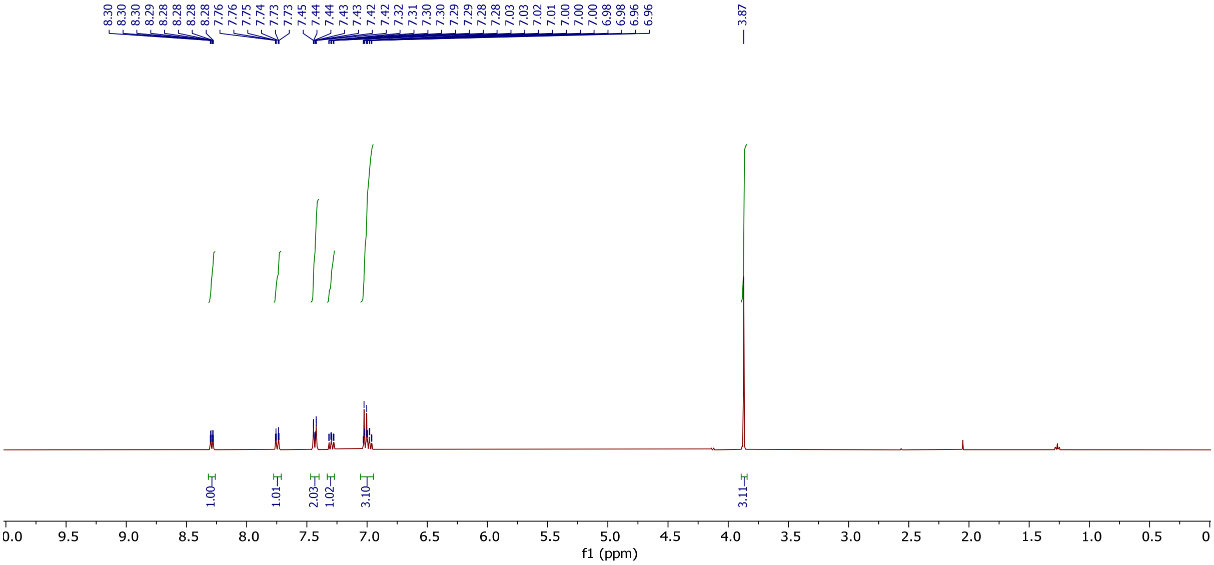

^{1}H NMR (400 MHz, $CDCl_3$) of **2c**.

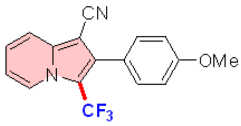

2c

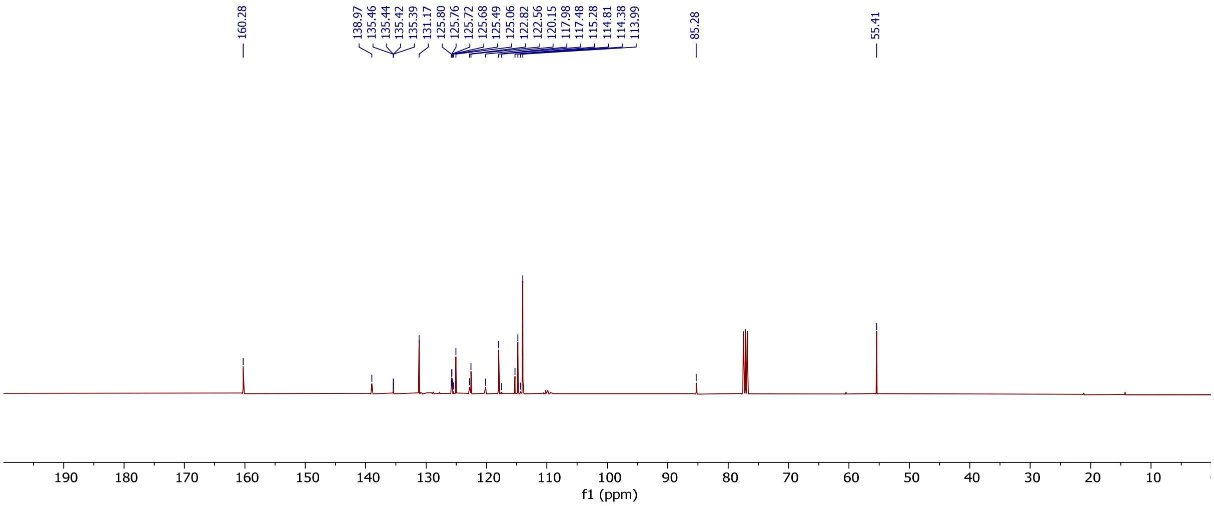

^{13}C{^{1}H} NMR (100 MHz, $CDCl_3$) of **2c**.

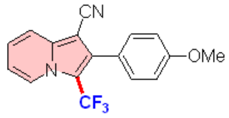

2c

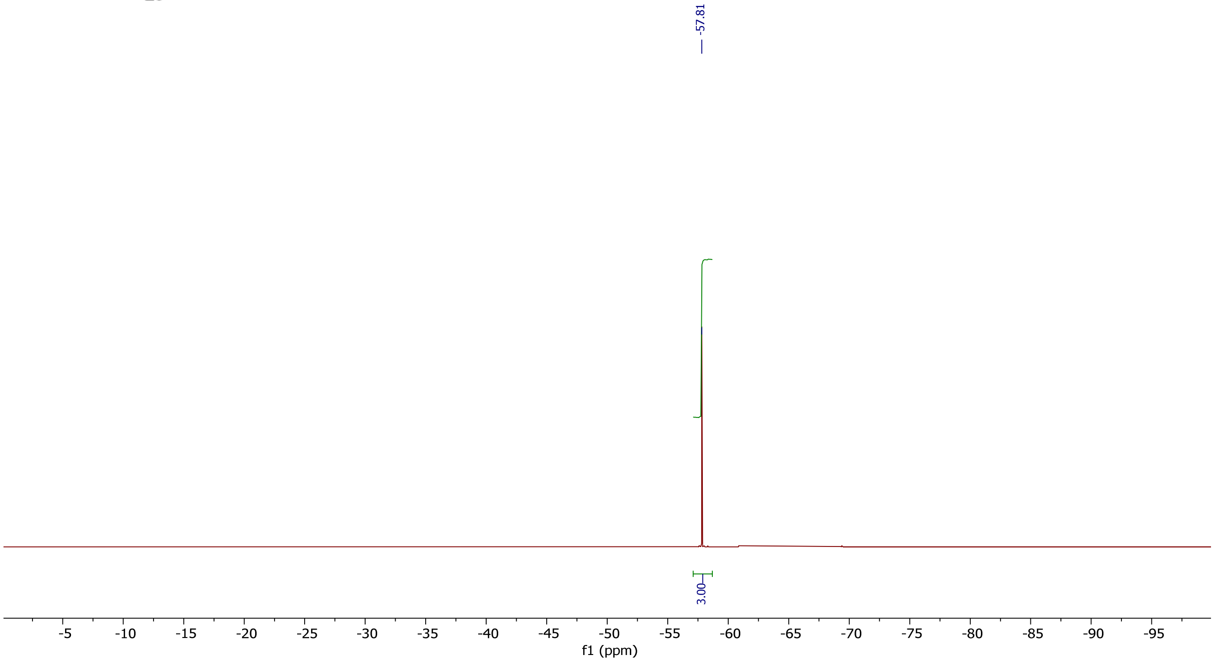

$^{19}F\{^{1}H\}$ NMR (377 MHz, $CDCl_3$) of **2c**.

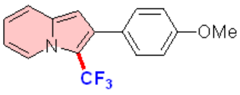

2d

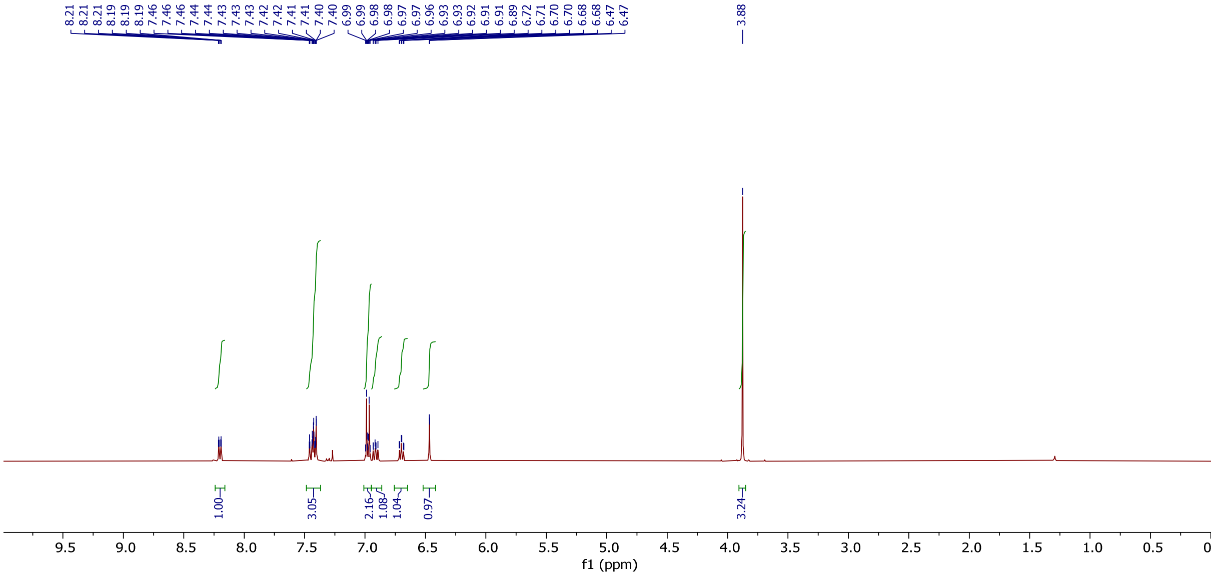

^{1}H NMR (400 MHz, $CDCl_3$) of **2d**.

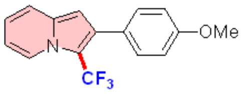

2d

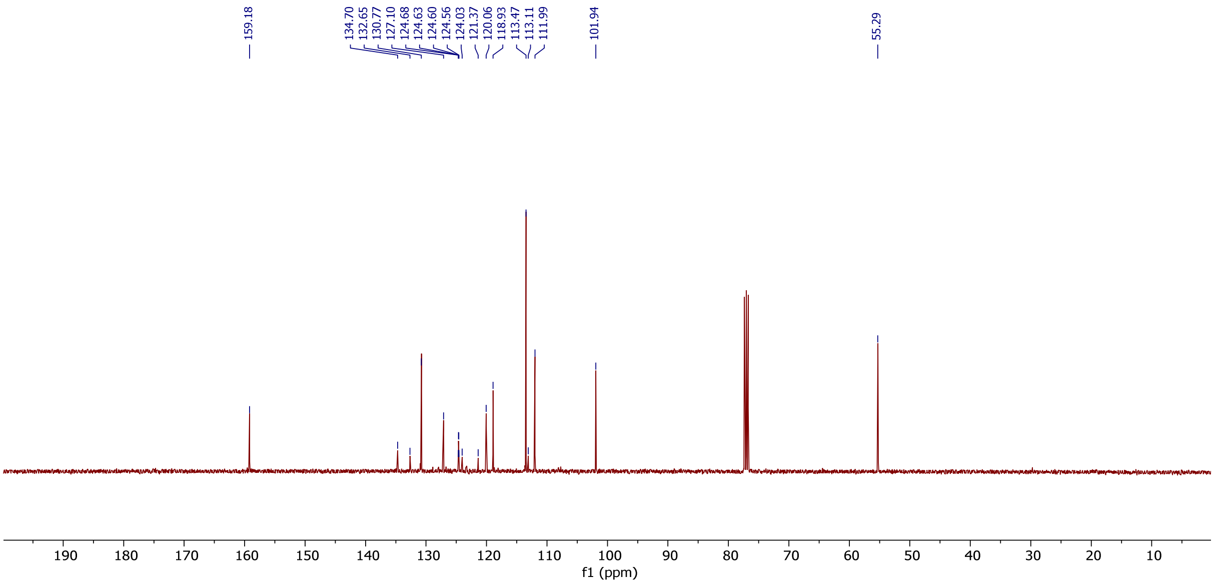

$^{13}C\{^{1}H\}$ NMR (100 MHz, $CDCl_3$) of **2d**.

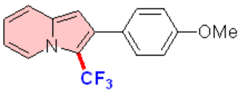

2d

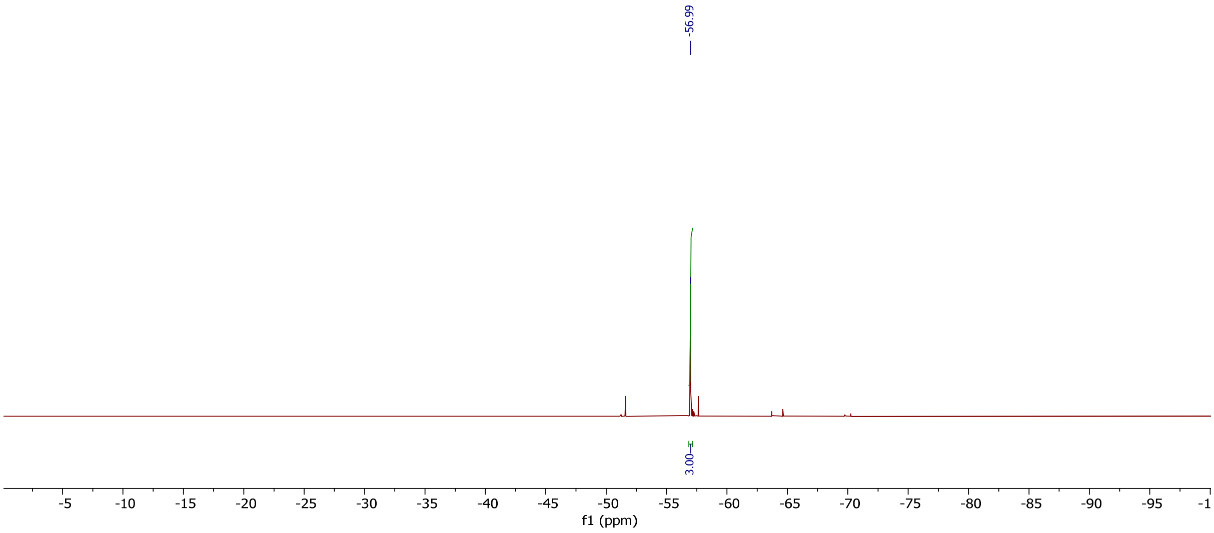

$^{19}F\{^{1}H\}$ NMR (377 MHz, $CDCl_3$) of **2d**.

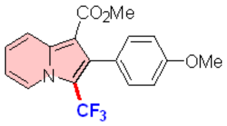

2e

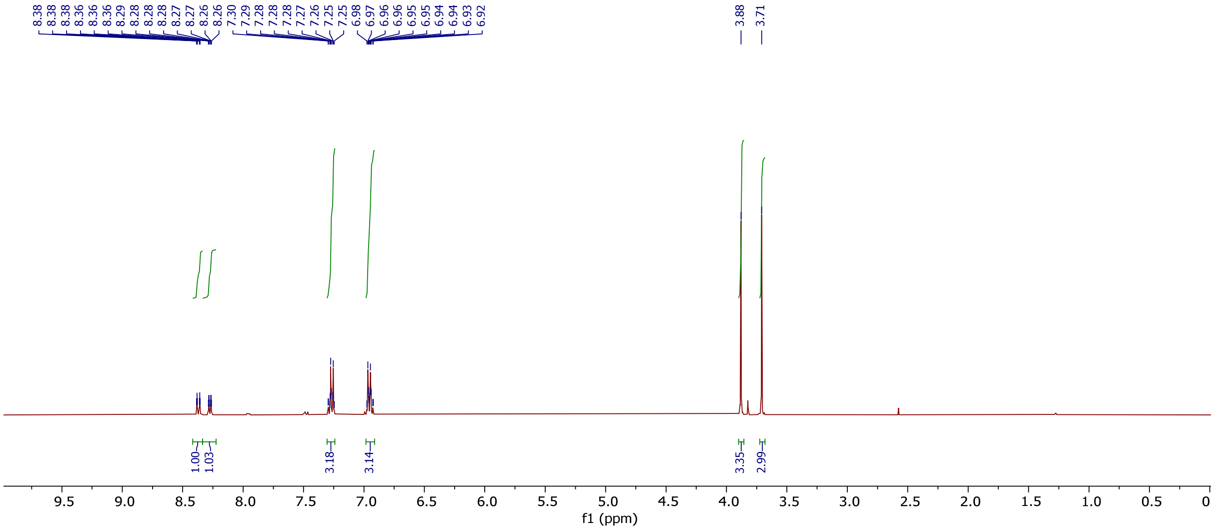

^{1}H NMR (400 MHz, $CDCl_3$) of **2e**.

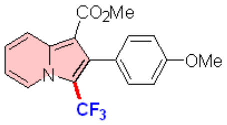

2e

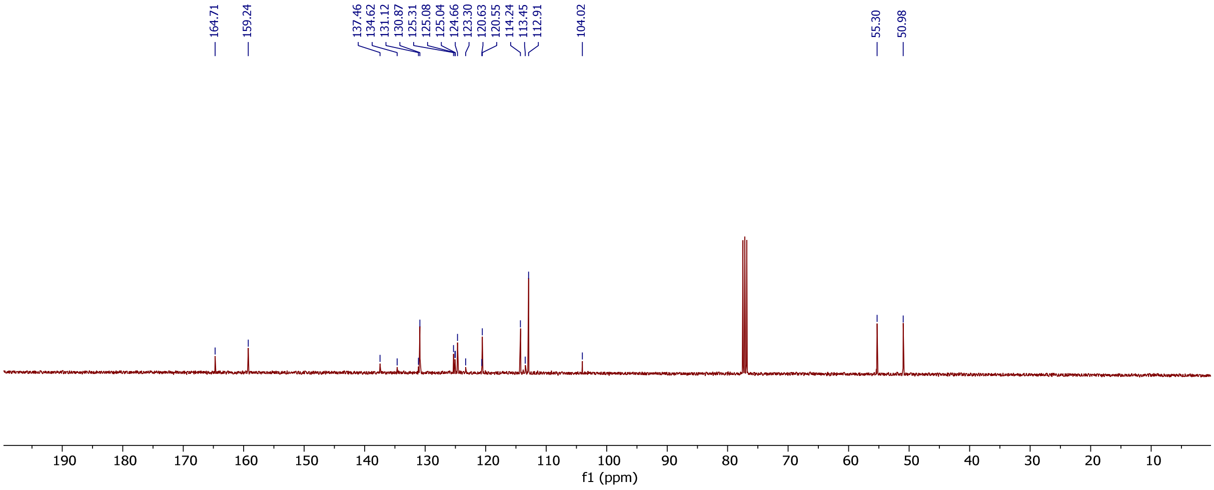

$^{13}C\{^1H\}$ NMR (100 MHz, $CDCl_3$) of **2e**.

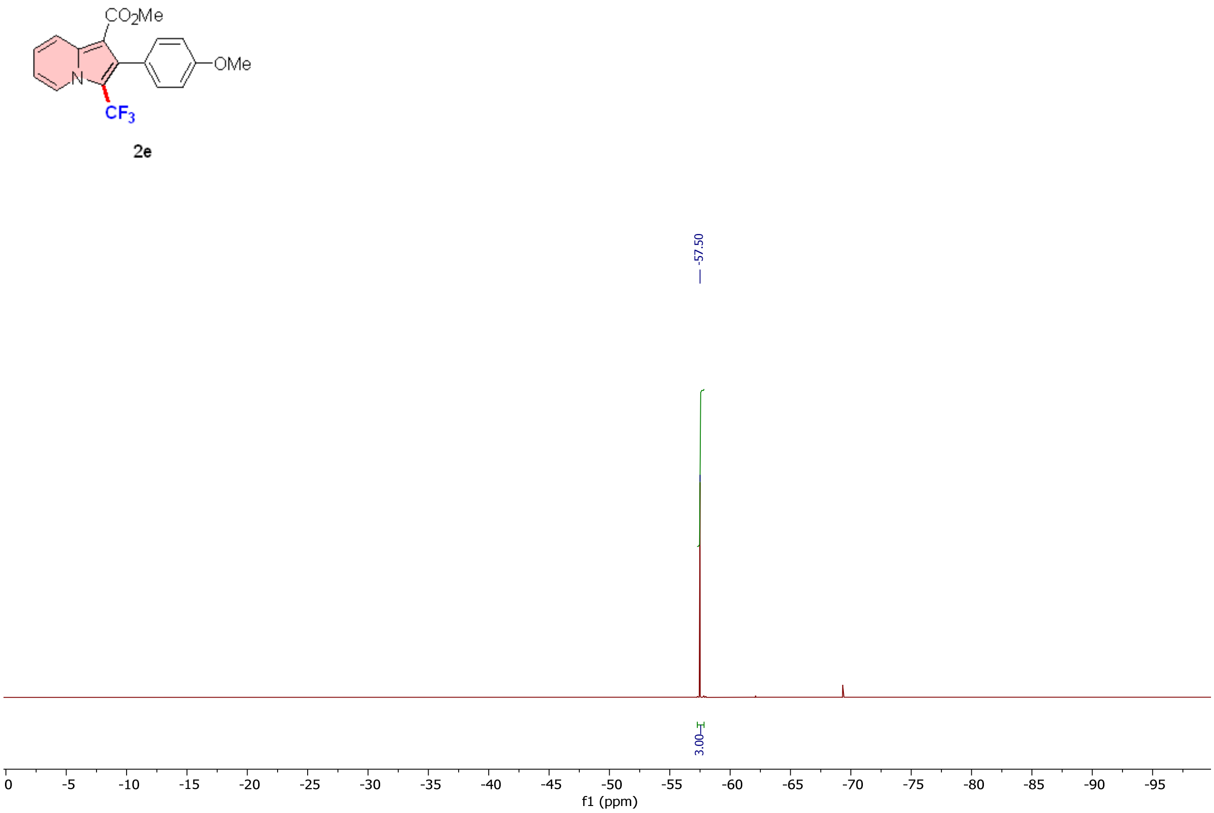

$^{19}F\{^1H\}$ NMR (377 MHz, $CDCl_3$) of **2e**.

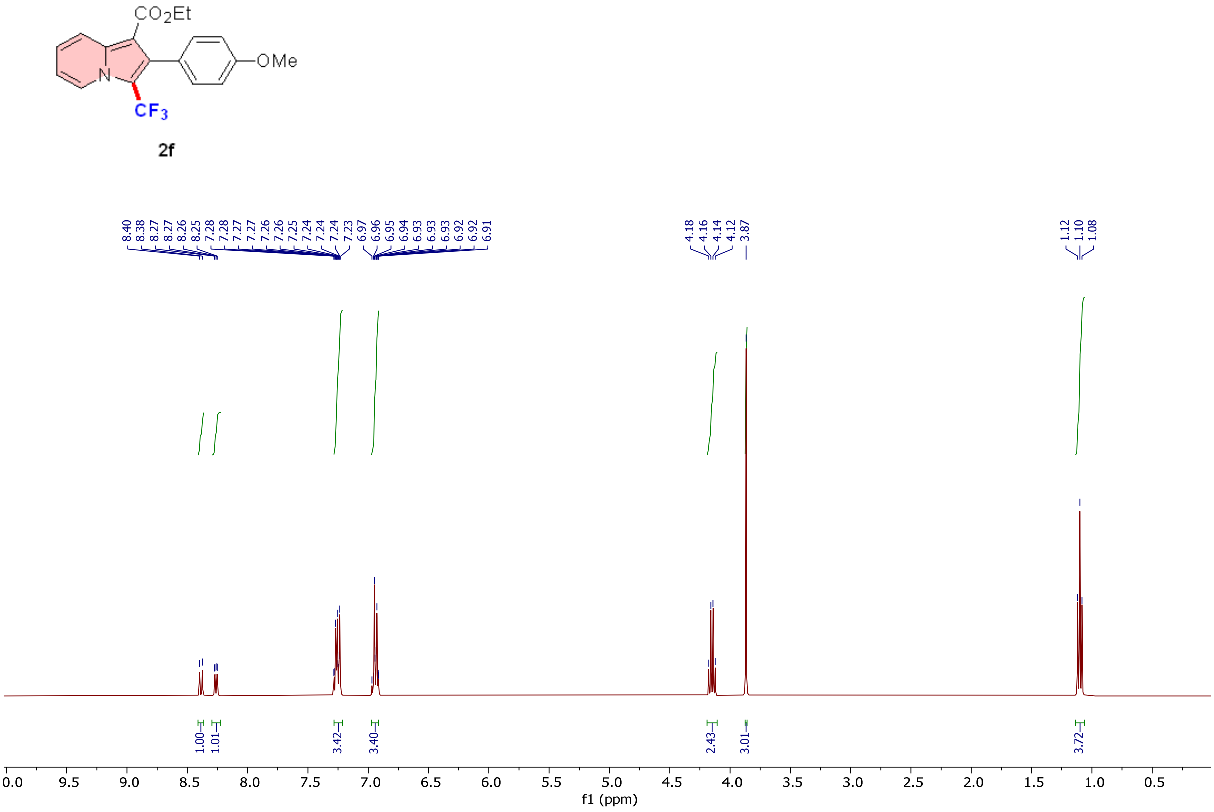

^1H NMR (400 MHz, $CDCl_3$) of **2f**.

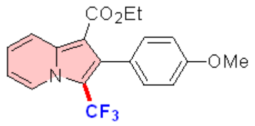

2f

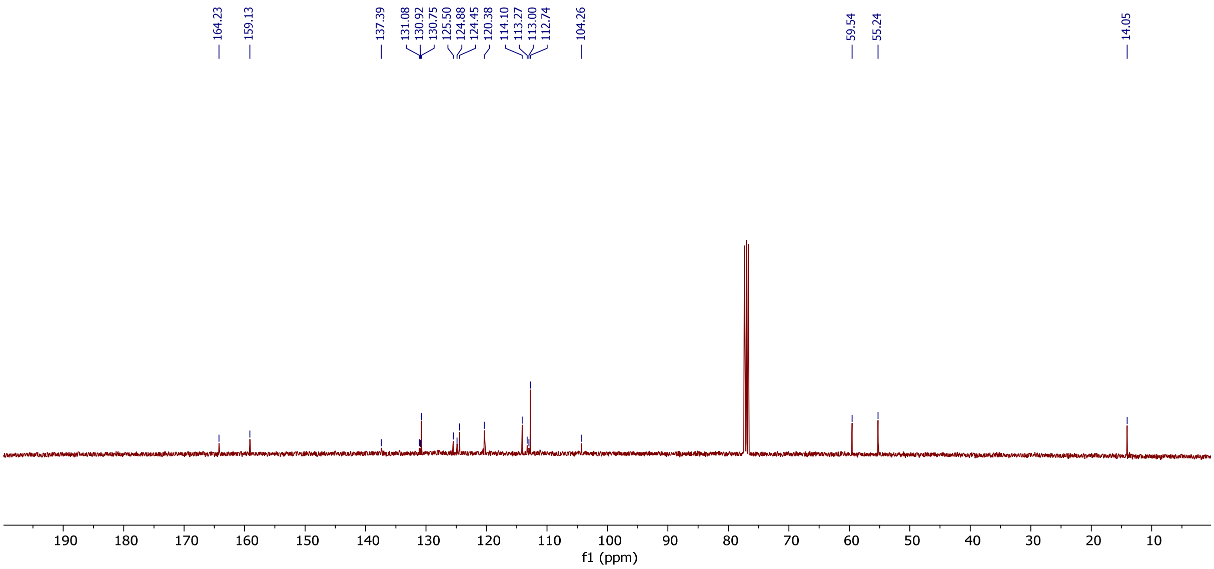

$^{13}C\{^{1}H\}$ NMR (100 MHz, $CDCl_3$) of **2f**.

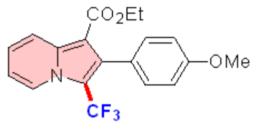

2f

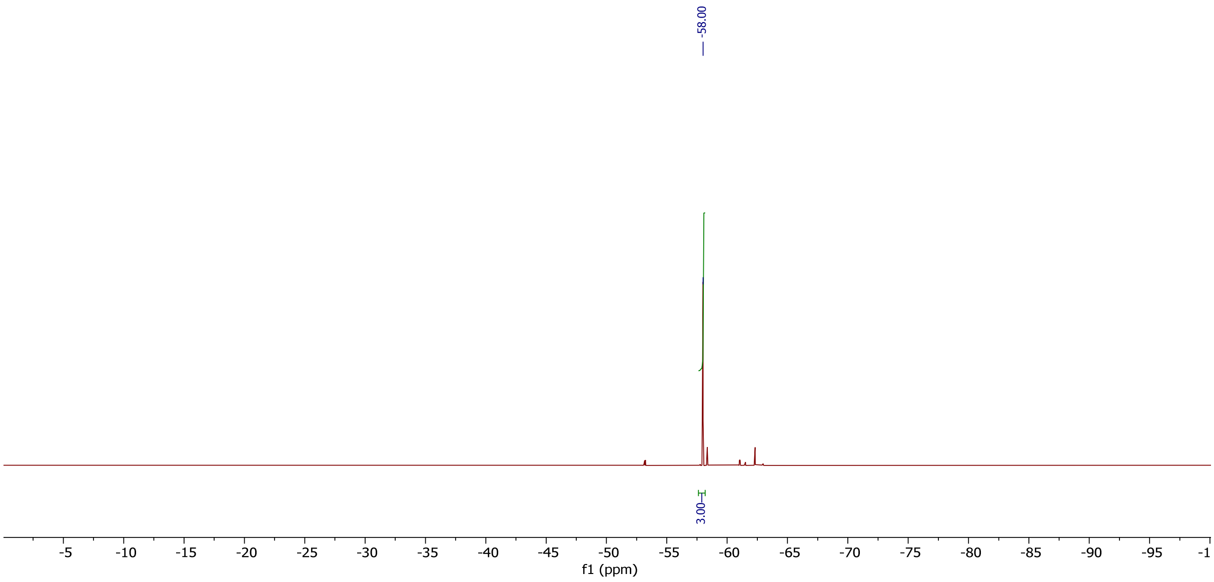

$^{19}F\{^{1}H\}$ NMR (377 MHz, $CDCl_3$) of **2f**.

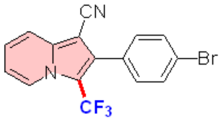

2g

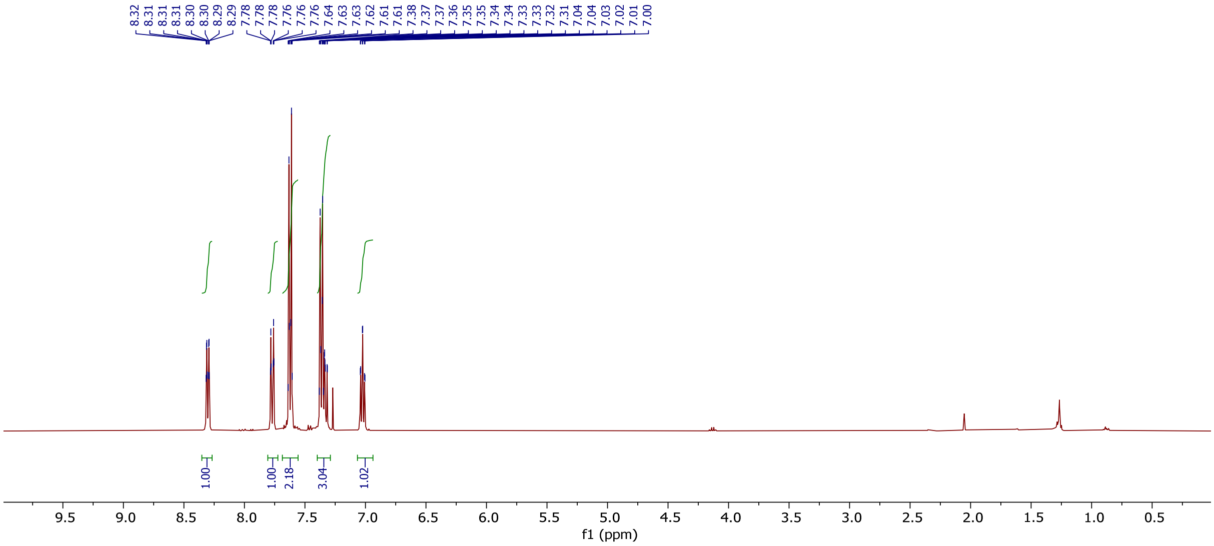

^{1}H NMR (400 MHz, $CDCl_3$) of **2g**.

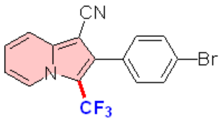

2g

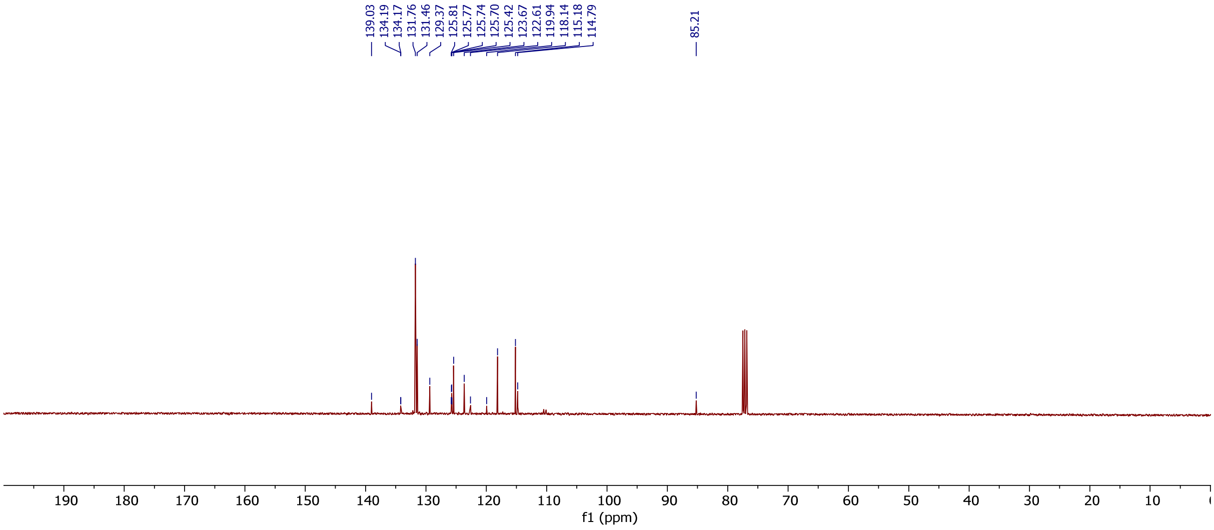

^{13}C{^{1}H} NMR (100 MHz, $CDCl_3$) of **2g**.

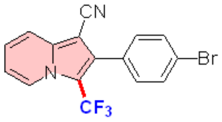

2g

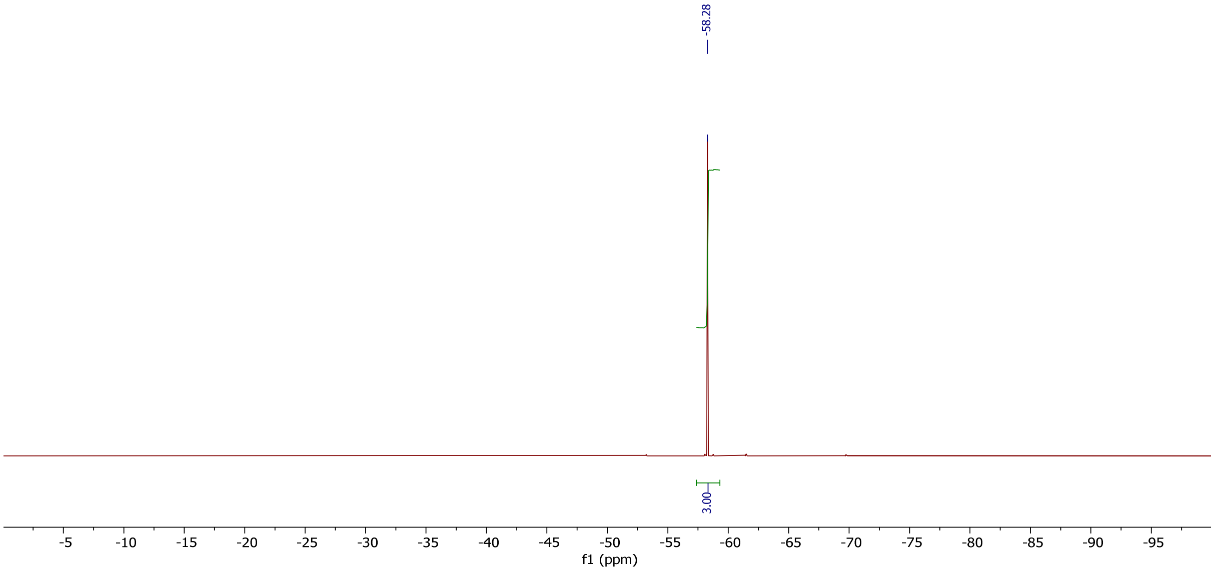

$^{19}F\{^1H\}$ NMR (377 MHz, $CDCl_3$) of **2g**.

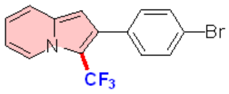

2h

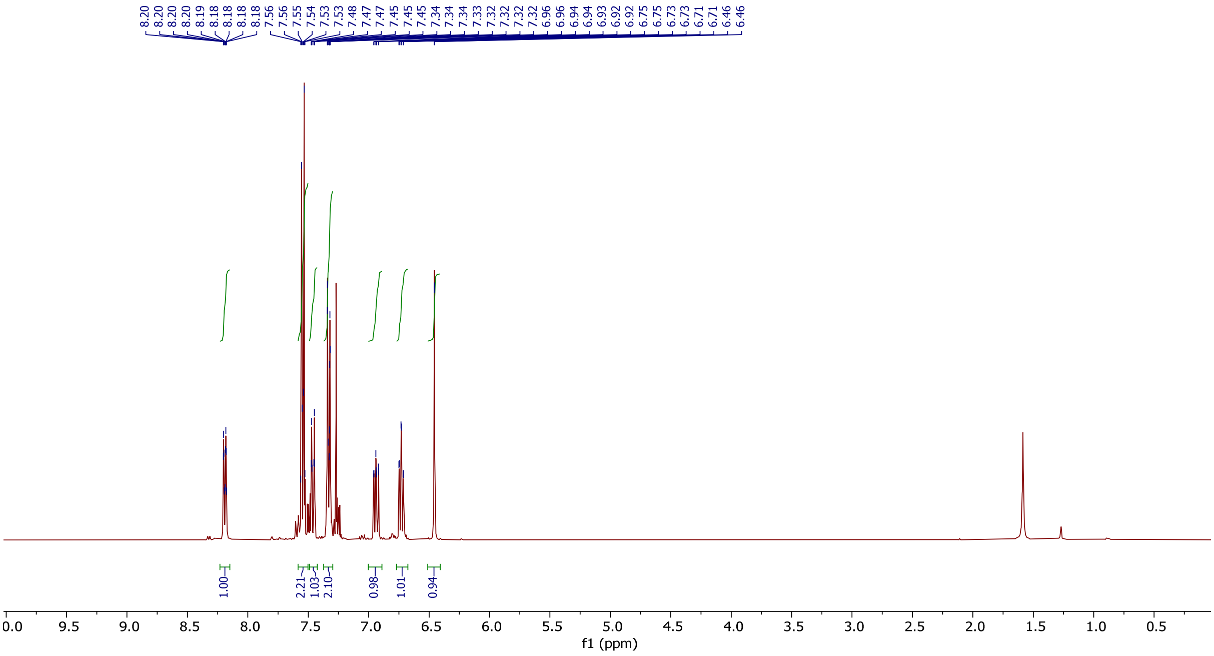

^1H NMR (400 MHz, $CDCl_3$) of **2h**.

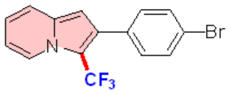

2h

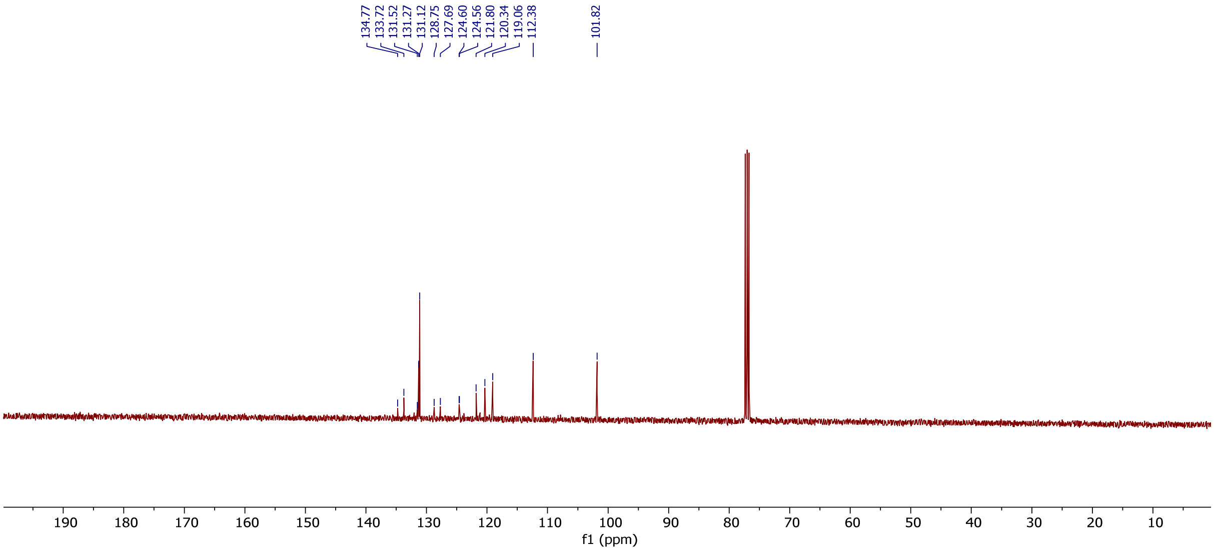

$^{13}C\{^{1}H\}$ NMR (100 MHz, $CDCl_3$) of **2h**.

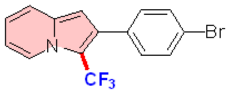

2h

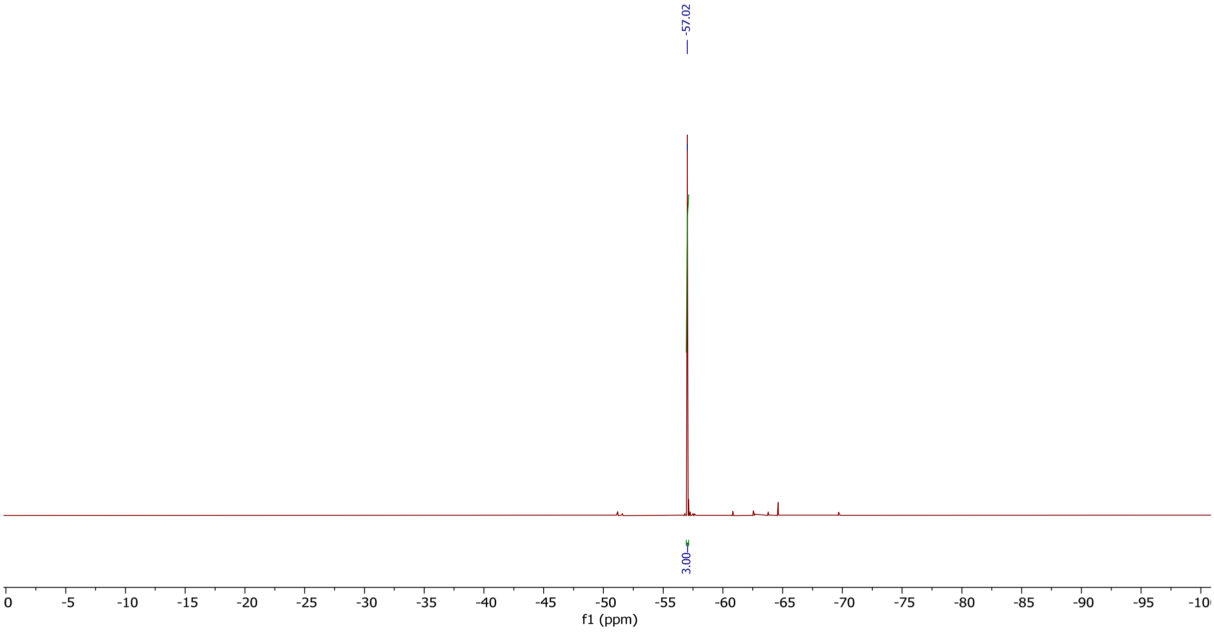

$^{19}F\{^{1}H\}$ NMR (377 MHz, $CDCl_3$) of **2h**.

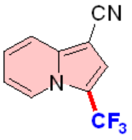

2i

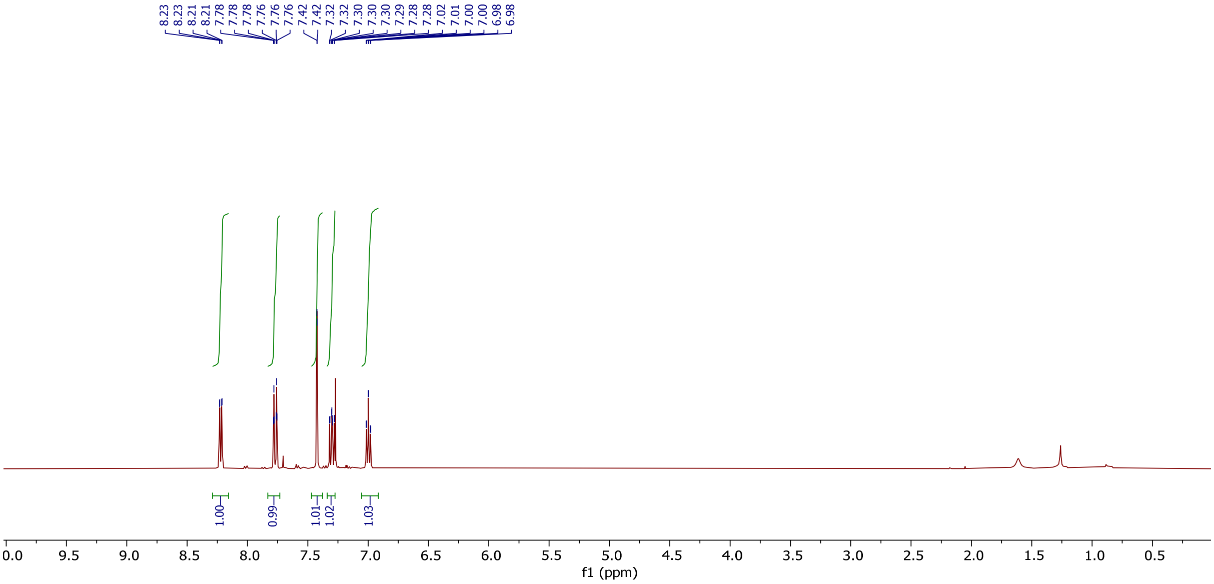

^{1}H NMR (400 MHz, $CDCl_3$) of **2i**.

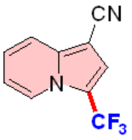

2i

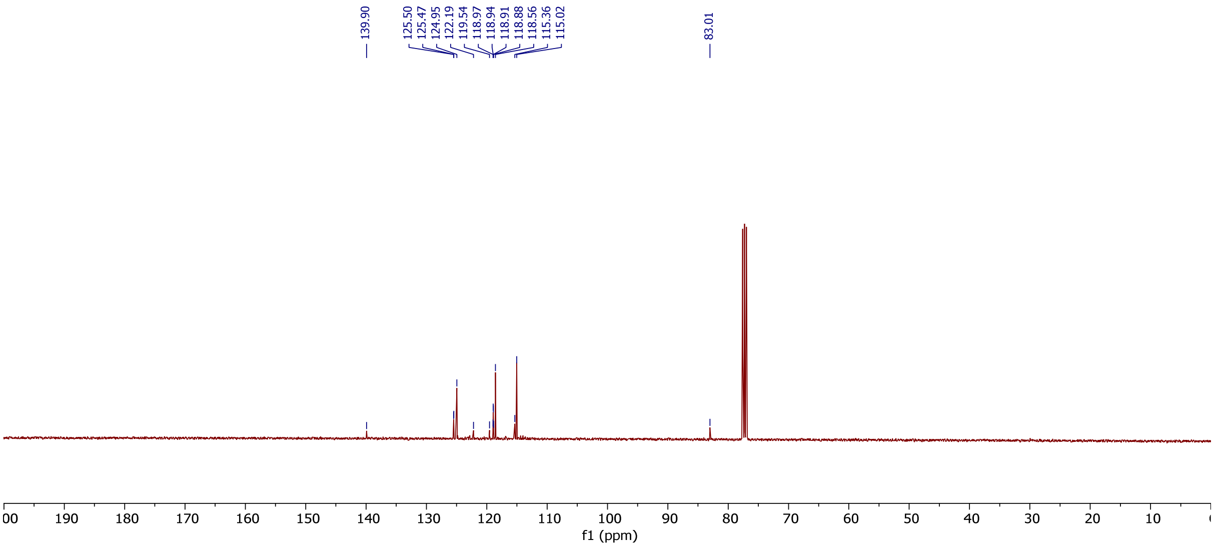

^{13}C{^{1}H} NMR (100 MHz, $CDCl_3$) of **2i**.

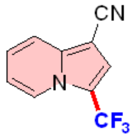

2i

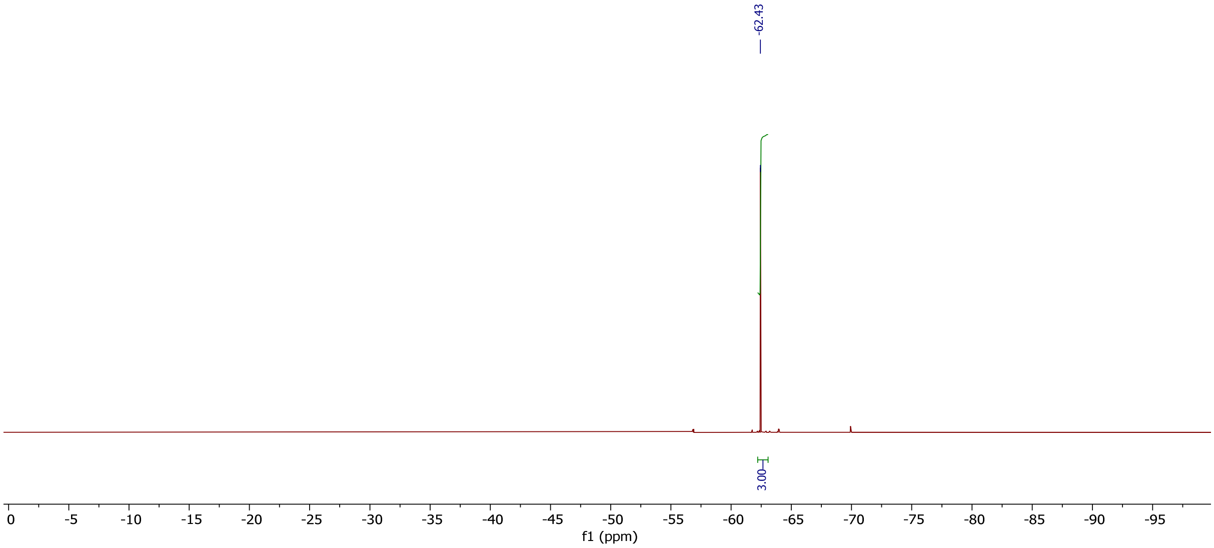

$^{19}F\{^{1}H\}$ NMR (377 MHz, $CDCl_3$) of **2i**.

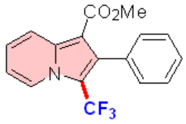

2j

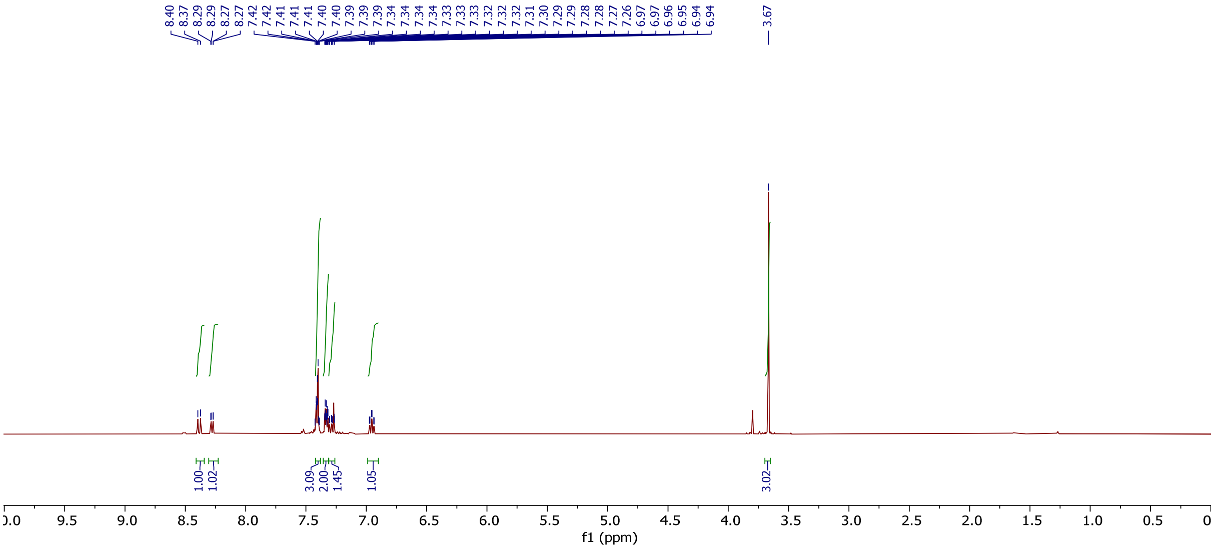

^{1}H NMR (400 MHz, $CDCl_3$) of **2j**.

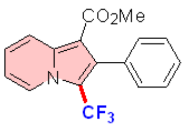

2j

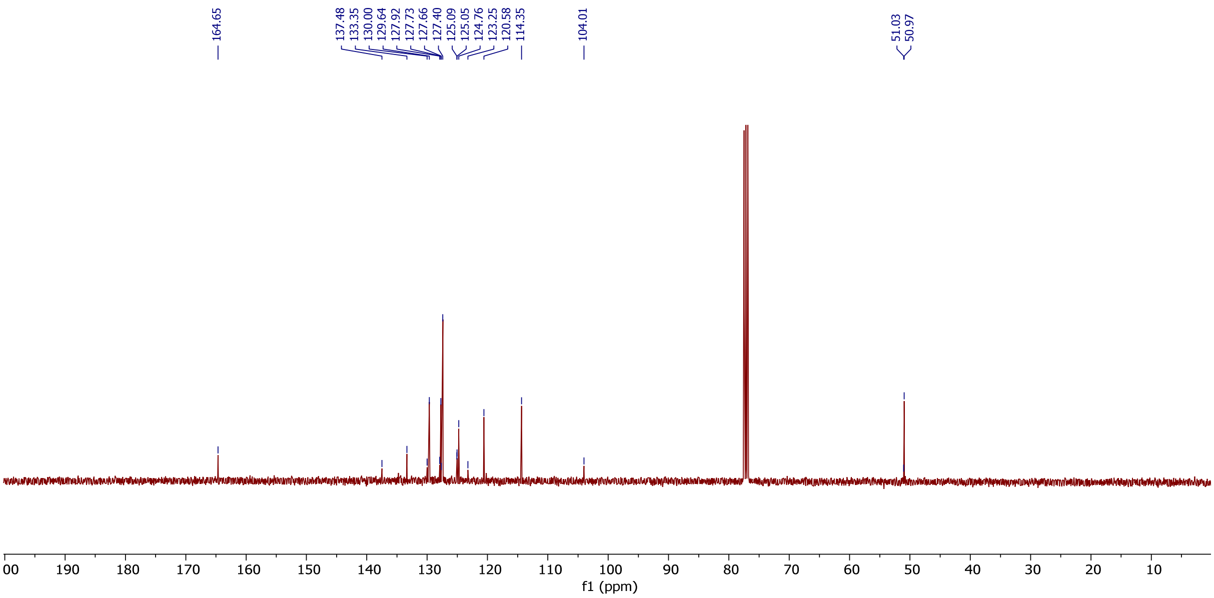

$^{13}C\{^{1}H\}$ NMR (100 MHz, $CDCl_3$) of **2j**.

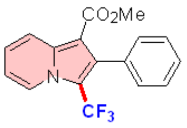

2j

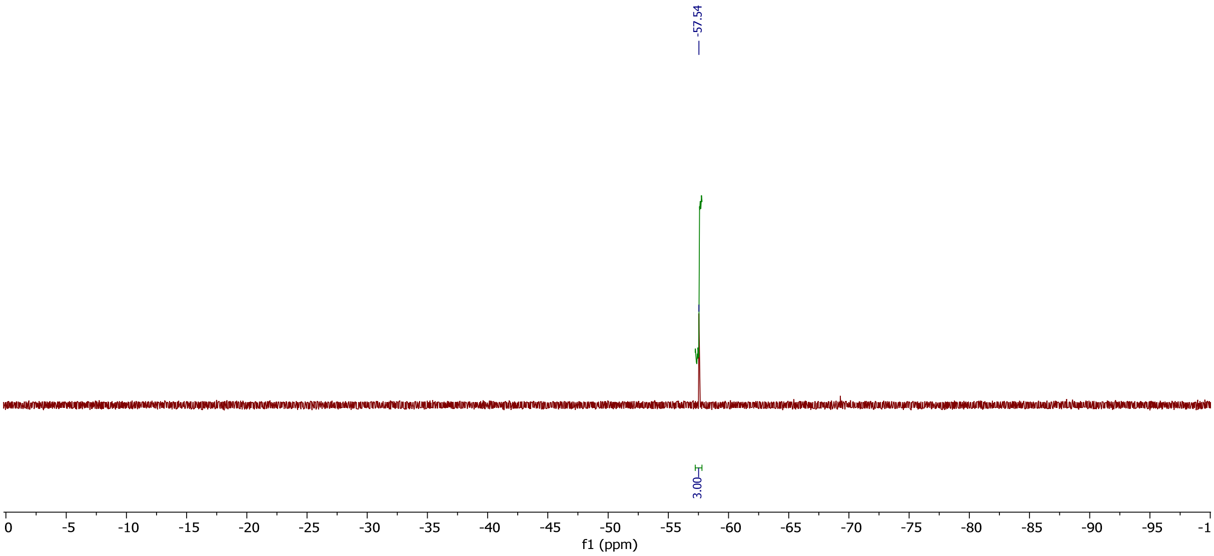

$^{19}F\{^{1}H\}$ NMR (377 MHz, $CDCl_3$) of **2j**.

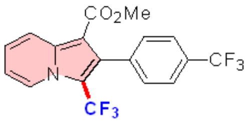

2k

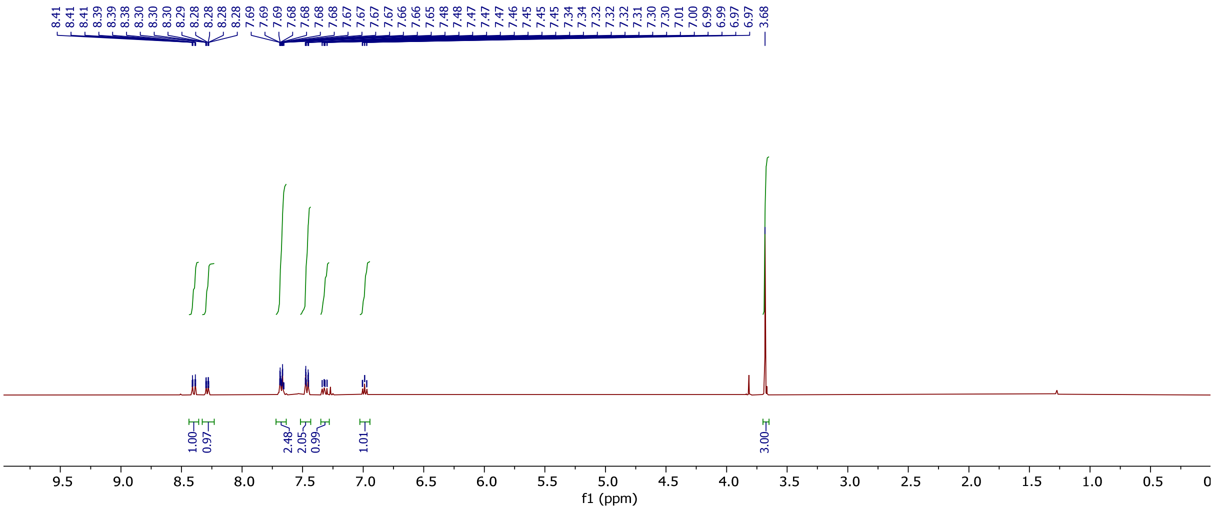

^{1}H NMR (400 MHz, $CDCl_3$) of **2k**.

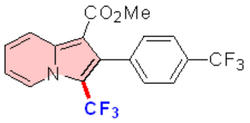

2k

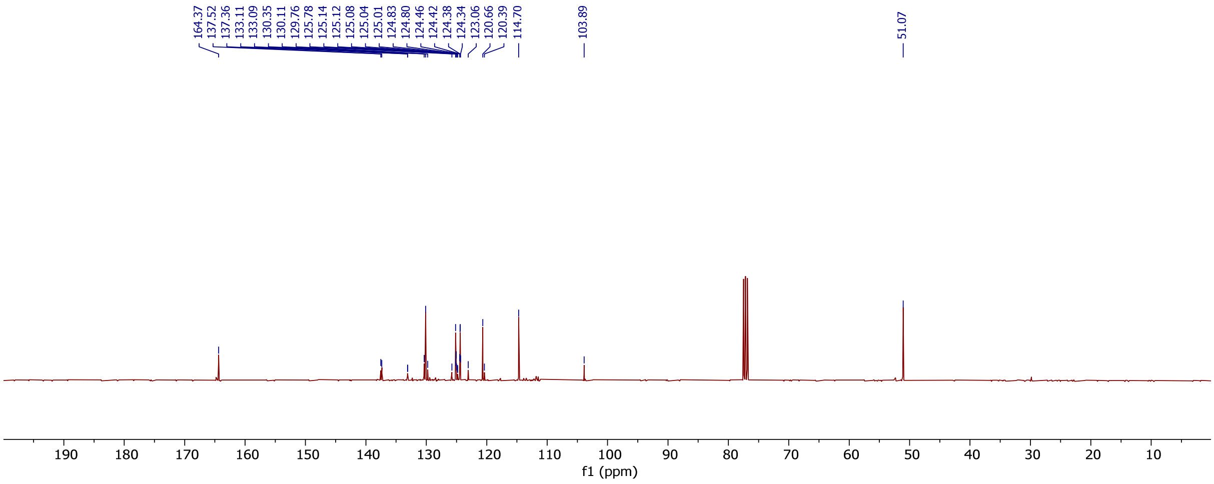

^{13}C{^{1}H} NMR (100 MHz, $CDCl_3$) of **2k**.

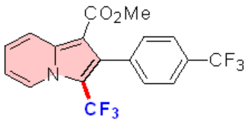

2k

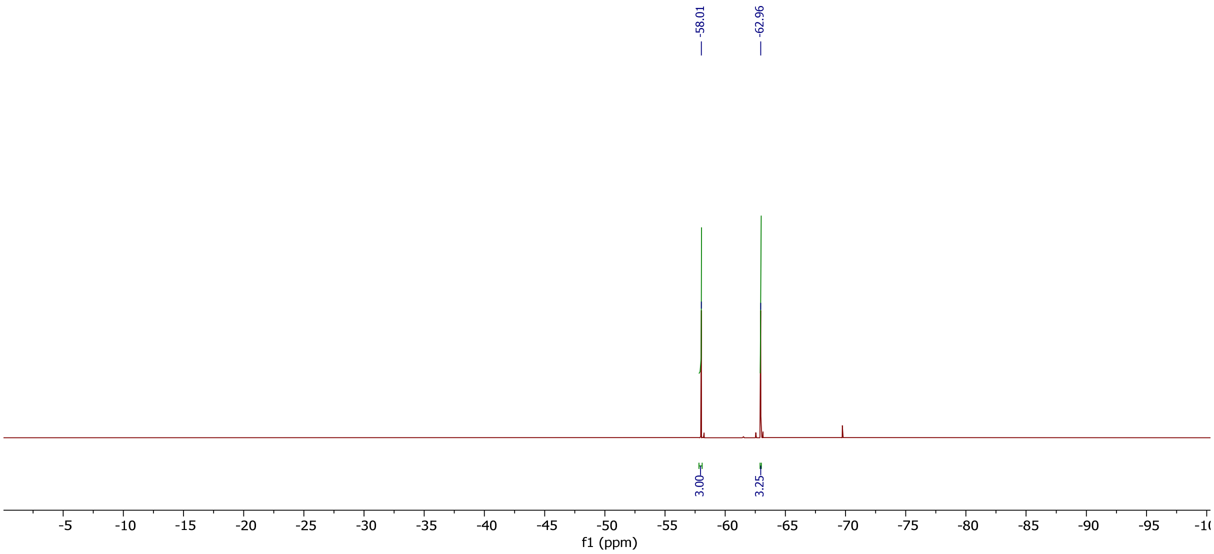

$^{19}F\{^{1}H\}$ NMR (377 MHz, $CDCl_3$) of **2k**.

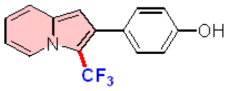

2l

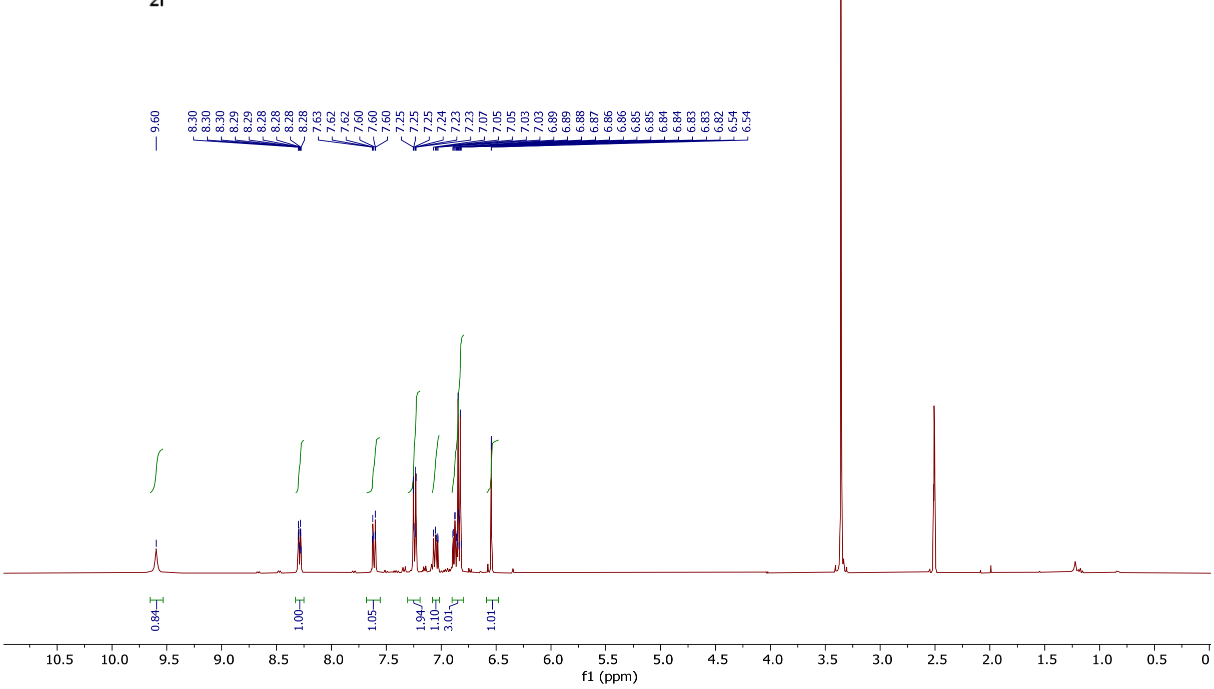

^{1}H NMR (400 MHz, DMSO[d^6]) of **2l**.

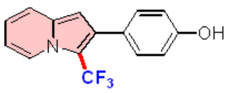

2l

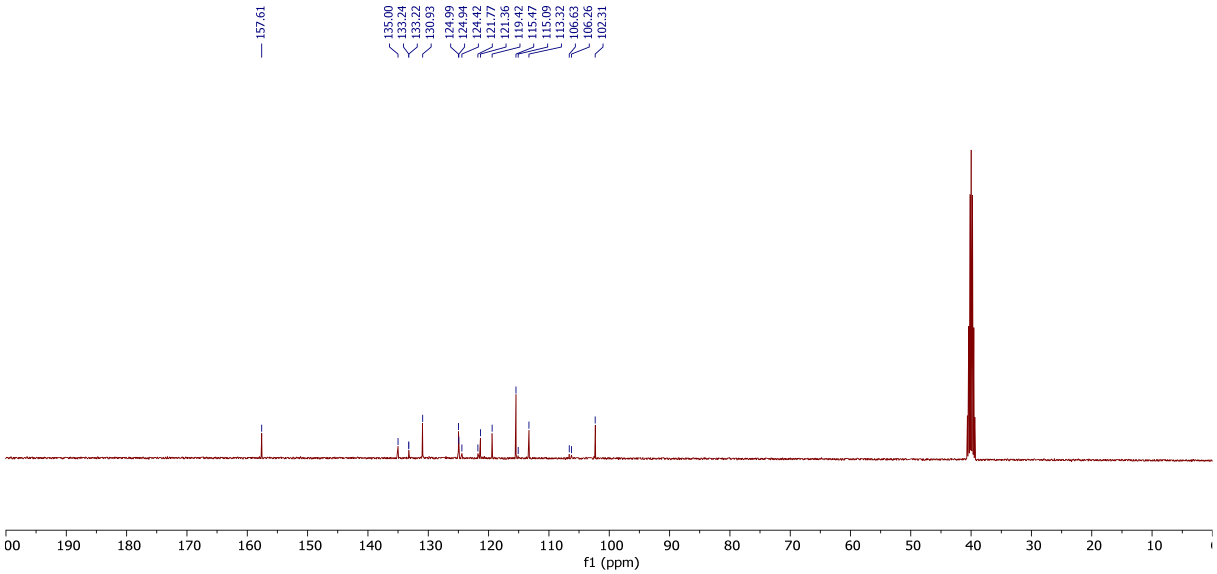

$^{13}C\{^{1}H\}$ NMR (100 MHz, DMSO[d^{6}]) of **2l**.

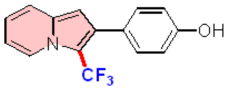

2l

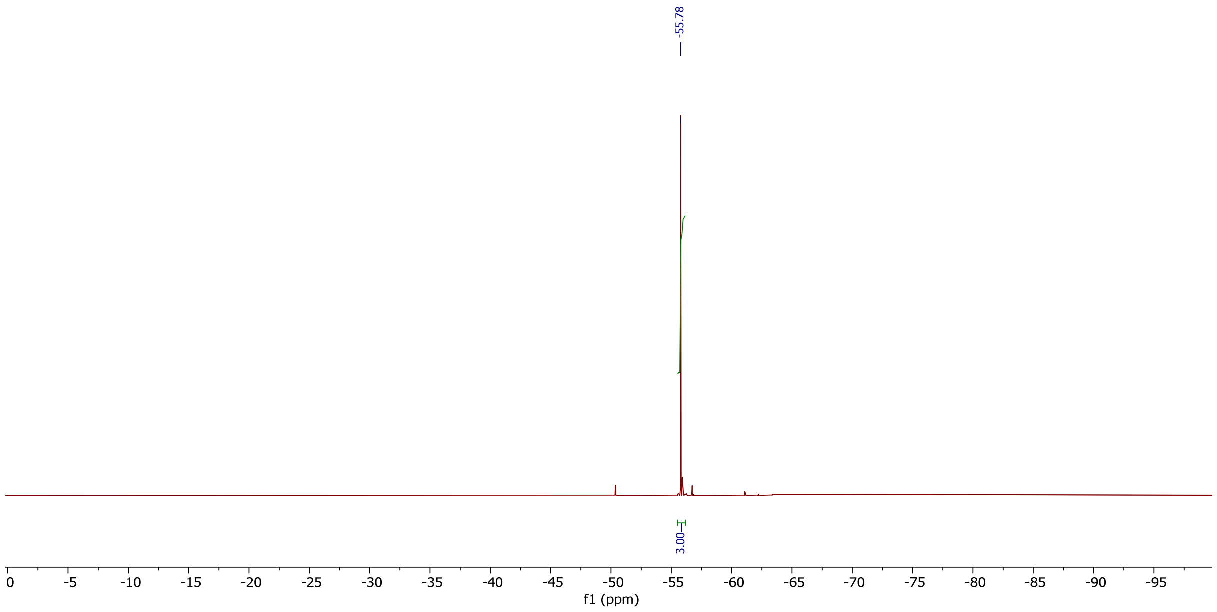

$^{19}F\{^{1}H\}$ NMR (377 MHz, DMSO[d^{6}]) of **2l**.

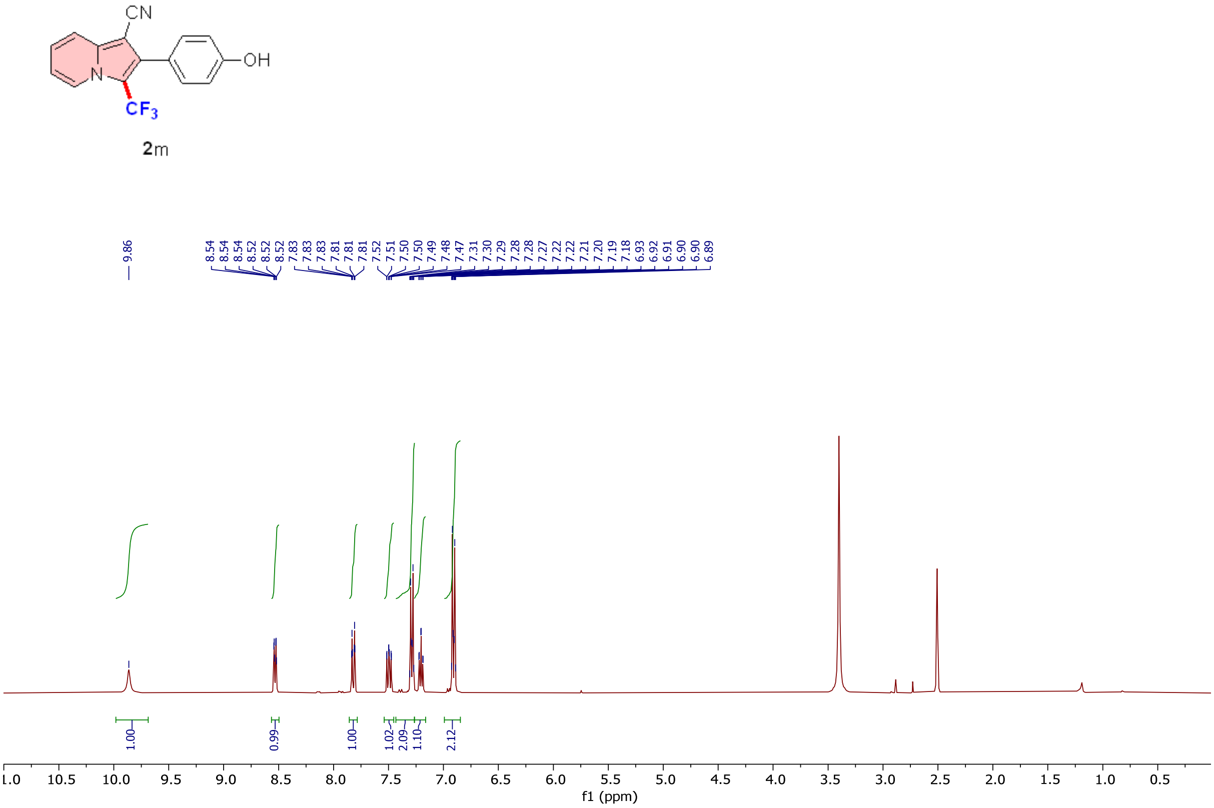

^{1}H NMR (400 MHz, DMSO[d^{6}]) of **2m**.

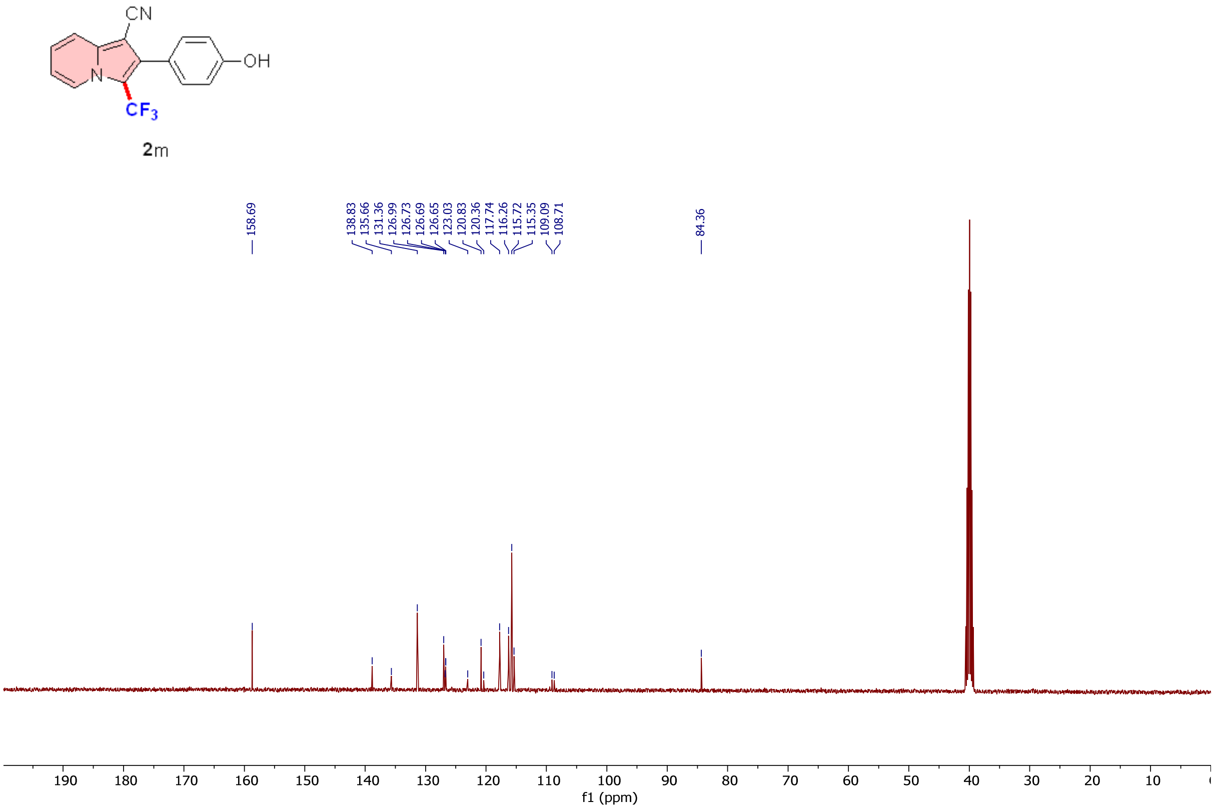

$^{13}C\{^{1}H\}$ NMR (100 MHz, DMSO[d^{6}]) of **2m**.

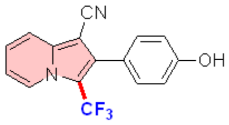

2m

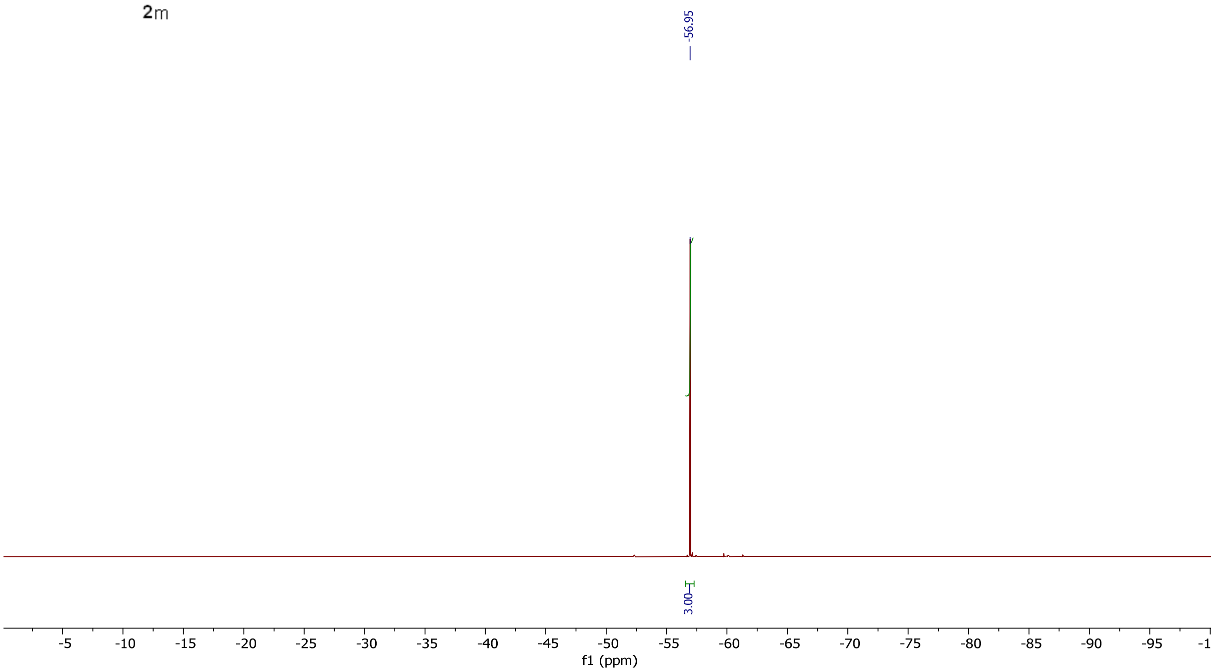

$^{19}F\{^1H\}$ NMR (377 MHz, DMSO[d[6]]) of **2m**.

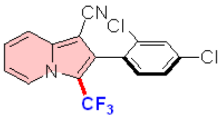

2n

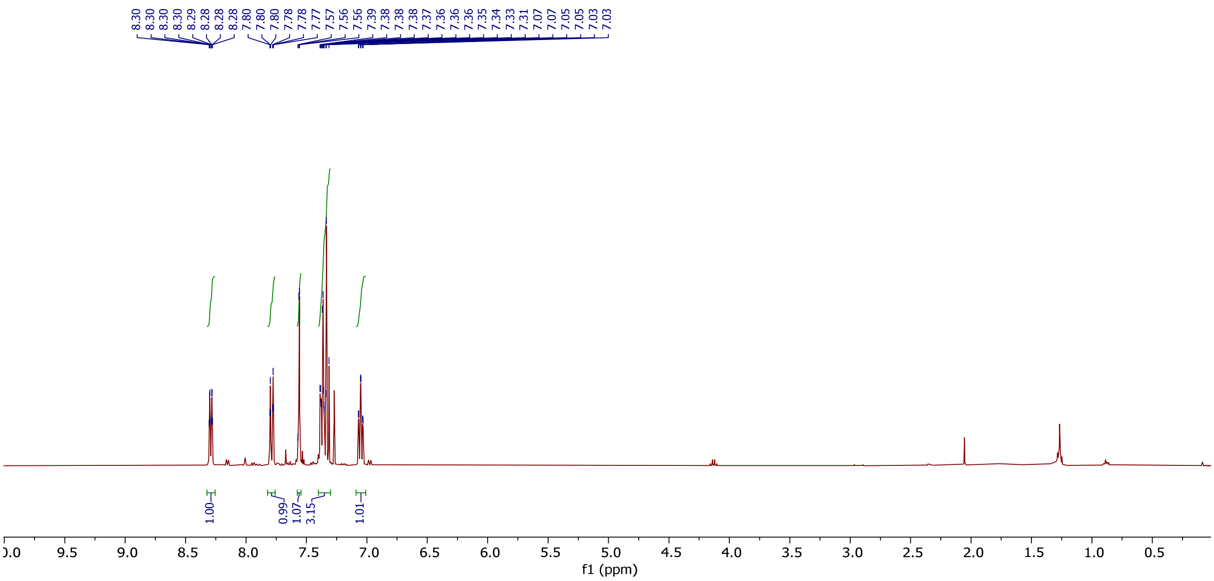

^1H NMR (400 MHz, $CDCl_3$) of **2n**.

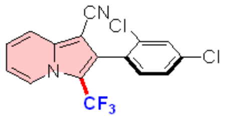

2n

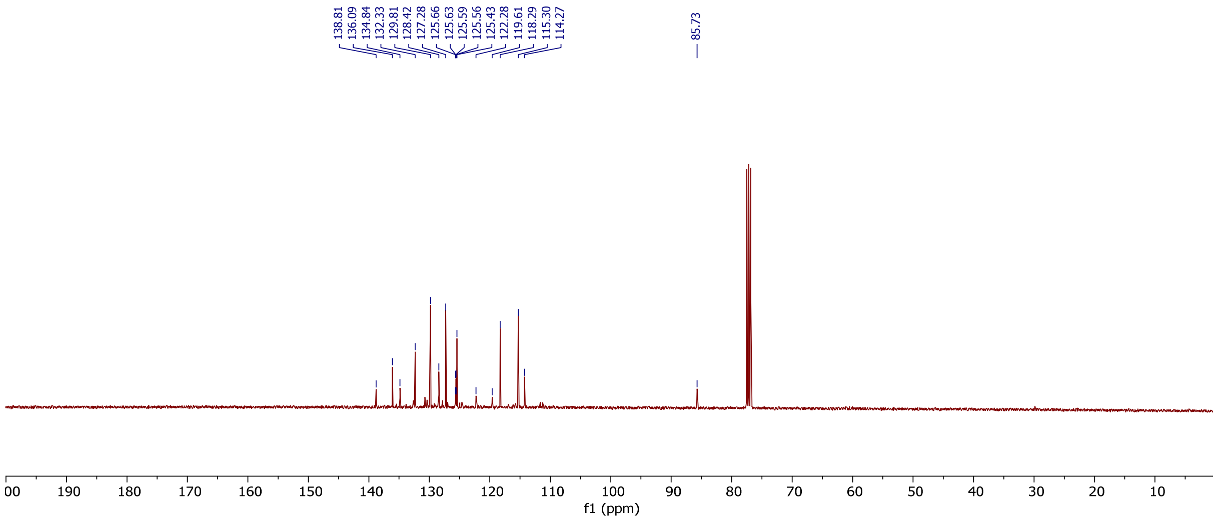

$^{13}C\{^1H\}$ NMR (100 MHz, $CDCl_3$) of **2n**.

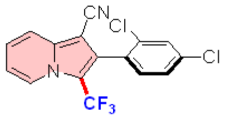

2n

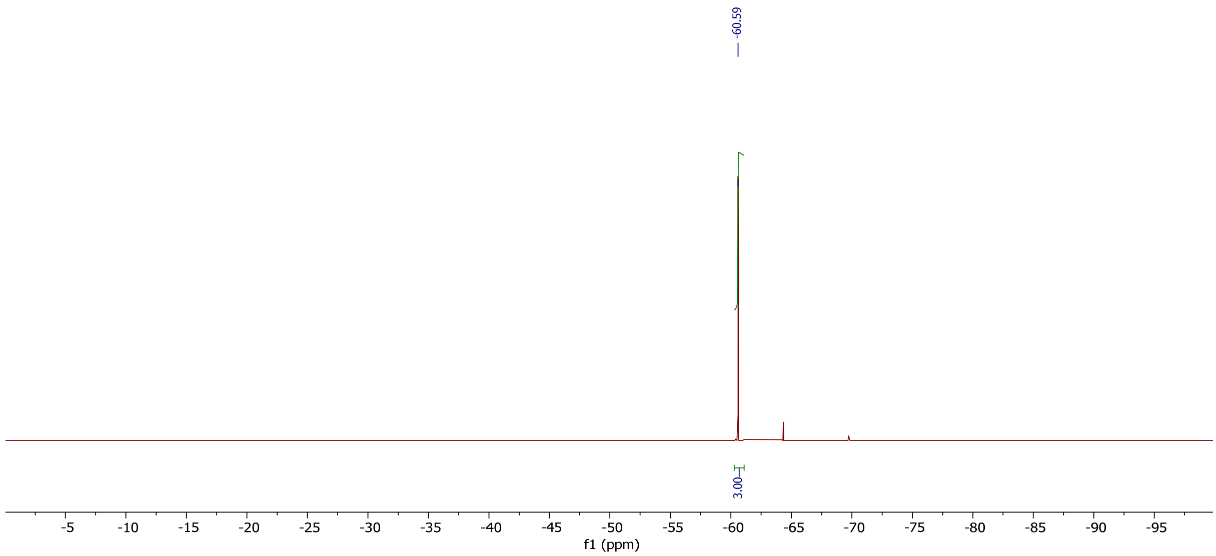

$^{19}F\{^1H\}$ NMR (377 MHz, $CDCl_3$) of **2n**.

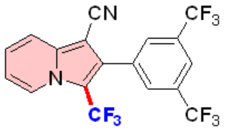

2o

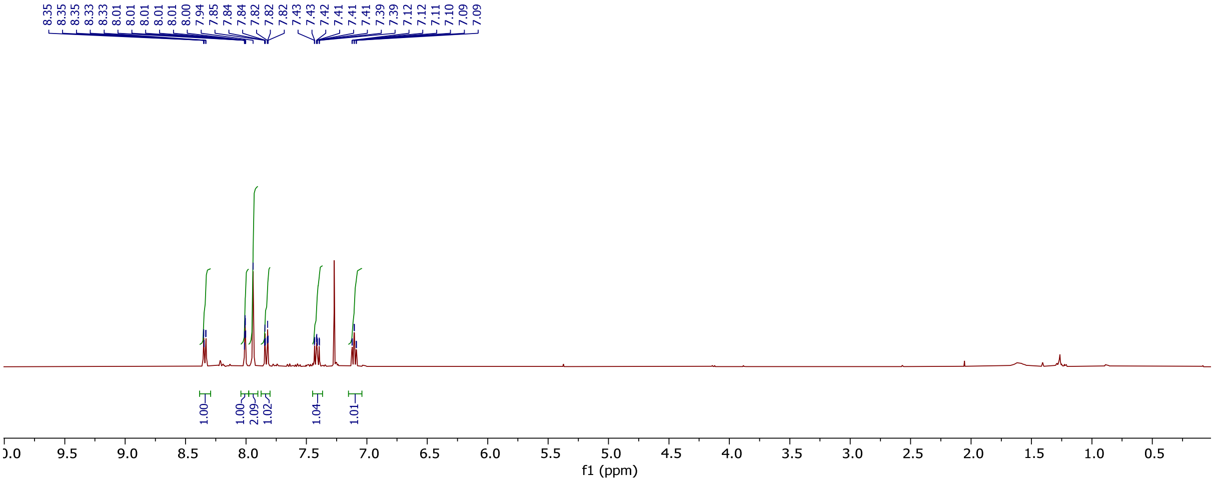

^{1}H NMR (400 MHz, $CDCl_3$) of **2o**.

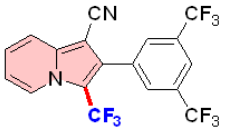

2o

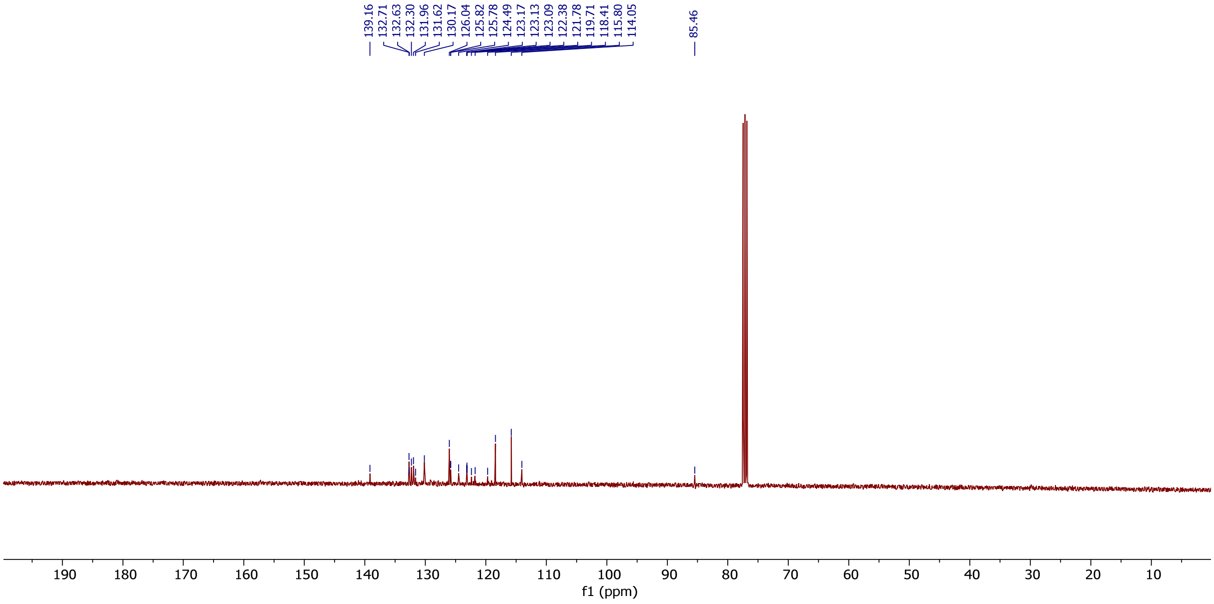

$^{13}C\{^{1}H\}$ NMR (100 MHz, $CDCl_3$) of **2o**.

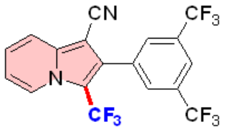

2o

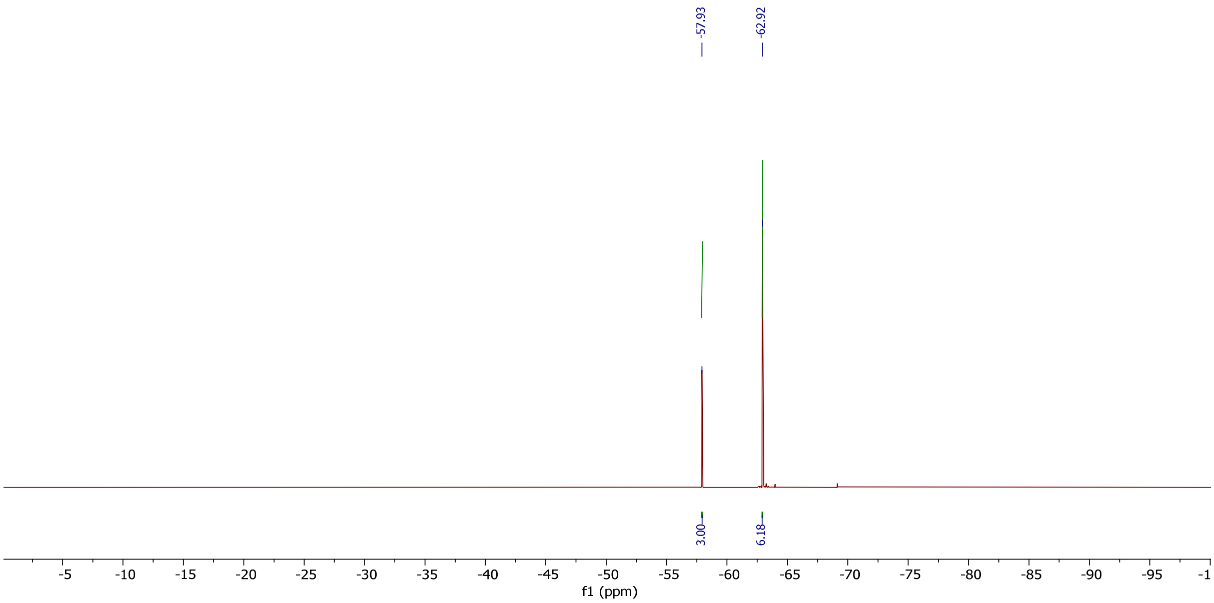

$^{19}F\{^{1}H\}$ NMR (377 MHz, $CDCl_3$) of **2o**.

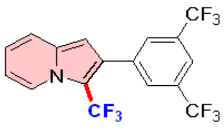

2p

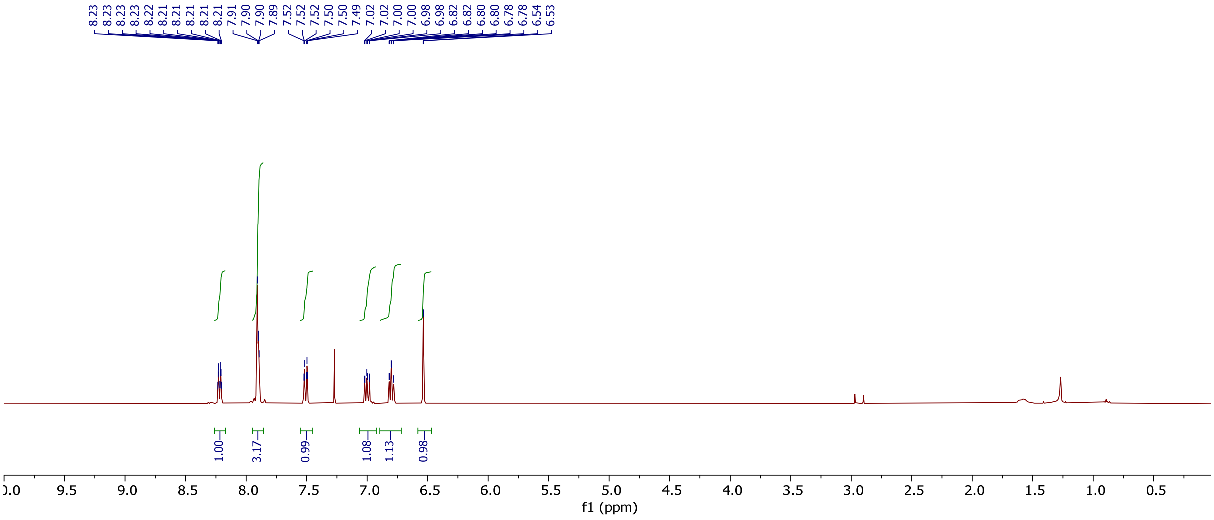

^{1}H NMR (400 MHz, $CDCl_3$) of **2p**.

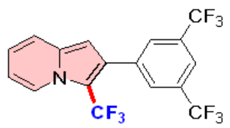

2p

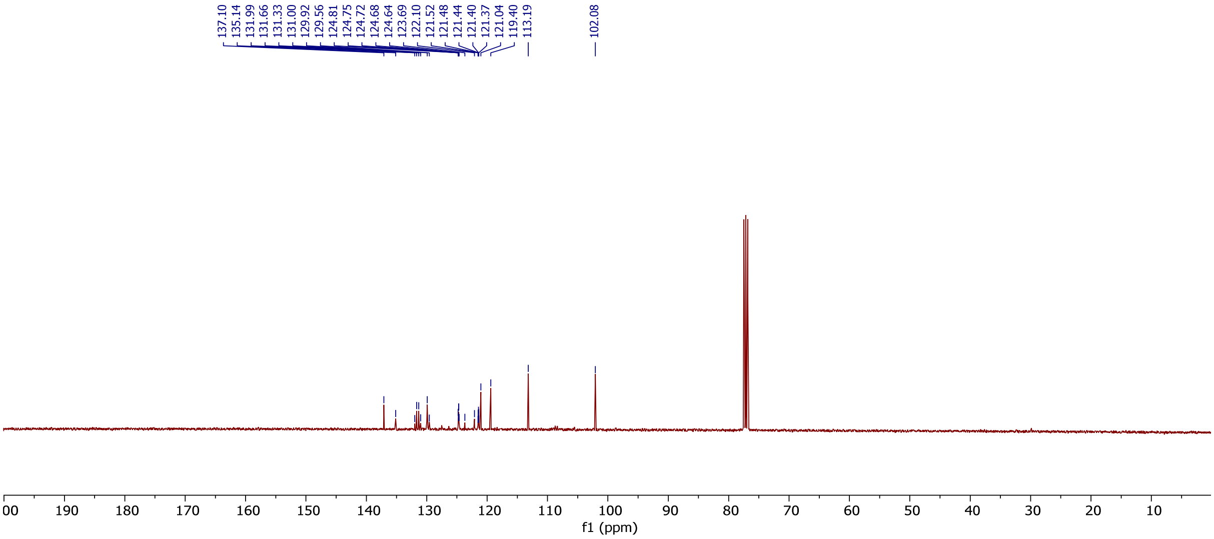

$^{13}C\{^1H\}$ NMR (100 MHz, $CDCl_3$) of **2p**.

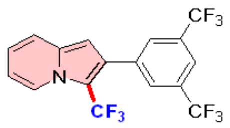

2p

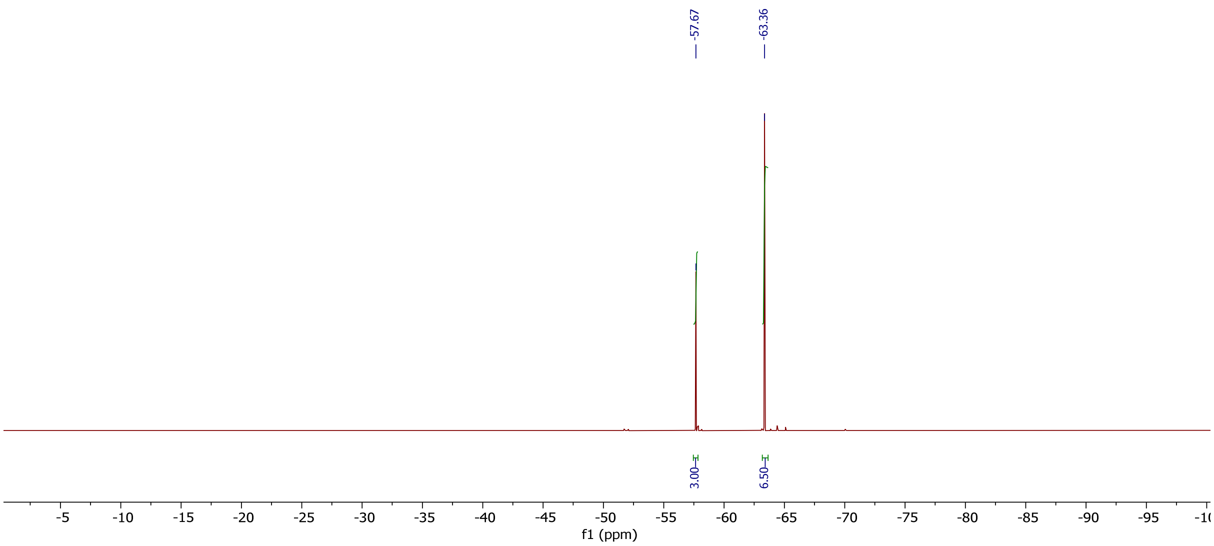

$^{19}F\{^1H\}$ NMR (377 MHz, $CDCl_3$) of **2p**.

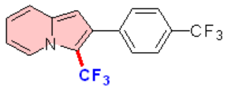

2q

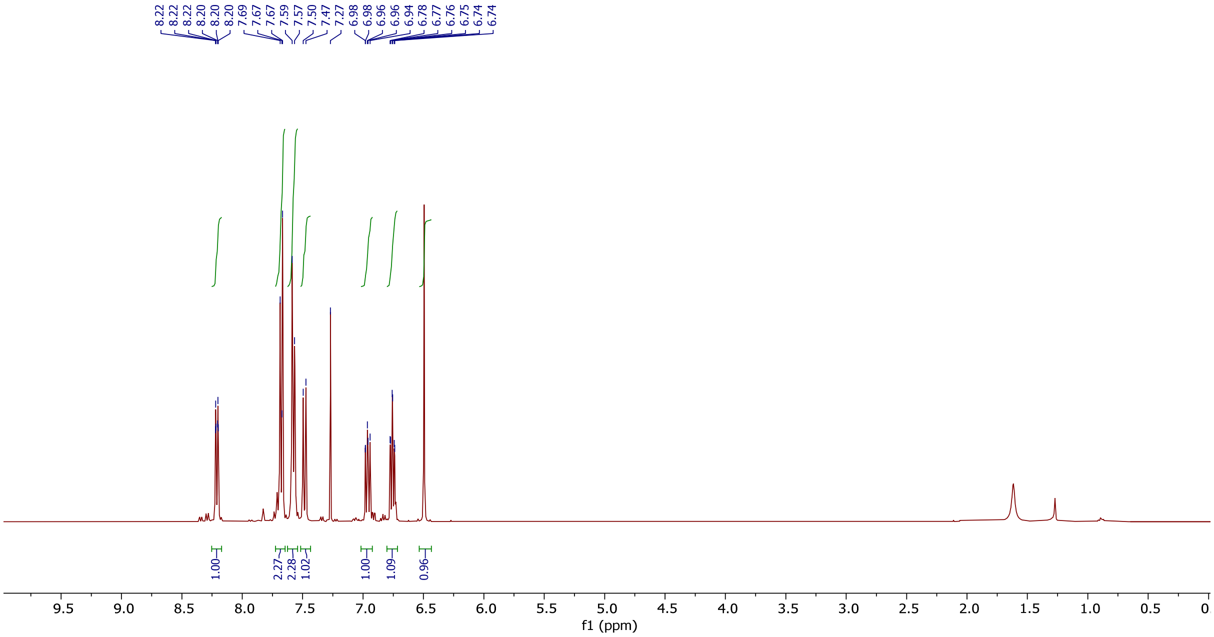

^{1}H NMR (400 MHz, $CDCl_3$) of **2q**.

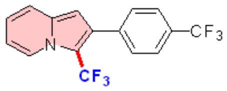

2q

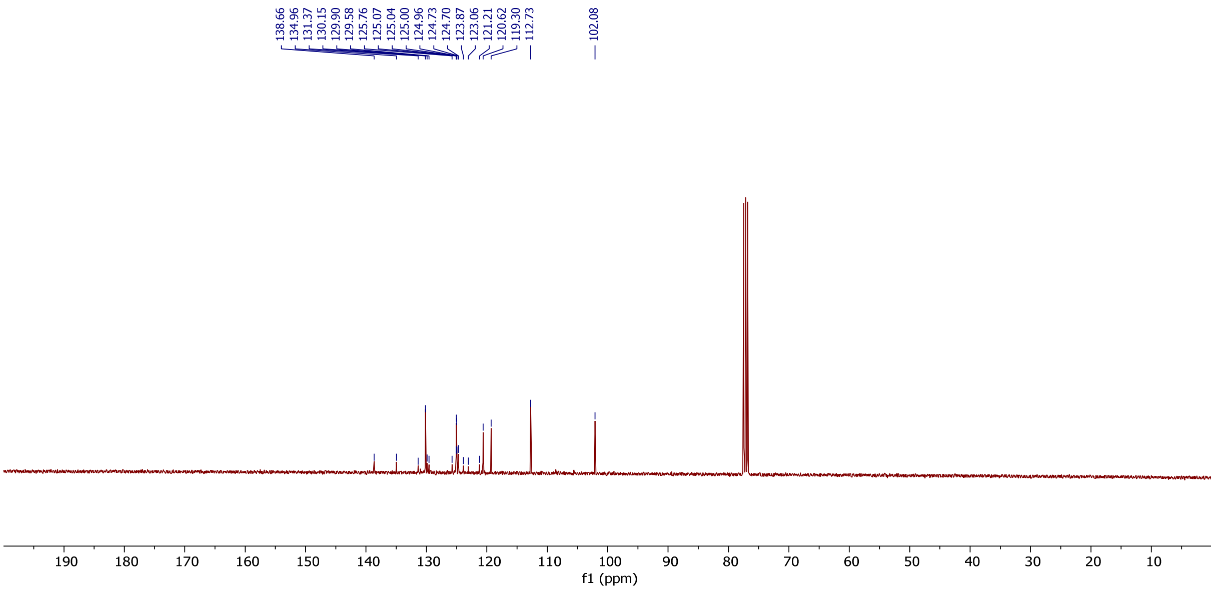

^{13}C{^{1}H} NMR (100 MHz, $CDCl_3$) of **2q**.

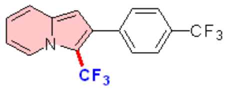

2q

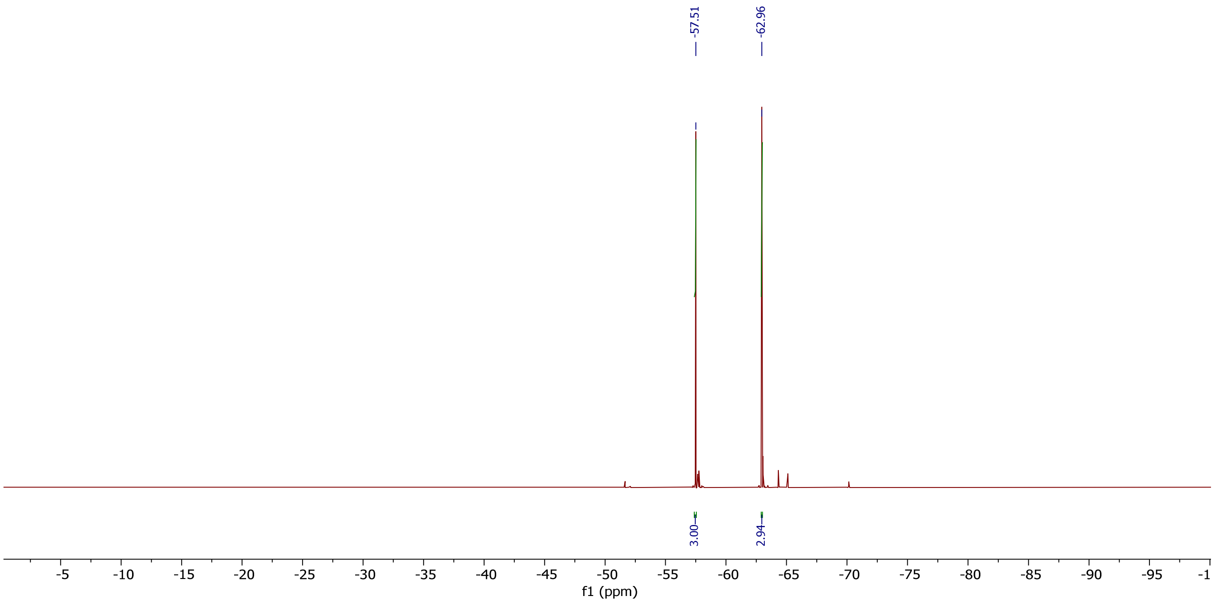

$^{19}F\{^{1}H\}$ NMR (377 MHz, $CDCl_3$) of **2q**.

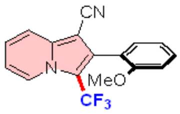

2r

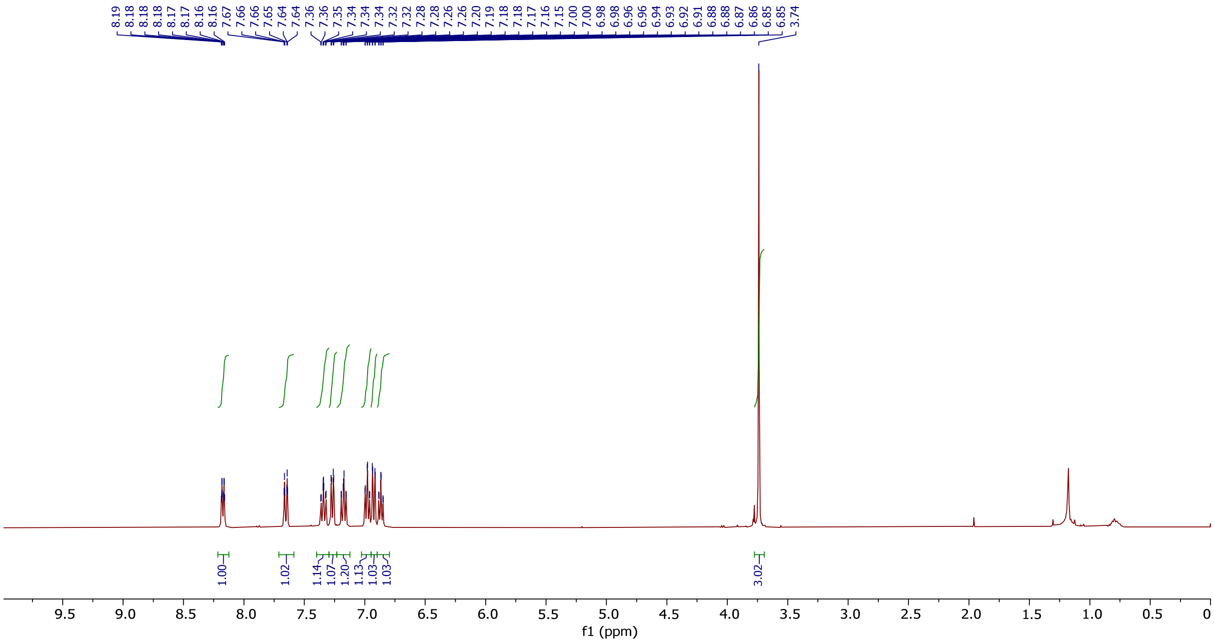

^{1}H NMR (400 MHz, $CDCl_3$) of **2r**.

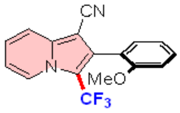

2r

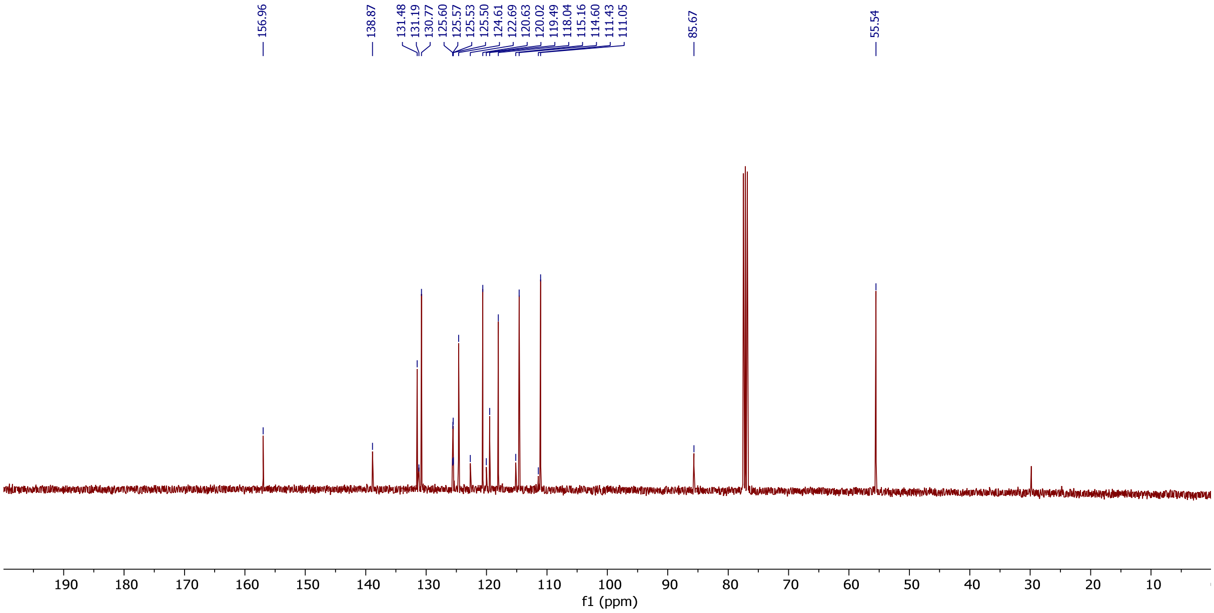

$^{13}C\{^{1}H\}$ NMR (100 MHz, $CDCl_3$) of **2r**.

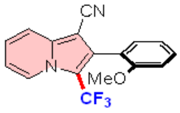

2r

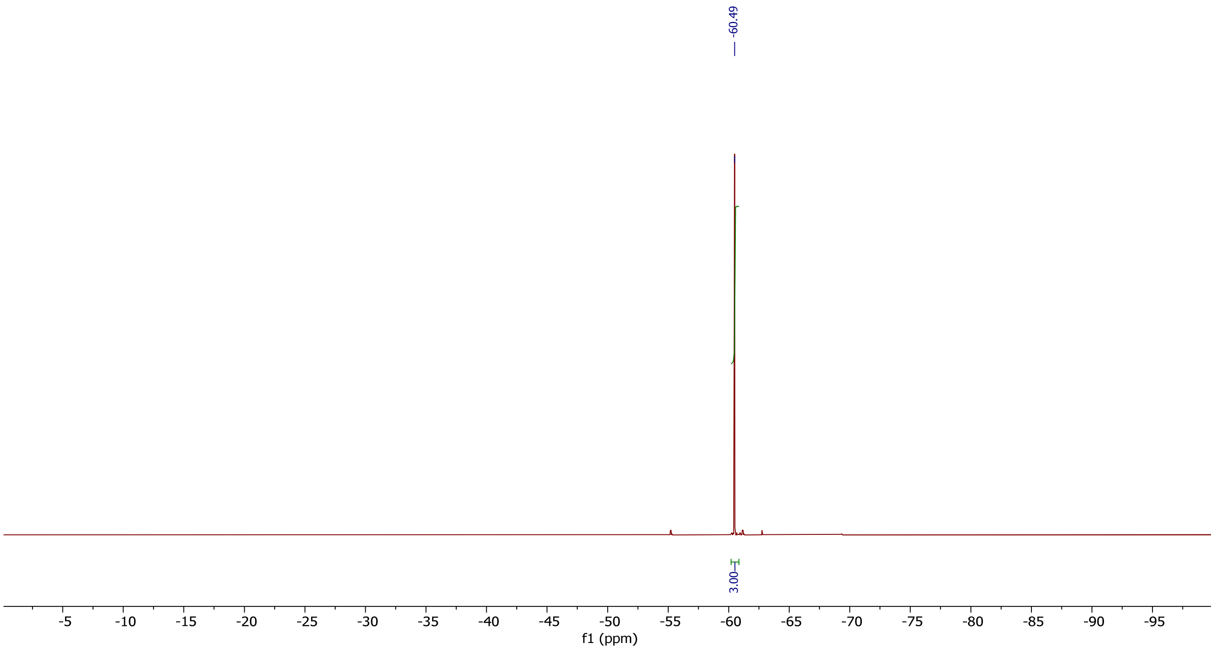

$^{19}F\{^{1}H\}$ NMR (377 MHz, $CDCl_3$) of **2r**.

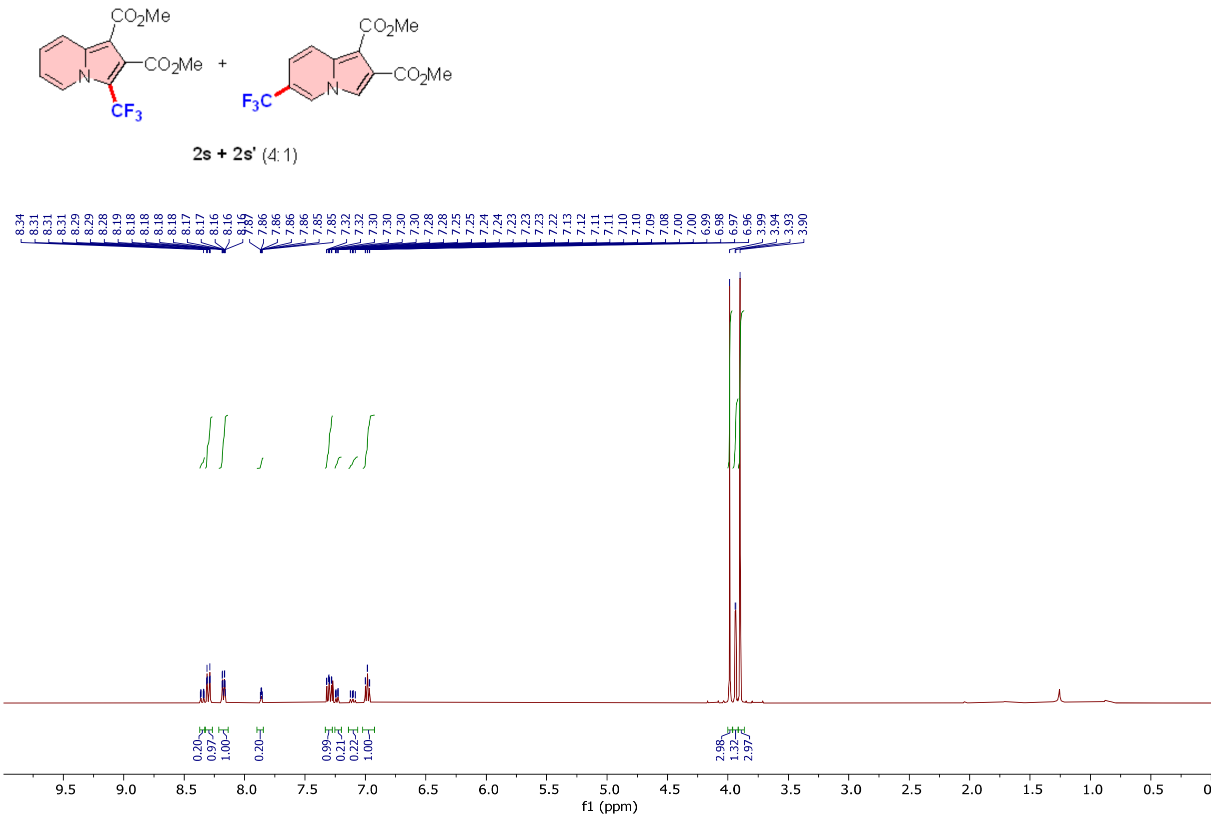

^{1}H NMR (400 MHz, $CDCl_3$) of **2s + 2s'**.

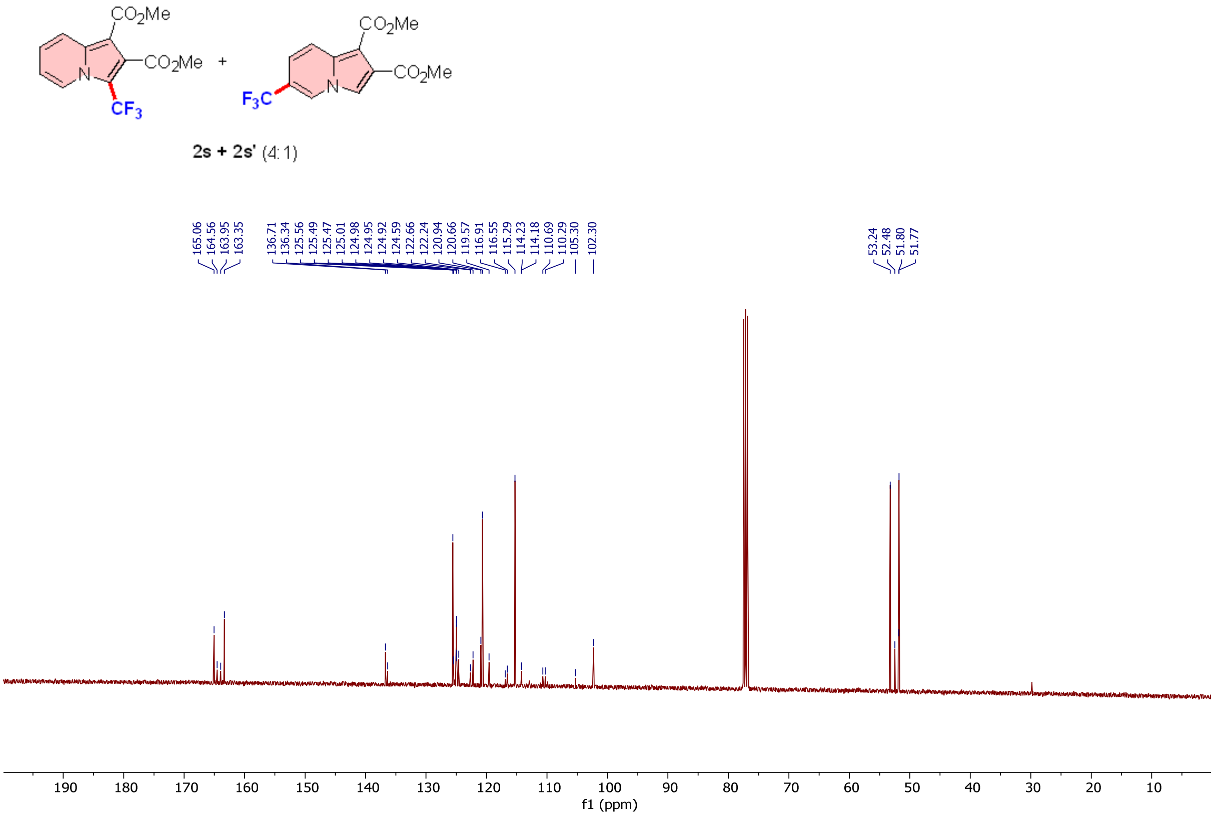

$^{13}C\{^{1}H\}$ NMR (100 MHz, $CDCl_3$) of **2s + 2s'**.

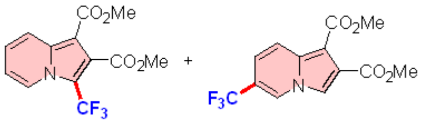

2s + 2s' (4:1)

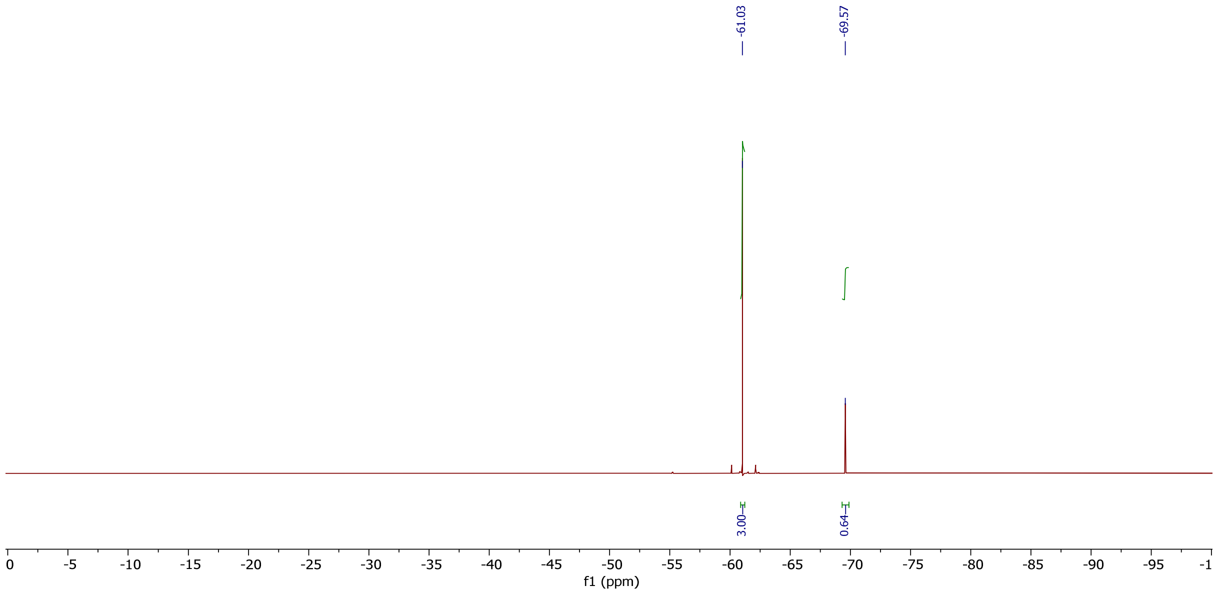

$^{19}F\{^{1}H\}$ NMR (377 MHz, $CDCl_3$) of **2s + 2s'**.

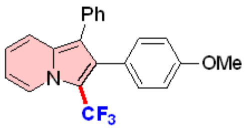

2t

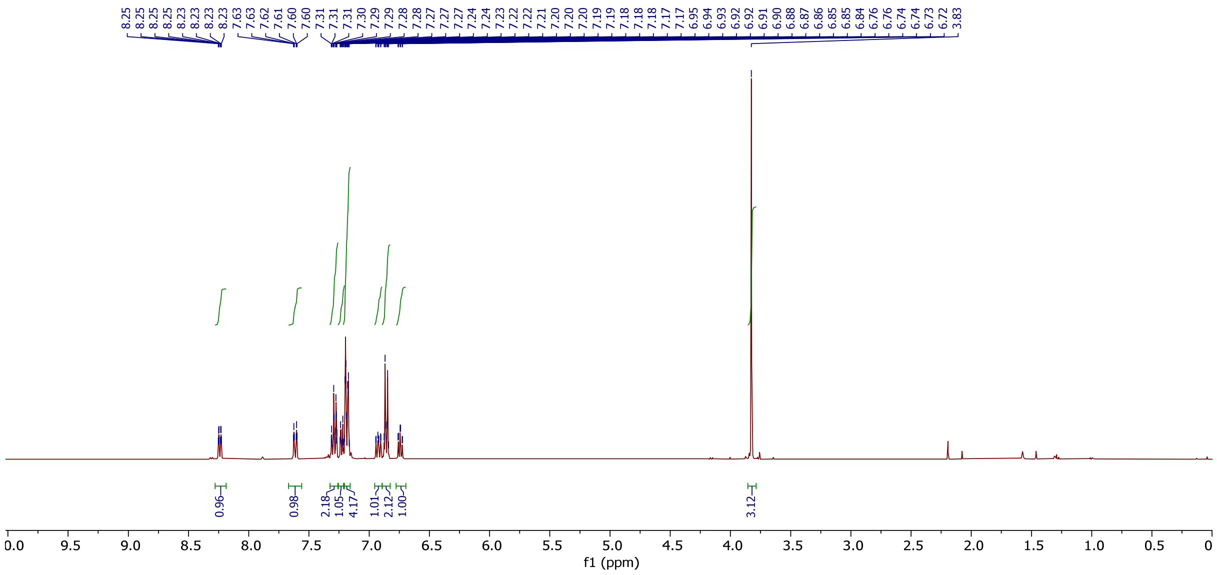

^{1}H NMR (400 MHz, $CDCl_3$) of **2t**.

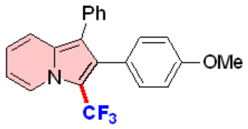

2t

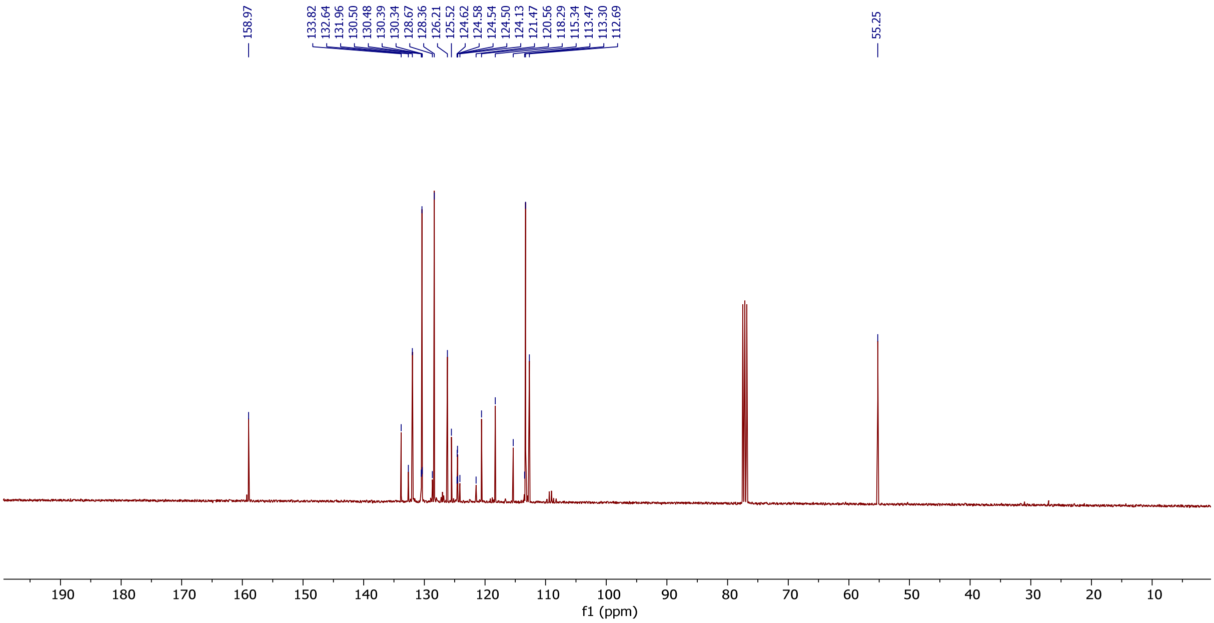

$^{13}C\{^{1}H\}$ NMR (100 MHz, $CDCl_3$) of **2t**.

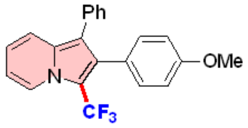

2t

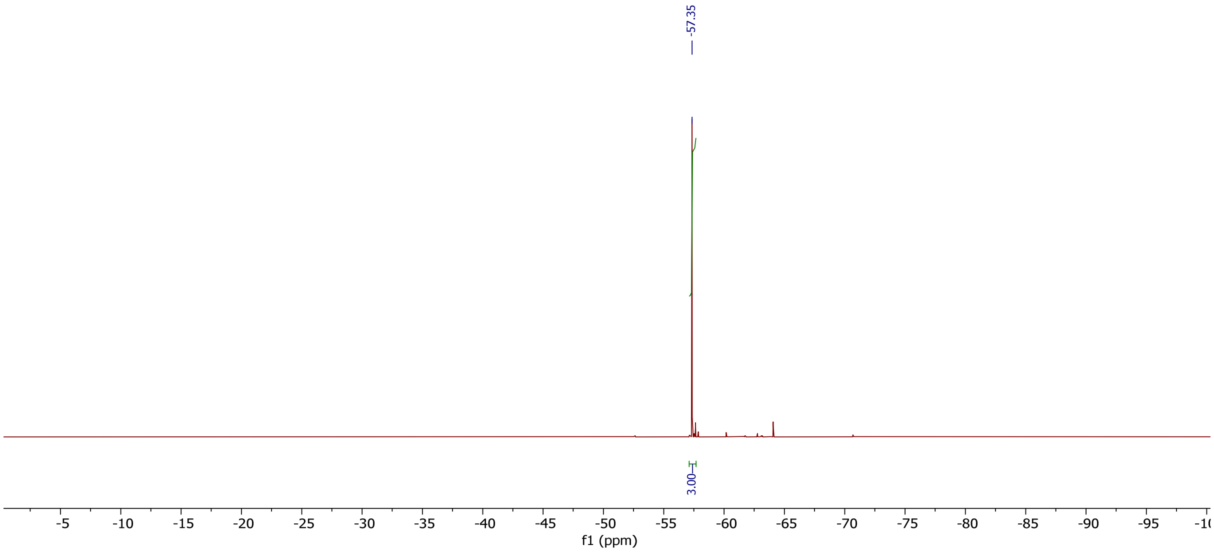

$^{19}F\{^{1}H\}$ NMR (377 MHz, $CDCl_3$) of **2t**.

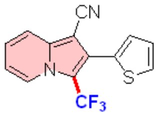

2u

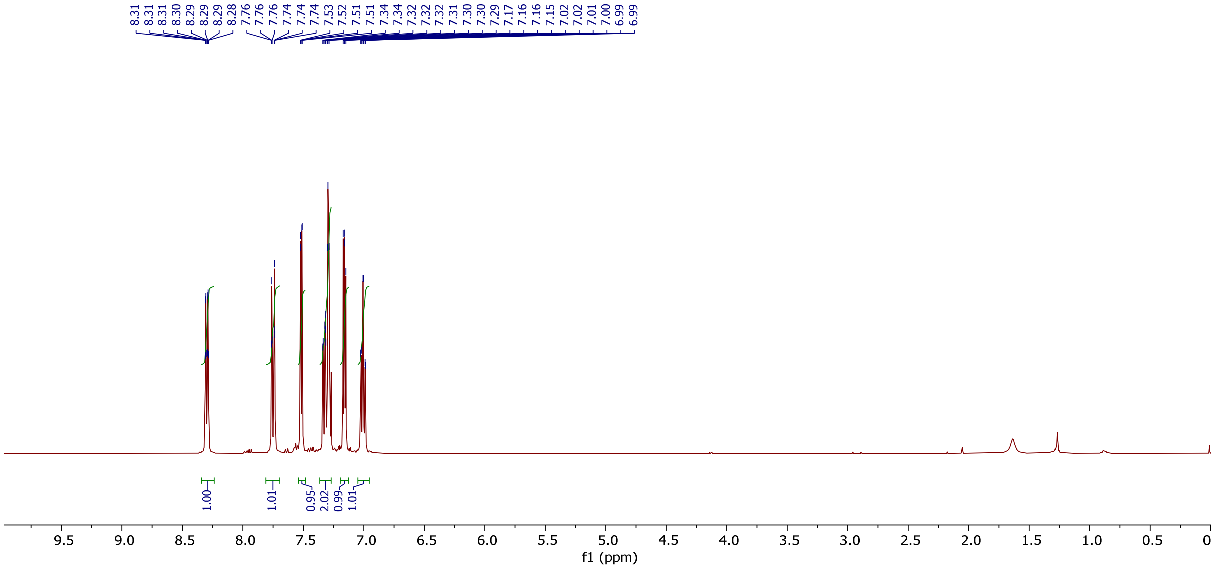

^{1}H NMR (400 MHz, $CDCl_3$) of **2u.**

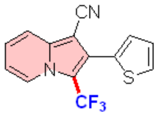

2u

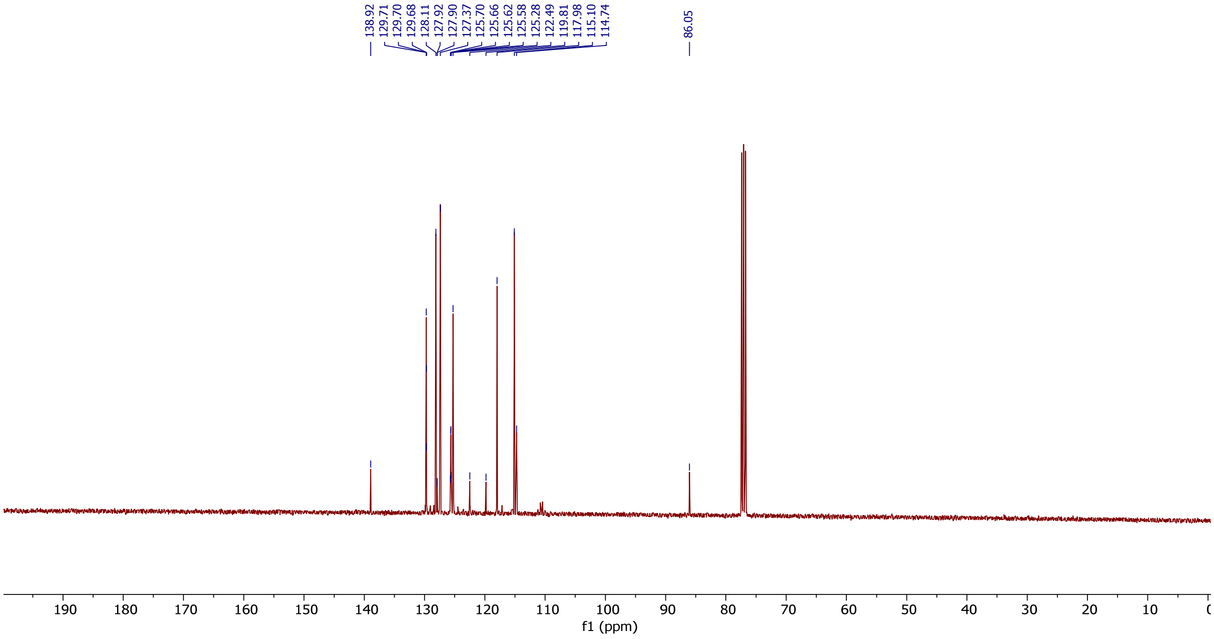

^{13}C{^{1}H} NMR (100 MHz, $CDCl_3$) of **2u**.

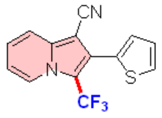

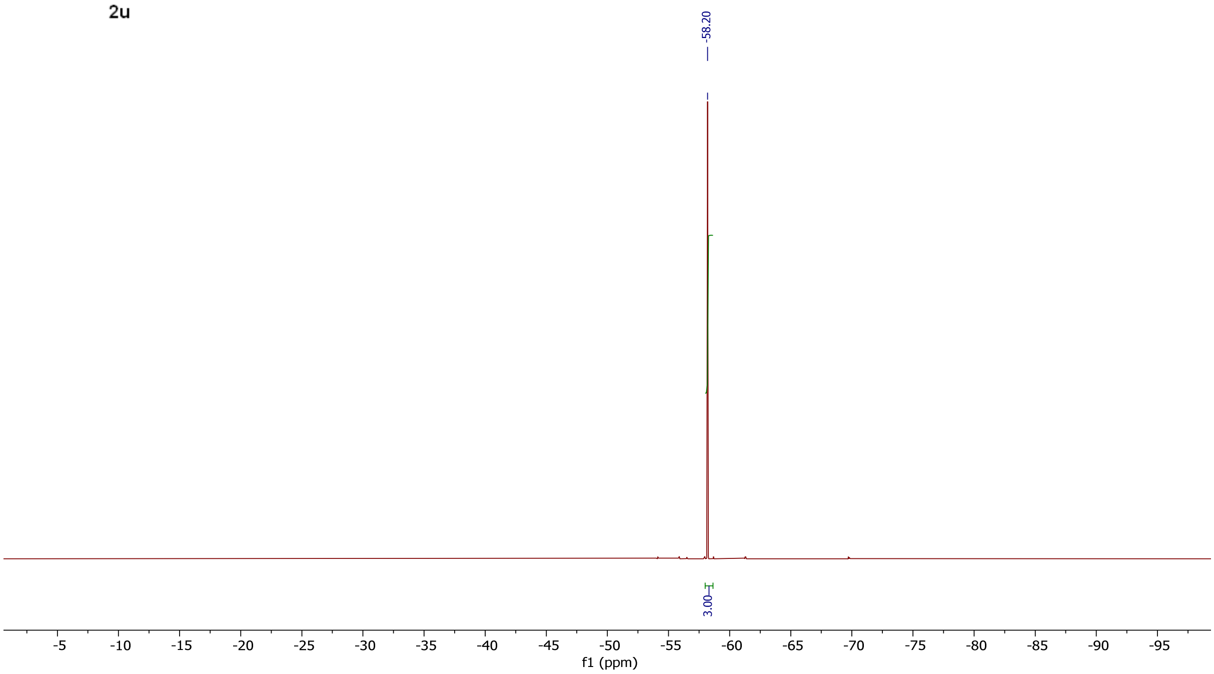

$^{19}F\{^1H\}$ NMR (377 MHz, $CDCl_3$) of **2u**.

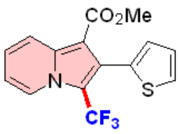

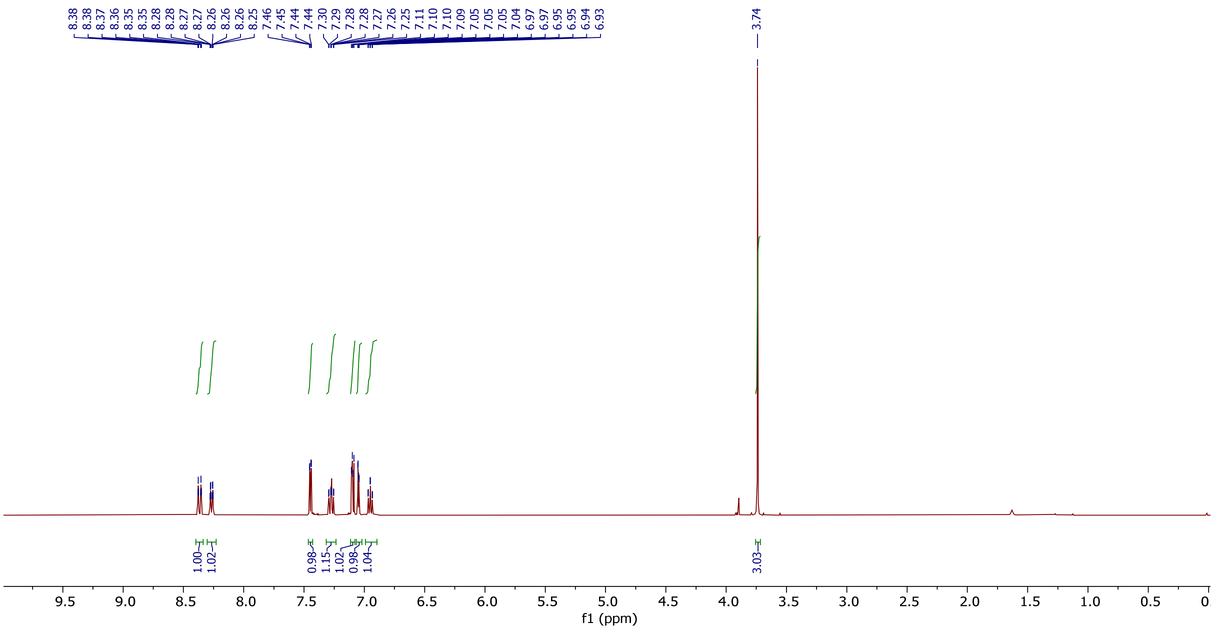

^1H NMR (400 MHz, $CDCl_3$) of **2w**.

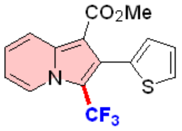

2w

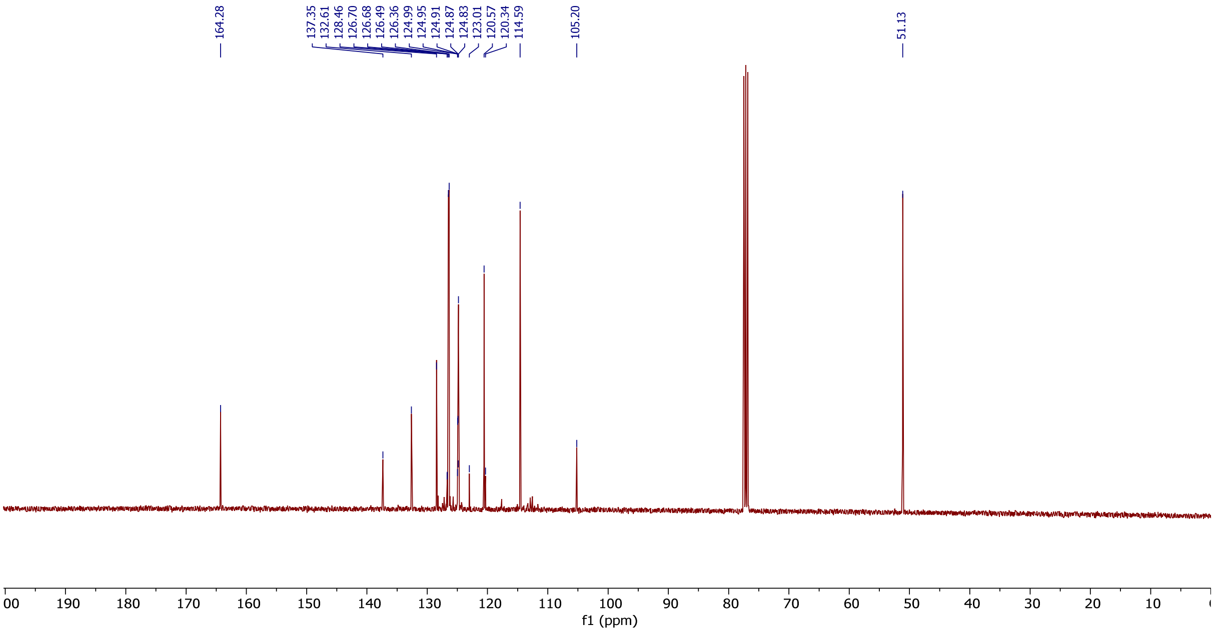

$^{13}C\{^{1}H\}$ NMR (100 MHz, $CDCl_3$) of **2w**.

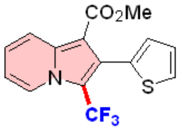

2w

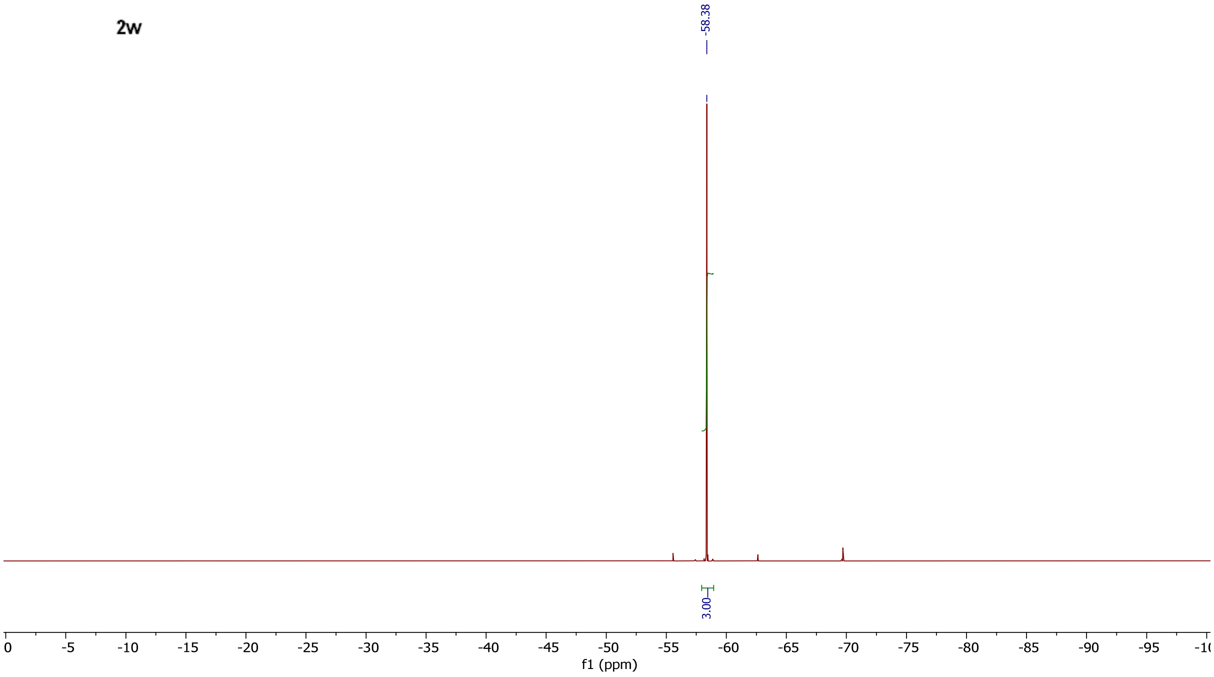

$^{19}F\{^{1}H\}$ NMR (377 MHz, $CDCl_3$) of **2w**.

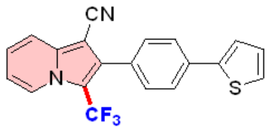

3

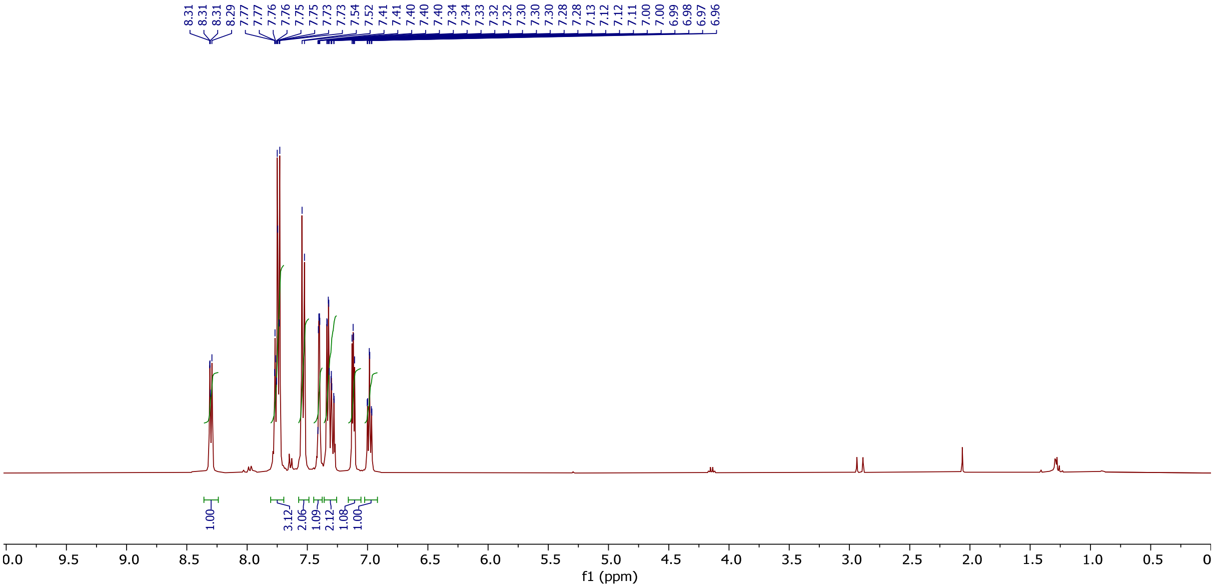

^{1}H NMR (400 MHz, $CDCl_3$) of **3**.

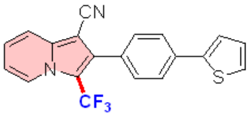

3

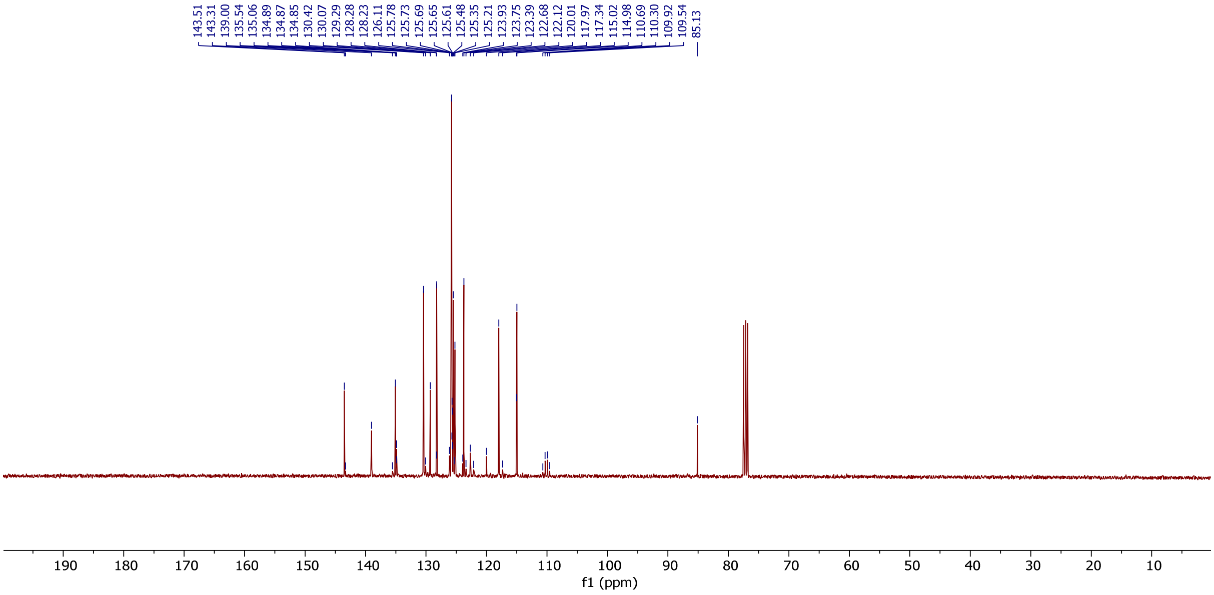

$^{13}C\{^1H\}$ NMR (100 MHz, $CDCl_3$) of **3**.

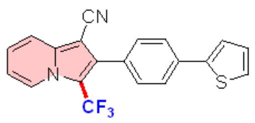

3

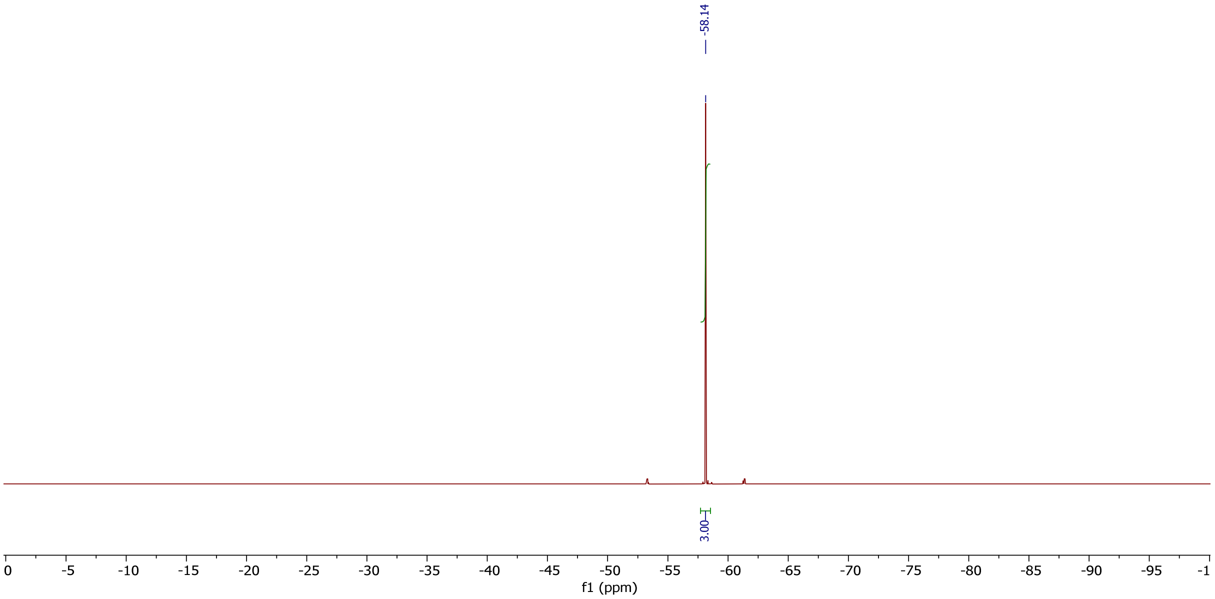

$^{19}F\{^1H\}$ NMR (377 MHz, $CDCl_3$) of **3**.

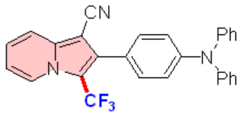

4

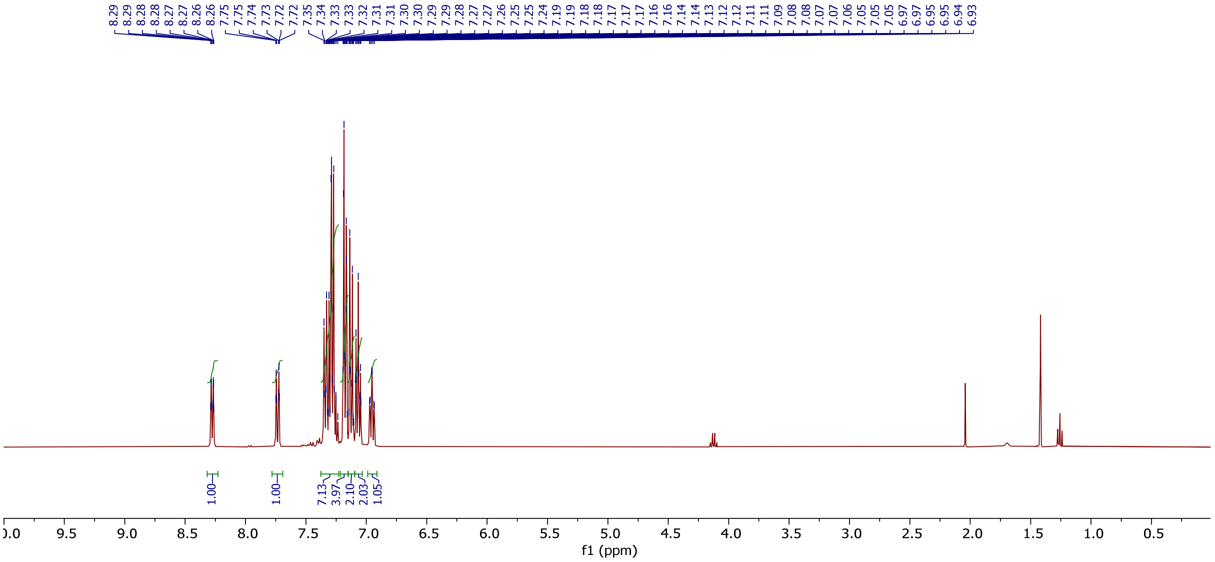

^1H NMR (400 MHz, $CDCl_3$) of **4**.

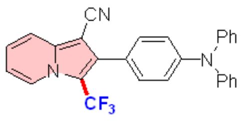

4

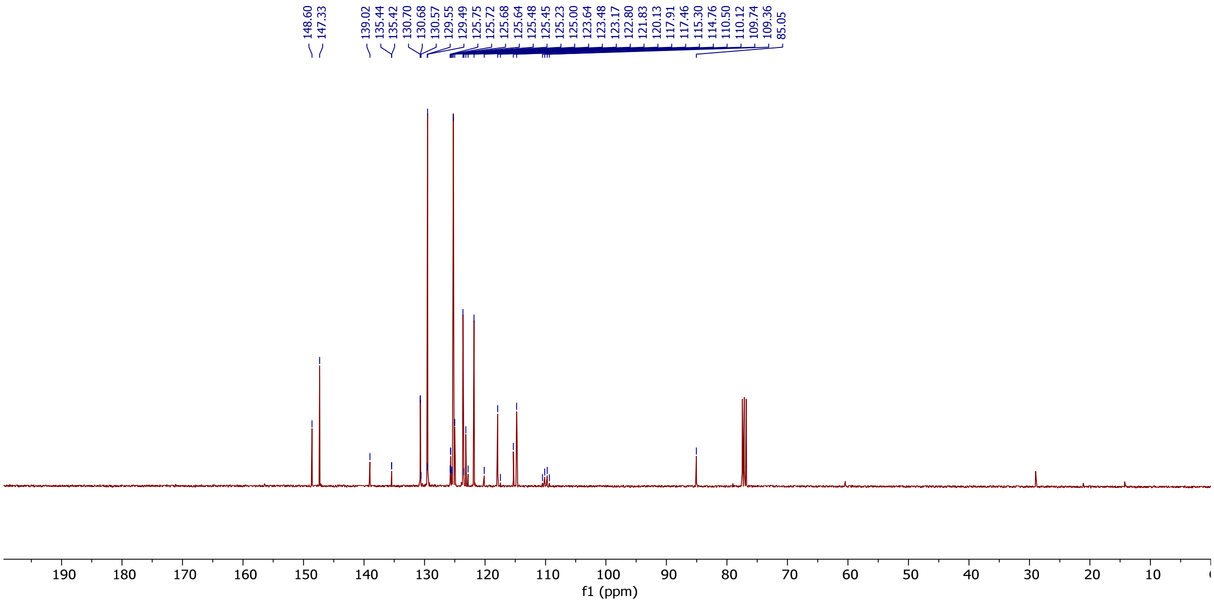

$^{13}C\{^1H\}$ NMR (100 MHz, $CDCl_3$) of **4**.

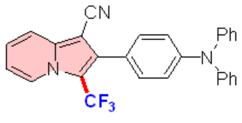

4

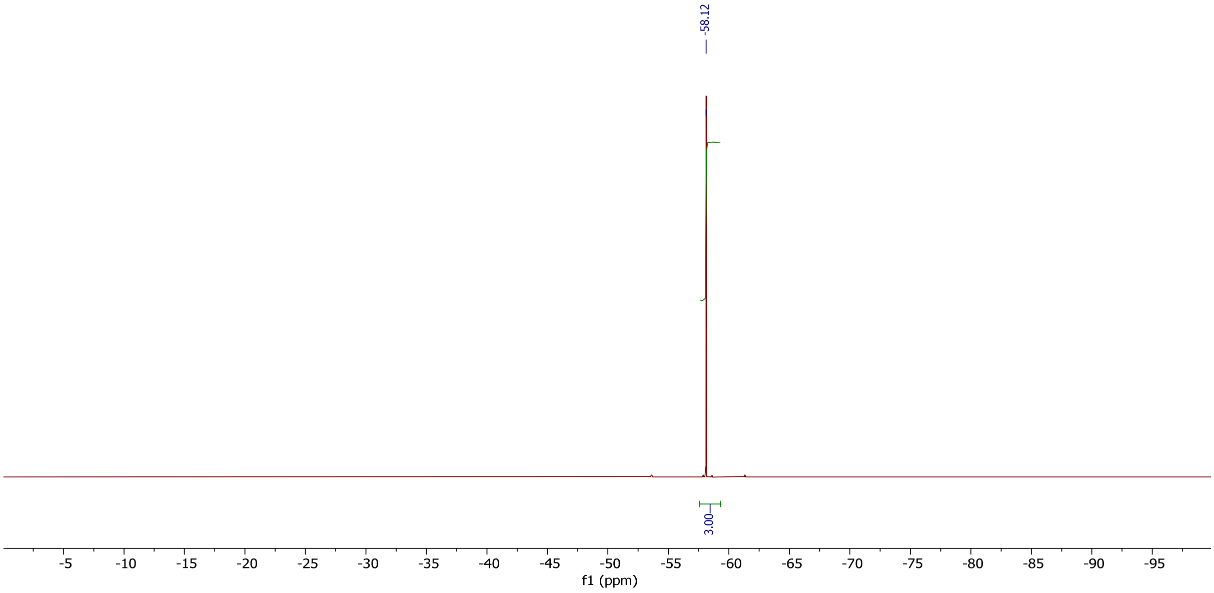

$^{19}F\{^1H\}$ NMR (377 MHz, $CDCl_3$) of **4**.

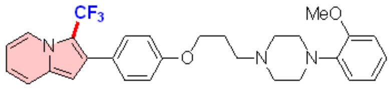

5

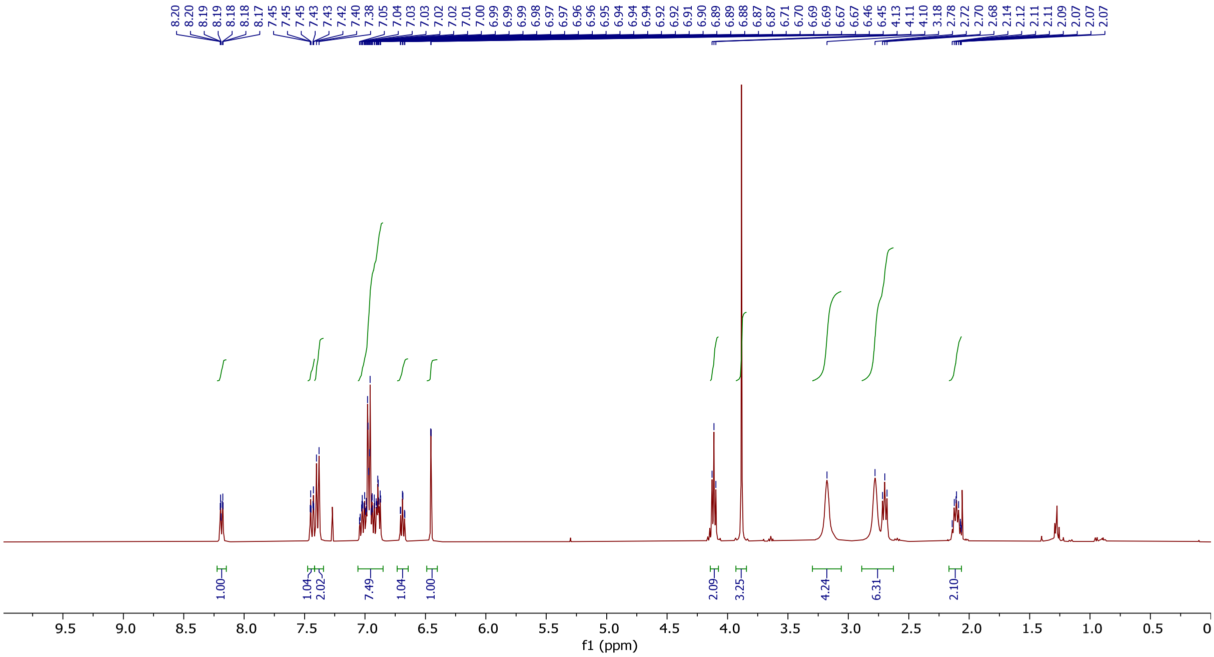

^{1}H NMR (400 MHz, $CDCl_3$) of **5**.

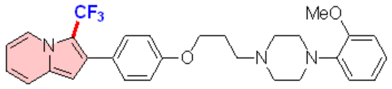

5

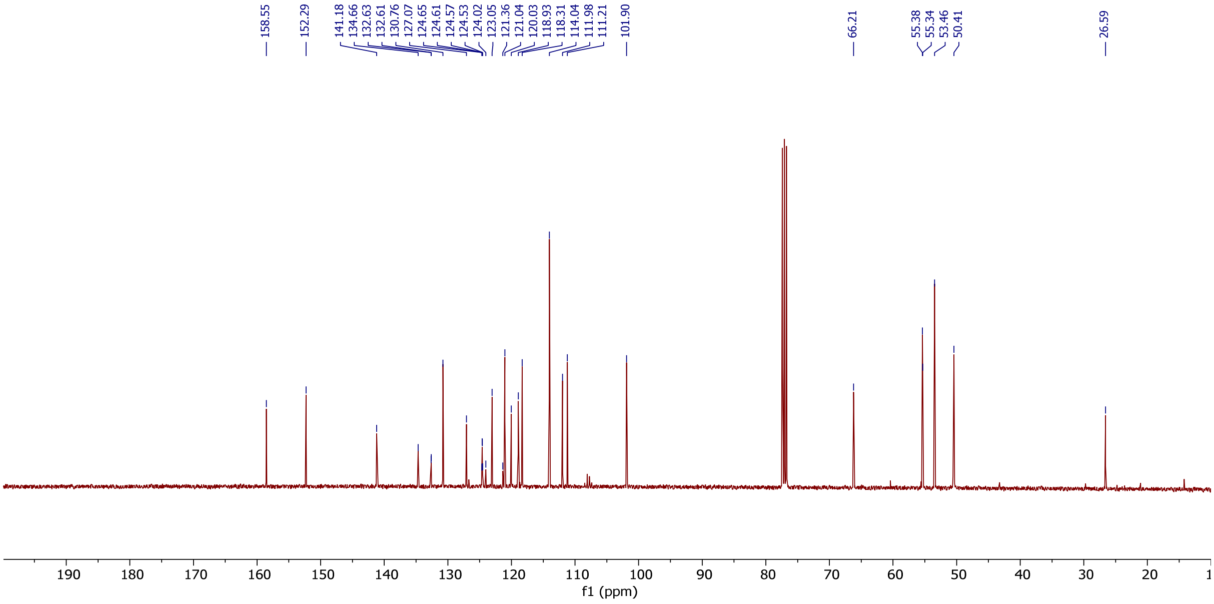

^{13}C{^{1}H} NMR (100 MHz, $CDCl_3$) of **5**.

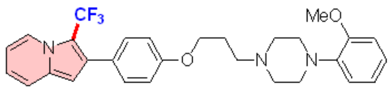

5

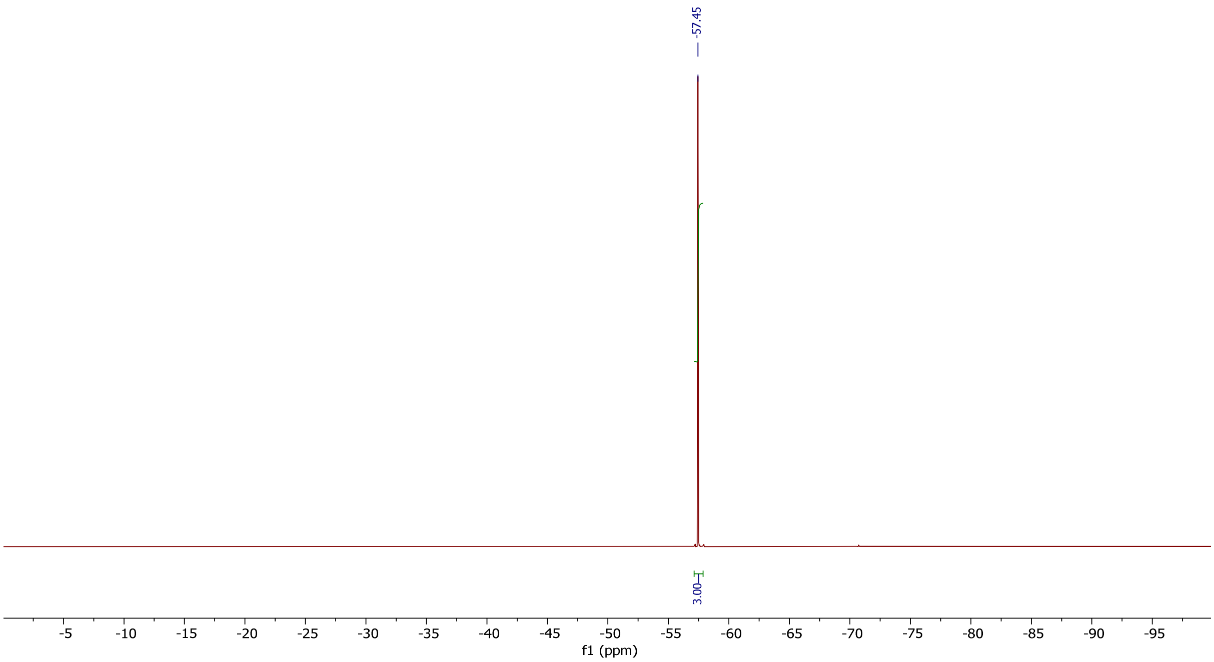

$^{19}F\{^{1}H\}$ NMR (377 MHz, $CDCl_3$) of **5**.

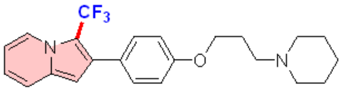

6

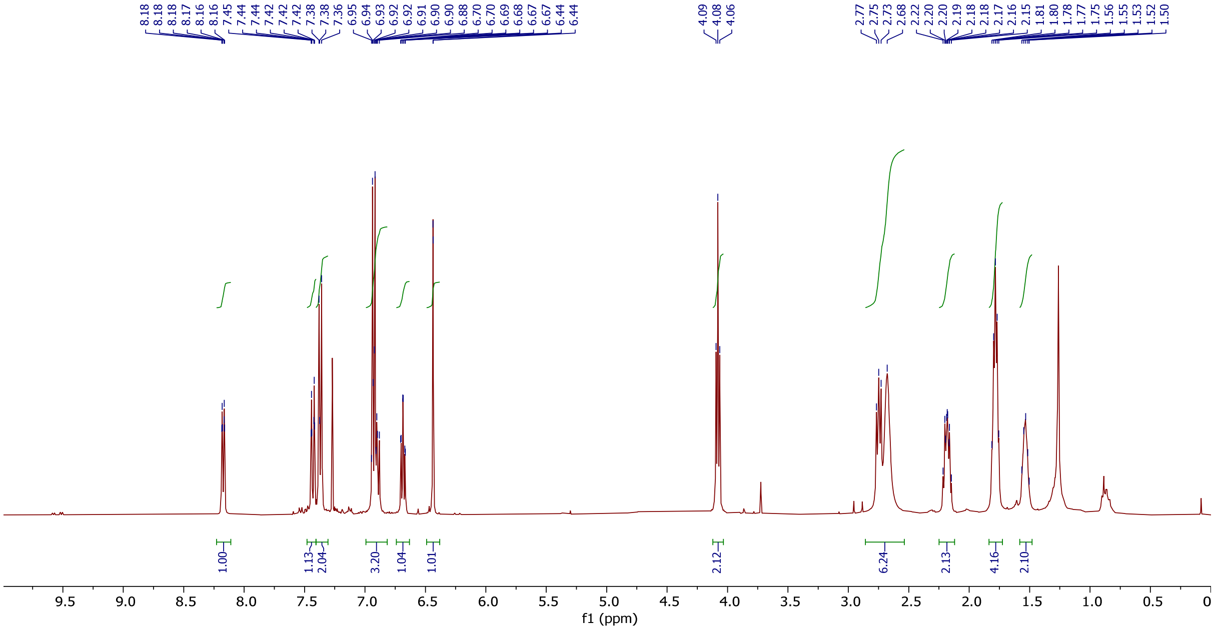

^{1}H NMR (400 MHz, $CDCl_3$) of **6**.

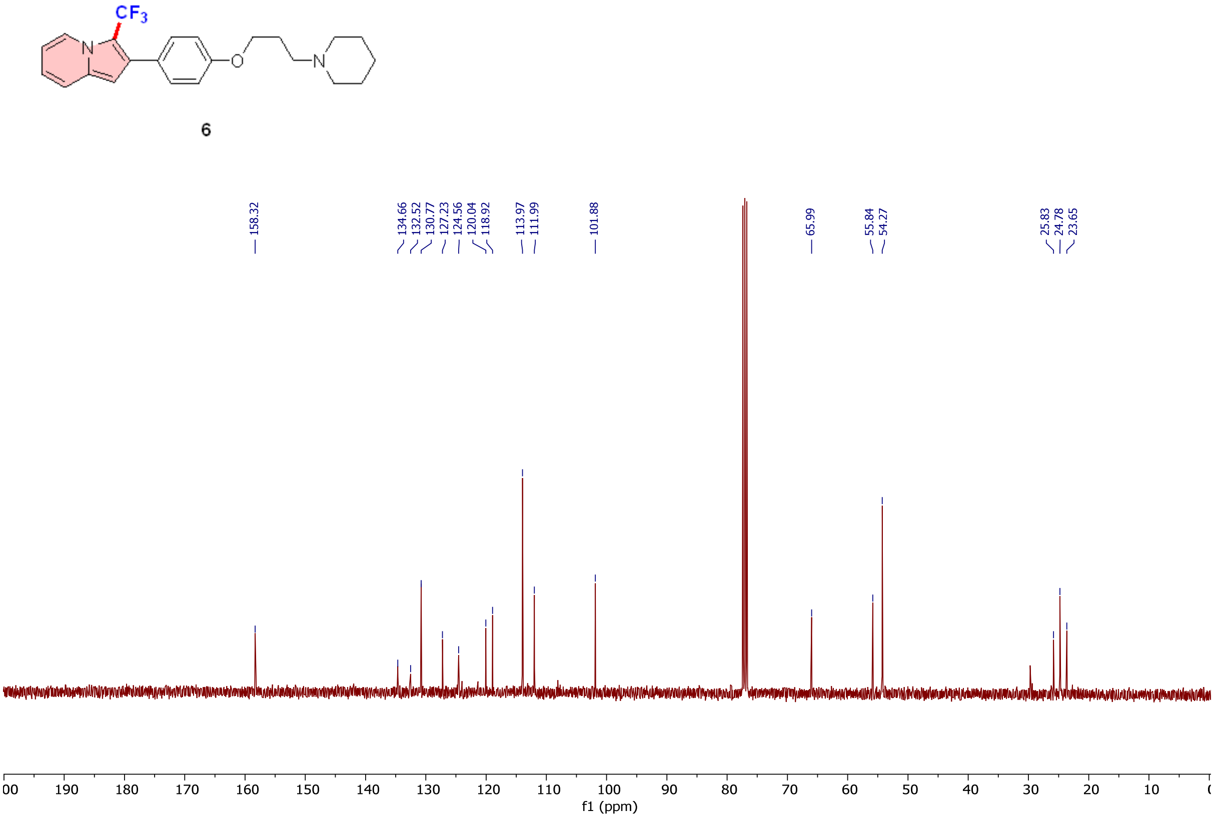

$^{13}C\{^{1}H\}$ NMR (100 MHz, $CDCl_3$) of **6**.

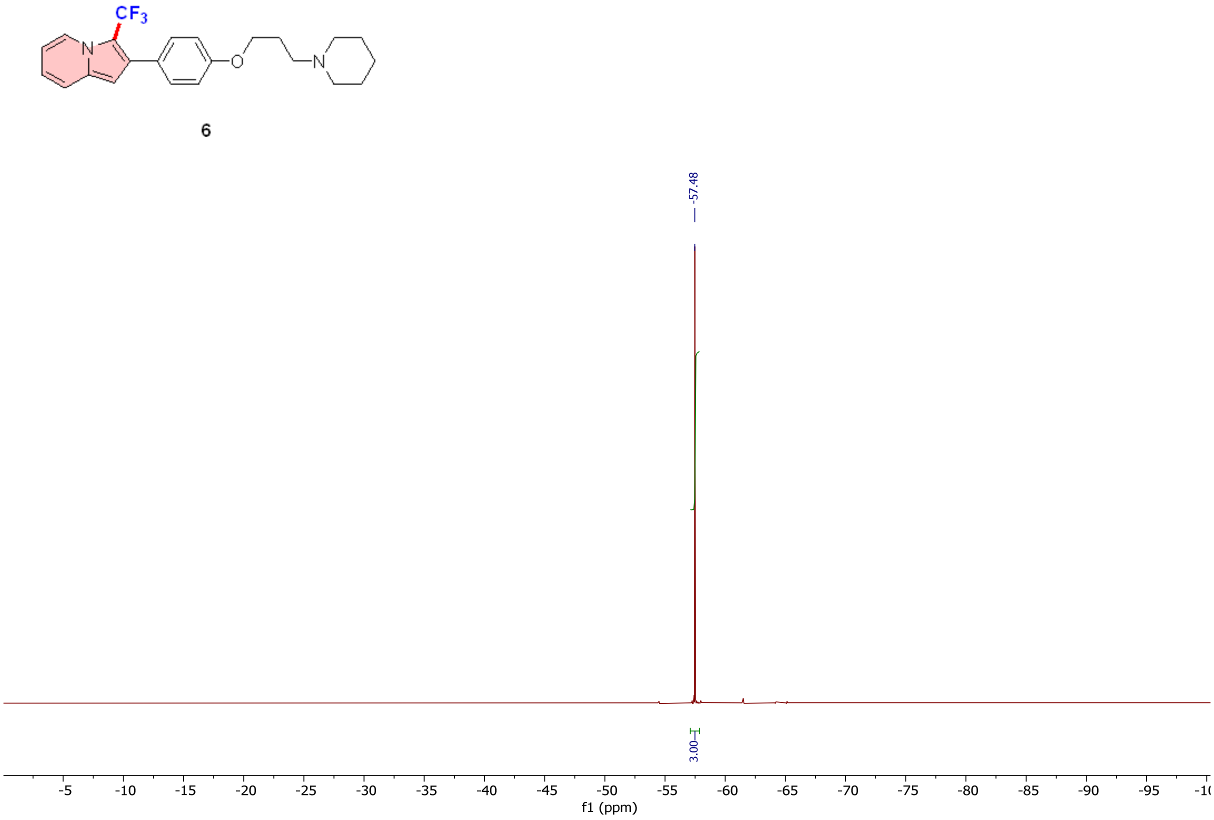

$^{19}F\{^{1}H\}$ NMR (377 MHz, $CDCl_3$) of **6**.

11. References

(1) Müller, C.; Pascher, T.; Eriksson, A.; Chabera, P.; Uhlig, J. KiMoPack: A Python Package for Kinetic Modeling of the Chemical Mechanism. *J. Phys. Chem. A* **2022**, *126* (25), 4087–4099. https://doi.org/10.1021/acs.jpca.2c00907.

(2) Bragg, D. R.; Wibberley, D. G. 609. Indolizines. Part II. Preparation from Ethyl 2-Pyridylacetate and Related Compounds. *J. Chem. Soc. Resumed* **1963**, 3277. https://doi.org/10.1039/jr9630003277.

(3) Li, B.; Chen, Z.; Cao, H.; Zhao, H. Transition-Metal-Free Regioselective Cross-Coupling: Controlled Synthesis of Mono- or Dithiolation Indolizines. *Org. Lett.* **2018**, *20* (11), 3291–3295. https://doi.org/10.1021/acs.orglett.8b01168.

(4) Kim, W.; Kim, H. Y.; Oh, K. Oxidation Potential-Guided Electrochemical Radical–Radical Cross-Coupling Approaches to 3-Sulfonylated Imidazopyridines and Indolizines. *J. Org. Chem.* **2021**, *86* (22), 15973–15991. https://doi.org/10.1021/acs.joc.1c00873.

(5) Zhang, L.; Liang, F.; Sun, L.; Hu, Y.; Hu, H. A Novel and Practical Synthesis of 3-Unsubstituted Indolizines. *Synthesis* **2000**, *2000*, 1733–1737. https://doi.org/10.1055/s-2000-8195.

(6) Baron, A.; Herrero, C.; Quaranta, A.; Charlot, M.-F.; Leibl, W.; Vauzeilles, B.; Aukauloo, A. Efficient Electron Transfer through a Triazole Link in Ruthenium(Ii) Polypyridine Type Complexes. *Chem. Commun.* **2011**, *47* (39), 11011. https://doi.org/10.1039/c1cc13683f.

(7) Norris, M. R.; Concepcion, J. J.; Glasson, C. R. K.; Fang, Z.; Lapides, A. M.; Ashford, D. L.; Templeton, J. L.; Meyer, T. J. Synthesis of Phosphonic Acid Derivatized Bipyridine Ligands and Their Ruthenium Complexes. *Inorg. Chem.* **2013**, *52* (21), 12492–12501. https://doi.org/10.1021/ic4014976.

(8) Anju; Chaturvedi, S.; Chaudhary, V.; Pant, P.; Jha, P.; Kumaran, S. S.; Hussain, F.; Kumar Mishra, A. 5-HT1A Targeting PARCEST Agent DO3AM-MPP with Potential for Receptor Imaging: Synthesis, Physico-Chemical and MR Studies. *Bioorganic Chem.* **2021**, *106*, 104487. https://doi.org/10.1016/j.bioorg.2020.104487.

(9) Umemoto, T.; Zhou, X.; Li, Y. A New Version of Umemoto's Reagents: A Three-Step One-Pot Preparation of 2,3,7,8-Tetrafluoro-S-(Trifluoromethyl)Dibenzothiophenium Triflate. *J. Fluor. Chem.* **2019**, *226*, 109347. https://doi.org/10.1016/j.jfluchem.2019.109347.

(10) Nociarová, J.; Osuský, P.; Rakovský, E.; Georgiou, D.; Polyzos, I.; Fakis, M.; Hrobárik, P. Direct Iodination of Electron-Deficient Benzothiazoles: Rapid Access to Two-Photon Absorbing Fluorophores with Quadrupolar D-π-A-π-D Architecture and Tunable Heteroaromatic Core. *Org. Lett.* **2021**, *23* (9), 3460–3465. https://doi.org/10.1021/acs.orglett.1c00893.

(11) Alatrash, N.; Issa, F. H.; Bawazir, N. S.; West, S. J.; Manen-Brush, K. E. V.; Shelor, C. P.; Dayoub, A. S.; Myers, K. A.; Janetopoulos, C.; Lewis, E. A.; MacDonnell, F. M. Disruption of Microtubule Function in Cultured Human Cells by a Cytotoxic Ruthenium(II) Polypyridyl Complex. *Chem. Sci.* **2019**, *11* (1), 264–275. https://doi.org/10.1039/C9SC05671H.

(12) Bannwarth, C.; Caldeweyher, E.; Ehlert, S.; Hansen, A.; Pracht, P.; Seibert, J.; Spicher, S.; Grimme, S. Extended Tight-Binding Quantum Chemistry Methods. *WIREs Comput. Mol. Sci.* **2021**, *11* (2), e1493. https://doi.org/10.1002/wcms.1493.

(13) Pracht, P.; Bohle, F.; Grimme, S. Automated Exploration of the Low-Energy Chemical Space with Fast Quantum Chemical Methods. *Phys. Chem. Chem. Phys.* **2020**, *22* (14), 7169–7192. https://doi.org/10.1039/C9CP06869D.

(14) Grimme, S. Exploration of Chemical Compound, Conformer, and Reaction Space with Meta-Dynamics Simulations Based on Tight-Binding Quantum Chemical Calculations. *J. Chem. Theory Comput.* **2019**, *15* (5), 2847–2862. https://doi.org/10.1021/acs.jctc.9b00143.

(15) Bohle, F.; Grimme, S. Hydrocarbon Macrocycle Conformer Ensembles and 13C-NMR Spectra. *Angew. Chem. Int. Ed.* **2022**, *61* (14), e202113905. https://doi.org/10.1002/anie.202113905.

(16) Grimme, S.; Bohle, F.; Hansen, A.; Pracht, P.; Spicher, S.; Stahn, M. Efficient Quantum Chemical Calculation of Structure Ensembles and Free Energies for Nonrigid Molecules. *J. Phys. Chem. A* **2021**, *125* (19), 4039–4054. https://doi.org/10.1021/acs.jpca.1c00971.

(17) Neese, F. Software Update: The ORCA Program System, Version 4.0. *WIREs Comput. Mol. Sci.* **2018**, *8* (1), e1327. https://doi.org/10.1002/wcms.1327.

(18) Neese, F. The ORCA Program System. *WIREs Comput. Mol. Sci.* **2012**, *2* (1), 73–78. https://doi.org/10.1002/wcms.81.

(19) Grimme, S.; Ehrlich, S.; Goerigk, L. Effect of the Damping Function in Dispersion Corrected Density Functional Theory. *J. Comput. Chem.* **2011**, *32* (7), 1456–1465. https://doi.org/10.1002/jcc.21759.
(20) Grimme, S.; Antony, J.; Ehrlich, S.; Krieg, H. A Consistent and Accurate Ab Initio Parametrization of Density Functional Dispersion Correction (DFT-D) for the 94 Elements H-Pu. *J. Chem. Phys.* **2010**, *132* (15), 154104. https://doi.org/10.1063/1.3382344.
(21) Weigend, F.; Ahlrichs, R. Balanced Basis Sets of Split Valence, Triple Zeta Valence and Quadruple Zeta Valence Quality for H to Rn: Design and Assessment of Accuracy. *Phys. Chem. Chem. Phys.* **2005**, *7* (18), 3297–3305. https://doi.org/10.1039/B508541A.
(22) Bertrams, M.-S.; Kerzig, C. Converting P-Terphenyl into a Novel Organo-Catalyst for LED-Driven Energy and Electron Transfer Photoreactions in Water. *Chem. Commun.* **2021**, *57* (55), 6752–6755. https://doi.org/10.1039/D1CC01947C.
(23) Müller, P.; Brettel, K. [Ru(Bpy)3]2+ as a Reference in Transient Absorption Spectroscopy: Differential Absorption Coefficients for Formation of the Long-Lived 3MLCT Excited State. *Photochem. Photobiol. Sci.* **2012**, *11* (4), 632–636. https://doi.org/10.1039/c2pp05333k.
(24) Tran, T.-T.; Ha-Thi, M.-H.; Pino, T.; Quaranta, A.; Lefumeux, C.; Leibl, W.; Aukauloo, A. Snapshots of Light Induced Accumulation of Two Charges on Methylviologen Using a Sequential Nanosecond Pump–Pump Photoexcitation. *J. Phys. Chem. Lett.* **2018**, *9* (5), 1086–1091. https://doi.org/10.1021/acs.jpclett.8b00169.
(25) Heinz, L. G.; Yushchenko, O.; Neuburger, M.; Vauthey, E.; Wenger, O. S. Tetramethoxybenzene Is a Good Building Block for Molecular Wires: Insights from Photoinduced Electron Transfer. *J. Phys. Chem. A* **2015**, *119* (22), 5676–5684. https://doi.org/10.1021/acs.jpca.5b03649.
(26) Hankache, J.; Wenger, O. S. Microsecond Charge Recombination in a Linear Triarylamine–Ru(Bpy)32+–Anthraquinone Triad. *Chem. Commun.* **2011**, *47* (36), 10145–10147. https://doi.org/10.1039/C1CC13831F.
(27) Lambert, C.; Nöll, G. The Class II/III Transition in Triarylamine Redox Systems. *J. Am. Chem. Soc.* **1999**, *121* (37), 8434–8442. https://doi.org/10.1021/ja991264s.
(28) Schmitz, M.; Bertrams, M.-S.; Sell, A. C.; Glaser, F.; Kerzig, C. Efficient Energy and Electron Transfer Photocatalysis with a Coulombic Dyad. *J. Am. Chem. Soc.* **2024**, *146* (37), 25799–25812. https://doi.org/10.1021/jacs.4c08551.
(29) Zanzi, J.; Pastorel, Z.; Duhayon, C.; Lognon, E.; Coudret, C.; Monari, A.; Dixon, I. M.; Canac, Y.; Smietana, M.; Baslé, O. Counterion Effects in [Ru(Bpy)3](X)2-Photocatalyzed Energy Transfer Reactions. *JACS Au* **2024**, *4* (8), 3049–3057. https://doi.org/10.1021/jacsau.4c00384.
(30) Farney, E. P.; Chapman, S. J.; Swords, W. B.; Torelli, M. D.; Hamers, R. J.; Yoon, T. P. Discovery and Elucidation of Counteranion Dependence in Photoredox Catalysis. *J. Am. Chem. Soc.* **2019**, *141* (15), 6385–6391. https://doi.org/10.1021/jacs.9b01885.
